# Targetable Brg1‐CXCL14 axis contributes to alcoholic liver injury by driving neutrophil trafficking

**DOI:** 10.15252/emmm.202216592

**Published:** 2023-02-01

**Authors:** Nan Li, Hong Liu, Yujia Xue, Zheng Xu, Xiulian Miao, Yan Guo, Zilong Li, Zhiwen Fan, Yong Xu

**Affiliations:** ^1^ Key Laboratory of Targeted Intervention of Cardiovascular Disease and Collaborative Innovation Center for Cardiovascular Translational Medicine, Department of Pathophysiology Nanjing Medical University Nanjing China; ^2^ Collage of Life Sciences and Institute of Biomedical Research, Liaocheng University Liaocheng China; ^3^ State Key Laboratory of Natural Medicines, Department of Pharmacology China Pharmaceutical University Nanjing China; ^4^ Department of Pathology Nanjing Drum Tower Hospital Affiliated to Nanjing University Medical School Nanjing China

**Keywords:** alcoholic live disease, chemokine, chromatin remodeling protein, neutrophil migration, transcriptional regulation, Digestive System

## Abstract

Alcoholic liver disease (ALD) accounts for a large fraction of patients with cirrhosis and hepatocellular carcinoma. In the present study we investigated the involvement of Brahma‐related gene 1 (Brg1) in ALD pathogenesis and implication in ALD intervention. We report that Brg1 expression was elevated in mouse models of ALD, in hepatocyte exposed to alcohol, and in human ALD specimens. Manipulation of Brg1 expression in hepatocytes influenced the development of ALD in mice. Flow cytometry showed that Brg1 deficiency specifically attenuated hepatic infiltration of Ly6G^+^ neutrophils in the ALD mice. RNA‐seq identified C‐X‐C motif chemokine ligand 14 (CXCL14) as a potential target for Brg1. CXCL14 knockdown alleviated whereas CXCL14 over‐expression enhanced ALD pathogenesis in mice. Importantly, pharmaceutical inhibition of Brg1 with a small‐molecule compound PFI‐3 or administration of an antagonist to the CXCL14 receptor ameliorated ALD pathogenesis in mice. Finally, a positive correlation between Brg1 expression, CXCL14 expression, and neutrophil infiltration was detected in ALD patients. In conclusion, our data provide proof‐of‐concept for targeting the Brg1‐CXCL14 axis in ALD intervention.

## Graphical abstract

Neutrophil homing to the liver constitutes a major pathophysiological process in alcoholic liver disease (ALD) by promoting inflammation, producing ROS, and altering metabolism. The chromatin remodeling protein Brg1 activates transcription of CXCL14 in hepatocytes to drive neutrophil trafficking.

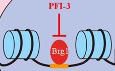

## Introduction

A wide range of sociocultural, neuropsychological, and genetic factors contribute to excessive alcohol consumption (Poikolainen, 2000). It is estimated, based on survey data published by the World Health Organization, that approximately 18% of the adult population is addicted to heavy alcohol use causing over three million premature deaths worldwide (Peacock *et al,* 2018). Alcohol use disorder (AUD), a.k.a. alcoholism, encompasses a series of severe pathologies that include alcoholic liver disease (ALD), alcoholic digestive disease, alcoholic heart disease, alcoholic‐related diabetic complications, alcoholic immune disorders, and alcoholic neurological complications (Mason & Heyser, 2021). In the US alone, 14.5 million people aged 12 and above are affected by AUD incurring an economic burden of 240 billion dollars each year (Friedmann, 2013). ALD, the most prominent AUD, contributes to more than a quarter of all deaths caused by chronic liver diseases such as cirrhosis and hepatocellular carcinoma (Akinyemiju *et al,* 2017). In addition, ALD is implicated in more than 30% of all patients with liver failure thus necessitating liver transplantation (Goldberg *et al,* 2017). Typical pathological characteristics of ALD include steatosis (accumulation of triglycerides and fatty acids in hepatocyte), parenchymal inflammation, and diffuse fibrosis (Celli & Zhang, 2014). Although abstinence is considered the best therapeutic strategy for ALD, the interventional options for those patients with advanced/irreversible liver damages have not evolved in the past decades and therefore are limited (Seitz *et al,* 2018), suggesting the existence of major gaps in our understanding of ALD pathogenesis.

Hepatic homeostasis, or the lack thereof, is acutely influenced by the immune cell populations (Robinson *et al,* 2016). Neutrophils, borne out of the bone marrow, are the most abundant circulating leukocytes in the human body (de Oliveira *et al,* 2016). A cell lineage short lived with rather heterogeneous nature, neutrophils are considered the first line of defense in the innate immune response (Ng *et al,* 2019). It has long been thought that neutrophils are scarcely present in the liver under physiological conditions, a notion verified recently by single‐cell RNA‐seq studies (Zhao *et al,* 2020). On the contrary, multiple injurious stimuli promote the trafficking of circulating neutrophils to the liver where they exert strong pro‐inflammatory effects (Ramaiah & Jaeschke, 2007; Gao *et al,* 2011; Xu *et al,* 2014). Increased neutrophil infiltration is observed in ALD patients and appears to be associated with poor prognosis (Mookerjee *et al,* 2007; Das *et al,* 2017). Typically, neutrophils, under the influence of excessive influx of ethanol, produce a large amount of pro‐inflammatory mediators, hydrolytic proteases, and reactive oxygen species, which in combination cause extensive hepatocellular damages (Lucey *et al,* 2009). The debris of the necroptotic hepatocytes may act as damage‐associated molecular patterns and fuel the activation of innate immunity inside the liver to aggravate the injuries (Cho & Szabo, 2021). Consistent with the pro‐pathogenic role of neutrophils in ALD, it has been independently demonstrated by the Gao group (Bertola *et al,* 2013b) and the Szabo group (Bukong *et al,* 2018) that depletion of neutrophils prior to alcohol drinking markedly attenuated the severity of liver injury in model animals. A host of chemoattractive substances, including complement C5a (Robbins *et al,* 1987), interleukin 8 (Hill *et al,* 1993; Joshi‐Barve *et al,* 2003), and interleukin 17 (Lemmers *et al,* 2009), have been implicated in neutrophil homing to the liver in the pathogenesis of ALD. However, the transcriptional regulation underlying neutrophil trafficking in this context remains largely underexplored.

Brahma‐related gene 1 (Brg1) is the core component of the SWI/SNF chromatin remodeling complex (Khavari *et al,* 1993). Although Brg1 is essential for organogenesis as evidenced by the observation that germline deletion of Brg1 leads to embryonic lethality (Bultman *et al,* 2000, 2005), post‐natal Brg1 deficiency in certain lineages is compatible with a normal life‐span under physiological conditions. We have previously reported that hepatocyte‐restricted Brg1 deletion in mice is associated with amelioration of a range of liver pathologies (Zhang *et al,* 2019; Fan *et al,* 2020; Hong *et al,* 2020; Dong *et al,* 2021; Li *et al,* 2021; Kong *et al,* 2021a). Here we present evidence to implicate Brg1 in ALD pathogenesis through stimulating hepatocyte‐derived chemokine CXCL14 to promote neutrophil infiltration.

## Results

### Alcohol exposure up‐regulates Brg1 expression *in vivo* and *in vitro*

**Figure 1:**
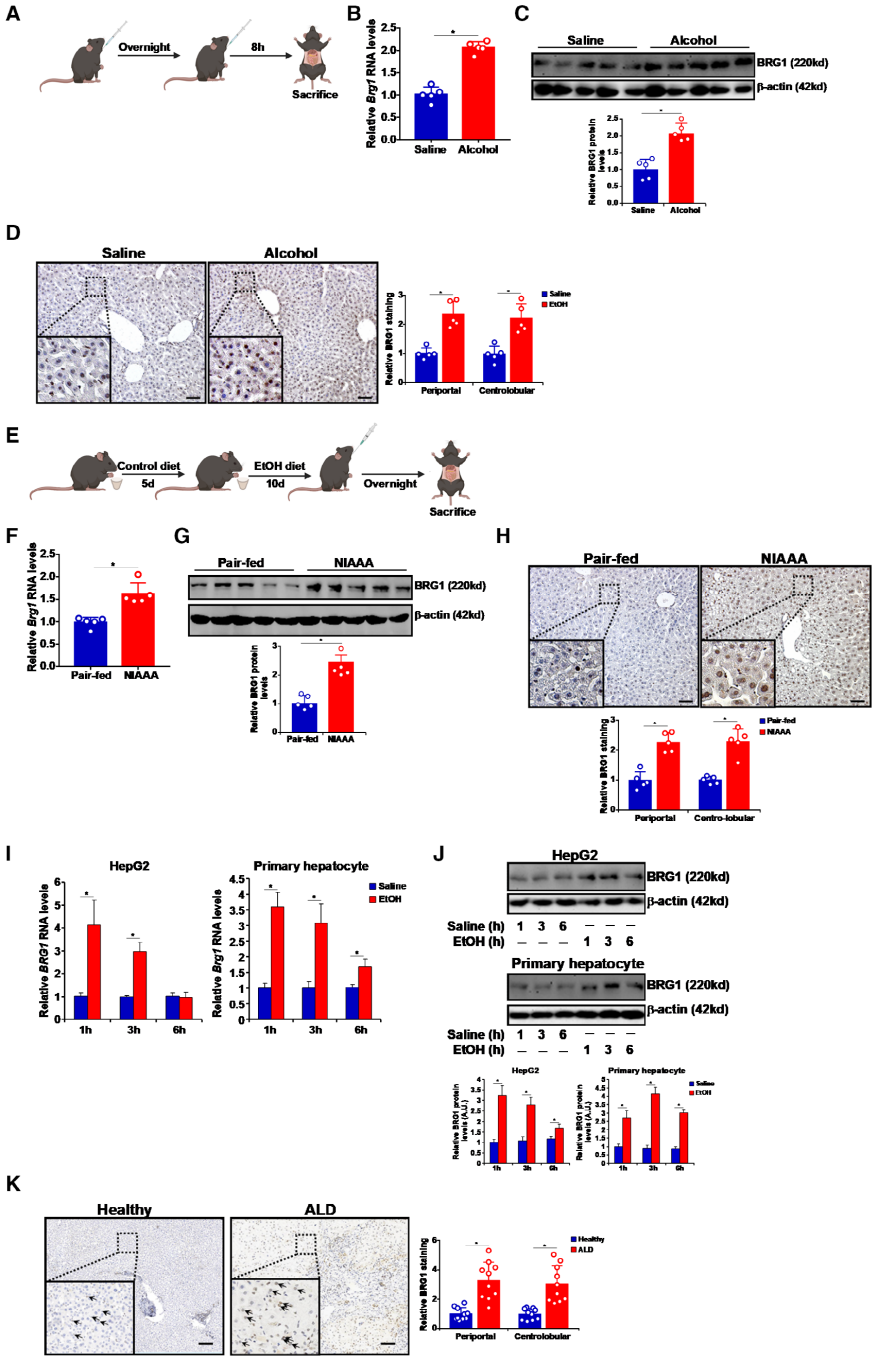
Alcohol exposure up‐regulates Brg1 expression *in vivo* and *in vitro* A–DAlcoholic liver injury was induced in C57/BL6 mice by gavage as described in Methods (A). Brg1 levels were examined by qPCR (B), Western (C), and immunohistochemical staining (D). N = 5 mice for each group. Scale bar, 100 μm.E–HAlcoholic liver injury was induced in C57/BL6 mice by NIAAA feeding as described in Methods (E). Brg1 levels were examined by qPCR (F), Western (G), and immunohistochemical staining (H). N = 5 mice for each group. Scale bar, 100 μm.I, JMouse primary hepatocytes and HepG2 cells were exposed to ethanol (50 mM) and harvested at indicated time points. N = 3 biological replicates.KHuman liver paraffin sections were stained with anti‐BRG1 and quantified by Image Pro. N = 10 for each group. Scale bar, 100 μm. Data information: Data are expressed as mean ± S.D. *P < 0.05, two‐tailed Student's test. Source data are available online for this figure.

In order to implicate Brg1 in the pathogenesis of ALD, response of hepatic Brg1 expression to alcohol exposure was tested in several different models. In the first model the C57/BL6 mice were subjected to acute alcohol consumption via two consecutive gavages of ethanol (5 g/kg) separated by 12 h and sacrificed 8 h after the second gavage (Fig 1A). Quantitative PCR (Fig 1B), Western blotting (Fig 1C), and immunohistochemical staining (Fig 1D) clearly indicated an increase of Brg1 expression in the livers of alcohol‐exposed mice compared to the saline‐exposed mice. In the second model, also known as the NIAAA model (Bertola *et al,* 2013a), the C57/BL6 mice were fed the Lieber–DeCarli ethanol liquid diet for 2 weeks immediately followed by a single dose of alcohol gavage (Fig 1E). Again, hepatic Brg1 expression was up‐regulated as measured by qPCR (Fig 1F), Western blotting (Fig 1G), and immunohistochemical staining (Fig 1H). Next, primary murine hepatocytes and human hepatoma cells (HepG2) were treated with ethanol; exposure to ethanol markedly and transiently stimulated Brg1 expression peaking as early as 1 h after the treatment (Fig 1I and J). Finally, a comparison of Brg1 immunochemical staining performed using paraffin sections of livers from ALD patients and healthy donors revealed that alcohol consumption probably led to up‐regulation of hepatic Brg1 proteins in humans (Fig 1K).

We also investigated the potential mechanism whereby Brg1 expression was up‐regulated in hepatocytes by ethanol exposure. The promoter region of Brg1 (encoded by *Smarca4*) was fused to a reporter gene and transfected into HepG2 cells. Ethanol exposure stimulated the Brg1 promoter activity, indicating that Brg1 might be transcriptionally activated. By comparing the sensitivity of the 1.5 kb reporter construct and the 1 kb reporter construct to ethanol exposure, it was determined that an alcohol‐response element might reside between −1,500 and − 1,000 of the Brg1 promoter relative to the transcription start site (TSS, Appendix Fig S1A). Upon close examination, an E2F1 motif was discovered to locate between −1,076 and − 1,071 of the BRG1 promoter; mutagenesis of this E2F1 motif completely abrogated the response to ethanol treatment (Appendix Fig S1B). Indeed, E2F1 depletion with siRNAs attenuated the up‐regulation of Brg1 expression by ethanol exposure (Appendix Fig S1C). Further, ChIP assay confirmed that E2F1 was recruited to the Brg1 promoter in hepatocytes following ethanol exposure (Appendix Fig S1D). Therefore, we conclude that E2F1 might mediate Brg1 up‐regulation in hepatocytes at the transcriptionally level.

### Manipulation of Brg1 expression influences alcoholic liver injury in mice

**Figure 2:**
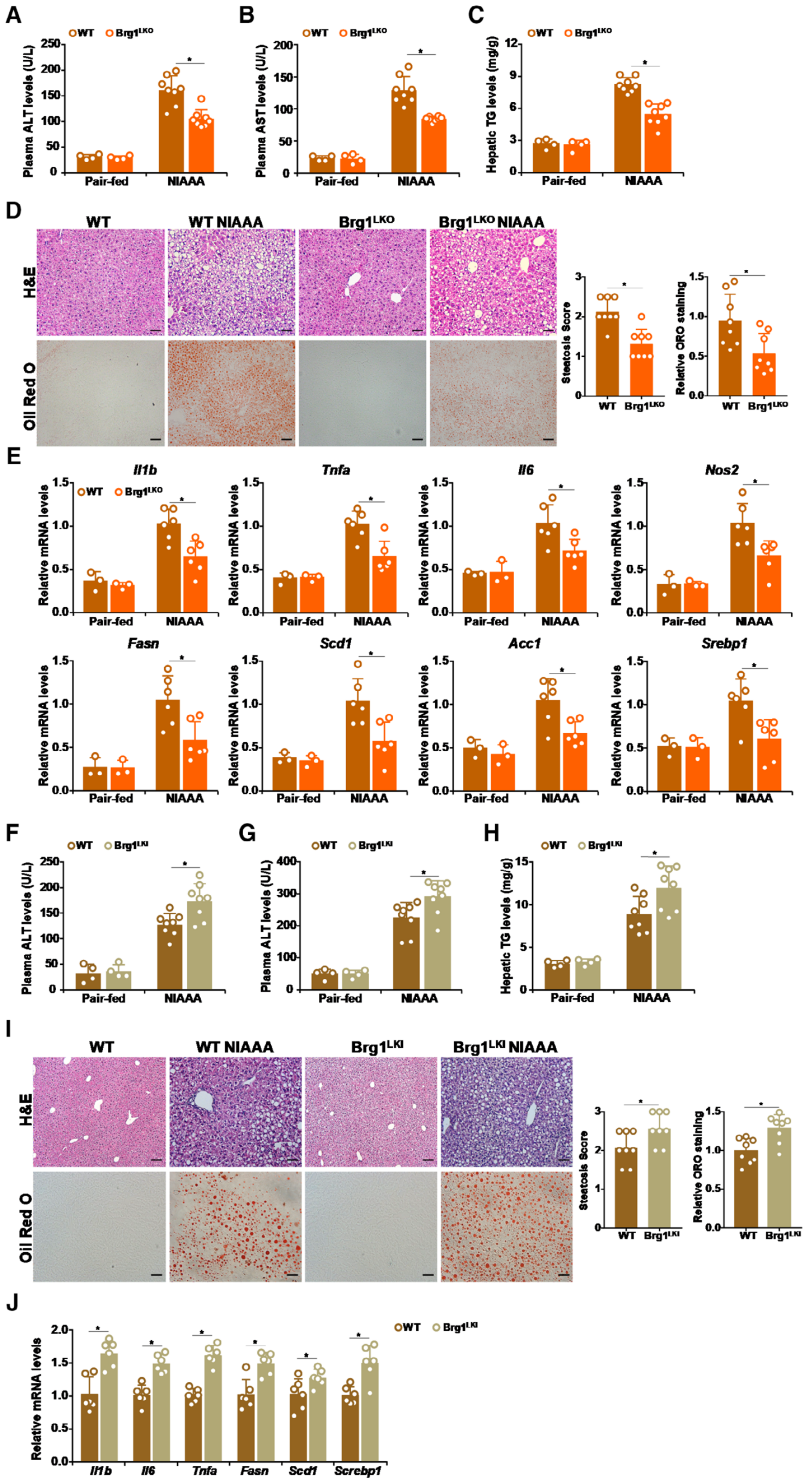
Manipulation of Brg1 expression influences alcoholic liver injury in mice A–EAlcoholic liver injury was induced in WT and Brg1 LKO mice by NIAAA feeding as described in Methods. (A) Plasma ALT levels. (B) Plasma AST levels. (C) Hepatic triglyceride levels. (D) H&E staining and Oil Red O staining. (E) Gene expression levels were examined by qPCR. N = 4–8 mice for each group. Scale bar, 100 μm.F–JAlcoholic liver injury was induced in WT and Brg1 LKI mice by NIAAA feeding as described in Methods. (F) Plasma ALT levels. (G) Plasma AST levels. (H) Hepatic triglyceride levels. (I) H&E staining and Oil Red O staining. (J) Gene expression levels were examined by qPCR. N = 4–8 mice for each group. Scale bar, 100 μm. Data information: Data are expressed as mean ± S.D. *P < 0.05, two‐tailed Student's test. Source data are available online for this figure.

In order to translate the correlation between Brg1 expression in hepatocytes and alcohol exposure into causality, the following experiments were performed in mice harboring hepatocyte‐specific Brg1 deletion or Brg1 over‐expression (Appendix Fig S2 for verification of Brg1 expression in different transgenic strains). In the first set of experiments, alcoholic liver injury was induced in hepatocyte‐specific Brg1 knockout (Brg1^LKO^) mice (Li *et al,* 2019a) and wild type (WT) littermates using the NIAAA model. It was observed that Brg1 deficiency attenuated the increase in liver weight and liver weight/body weight ratio but did not alter body weight, gonadal white adipose tissue (WAT) tissue weight, or WAT weight/body weight ratio in mice (Appendix Fig S3). Plasma ALT (Fig 2A) and AST (Fig 2B) measurements indicated that liver injury was less severe in the Brg1^LKO^ mice than in the WT mice. In addition, less hepatic accumulation of triglycerides was detected in the Brg1^LKO^ mice than in the WT mice (Fig 2C). Histological evaluation of liver sections (H&E staining and oil red O staining) confirmed that there were fewer lipid droplets in the Brg1^LKO^ livers than in the WT livers (Fig 2D). QPCR analysis showed that hepatic expression levels of pro‐inflammatory mediators, including interleukin 1 beta (*Il1b*), interleukin 6 (*Il6*), tumor necrosis factor alpha (*Tnfa*), and inducible NO synthase (*Nos2*), and molecules involved in lipid metabolism, including fatty acid synthase (*Fasn*), stearoyl‐CoA desaturase 1 (*Scd1*), acetyl‐CoA carboxylase 1 (*Acc1*), and sterol response element binding protein 1 (*Screbp1*) were down‐regulated in the Brg1^LKO^ mice compared to the WT mice (Fig 2E). Notably, Brg1 deficiency did not influence alcohol intake or blood alcohol levels in mice (Appendix Fig S4). In addition, expression levels of key enzymes involved in metabolism of ethanol were not significantly altered by Brg1 manipulation in the liver (Appendix Fig S5), suggesting that regulation of ALD by Brg1 is unlikely attributable to alcohol metabolism. Similarly, in an acute gavage model of ALD it was found that the Brg1^LKO^ mice exhibited a less severe phenotype than the WT mice (Appendix Fig S6).

Next, ALD was induced in the hepatocyte‐specific Brg1 over‐expression mice (Brg1^LKI^) (Kong *et al,* 2021a) by the NIAAA procedure. Plasma ALT (Fig 2F) and AST (Fig 2G) measurements suggested that Brg1 over‐expression enhanced alcoholic liver injury. In accordance, biochemical quantification (Fig 2H) and histological evaluation (Fig 2I) demonstrated an acceleration of lipid accumulation in the Brg1^LKI^ livers than in the WT livers. Additionally, qPCR analysis showed a trend of up‐regulation in the expression levels of pro‐inflammatory/pro‐lipogenic genes (Fig 2J). Taken together, these data link Brg1 manipulation in hepatocytes to altered ALD phenotype in mice.

### Brg1 deficiency attenuates leukocyte trapping in the liver

**Figure 3:**
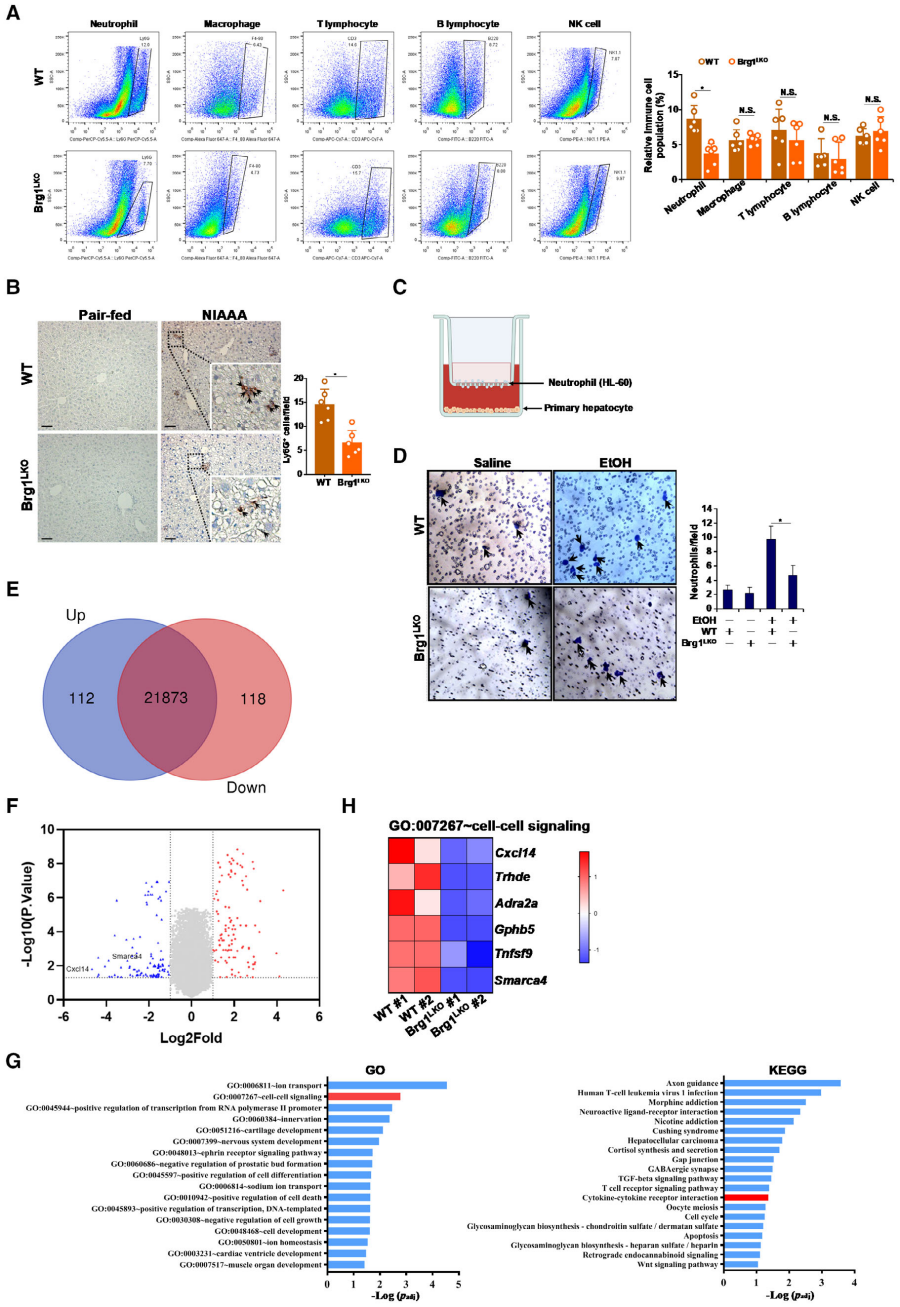
Brg1 deficiency attenuates leukocyte trapping in the liver AAlcoholic liver injury was induced in WT and LKO mice by NIAAA feeding as described in Methods. Flow cytometry was performed as described in Methods. N = 6 mice for each group. Data are expressed as mean ± S.D. *P < 0.05, two‐tailed Student's test.BImmunohistochemical staining of paraffin section with anti‐Ly6G. N = 6 mice for each group. Scale bar, 100 μm. Arrows, Ly6G^+^ neutrophils. Data are expressed as mean ± S.D. *P < 0.05, two‐tailed Student's test.CA scheme of Boyden chamber transwell assay.DPrimary hepatocytes were isolated from WT and LKO mice and treated with or without EtOH. Transwell assay was performed as described in Methods. N = 3 biological repeats. Data are expressed as mean ± S.D. *P < 0.05, two‐tailed Student's test.E–HAlcoholic liver injury was induced in WT and Brg1 LKO mice by NIAAA feeding as described in Methods. RNA‐seq was performed using liver tissues. Venn diagram (E). Volcano plot (F). GO and KEGG analyses (G). Heat map (H). Source data are available online for this figure.

There is a growing consensus that cells of different immune lineages play an important role in ALD pathogenesis (Zetterman & Sorrell, 1981; Vidali *et al,* 2008; Gao *et al,* 2011). Flow cytometric analysis was performed to evaluate the role of Brg1 on the composition of immune cells in the ALD livers. As shown in Fig 3A, Brg1 deficiency led to a significant decrease in Ly6G^+^ neutrophils in the livers but minimally affected the populations of F4/80^+^ macrophages, CD3^+^ T lymphocytes, B220^+^ B lymphocytes, and NK1.1^+^ NK cells. Immunohistochemical staining detected fewer Ly6G^+^ neutrophils in the Brg1^LKO^ livers than in the WT livers (Fig 3B). In contrast, infiltration of F4/80^+^ macrophages and CD3^+^ lymphocytes was indistinguishable between the WT livers and the Brg1^LKO^ livers (Appendix Fig S7). Based on these observations, we hypothesized that Brg1 might contribute to neutrophil trafficking by modulating hepatocyte‐derived chemoattractive cues. To test this hypothesis, Boyden chamber transwell assay was performed (Fig 3C). Co‐culture with WT hepatocytes exposed to ethanol treatment stimulated neutrophil migration stronger than with Brg1^LKO^ hepatocytes (Fig 3D). Brg1 knockdown in HepaRG cells achieved similar effects by reducing the migration of neutrophils (Appendix Fig S8). On the contrary, more Ly6G^+^ neutrophils were detected in Brg1^LKI^ livers than in the WT livers (Appendix Fig S9).

To determine the nature of the chemoattractive cue that emanates from WT livers but diminishes in the Brg1^LKO^ livers, we compared the liver transcriptomics of two WT mice subjected to the NIAAA procedure and two Brg1^LKO^ mice subjected to the NIAAA procedure. RNA‐seq data revealed that over 100 genes were differentially expressed between the two groups (Fig 3E and F). Enrichment analysis suggested that cell–cell communication pathways were among those most influenced by Brg1 deficiency (Fig 3G). C‐X‐C motif ligand chemokine 14 (CXCL14) was detected to trend with Brg1 (Fig 3F and H). QPCR analysis indicated that NIAAA diet feeding led to robust induction of CXCL14 expression in liver parenchymal cells (PCs) but not in non‐parenchymal cells (NPCs), indicating that hepatocytes might be the major source from which CXCL14 is derived during ALD pathogenesis (Appendix Fig S10). Although several other genes appeared to be down‐regulated by Brg1 deletion (Fig 3H), none possess clear chemotactic activities. In addition, expression levels of CXCL1, a chemokine known to regulate neutrophil migration, were comparable between the WT group and the Brg1^LKO^ group (Appendix Fig S11). Because of the well‐established role of CXCL chemoattractants in regulating immune cell trafficking (Griffith *et al,* 2014), we focused on the regulation of CXCL14 transcription by Brg1 for the remainder of the study.

### Brg1 regulates neutrophil migration by activating CXCL14 transcription

**Figure 4:**
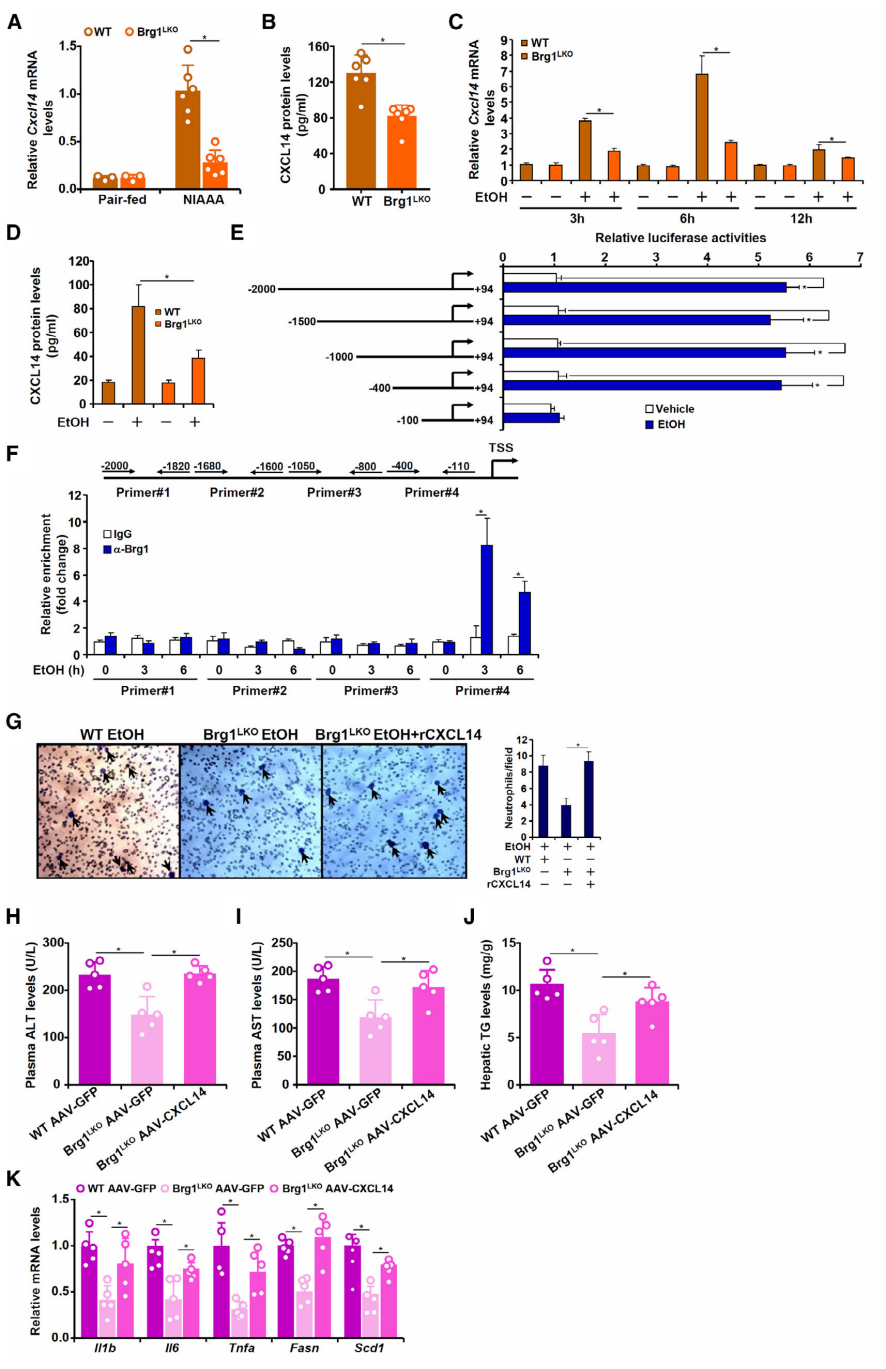
Brg1 regulates neutrophil migration by activating CXCL14 transcription A, BAlcoholic liver injury was induced in WT and Brg1 LKO mice by NIAAA feeding as described in Methods. CXCL14 levels were examined by qPCR and ELISA. N = 3–6 mice for each group. Data are expressed as mean ± S.D. *P < 0.05, one‐way ANOVA with post‐hoc Scheff'e.CPrimary Mouse primary hepatocytes were exposed to ethanol (50 mM) and harvested at indicated time points. CXCL14 levels were examined by qPCR. N = 3 biological replicates. Data are expressed as mean ± S.D. *P < 0.05, one‐way ANOVA with post‐hoc Scheff'e.DPrimary Mouse primary hepatocytes were exposed to ethanol (50 mM) for 6 h. CXCL14 levels in the media were examined by ELISA. N = 3 biological replicates. Data are expressed as mean ± S.D. *P < 0.05, one‐way ANOVA with post‐hoc Scheff'e.ECXCL14 promoter constructs of different lengths was transfected into HepG2 cells with or without Brg1 followed by treatment with ethanol. Luciferase activities were normalized by protein concentration and GFP fluorescence. N = 3 biological replicates. Data are expressed as mean ± S.D. *P < 0.05, two‐tailed Student's test.FPrimary Mouse primary hepatocytes were exposed to ethanol (50 mM) and harvested at indicated time points. ChIP assays were performed with anti‐Brg1 or IgG. N = 3 biological replicates. Data are expressed as mean ± S.D. *P < 0.05, two‐tailed Student's test.GPrimary hepatocytes isolated from WT and Brg1 LKO mice were exposed to ethanol (50 mM) in the presence or absence of recombinant CXCL14. Transwell assay was performed as described in Methods. N = 3 biological repeats. Data are expressed as mean ± S.D. *P < 0.05, two‐tailed Student's test. Arrows, migrated neutrophils.H–KWT and Brg1 LKO mice were injected with AAV8‐CXCL14 or AAV8‐GFP followed by NIAAA feeding. Plasma ALT (H) and AST (I) levels. (J) Hepatic triglyceride levels. (K) Gene expression levels were examined by qPCR. N = 5 mice for each group. Data are expressed as mean ± S.D. *P < 0.05, one‐way ANOVA with post‐hoc Scheff'e. Source data are available online for this figure.

We next examined the transcriptional and functional relationship between Brg1 and CXCL14. QPCR (Fig 4A) and ELISA (Fig 4B) assays showed that CXCL14 levels were significantly lower in the NIAAA diet‐challenged Brg1^LKO^ livers than in the WT livers. In contrast, increased CXCL14 levels were detected in the NIAAA diet‐challenged Brg1^LKI^ livers compared to the WT livers (Appendix Fig S12). In response to ethanol treatment, primary hepatocytes isolated from the WT mice produced more CXCL14 molecules than those from the Brg1^LKO^ mice (Fig 4C and D). To determine whether ethanol‐induced CXCL14 occurred at the transcriptional level, the CXCL14 promoter extending ~2 kb from TSS was fused to a reporter and introduced into HepG2 cells via transient transfection. As shown in Fig 4E, ethanol treatment significantly up‐regulated CXCL14 promoter‐reporter activity. In addition, the ethanol response element appeared to reside between −400 and − 100 relative to the TSS as revealed by truncation mutagenesis (Fig 4E). Indeed, ChIP assay demonstrated that Brg1 was recruited to the proximal CXCL14 promoter region, but not to the more distal regions, when hepatocytes were exposed to ethanol treatment (Fig 4F), suggesting that Brg1 could directly bind to the CXCL14 promoter and activate CXCL14 transcription. Binding of Brg1 to the proximal CXCL14 promoter was further validated in the murine livers (Appendix Fig S13).

Transwell assay showed that the addition of recombinant CXCL14 rescued the deficiency in the ability of Brg1‐null hepatocytes to promote neutrophil migration indicating that CXCL14 might be downstream of Brg1 functionally (Fig 4G). To further authenticate the functional relationship between Brg1 and CXCL14 in ALD pathogenesis, AAV‐mediated delivery was exploited to re‐introduce CXCL14 into the Brg1^LKO^ mice. Over‐expression of exogenous CXCL14 largely restored NIAAA diet‐induced liver injury (Fig 4H and I) and lipid accumulation (Fig 4J) in the Brg1^LKO^ mice bringing the levels closer to those observed in the WT mice. Consistently, the expression levels of pro‐inflammatory and pro‐lipogenic genes were up‐regulated in AAV‐CXCL14 infected Brg1^LKO^ mice compared to the AAV‐GFP infected Brg1^LKO^ mice (Fig 4K).

### Manipulation of CXCL14 regulates alcoholic liver injury in mice

**Figure 5:**
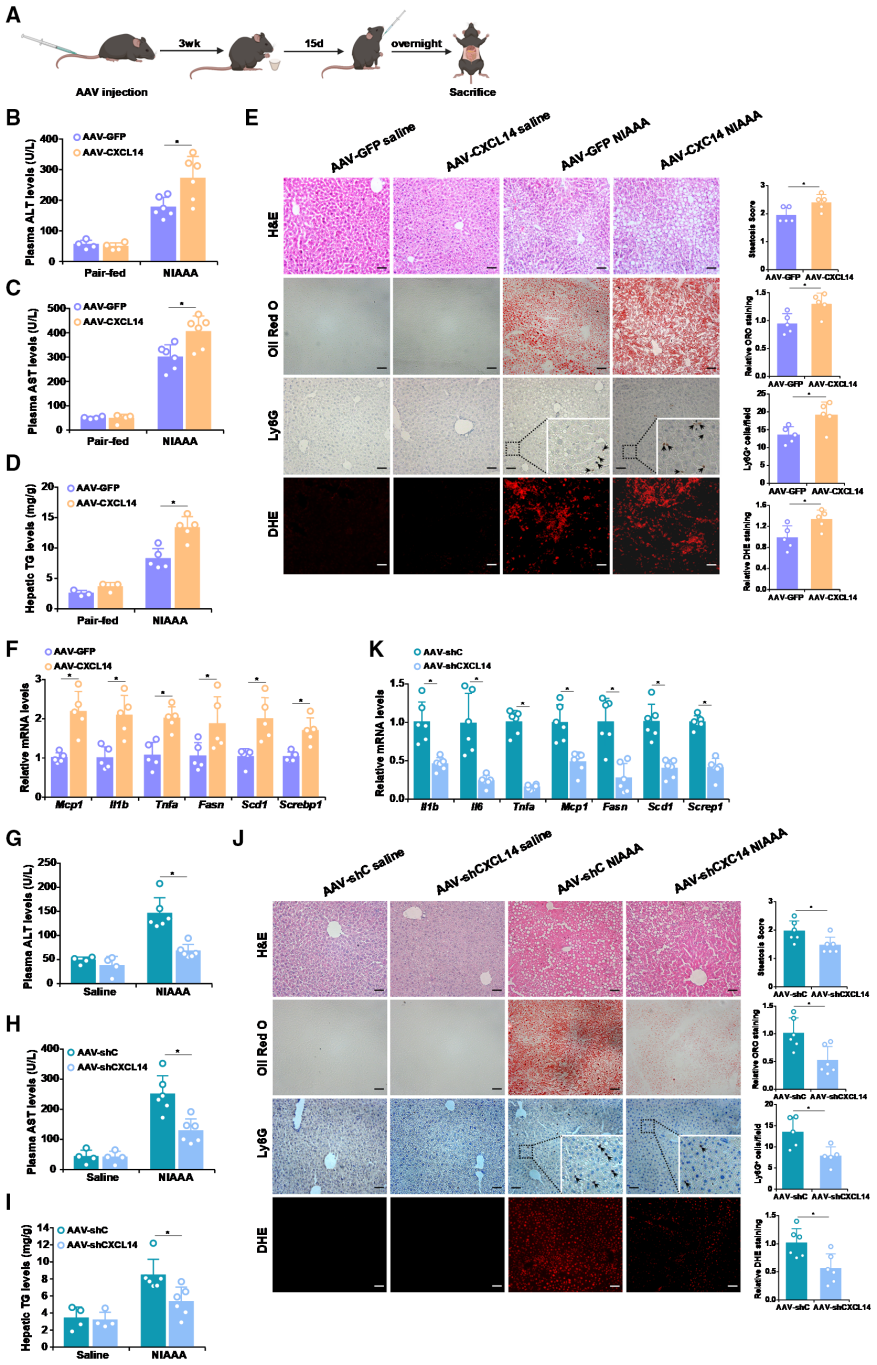
Manipulation of CXCL14 regulates alcoholic liver injury in mice A–FC57/BL6 were injected via tailed AAV8‐CXCL14 or AAV8‐GFP followed by induction of alcoholic liver injury. (A) Scheme of animal protocol. Plasma ALT (B) and AST (C) levels. (D) Hepatic triglyceride levels. (E) Liver sections were stained with H&E, oil red O, DHE, and anti‐Lys6G (left panel). Steatosis score and quantifications of staining (right panel). (F) Gene expression levels were examined by qPCR. N = 3–5 mice for each group. Scale bar, 100 μm. Arrows, Ly6G^+^ neutrophils. Data are expressed as mean ± S.D. *P < 0.05, two‐tailed Student's test.G–KC57/BL6 were injected via tailed AAV8‐CXCL14 or AAV8‐GFP followed by induction of alcoholic liver injury. Plasma ALT (G) and AST (H) levels. (I) Hepatic triglyceride levels. (J) Liver sections were stained with H&E, oil red O, DHE, and anti‐Lys6G (left panel). Steatosis score and quantifications of staining (right panel). (K) Gene expression levels were examined by qPCR. N = 4–6 mice for each group. Scale bar, 100 μm. Arrows, Ly6G^+^ neutrophils. Data are expressed as mean ± S.D. *P < 0.05, two‐tailed Student's test. Source data are available online for this figure.

We then asked whether manipulation of CXCL14 by itself would be sufficient to influence ALD pathogenesis in mice. In the first set of experiments, ectopic CXCL14 was delivered through injection with AAV viral particles followed by ALD induction using the NIAAA procedure (Fig 5A). Levels of CXCL14 over‐expression were verified by qPCR and ELISA (Appendix Fig S14). CXCL14 over‐expression enhanced alcoholic injury as shown by measurements of plasma ALT (Fig 5B), plasma AST (Fig 5C), and hepatic triglyceride (Fig 5D) levels. Histological analyses revealed that AAV‐CXCL14 injection enhanced NIAAA‐induced steatosis, ROS production, and neutrophil infiltration (Fig 5E). In addition, higher levels of pro‐inflammatory/pro‐lipogenic genes were detected in the AAV‐CXCL14 injected livers than in the AAV‐GFP injected livers (Fig 5F).

In the second set of experiments, endogenous CXCL14 was depleted by AAV delivery of targeting shRNA and the knockdown efficiency was confirmed by qPCR and ELISA (Appendix Fig S15). CXCL14 silencing led to an amelioration of alcoholic liver injury as indicated by plasma ALT (Fig 5G), plasma AST (Fig 5H), and hepatic triglyceride (Fig 5I) levels. Moreover, H&E staining, oil red O staining, DHE staining, and histochemical staining with an anti‐Ly6G antibody all pointed to a less severe ALD phenotype owing to CXCL14 knockdown (Fig 5J). QPCR measurements of pro‐inflammatory/pro‐lipogenic gene expression added further support to the notion that CXCL14 might be essential for ALD pathogenesis (Fig 5K). Of note, CXCL14 did not influence the infiltration of F4/80+ macrophages or CD3+ lymphocytes (Appendix Fig S16).

### Targeting the Brg1‐CXCL14 axis ameliorates alcoholic liver injury in mice

**Figure 6:**
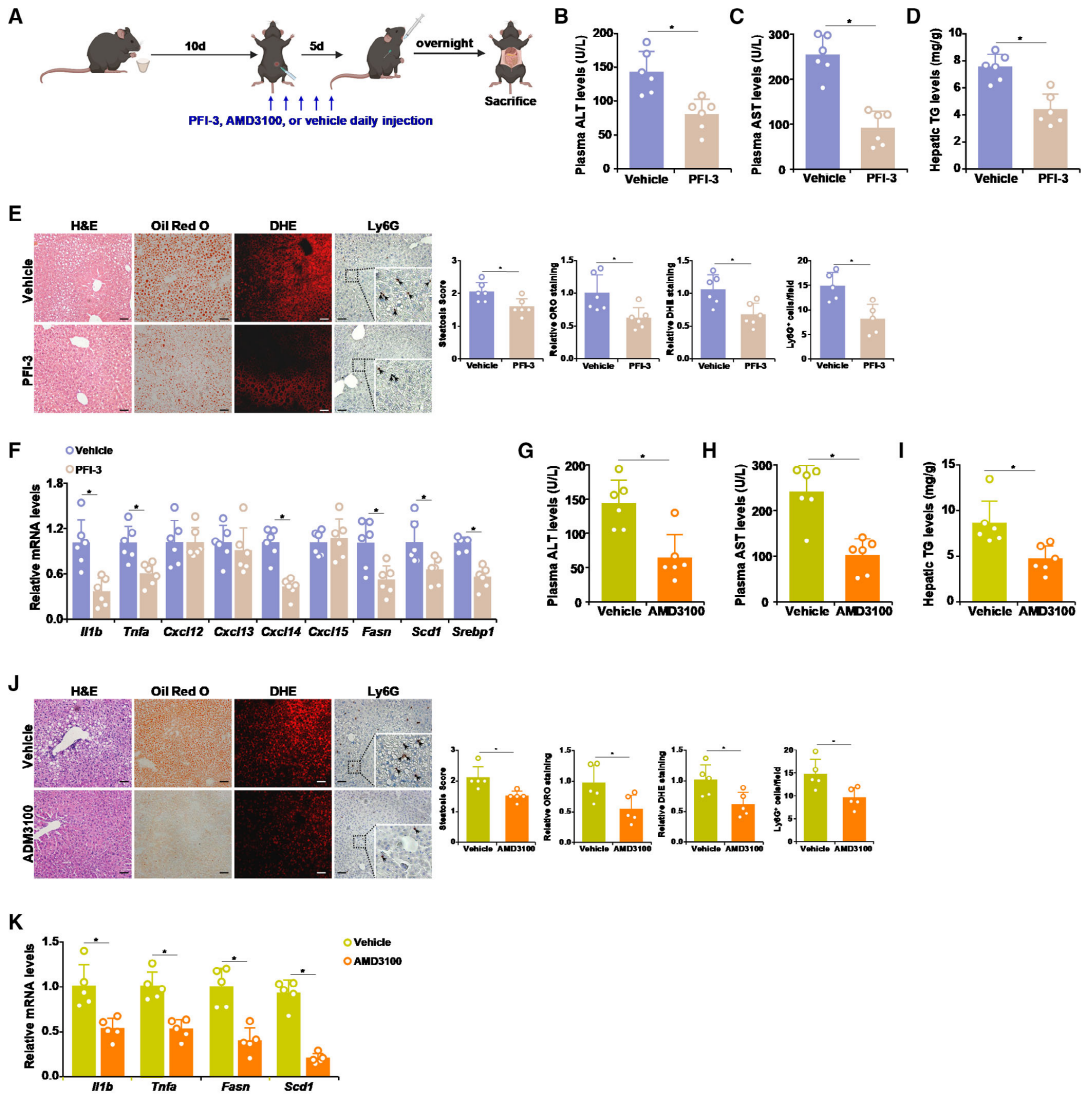
Targeting the Brg1‐CXCL14 axis ameliorates alcoholic liver injury in mice A–FChronic alcoholic liver injury was induced in mice as described in Methods and a Brg1 inhibitor PFI‐3 (5 mg/kg) was administered at day 10. Scheme of protocol (A). Plasma ALT (B) and AST (C) levels. Hepatic triglyceride levels (D). Liver sections were stained with H&E, oil red O, DHE, and anti‐Lys6G (E). Gene expression was measured by qPCR (F). Scale bar, 100 μm. Arrows, Ly6G^+^ neutrophils.G–KChronic alcoholic liver injury was induced in mice as described in Methods and a CXCR4 antagonist AMD3100 (5 mg/kg) was administered at day 10. Plasma ALT (G) and AST (H) levels. Hepatic triglyceride levels (I). Liver sections were stained with H&E, oil red O, DHE, and anti‐Lys6G (J). Gene expression was measured by qPCR (K). Scale bar, 100 μm. Arrows, Ly6G^+^ neutrophils. Data information: N = 6 mice for each group. Data are expressed as mean ± S.D. *P < 0.05, two‐tailed Student's test. Source data are available online for this figure.

Based on the observation that manipulation of either Brg1 expression or CXCL14 expression was associated altered ALD phenotype, we next entertained the idea that small‐molecule compounds that specifically target Brg1 or CXCL14 might be effective in ALD intervention. To test this idea, the mice were induced to develop ALD by the NIAAA procedure followed by injection with a specific Brg1 inhibitor (PFI‐3) (Vangamudi *et al,* 2015; Wu *et al,* 2016; Sharma *et al,* 2021) or a specific antagonist to the CXCL14 receptor (AMD3100) (Salogni *et al,* 2009; Collins *et al,* 2017) (Fig 6A). Administration of PFI‐3 significantly alleviated alcoholic liver injury as evidenced by plasma ALT levels (Fig 6B), plasma AST levels (Fig 6C), and hepatic triglyceride levels (Fig 6D). Further evidence that Brg1 inhibition by PFI‐3 administration could potentially mitigate alcoholic liver injury was provided by histological stainings that showed reduced lipid droplets, ROS production, and neutrophil infiltration in the liver (Fig 6 E). QPCR examination of pro‐inflammatory/pro‐lipogenic gene expression levels further attested to the effectiveness of PFI‐3 administration (Fig 6F). Similarly, CXCL14 blockade by AMD3100 corroborated the finding that CXCL14 is essential for ALD pathogenesis (Fig 6G–K). *In vitro* trans‐well assays confirmed that treatment with either PFI‐3 (Appendix Fig S17) or AMD3100 (Appendix Fig S18) suppressed neutrophil migration. AMD3100 is known to target CXCR4, a receptor for CXCL12. To rule out the involvement of hepatocyte‐derived CXCL12 in neutrophil migration, endogenous CXCL12 was depleted with siRNAs. Notably, CXCL12 depletion did not influence the emission of ethanol‐induced, hepatocyte‐derived chemoattractive cue to promote neutrophil migration (Appendix Fig S19).

### The Brg1‐CXCL14 axis may play a role in alcoholic liver disease in humans

**Figure 7:**
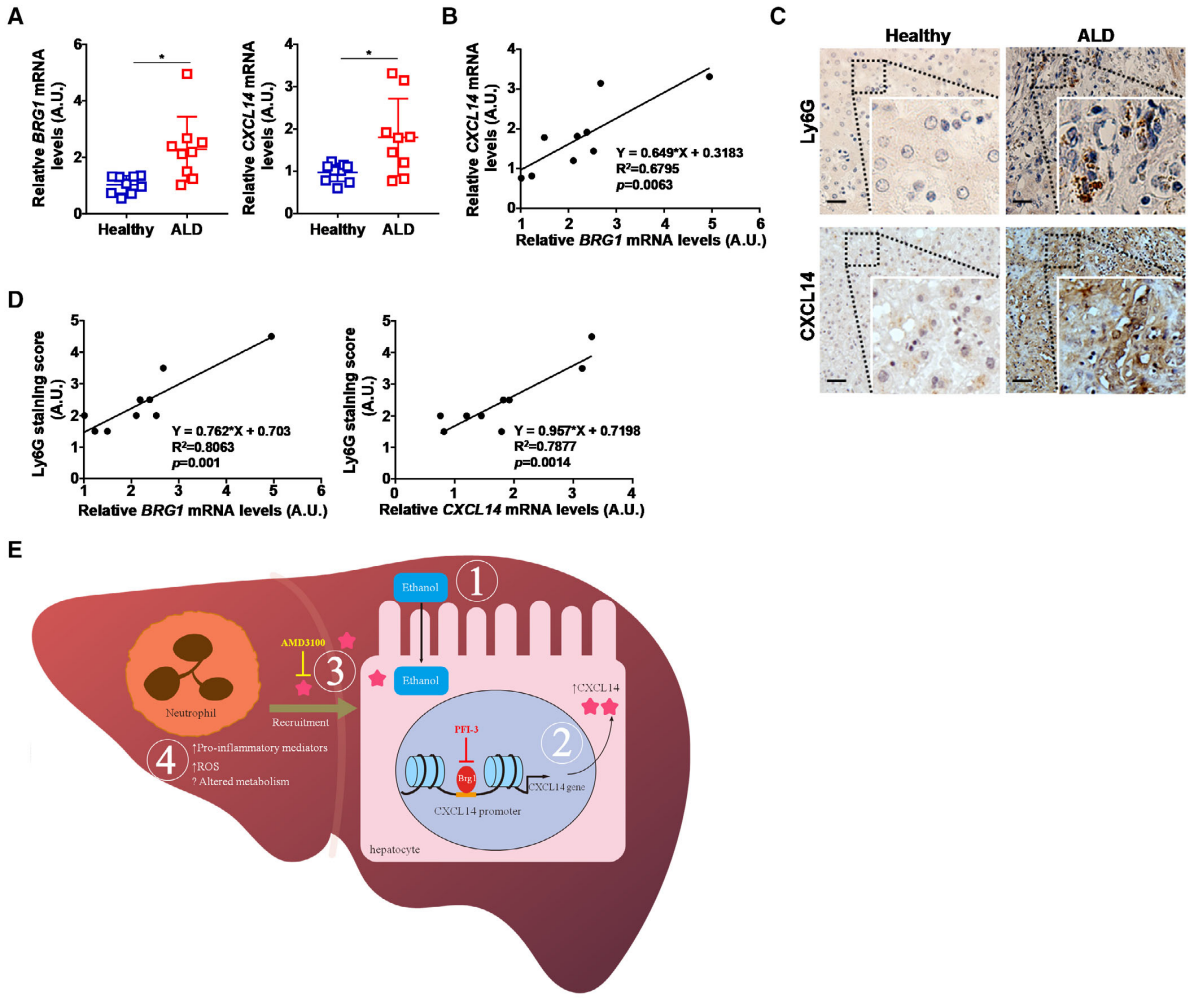
The Brg1‐CXCL14 axis may play a role in alcoholic liver disease in humans BRG1 and CXCL14 expression levels in ALD patients and healthy individuals were examined by qPCR. N = 9 cases for each group. Data are expressed as mean ± S.D. *P < 0.05, two‐tailed Student's test.Linear regression was performed with Graphpad Prism.Representative images of anti‐Ly6G staining and anti‐CXCL14 staining. Scale bar, 100 μm.Linear regression was performed with Graphpad Prism.A schematic diagram. Source data are available online for this figure.

We finally assessed the relevance of the newly identified Brg1‐CXCL14 axis in ALD pathogenesis in humans. QPCR detected significantly higher levels of Brg1 and CXCL14 in the liver specimens from ALD patients compared to healthy individuals (Fig 7A). Positive correlation between Brg1 expression and CXCL14 expression was identified in the ALD specimens (Fig 7B). Using publicly deposited datasets, we were able to confirm the positive correlation between Brg1 expression and CXCL14 expression in the livers of ALD patients (Appendix Fig S20). Further, elevated levels of neutrophil infiltration were revealed by immunohistochemical staining (Fig 7C) and both Brg1 expression and CXCL14 expression were found to be positively correlated with neutrophil infiltration (Fig 7D).

## Discussion

Alcoholic liver disease is the most prevalent form of alcohol use disorder. Herein we describe a novel regulatory axis where the chromatin remodeling protein Brg1 activates the transcription of chemokine CXCL14 to promote neutrophil infiltration and consequently ALD (Fig 7E). Previous studies have shown that Brg1 is able to stimulate the production of multiple hepatocyte‐derived chemoattractive substances to promote homing of different immune cells. In a model of concanavalin A induced fulminant hepatitis, Brg1 deficiency attenuates hepatic infiltration of T lymphocytes owing to reduced production of the chemokine nephronectin (Hong *et al,* 2020). Alternatively, Brg1 deficiency dampens macrophage infiltration by limiting the availability of the chemokine CCL7 (Kong *et al,* 2021a). Of note, Brg1 deficiency did not alter the recruitment of either macrophages or T lymphocytes in the ALD models (Fig 3A). The context‐dependent requirement for Brg1 in modulating the trafficking of specific immune cell sub‐populations remains unclear. Brg1 relies on sequence‐specific transcription factors to be recruited to target promoters and, by extension, participate in the regulation of pathophysiological processes. Therefore, it is possible that certain ethanol‐sensitive transcription factors (TFs) may become rate‐limiting for Brg1 recruitment, CXCL14 trans‐activation and, ultimately, neutrophil homing. Characterization of the proximal CXCL14 promoter reveals several TFs including C/EBPβ (Niu *et al,* 2020) and ETS1 (Komori *et al,* 2010). Both C/EBPβ (Fan *et al,* 2019) and ETS1 (Chen *et al,* 2020) have been indicated as potential binding partners for Brg1. Further, both C/EBPβ (Chen *et al,* 2009) and ETS1 (McMullen *et al,* 2005) have been implicated in ALD pathogenesis. It would be of interest to determine whether mice with hepatocyte‐specific deficiency in C/EBPβ or ETS1 would phenocopy the Brg1^LKO^ mice and exhibit reduced neutrophil infiltration when challenged with alcohol.

Although there is compelling evidence to show that CXCL14 is directly downstream of Brg1 and mediates neutrophil infiltration during ALD pathogenesis, there might be alternative and otherwise indirect mechanism underlying Brg1‐dependent regulation of neutrophil trafficking. For instance, RNA‐seq data (Fig 3H) point to reduced TNFSF9 (also known as CD137L) expression in the Brg1^LKO^ livers compared to the WT livers. Several reports have suggested that CD137L, along with its cognate receptor CD137, can be involved in reverse signaling to regulate chemotaxis. Of interest, Kim *et al* (2012) have shown that CD137L expression in tubular epithelial cells can induce the synthesis of CXCL1 and CXCL2, which in turn promote neutrophil chemotaxis in a murine model of renal ischemia–reperfusion injury (Kim *et al,* 2012). Another attention‐worthy Brg1‐dependent candidate gene revealed by RNA‐seq data is ADRA2A, which encodes a subunit of adrenoreceptor. Adrenoreceptor signaling is known to modulate the behaviors of neutrophils including migration and chemotaxis (de Coupade *et al,* 2004; Brunskole Hummel *et al,* 2013). Thus, the observation that there was subdued neutrophil infiltration in the Brg1^LKO^ livers may be attributed to impaired adrenoreceptor signaling. Of note, neutrophil infiltration and consequent alcoholic hepatitis are associated with up‐regulation of the adhesion molecule E‐selectin (Bertola *et al,* 2013b), a well characterized transcriptional target of Brg1 (Fang *et al,* 2013). We have previously shown that Brg1 promotes neutrophil migration to the ischemic heart by up‐regulating the transcription of the adhesion molecule podocalyxin (PODXL) in vascular endothelial cells (Zhang *et al,* 2018). It is therefore likely that altered expression of adhesion molecules may contribute to suppression of neutrophil recruitment in the Brg1^LKO^ livers. These lingering possibilities should be further investigated to clarify the mechanistic link between Brg1 and intrahepatic immune cell composition.

Although we establish a connection between Brg1 deletion/inhibition and blockade of neutrophil infiltration, it should be noted that non‐chemotactic role of Brg1 in ALD pathogenesis cannot be ruled out. One of the key pathological characteristics of ALD is the spillover of pro‐inflammatory mediators in the liver (Zhang *et al,* 2018). Tian *et al* (2013) have previously demonstrated that Brg1 mediates palmitate‐induced pro‐inflammatory mediators in cultured hepatocytes by interacting with NF‐κB (Tian *et al,* 2013). Because ethanol exposure can directly elevate the synthesis of pro‐inflammatory mediators in hepatocytes *in vitro* in an NF‐κB‐dependent manner (Szabo *et al,* 2001; Senthil Kumar *et al,* 2012; Chiu *et al,* 2014), we propose that Brg1 may contribute to ALD pathogenesis by, at least in part, by stimulating the production of pro‐inflammatory mediators from hepatocytes. Similarly, ALD patients typically display marked hepatic steatosis indicative of altered lipid metabolism. Mounting evidence suggests a direct role for Brg1 in metabolic reprograming in a range of settings. For instance, we have shown that Brg1, by interacting with SREBP, regulates the transcription of genes involved in fatty acid synthesis and cholesterol synthesis in hepatocytes (Li *et al,* 2018a; Fan *et al,* 2020; Kong *et al,* 2021b). These observations certainly caution the interpretation of the data presented by this report and allude to a more complicated scenario in which multiple Brg1‐dependent but otherwise independent threads collectively contribute to ALD pathogenesis.

There are several issues that deserve further attention. First, it appears counterintuitive that Brg1 deficiency selectively impacts neutrophils despite collective down‐regulation of cytokines/chemokines known to promote trafficking of macrophages (Fig 2E). It is possible that a compensatory mechanism in the absence of hepatocyte Brg1 is activated to promote macrophage infiltration despite low levels of chemokines. Alternatively, an early surge followed by a decline of F4/80+ macrophages has been well documented for a wide range of organ injuries. We did not profile the dynamic changes in macrophage population in the liver during the entire course of ALD pathogenesis. Therefore, the possibility that Brg1 may regulate macrophage infiltration at earlier points during ALD development cannot be conclusively excluded. Second, we observed that targeting CXCL14 appeared to attenuate both neutrophil infiltration and steatosis in mice. It remains unclear whether these two processes occur in tandem or parallel. On the one hand, increased neutrophil infiltration in the liver may promote steatosis by producing reactive oxygen species and pro‐inflammatory mediators to alter metabolism in hepatocytes. Neutrophil depletion by Ly6G blocking antibody has been shown to dampen steatosis in a model of non‐alcoholic fatty liver disease (Gonzalez‐Teran *et al,* 2016). A similar strategy has been exploited by Szabo *et al* (2001) in a model of binge alcohol intake to show that neutrophil depletion alleviates liver inflammation and injury (Bukong *et al,* 2018); it was not determined whether steatosis was also altered in this model. Therefore, it is reasonable to speculate that CXCL14 contributes to steatosis by virtue of promoting neutrophil infiltration. On the other hand, Clement *et al* (2008) have previously reported that adipose tissue‐derived MCP‐1, a classic chemokine, can directly induce lipid accumulation when added to and incubated with hepatocytes in culture (Clement *et al,* 2008), pointing to the possibility that chemokines can possess pro‐lipogenic activities in addition to chemoattractive activities. More studies are warranted to clearly and definitively resolve these lingering issues.

We focused on the role of hepatocyte‐specific Brg1 in ALD pathogenesis in the present study. However, the contribution of non‐parenchymal cell Brg1 in this process cannot be ruled out. For instance, it has been well documented that Brg1 in liver sinusoid endothelial cells (LSECs) is able to regulate liver injury and fibrosis (Li *et al,* 2019b; Dong *et al,* 2020; Shao *et al,* 2020). In addition, Zhou *et al* (2021) have shown that deletion of Brg1 in hepatic progenitor cells (HPCs) attenuates cholangiocarcinoma in mice (Zhou *et al,* 2021). More importantly, it is highly likely that Brg1 may directly regulate neutrophil‐autonomous behaviors to contribute to ALD pathogenesis given the extensive regulatory role of Brg1 in different immune cells (Chaiyachati *et al,* 2013; Bossen *et al,* 2015; Qi *et al,* 2021). Future studies using lineage‐specific Brg1 transgenic mice should help resolve the issue conclusively.

In summary, our data unveil a previously unrecognized role of the Brg1‐CXCL14 in ALD pathogenesis. Most significantly, small‐molecule Brg1 inhibitors and CXCL14 antagonists appear to possess therapeutic potentials in model animals. Since neither Brg1 deletion (Wang *et al,* 2019; Li *et al,* 2019a) or CXCL14 deletion (Nara *et al,* 2007) in adult animals leads to any discernable detrimental phenotype, targeting the Brg1‐CXCL14 axis would presumably yield safe and effective interventional strategies in treating ALD. It should be noted that ALD develops after years, if not decades, of heavy drinking in human patients, the pathophysiology of which is unlikely to be faithfully recapitulated by the current animal model in its entirety. Therefore, more work, ideally employing humanized animal model, is needed before the results of the present study can be translated into clinical applications.

## Materials and Methods

### Animals

All animal protocols were reviewed and approved by the intramural Committee on Ethical Treatment of Experimental Animals. Hepatocyte conditional Brg1 knockout (Brg1^LKO^) mice were generated by cross‐breeding the *Smarca4* ^f/f^ strain with the *Alb*‐Cre strain as previously described (Li *et al,* 2018b). Hepatocyte conditional Brg1 knock‐in (Brg1^LKI^) mice were generated by cross‐breeding the Rosa^Brg1/+^ mice (Liu *et al,* 2019) with the *Alb*‐Cre mice. Alcoholic liver injury was induced in 8‐wk, male mice by oral gavage (Yin *et al,* 2007) or chronic‐plus‐single‐binge ethanol feeding (the National Institute on Alcohol Abuse and Alcoholism model, or the NIAAA model; hereafter referred to as the NIAAA feeding) (Bertola *et al,* 2013a) as previously described. To manipulate CXCL14 expression in mice, murine CXCL14 cDNA or short hairpin RNA (shRNA) targeting murine CXCL14 sequences (GCGAGGAGAAGAUGGUUAUTT) were cloned into the pAAV‐DJ‐CMV vector. Each mouse received a single injection of 100 μl viral particles (1X10^11^GV/ml) through tail vein.

### Cell culture, plasmids, and transient transfection

Human hepatoma cells (HepG2 and HepaRG) and human neutrophil‐like leukemia cells (HL‐60) were maintained in DMEM supplemented with 10% fetal bovine serum (FBS, Hyclone). Primary hepatocytes were isolated and cultured as previously described (Fan *et al,* 2019). Human *CXCL14* promoter‐luciferase construct was generated by amplifying genomic DNA spanning the proximal promoter and the first exon of CXCL14 gene (−2,000/+94) and ligating into a pGL3‐basic vector (Promega). The mouse *Smarca4* promoter‐luciferase constructs were cloned using a similar strategy. Mutant constructs were generated by a QuikChange kit (Thermo Fisher, Waltham, cat# 200514) and verified by direct sequencing. Small interfering RNAs were purchased from Dharmacon. Transient transfections were performed with Lipofectamine 2000. Luciferase activities were assayed 24–48 h after transfection using a luciferase reporter assay system (Promega, cat# E1500).

### Protein extraction and Western blot

Whole cell lysates were obtained by re‐suspending cell pellets in RIPA buffer (50 mM Tris pH7.4, 150 mM NaCl, 1% Triton X‐100) with freshly added protease inhibitor (Roche). Western blot analyses were performed with anti‐BRG1 (Santa Cruz, cat# sc‐10,768, 1:1000) and anti‐β‐actin (Sigma, cat# A2228, 1:5000) antibodies.

### RNA isolation and real‐time PCR

RNA was extracted with the RNeasy RNA isolation kit (Qiagen, cat# 74106). Reverse transcriptase reactions were performed using a SuperScript First‐strand Synthesis System (Invitrogen, cat# 12574026). Real‐time PCR reactions were performed on an ABI Prism 7500 system with the following primers: human *CXCL14*, 5′‐CGCTACAGCGACGTGAAGAA‐3′ and 5′‐GTTCCAGGCGTTGTACCAC‐3′; human *BRG1*, 5′‐TCATGTTGGCGAGCTATTTCC‐3′ and 5′‐GGTTCCGAAGTCTCAACGATG‐3′; mouse *Cxcl14*, 5′‐GAAGATGGTTATCGTCACCACC‐3′ and 5′‐CGTTCCAGGCATTGTACCACT‐3′; mouse *Brg1*, 5′‐CAAAGACAAGCATATCCTAGCCA‐3′ and 5′‐CACGTAGTGTGTGTTAAGGACC‐3′; mouse *Il1b*, 5′‐GCAACTGTTCCTGAACTCAACT‐3′ and 5′‐ATCTTTTGGGGTCCGTCAACT‐3′; mouse *Il6*, 5′‐TGGGGCTCTTCAAAAGCTCC‐3′ and 5′‐AGGAACTATCACCGGATCTTCAA‐3′; mouse *Tnfa*, 5′‐CTGGATGTCAATCAACAATGGGA‐3′ and 5′‐ACTAGGGTGTGAGTGTTTTCTGT‐3′; mouse *Nos2*, 5′‐GTTCTCAGCCCAACAATACAAGA‐3′ and 5′‐GTGGACGGGTCGATGTCAC‐3′; mouse *Mcp1*, 5′‐AAAACACGGGACGAGAAACCC‐3′ and 5′‐ACGGGAACCTTTATTAACCCCT‐3′; mouse *Fasn*, 5′‐GGAGGTGGTGATAGCCGGTAT‐3′ and 5′‐TGGGTAATCCATAGAGCCCAG‐3′; mouse *Scd1*, 5′‐ACTGTGGAGACGTGTTCTGGA‐3′ and 5′‐ACGGGTGTCTGGTAGACCTC‐3′; mouse *Acc1*, 5′‐GCGGCTACAGGGACTATACTG‐3′ and 5′‐CGGAAGTAAGAGCTACTAGCGG‐3′; mouse *Srebp1*, 5′‐TGACCCGGCTATTCCGTGA‐3′ and 5′‐CTGGGCTGAGCAATACAGTTC‐3′. Ct values of target genes were normalized to the Ct values of house keeping control gene (18 s, 5′‐CGCGGTTCTATTTTGTTGGT‐3′ and 5′‐TCGTCTTCGAAACTCCGACT‐3′ for both human and mouse genes) using the ΔΔCt method and expressed as relative mRNA expression levels compared to the control group which is arbitrarily set as 1.

### Chromatin immunoprecipitation (ChIP)

Chromatin immunoprecipitation (ChIP) assays were performed essentially as described before (Dong *et al,* 2022). In brief, chromatin in control and treated cells were cross‐linked with 1% formaldehyde. Cells were incubated in lysis buffer (150 mM NaCl, 25 mM Tris pH 7.5, 1% Triton X‐100, 0.1% SDS, 0.5% deoxycholate) supplemented with protease inhibitor tablet and PMSF. DNA was fragmented into ~200 bp pieces using a Branson 250 sonicator. Aliquots of lysates containing 200 μg of protein were used for each immunoprecipitation reaction with 5 μg of anti‐BRG1 (Santa Cruz, cat# sc‐10,768), anti‐E2F1 (Cell Signaling Tech, cat# 3472), or pre‐immune IgG.

### Flow cytometry

Hepatic tissue was perfused with warm HBSS (37°C) containing collagenase IV (500 mg/L), DNase I (50 μg/L), FCS (2%) and BSA (0.6%). After digestion, the tissue was gently teased apart with a sterile blade and incubated in warm collagenase/HBSS solution (37°C) for 15 min with frequent shaking. The cell suspension was collected, filtered, and washed with PBS followed by centrifugation at 50 × g for 2 min. The cell pellets (parenchymal cells) were discarded and supernatants (non‐parenchymal cells, NPCs) were re‐pelleted, washed, and re‐suspended in PBS for flow cytometric analysis using the following antibodies: anti‐Ly6G (Invitrogen, cat# 45–5,931‐30, 1:100), anti‐F4/80 (BD Biosciences, cat# 565853, 1:100), anti‐CD3 (BD Biosciences, cat# 557596, 1:100), anti‐NK1.1 (Invitrogen, Cat# 12–5,941‐83, 1:100), anti‐B220 (BD, cat# 563103, 1:100), anti‐CD45 (Biolegend, cat# 103108, 1:100) as previously described (Daemen *et al,* 2021).

### Neutrophil migration assay

Neutrophil migration was measured using the Boyden chamber inserts (5 μm, Corning cat# 3496). Prior to the assay, HL‐60 cells were differentiated with DMSO (1.3% v/v) for 6 days as previously described (Babatunde *et al,* 2021). The hepatocytes were seeded into the lower chamber and differentiated HL‐60 cells were seeded into the upper chamber. 24 h after seeding, the inserts were lifted using forceps and washed with PBS. The cells on the inside of the inserts were gently removed using moistened cotton swabs and the cells on the lower surface of the membrane were then stained with crystal violet. The inserts were then rinsed with PBS to remove unbound dye and air‐dried. The migrated cells were observed and imaged under a microscope. In certain experiments, recombinant human CXCL14 (20 ng/ml, R&D, cat# 866‐CX‐025) was directly added to the conditioned media. Migrated cells were counted in at least five different fields for each well. The data are expressed relative cell migration compared to the control group which is set arbitrarily as 1. All experiments were performed in triplicates and repeated three times.

### Enzyme‐linked immunosorbent assay

Secreted CXCL14 levels were examined by ELISA using commercially available kits according to vendor′s recommendations (Raybiotech, cat# ELM‐CXCL14‐1).

### RNA sequencing and data analysis

RNA‐seq was performed as previously described (Wu *et al,* 2021). Total RNA was extracted using the TRIzol reagent according to the manufacturer's protocol. RNA purity and quantification were evaluated using the NanoDrop 2000 spectrophotometer (Thermo Scientific, USA). RNA integrity was assessed using the Agilent 2100 Bioanalyzer (Agilent Technologies, Santa Clara, CA, USA). Then the libraries were constructed using TruSeq Stranded mRNA LT Sample Prep Kit (Illumina, San Diego, CA, USA) according to the manufacturer's instructions and sequenced on an Illumina HiSeq X Ten platform and 150 bp paired‐end reads were generated. Raw data (raw reads) of fastq format were firstly processed using Trimmomatic and the low quality reads were removed to obtain the clean reads. The clean reads were mapped to the mouse genome (Mus_musculus.GRCm38.99) using HISAT2. FPKM of each gene was calculated using Cufflinks, and the read counts of each gene were obtained by HTSeqcount. Differential expression analysis was performed using the DESeq (2012) R package. *P* value < 0.05 and fold change > 2 or fold change < 0.5 was set as the threshold for significantly differential expression. Hierarchical cluster analysis of differentially expressed genes (DEGs) was performed to demonstrate the expression pattern of genes in different groups and samples. GO enrichment and KEGG pathway enrichment analysis of DEGs were performed respectively using R based on the hypergeometric distribution. The raw RNA‐seq data have been deposited in the NCBI functional genomics data repository (GSE207090).

### Human specimen collection

Liver biopsies were collected from patients with ALD referring to Nanjing Drum Tower Hospital. Written informed consent was obtained from subjects or families of liver donors. All procedures that involved human samples were approved by the Ethics Committee of Nanjing Drum Tower Hospital and adhered to the World Medical Association (WMA) Declaration of Helsinki and to the Department of Health and Human Services Belmont Report. For inclusion in the study, the patients have to meet the following criteria: (i) daily alcohol consumption > 40 g for at least 30 years; (ii) significantly elevated (3X over normal threshold) levels of AST, ALT, and GGT with a AST/ALT ratio > 2; (iii) pathological score > 6 (based on steatosis/0–4, inflammation/0–4, and fibrosis/0–4). Those who have viral hepatitis, drug‐induced hepatitis, and/or autoimmune hepatitis were excluded. Patient information is summarized in the Appendix Table S1.

### Histology

Histological analyses were performed essentially as described before. Briefly, the paraffin embedded sections were blocked with 10% normal goat serum for 1 h at room temperature and then incubated with an anti‐Ly6G antibody (Abcam, cat# ab238132, 1:100) or anti‐BRG1 antibody (Abcam, cat# ab110641, 1:100). Staining was visualized by incubation with anti‐rabbit secondary antibody and developed with a streptavidin‐horseradish peroxidase kit (Pierce, cat# 21140) for 20 min. Pictures were taken using an Olympus IX‐70 microscope. Slides were observed under a light microscope at high power (X40) by two pathologists independently in a double‐blind fashion. The scoring system was based on the following criterion: the staining intensity was divided into quintiles; the slides with the strongest staining were given a score of 5 (top quintile) whereas the slides with the dimmest staining were given a score of 1 (bottom quintile).

### Statistical analysis

One‐way ANOVA with post‐hoc Scheff'e analyses were performed by SPSS software (IBM SPSS v18.0, Chicago, IL, USA). Unless otherwise specified, values of *P* < 0.05 were considered statistically significant.

## Author contributions

**Nan Li:** Conceptualization; formal analysis; funding acquisition; investigation; methodology; writing – review and editing. **Hong Liu:** Investigation; methodology; writing – review and editing. **Yujia Xue:** Investigation; methodology; writing – review and editing. **Zheng Xu:** Investigation; methodology; writing – review and editing. **Xiulian Miao:** Investigation; methodology; writing – review and editing. **Yan Guo:** Investigation; methodology; writing – review and editing. **Zilong Li:** Conceptualization; funding acquisition; investigation; methodology; writing – review and editing. **Zhiwen Fan:** Conceptualization; funding acquisition; investigation; methodology; writing – review and editing. **Yong Xu:** Conceptualization; data curation; supervision; funding acquisition; writing – original draft; writing – review and editing.

## Disclosure and competing interests statement

The authors declare that they have no conflict of interest.

**The paper explained**

**Problem**

Chronic alcoholic consumption leads to alcohol liver disease (ALD), a prelude to cirrhosis and hepatocellular carcinoma. Brahma‐related gene 1 (Brg1) has been associated with a range of liver pathologies, however, Brg1 function in ALD has not been investigated.

**Results**

In this paper we describe a novel mechanism whereby the chromatin remodeling protein Brg1 contributes to ALD. There is both a correlative and a causal relationship between Brg1 and ALD pathogenesis. Brg1 orchestrates the transcription of CXCL14, a chemokine, in hepatocytes to direct trafficking of neutrophils to the liver leading to ALD. Importantly, Brg1 inhibition or CXCL14 antagonism by small‐molecule compounds attenuates ALD in mice.

**Impact**

These data provide novel insights into ALD pathogenesis and suggest Brg1 and CXCL14 as potential targets for ALD treatment.

## Supporting information



Appendix S1

Source Data for Appendix

Source Data for Figure 1

Source Data for Figure 2

Source Data for Figure 3

Source Data for Figure 4

Source Data for Figure 5

Source Data for Figure 6

Source Data for Figure 7

## Data Availability

RNA‐seq data generated for this study have been deposited in the Gene Expression Omnibus repository with the accession number GSE207090 (https://www.ncbi.nlm.nih.gov/geo/query/acc.cgi?acc=GSE207090).

## References

[emmm202216592-bib-0001] Akinyemiju T , Abera S , Ahmed M , Alam N , Alemayohu MA , Allen C , Al‐Raddadi R , Alvis‐Guzman N , Amoako Y , Artaman A *et al* (2017) The burden of primary liver cancer and underlying etiologies from 1990 to 2015 at the global, regional, and National Level: results from the global burden of disease study 2015. JAMA Oncol 3: 1683–1691 28983565 10.1001/jamaoncol.2017.3055 PMC5824275

[emmm202216592-bib-0002] Babatunde KA , Wang X , Hopke A , Lannes N , Mantel PY , Irimia D (2021) Chemotaxis and swarming in differentiated HL‐60 neutrophil‐like cells. Sci Rep 11: 778 33436661 10.1038/s41598-020-78854-6 PMC7804120

[emmm202216592-bib-0003] Bertola A , Mathews S , Ki SH , Wang H , Gao B (2013a) Mouse model of chronic and binge ethanol feeding (the NIAAA model). Nat Protoc 8: 627–637 23449255 10.1038/nprot.2013.032 PMC3788579

[emmm202216592-bib-0004] Bertola A , Park O , Gao B (2013b) Chronic plus binge ethanol feeding synergistically induces neutrophil infiltration and liver injury in mice: a critical role for E‐selectin. Hepatology 58: 1814–1823 23532958 10.1002/hep.26419 PMC3726575

[emmm202216592-bib-0005] Bossen C , Murre CS , Chang AN , Mansson R , Rodewald HR , Murre C (2015) The chromatin remodeler Brg1 activates enhancer repertoires to establish B cell identity and modulate cell growth. Nat Immunol 16: 775–784 25985234 10.1038/ni.3170 PMC4474778

[emmm202216592-bib-0006] Brunskole Hummel I , Reinartz MT , Kalble S , Burhenne H , Schwede F , Buschauer A , Seifert R (2013) Dissociations in the effects of beta2‐adrenergic receptor agonists on cAMP formation and superoxide production in human neutrophils: support for the concept of functional selectivity. PLoS One 8: e64556 23741338 10.1371/journal.pone.0064556 PMC3669315

[emmm202216592-bib-0007] Bukong TN , Cho Y , Iracheta‐Vellve A , Saha B , Lowe P , Adejumo A , Furi I , Ambade A , Gyongyosi B , Catalano D *et al* (2018) Abnormal neutrophil traps and impaired efferocytosis contribute to liver injury and sepsis severity after binge alcohol use. J Hepatol 69: 1145–1154 30030149 10.1016/j.jhep.2018.07.005 PMC6310218

[emmm202216592-bib-0008] Bultman S , Gebuhr T , Yee D , La Mantia C , Nicholson J , Gilliam A , Randazzo F , Metzger D , Chambon P , Crabtree G *et al* (2000) A Brg1 null mutation in the mouse reveals functional differences among mammalian SWI/SNF complexes. Mol Cell 6: 1287–1295 11163203 10.1016/s1097-2765(00)00127-1

[emmm202216592-bib-0009] Bultman SJ , Gebuhr TC , Magnuson T (2005) A Brg1 mutation that uncouples ATPase activity from chromatin remodeling reveals an essential role for SWI/SNF‐related complexes in beta‐globin expression and erythroid development. Genes Dev 19: 2849–2861 16287714 10.1101/gad.1364105 PMC1315392

[emmm202216592-bib-0010] Celli R , Zhang X (2014) Pathology of alcoholic liver disease. J Clin Transl Hepatol 2: 103–109 26357621 10.14218/JCTH.2014.00010 PMC4521259

[emmm202216592-bib-0011] Chaiyachati BH , Jani A , Wan Y , Huang H , Flavell R , Chi T (2013) BRG1‐mediated immune tolerance: facilitation of Treg activation and partial independence of chromatin remodelling. EMBO J 32: 395–408 23321680 10.1038/emboj.2012.350 PMC3567501

[emmm202216592-bib-0012] Chen YH , Yang CM , Chang SP , Hu ML (2009) C/EBP beta and C/EBP delta expression is elevated in the early phase of ethanol‐induced hepatosteatosis in mice. Acta Pharmacol Sin 30: 1138–1143 19617893 10.1038/aps.2009.109 PMC4006677

[emmm202216592-bib-0013] Chen B , Zhao Q , Xu T , Yu L , Zhuo L , Yang Y , Xu Y (2020) BRG1 activates PR65A transcription to regulate NO bioavailability in vascular endothelial cell. Front Cell Dev Biol 8: 774 32903816 10.3389/fcell.2020.00774 PMC7443572

[emmm202216592-bib-0014] Chiu YH , Tsai JJ , Lin SL , Lin MY (2014) Lactobacillus casei MYL01 modulates the proinflammatory state induced by ethanol in an in vitro model. J Dairy Sci 97: 2009–2016 24485689 10.3168/jds.2013-7514

[emmm202216592-bib-0015] Cho Y , Szabo G (2021) Two faces of neutrophils in liver disease development and progression. Hepatology 74: 503–512 33314193 10.1002/hep.31680 PMC9235297

[emmm202216592-bib-0016] Clement S , Juge‐Aubry C , Sgroi A , Conzelmann S , Pazienza V , Pittet‐Cuenod B , Meier CA , Negro F (2008) Monocyte chemoattractant protein‐1 secreted by adipose tissue induces direct lipid accumulation in hepatocytes. Hepatology 48: 799–807 18570214 10.1002/hep.22404

[emmm202216592-bib-0017] Collins PJ , McCully ML , Martinez‐Munoz L , Santiago C , Wheeldon J , Caucheteux S , Thelen S , Cecchinato V , Laufer JM , Purvanov V *et al* (2017) Epithelial chemokine CXCL14 synergizes with CXCL12 via allosteric modulation of CXCR4. FASEB J 31: 3084–3097 28360196 10.1096/fj.201700013R PMC5472405

[emmm202216592-bib-0018] de Coupade C , Gear RW , Dazin PF , Sroussi HY , Green PG , Levine JD (2004) Beta 2‐adrenergic receptor regulation of human neutrophil function is sexually dimorphic. Br J Pharmacol 143: 1033–1041 15477226 10.1038/sj.bjp.0705972 PMC1575953

[emmm202216592-bib-0019] Daemen S , Chan MM , Schilling JD (2021) Comprehensive analysis of liver macrophage composition by flow cytometry and immunofluorescence in murine NASH. STAR Protoc 2: 100511 33997821 10.1016/j.xpro.2021.100511 PMC8102804

[emmm202216592-bib-0020] Das S , Maras JS , Hussain MS , Sharma S , David P , Sukriti S , Shasthry SM , Maiwall R , Trehanpati N , Singh TP *et al* (2017) Hyperoxidized albumin modulates neutrophils to induce oxidative stress and inflammation in severe alcoholic hepatitis. Hepatology 65: 631–646 27775820 10.1002/hep.28897

[emmm202216592-bib-0021] Dong W , Kong M , Zhu Y , Shao Y , Wu D , Lu J , Guo J , Xu Y (2020) Activation of TWIST transcription by chromatin remodeling protein BRG1 contributes to liver fibrosis in mice. Front Cell Dev Biol 8: 340 32478075 10.3389/fcell.2020.00340 PMC7237740

[emmm202216592-bib-0022] Dong W , Zhu Y , Zhang Y , Fan Z , Zhang Z , Fan X , Xu Y (2021) BRG1 links TLR4 trans‐activation to LPS‐induced SREBP1a expression and liver injury. Front Cell Dev Biol 9: 617073 33816466 10.3389/fcell.2021.617073 PMC8012493

[emmm202216592-bib-0023] Dong W , Kong M , Liu H , Xue Y , Li Z , Wang Y , Xu Y (2022) Myocardin‐related transcription factor a drives ROS‐fueled expansion of hepatic stellate cells by regulating p38‐MAPK signalling. Clin Transl Med 12: e688 35184409 10.1002/ctm2.688 PMC8858634

[emmm202216592-bib-0024] Fan Z , Li N , Xu Z , Wu J , Fan X , Xu Y (2019) An interaction between MKL1, BRG1, and C/EBPbeta mediates palmitate induced CRP transcription in hepatocytes. Biochim Biophys Acta 1862: 194412 10.1016/j.bbagrm.2019.194412 31356989

[emmm202216592-bib-0025] Fan Z , Kong M , Li M , Hong W , Fan X , Xu Y (2020) Brahma related gene 1 (Brg1) regulates cellular cholesterol synthesis by acting as a Co‐factor for SREBP2. Front Cell Dev Biol 8: 259 32500071 10.3389/fcell.2020.00259 PMC7243037

[emmm202216592-bib-0026] Fang F , Chen D , Yu L , Dai X , Yang Y , Tian W , Cheng X , Xu H , Weng X , Fang M *et al* (2013) Proinflammatory stimuli engage brahma related gene 1 and brahma in endothelial injury. Circ Res 113: 986–996 23963727 10.1161/CIRCRESAHA.113.301296 PMC4049295

[emmm202216592-bib-0027] Friedmann PD (2013) Clinical practice. Alcohol use in adults. N Engl J Med 368: 365–373 23343065 10.1056/NEJMcp1204714

[emmm202216592-bib-0028] Gao B , Seki E , Brenner DA , Friedman S , Cohen JI , Nagy L , Szabo G , Zakhari S (2011) Innate immunity in alcoholic liver disease. Am J Physiol Gastrointest Liver Physiol 300: G516–G525 10.1152/ajpgi.00537.2010 PMC3774265 21252049

[emmm202216592-bib-0029] Goldberg D , Ditah IC , Saeian K , Lalehzari M , Aronsohn A , Gorospe EC , Charlton M (2017) Changes in the prevalence of hepatitis C virus infection, nonalcoholic steatohepatitis, and alcoholic liver disease among patients with cirrhosis or liver failure on the waitlist for liver transplantation. Gastroenterology 152: 1090–1099.e1 28088461 10.1053/j.gastro.2017.01.003 PMC5367965

[emmm202216592-bib-0030] Gonzalez‐Teran B , Matesanz N , Nikolic I , Verdugo MA , Sreeramkumar V , Hernandez‐Cosido L , Mora A , Crainiciuc G , Saiz ML , Bernardo E *et al* (2016) p38gamma and p38delta reprogram liver metabolism by modulating neutrophil infiltration. EMBO J 35: 536–552 26843485 10.15252/embj.201591857 PMC4772851

[emmm202216592-bib-0031] Griffith JW , Sokol CL , Luster AD (2014) Chemokines and chemokine receptors: positioning cells for host defense and immunity. Annu Rev Immunol 32: 659–702 24655300 10.1146/annurev-immunol-032713-120145

[emmm202216592-bib-0032] Hill DB , Marsano LS , McClain CJ (1993) Increased plasma interleukin‐8 concentrations in alcoholic hepatitis. Hepatology 18: 576–580 8359798

[emmm202216592-bib-0033] Hong W , Kong M , Qi M , Bai H , Fan Z , Zhang Z , Sun A , Fan X , Xu Y (2020) BRG1 mediates Nephronectin activation in hepatocytes to promote T lymphocyte infiltration in ConA‐induced hepatitis. Front Cell Dev Biol 8: 587502 33553140 10.3389/fcell.2020.587502 PMC7858674

[emmm202216592-bib-0034] Joshi‐Barve S , Barve SS , Butt W , Klein J , McClain CJ (2003) Inhibition of proteasome function leads to NF‐kappaB‐independent IL‐8 expression in human hepatocytes. Hepatology 38: 1178–1187 14578856 10.1053/jhep.2003.50470

[emmm202216592-bib-0035] Khavari PA , Peterson CL , Tamkun JW , Mendel DB , Crabtree GR (1993) BRG1 contains a conserved domain of the SWI2/SNF2 family necessary for normal mitotic growth and transcription. Nature 366: 170–174 8232556 10.1038/366170a0

[emmm202216592-bib-0036] Kim HJ , Lee JS , Kim JD , Cha HJ , Kim A , Lee SK , Lee SC , Kwon BS , Mittler RS , Cho HR *et al* (2012) Reverse signaling through the costimulatory ligand CD137L in epithelial cells is essential for natural killer cell‐mediated acute tissue inflammation. Proc Natl Acad Sci U S A 109: E13–E22 10.1073/pnas.1112256109 PMC3252952 22160719

[emmm202216592-bib-0037] Komori R , Ozawa S , Kato Y , Shinji H , Kimoto S , Hata R (2010) Functional characterization of proximal promoter of gene for human BRAK/CXCL14, a tumor‐suppressing chemokine. Biomed Res 31: 123–131 20460740 10.2220/biomedres.31.123

[emmm202216592-bib-0038] Kong M , Dong W , Zhu Y , Fan Z , Miao X , Guo Y , Li C , Duan Y , Lu Y , Li Z *et al* (2021a) Redox‐sensitive activation of CCL7 by BRG1 in hepatocytes during liver injury. Redox Biol 46: 102079 34454163 10.1016/j.redox.2021.102079 PMC8406035

[emmm202216592-bib-0039] Kong M , Zhu Y , Shao J , Fan Z , Xu Y (2021b) The chromatin remodeling protein BRG1 regulates SREBP maturation by activating SCAP transcription in hepatocytes. Front Cell Dev Biol 9: 622866 33718362 10.3389/fcell.2021.622866 PMC7947303

[emmm202216592-bib-0040] Lemmers A , Moreno C , Gustot T , Marechal R , Degre D , Demetter P , de Nadai P , Geerts A , Quertinmont E , Vercruysse V *et al* (2009) The interleukin‐17 pathway is involved in human alcoholic liver disease. Hepatology 49: 646–657 19177575 10.1002/hep.22680

[emmm202216592-bib-0041] Li N , Li M , Hong W , Shao J , Xu H , Shimano H , Lu J , Xu Y (2018a) Brg1 regulates pro‐lipogenic transcription by modulating SREBP activity in hepatocytes. Biochim Biophys Acta Mol Basis Dis 1864: 2881–2889 29857051 10.1016/j.bbadis.2018.05.022

[emmm202216592-bib-0042] Li N , Li M , Hong W , Shao J , Xu H , Shimano H , Lu J , Xu Y (2018b) Brg1 regulates pro‐lipogenic transcription by modulating SREBP activity in hepatocytes. Biochim Biophys Acta 1864: 3487–3495 10.1016/j.bbadis.2018.05.022 29857051

[emmm202216592-bib-0043] Li N , Kong M , Zeng S , Hao C , Li M , Li L , Xu Z , Zhu M , Xu Y (2019a) Brahma related gene 1 (Brg1) contributes to liver regeneration by epigenetically activating the Wnt/beta‐catenin pathway in mice. FASEB J 33: 327–338 30001167 10.1096/fj.201800197R

[emmm202216592-bib-0044] Li Z , Chen B , Dong W , Kong M , Shao Y , Fan Z , Yu L , Wu D , Lu J , Guo J *et al* (2019b) The chromatin remodeler Brg1 integrates ROS production and endothelial‐mesenchymal transition to promote liver fibrosis in mice. Front Dev Cell Biol 7: 245 10.3389/fcell.2019.00245 PMC6842935 31750301

[emmm202216592-bib-0045] Li Z , Zhao Q , Lu Y , Zhang Y , Li L , Li M , Chen X , Sun D , Duan Y , Xu Y (2021) DDIT4 S‐Nitrosylation aids p38‐MAPK signaling complex assembly to promote hepatic reactive oxygen species production. Adv Sci 8: 2101957 10.1002/advs.202101957 PMC8456271 34310076

[emmm202216592-bib-0046] Liu M , Sun T , Li N , Peng J , Fu D , Li W , Li L , Gao WQ (2019) BRG1 attenuates colonic inflammation and tumorigenesis through autophagy‐dependent oxidative stress sequestration. Nat Commun 10: 4614 31601814 10.1038/s41467-019-12573-z PMC6787222

[emmm202216592-bib-0047] Lucey MR , Mathurin P , Morgan TR (2009) Alcoholic hepatitis. N Engl J Med 360: 2758–2769 19553649 10.1056/NEJMra0805786

[emmm202216592-bib-0048] Mason BJ , Heyser CJ (2021) Alcohol use disorder: the role of medication in recovery. Alcohol Res 41: 7 10.35946/arcr.v41.1.07 PMC8184096 34113531

[emmm202216592-bib-0049] McMullen MR , Pritchard MT , Wang Q , Millward CA , Croniger CM , Nagy LE (2005) Early growth response‐1 transcription factor is essential for ethanol‐induced fatty liver injury in mice. Gastroenterology 128: 2066–2076 15940638 10.1053/j.gastro.2005.02.065 PMC1959407

[emmm202216592-bib-0050] Mookerjee RP , Stadlbauer V , Lidder S , Wright GA , Hodges SJ , Davies NA , Jalan R (2007) Neutrophil dysfunction in alcoholic hepatitis superimposed on cirrhosis is reversible and predicts the outcome. Hepatology 46: 831–840 17680644 10.1002/hep.21737

[emmm202216592-bib-0051] Nara N , Nakayama Y , Okamoto S , Tamura H , Kiyono M , Muraoka M , Tanaka K , Taya C , Shitara H , Ishii R *et al* (2007) Disruption of CXC motif chemokine ligand‐14 in mice ameliorates obesity‐induced insulin resistance. J Biol Chem 282: 30794–30803 17724031 10.1074/jbc.M700412200

[emmm202216592-bib-0052] Ng LG , Ostuni R , Hidalgo A (2019) Heterogeneity of neutrophils. Nat Rev Immunol 19: 255–265 30816340 10.1038/s41577-019-0141-8

[emmm202216592-bib-0053] Niu L , Zheng Z , Xue Q , Cheng H , Liu Y , Wang H , Hu X , Zhang A , Liu B , Xu X (2020) Two coupled mutations abolished the binding of CEBPB to the promoter of CXCL14 that displayed an antiviral effect on PRRSV by activating IFN signaling. FASEB J 34: 11257–11271 32648265 10.1096/fj.202000477R

[emmm202216592-bib-0054] de Oliveira S , Rosowski EE , Huttenlocher A (2016) Neutrophil migration in infection and wound repair: going forward in reverse. Nat Rev Immunol 16: 378–391 27231052 10.1038/nri.2016.49 PMC5367630

[emmm202216592-bib-0055] Peacock A , Leung J , Larney S , Colledge S , Hickman M , Rehm J , Giovino GA , West R , Hall W , Griffiths P *et al* (2018) Global statistics on alcohol, tobacco and illicit drug use: 2017 status report. Addiction 113: 1905–1926 29749059 10.1111/add.14234

[emmm202216592-bib-0056] Poikolainen K (2000) Risk factors for alcohol dependence: a case‐control study. Alcohol Alcohol 35: 190–196 10787396 10.1093/alcalc/35.2.190

[emmm202216592-bib-0057] Qi X , Qiu J , Chang J , Ji Y , Yang Q , Cui G , Sun L , Chai Q , Qin J (2021) Brg1 restrains the pro‐inflammatory properties of ILC3s and modulates intestinal immunity. Mucosal Immunol 14: 38–52 32612160 10.1038/s41385-020-0317-3 PMC7790751

[emmm202216592-bib-0058] Ramaiah SK , Jaeschke H (2007) Role of neutrophils in the pathogenesis of acute inflammatory liver injury. Toxicol Pathol 35: 757–766 17943649 10.1080/01926230701584163

[emmm202216592-bib-0059] Robbins RA , Zetterman RK , Kendall TJ , Gossman GL , Monsour HP , Rennard SI (1987) Elevation of chemotactic factor inactivator in alcoholic liver disease. Hepatology 7: 872–877 3653852 10.1002/hep.1840070513

[emmm202216592-bib-0060] Robinson MW , Harmon C , O'Farrelly C (2016) Liver immunology and its role in inflammation and homeostasis. Cell Mol Immunol 13: 267–276 27063467 10.1038/cmi.2016.3 PMC4856809

[emmm202216592-bib-0061] Salogni L , Musso T , Bosisio D , Mirolo M , Jala VR , Haribabu B , Locati M , Sozzani S (2009) Activin a induces dendritic cell migration through the polarized release of CXC chemokine ligands 12 and 14. Blood 113: 5848–5856 1933969410.1182/blood-2008-12-194597

[emmm202216592-bib-0062] Seitz HK , Bataller R , Cortez‐Pinto H , Gao B , Gual A , Lackner C , Mathurin P , Mueller S , Szabo G , Tsukamoto H (2018) Alcoholic liver disease. Nat Rev Dis Primers 4: 16 30115921 10.1038/s41572-018-0014-7

[emmm202216592-bib-0063] Senthil Kumar KJ , Liao JW , Xiao JH , Gokila Vani M , Wang SY (2012) Hepatoprotective effect of lucidone against alcohol‐induced oxidative stress in human hepatic HepG2 cells through the up‐regulation of HO‐1/Nrf‐2 antioxidant genes. Toxicol In Vitro 26: 700–708 22484158 10.1016/j.tiv.2012.03.012

[emmm202216592-bib-0064] Shao J , Xu Y , Fang M (2020) BRG1 deficiency in endothelial cells alleviates thioacetamide induced liver fibrosis in mice. Biochem Biophys Res Commun 521: 212–219 31635808 10.1016/j.bbrc.2019.10.109

[emmm202216592-bib-0065] Sharma T , Robinson DCL , Witwicka H , Dilworth FJ , Imbalzano AN (2021) The Bromodomains of the mammalian SWI/SNF (mSWI/SNF) ATPases Brahma (BRM) and Brahma related gene 1 (BRG1) promote chromatin interaction and are critical for skeletal muscle differentiation. Nucleic Acids Res 49: 8060–8077 34289068 10.1093/nar/gkab617 PMC8373147

[emmm202216592-bib-0066] Szabo G , Catalano D , Bellerose G , Mandrekar P (2001) Interferon alpha and alcohol augment nuclear regulatory factor‐kappaB activation in HepG2 cells, and interferon alpha increases pro‐inflammatory cytokine production. Alcohol Clin Exp Res 25: 1188–1197 11505050

[emmm202216592-bib-0067] Tian W , Xu H , Fang F , Chen Q , Xu Y , Shen A (2013) Brahma‐related gene 1 bridges epigenetic regulation of proinflammatory cytokine production to steatohepatitis in mice. Hepatology 58: 576–588 23281043 10.1002/hep.26207

[emmm202216592-bib-0068] Vangamudi B , Paul TA , Shah PK , Kost‐Alimova M , Nottebaum L , Shi X , Zhan Y , Leo E , Mahadeshwar HS , Protopopov A *et al* (2015) The SMARCA2/4 ATPase domain surpasses the Bromodomain as a drug target in SWI/SNF‐mutant cancers: insights from cDNA rescue and PFI‐3 inhibitor studies. Cancer Res 75: 3865–3878 26139243 10.1158/0008-5472.CAN-14-3798 PMC4755107

[emmm202216592-bib-0069] Vidali M , Stewart SF , Albano E (2008) Interplay between oxidative stress and immunity in the progression of alcohol‐mediated liver injury. Trends Mol Med 14: 63–71 18222109 10.1016/j.molmed.2007.12.005

[emmm202216592-bib-0070] Wang B , Kaufmann B , Engleitner T , Lu M , Mogler C , Olsavszky V , Ollinger R , Zhong S , Geraud C , Cheng Z *et al* (2019) Brg1 promotes liver regeneration after partial hepatectomy via regulation of cell cycle. Sci Rep 9: 2320 30787318 10.1038/s41598-019-38568-w PMC6382836

[emmm202216592-bib-0071] Wu Q , Sharma S , Cui H , LeBlanc SE , Zhang H , Muthuswami R , Nickerson JA , Imbalzano AN (2016) Targeting the chromatin remodeling enzyme BRG1 increases the efficacy of chemotherapy drugs in breast cancer cells. Oncotarget 7: 27158–27175 27029062 10.18632/oncotarget.8384 PMC5053639

[emmm202216592-bib-0072] Wu X , Dong W , Kong M , Ren H , Wang J , Shang L , Zhu Z , Zhu W , Shi X (2021) Down‐regulation of CXXC5 de‐represses MYCL1 to promote hepatic stellate cell activation. Front Cell Dev Biol 9: 680344 34621736 10.3389/fcell.2021.680344 PMC8490686

[emmm202216592-bib-0073] Xu R , Huang H , Zhang Z , Wang FS (2014) The role of neutrophils in the development of liver diseases. Cell Mol Immunol 11: 224–231 24633014 10.1038/cmi.2014.2 PMC4085492

[emmm202216592-bib-0074] Yin HQ , Kim M , Kim JH , Kong G , Kang KS , Kim HL , Yoon BI , Lee MO , Lee BH (2007) Differential gene expression and lipid metabolism in fatty liver induced by acute ethanol treatment in mice. Toxicol Appl Pharmacol 223: 225–233 17655900 10.1016/j.taap.2007.06.018

[emmm202216592-bib-0075] Zetterman RK , Sorrell MF (1981) Immunologic aspects of alcoholic liver disease. Gastroenterology 81: 616–624 6166511

[emmm202216592-bib-0076] Zhang X , Liu S , Weng X , Zeng S , Yu L , Guo J , Xu Y (2018) Brg1 deficiency in vascular endothelial cells blocks neutrophil recruitment and ameliorates cardiac ischemia‐reperfusion injury in mice. Int J Cardiol 269: 250–258 30049497 10.1016/j.ijcard.2018.07.105

[emmm202216592-bib-0077] Zhang Y , Yuan Y , Li Z , Chen H , Fang M , Xiao P , Xu Y (2019) An interaction between BRG1 and histone modifying enzymes mediates lipopolysaccharide‐induced proinflammatory cytokines in vascular endothelial cells. J Cell Biochem 120: 13216–13225 30891798 10.1002/jcb.28595

[emmm202216592-bib-0078] Zhao J , Zhang S , Liu Y , He X , Qu M , Xu G , Wang H , Huang M , Pan J , Liu Z *et al* (2020) Single‐cell RNA sequencing reveals the heterogeneity of liver‐resident immune cells in human. Cell Discov 6: 22 32351704 10.1038/s41421-020-0157-z PMC7186229

[emmm202216592-bib-0079] Zhou Y , Chen Y , Zhang X , Xu Q , Wu Z , Cao X , Shao M , Shu Y , Lv T , Lu C *et al* (2021) Brahma‐related gene 1 inhibition prevents liver fibrosis and cholangiocarcinoma by attenuating progenitor expansion. Hepatology 74: 797–815 33650193 10.1002/hep.31780

