# Supplementary material for: Targetable Brg1‐CXCL14 axis contributes to alcoholic liver injury by driving neutrophil trafficking
Source: EMBO Mol Med. 2023 Feb 1;15(3):e16592. doi: 10.15252/emmm.202216592 (PMC9994483; doi:10.15252/emmm.202216592)
Supplement: Supplementary file 1 — Appendix S1 [file EMMM-15-e16592-s006.pdf]

Li N et al: Targetable Brg1-CXCL14 axis contributes to alcoholic liver injury by driving neutrophil trafficking

Appendix

Supplementary figures: 20

Supplementary table: 1

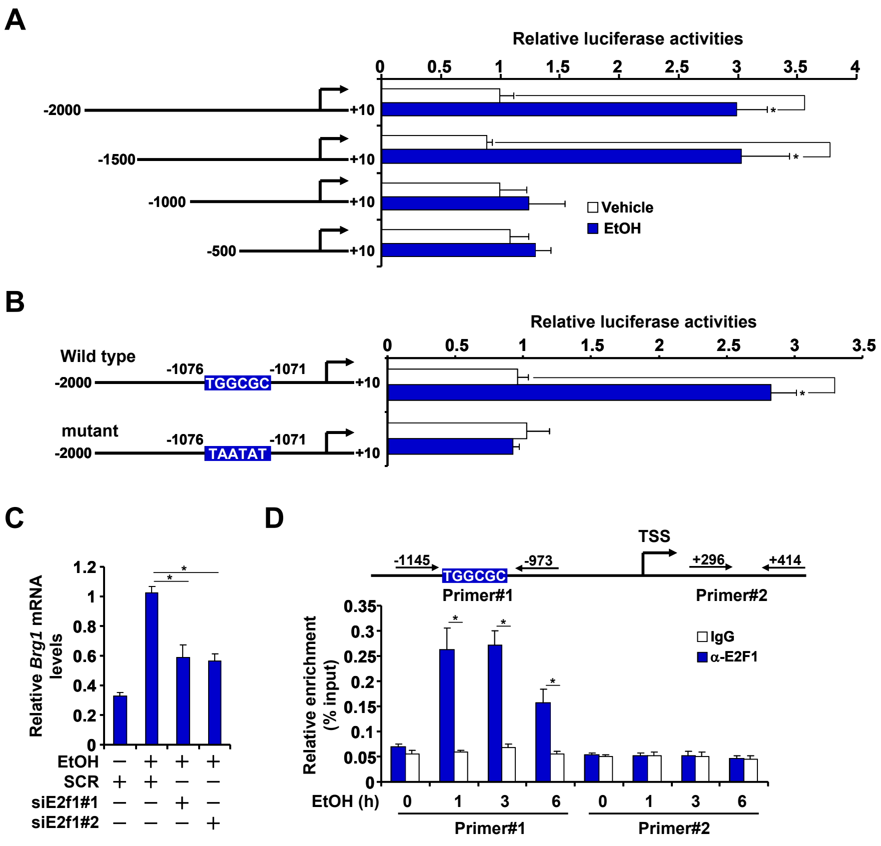

Appendix Figure S1: (**A**) *Smarca4* promoter constructs of different lengths were transfected into HepG2 cells followed by treatment with ethanol for 1h. Luciferase activities were normalized by protein concentration and GFP fluorescence. (**B**) Wild type and E2F1 mutant *Smarca4* promoter constructs were transfected into HepG2 cells followed by treatment with ethanol for 1h. Luciferase activities were normalized by protein concentration and GFP fluorescence. (**C**) Primary hepatocytes were transfected with indicated siRNAs followed by treatment with ethanol for 1h. Brg1 expression levels were examined by qPCR. (**D**) Primary Mouse primary hepatocytes were exposed to ethanol (50mM) and harvested at indicated time points. ChIP assays were performed with anti-E2F1 or IgG.

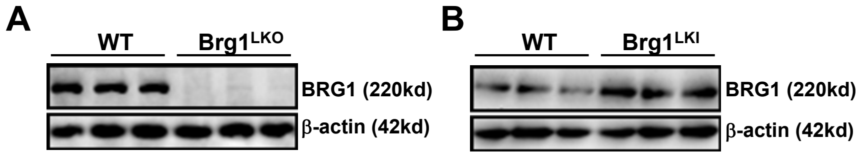

Appendix Figure S2: (**A**) Primary hepatocytes were isolated from WT and Brg1^{LKO} mice and BRG1 expression was examined by Western blotting. (**B**) Primary hepatocytes were isolated from WT and Brg1^{LKI} mice and BRG1 expression was examined by Western blotting.

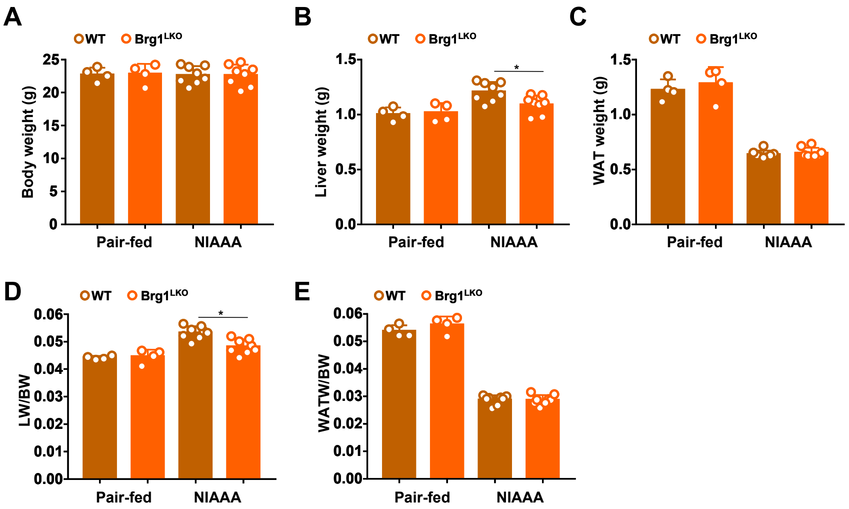

Appendix Figure S3: Alcoholic liver injury was induced in WT and Brg1 LKO mice by NIAAA feeding as described in Methods. (**A**) Body weight. (**B**) Liver weight. (**C**) Gonadal white adipose tissue (WAT) weight. (**D**) Liver weight/body weight ratio. (**E**) WAT weight/body weight ratio.

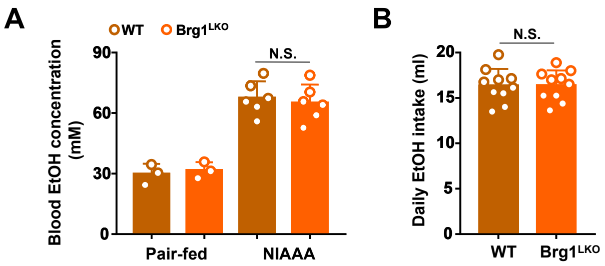

Appendix Figure S4: Alcoholic liver injury was induced in WT and Brg1 LKO mice by chronic ethanol feeding as described in Methods. (**A**) Blood alcohol levels. (**B**) Daily alcohol consumption.

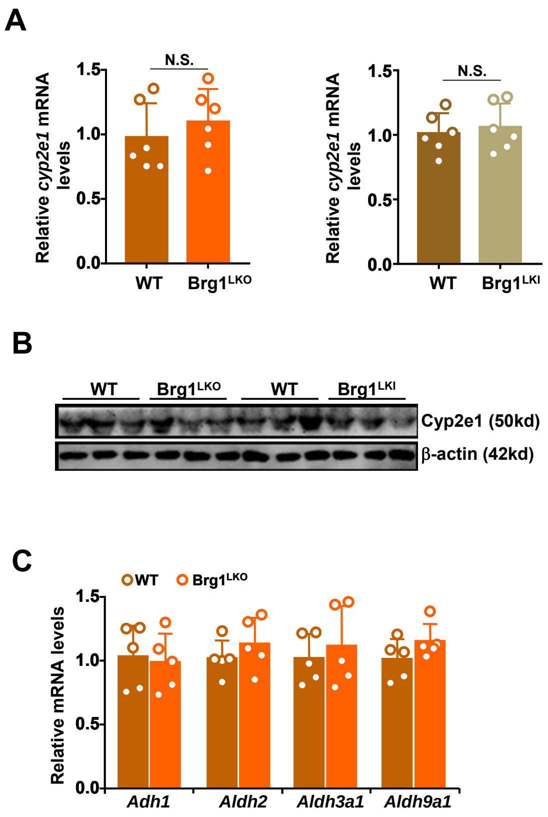

Appendix Figure S5: (**A, B**) Alcoholic liver injury was induced in WT, Brg1 LKO mice, or Brg1 LKI mice by chronic ethanol feeding as described in Methods. Cyp2e1 levels were examined by qPCR and Western blotting. (**C**) Alcoholic liver injury was induced in WT and Brg1 LKO mice by chronic ethanol feeding as described in Methods. Gene expression levels were examined by qPCR.

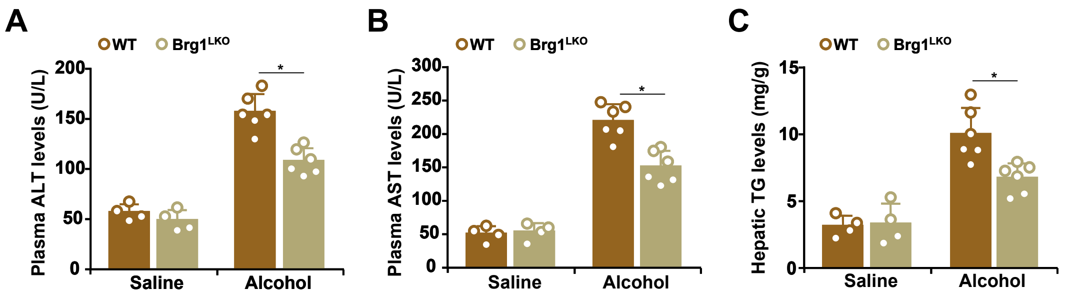

Appendix Figure S6: Alcoholic liver injury was induced in WT and Brg1 LKO mice by two consecutive oral gavages as described in Methods. (A) Plasma ALT levels. (B) Plasma AST levels. (C) Hepatic triglyceride levels.

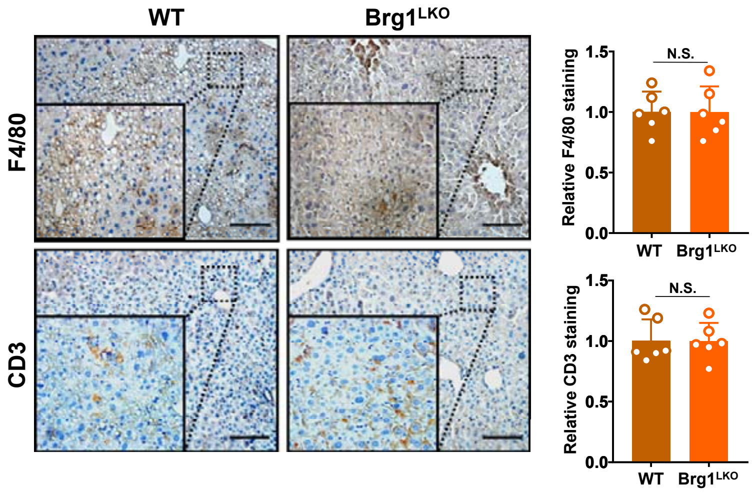

Appendix Figure S7: Alcoholic liver injury was induced in WT and Brg1 LKO mice by NIAAA feeding as described in Methods. Immunohistochemical staining was performed with anti-F4/80 or anti-CD3.

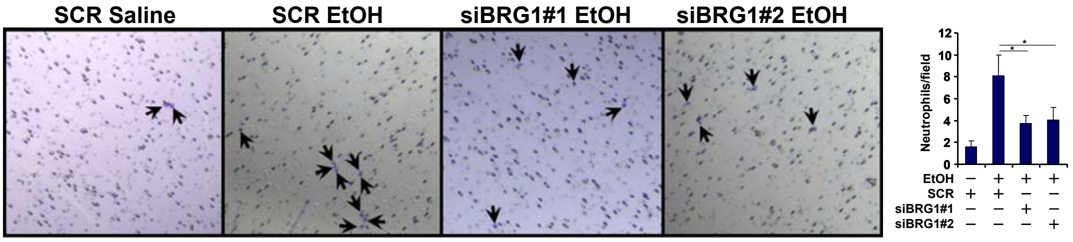

Appendix Figure S8: HepaRG cells were transfected with indicated siRNAs followed by treatment with followed by treatment with ethanol. Transwell assay was performed as described in Methods.

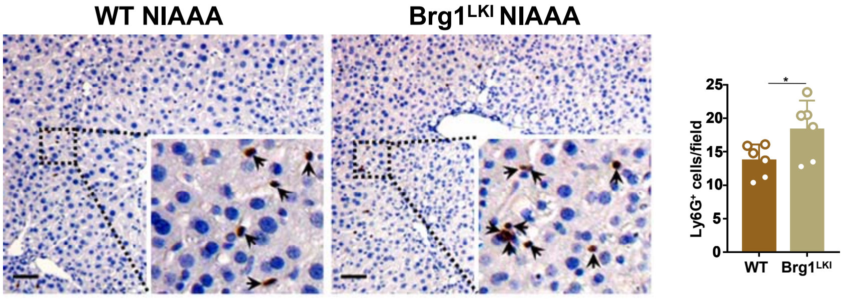

Appendix Figure S9: Alcoholic liver injury was induced in WT and Brg1 LKI mice by NIAAA feeding as described in Methods. Immunohistochemical staining was performed with anti-Ly6G.

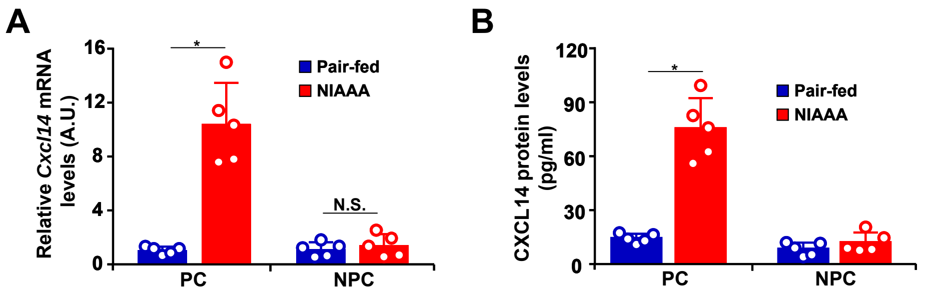

Appendix Figure S10: (**A, B**) Alcoholic liver injury was induced in C57/BL6 mice by NIAAA feeding as described in Methods. Parenchymal cells (PC) and non-parenchymal cells (NPC) were isolated and CXCL14 expression levels were examined by qPCR (A) and ELISA (B).

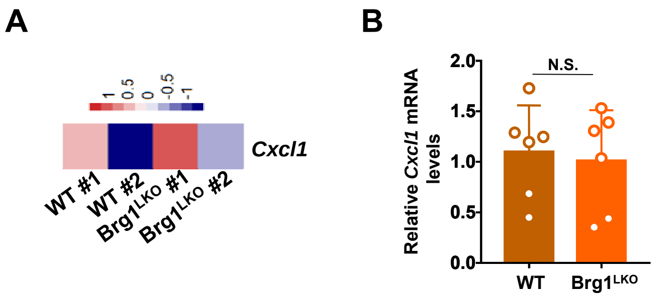

Appendix Figure S11: (**A**) Alcoholic liver injury was induced in WT and Brg1 LKO mice by NIAAA feeding as described in Methods. RNA-seq was performed using liver homogenates. Heatmap shows relative expression of CXCL1. (**B**) Alcoholic liver injury was induced in WT and Brg1 LKI mice by NIAAA feeding as described in Methods. CXCL1 levels were examined by qPCR.

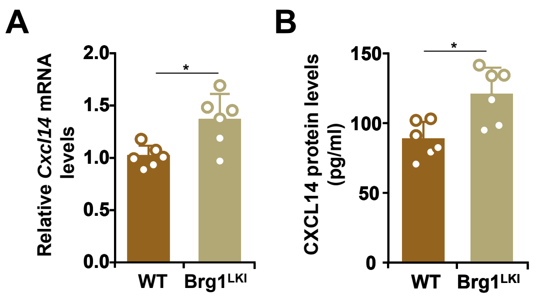

Appendix Figure S12: Alcoholic liver injury was induced in WT and Brg1 LKI mice by chronic ethanol feeding as described in Methods. CXCL14 levels were examined by qPCR and ELISA.

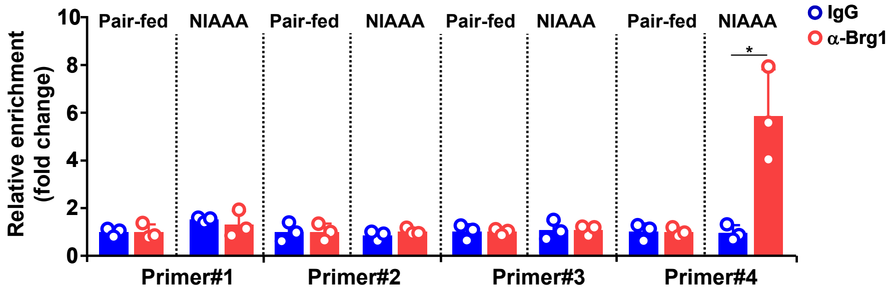

Appendix Figure S13: C57/B6 mice were subjected to the NIAAA procedure or pair-fed for 2 weeks as described in Methods. ChIP assay were performed with anti-Brg1 or IgG using liver homogenates.

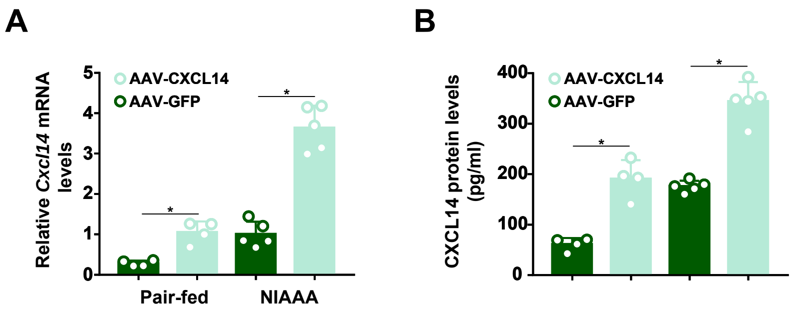

Appendix Figure S14: C57B/6 were injected via tailed AAV8-CXCL14 or AAV8-GFP followed by induction of alcoholic liver injury. CXCL14 levels were examined by qPCR (A) and ELISA (B).

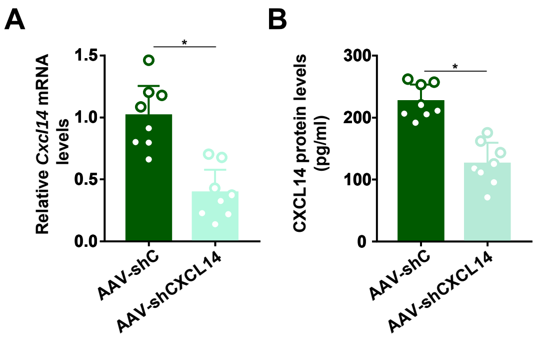

Appendix Figure S15: C57B/6 were injected via tailed AAV8-CXCL14 or AAV8-GFP followed by induction of alcoholic liver injury. CXCL14 levels were examined by qPCR (A) and ELISA (B).

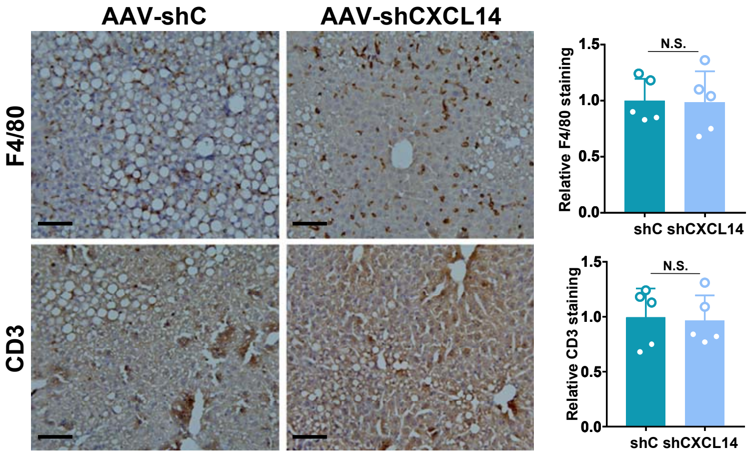

Appendix Figure S16: C57B/6 were injected via tailed AAV8-CXCL14 or AAV8-GFP followed by induction of alcoholic liver injury. Immunohistochemical staining was performed with anti-F4/80 or anti-CD3.

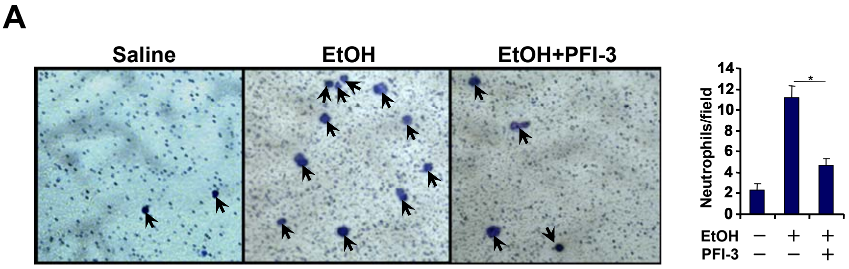

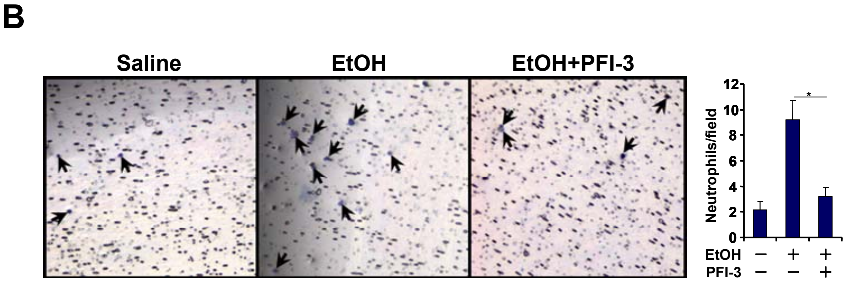

Appendix Figure S17: (**A**) Primary hepatocytes were treated with ethanol in the presence or absence of PFI-3. Transwell assay was performed as described in Methods. (**B**) HepaRG cells were treated with ethanol in the presence or absence of PFI-3. Transwell assay was performed as described in Methods.

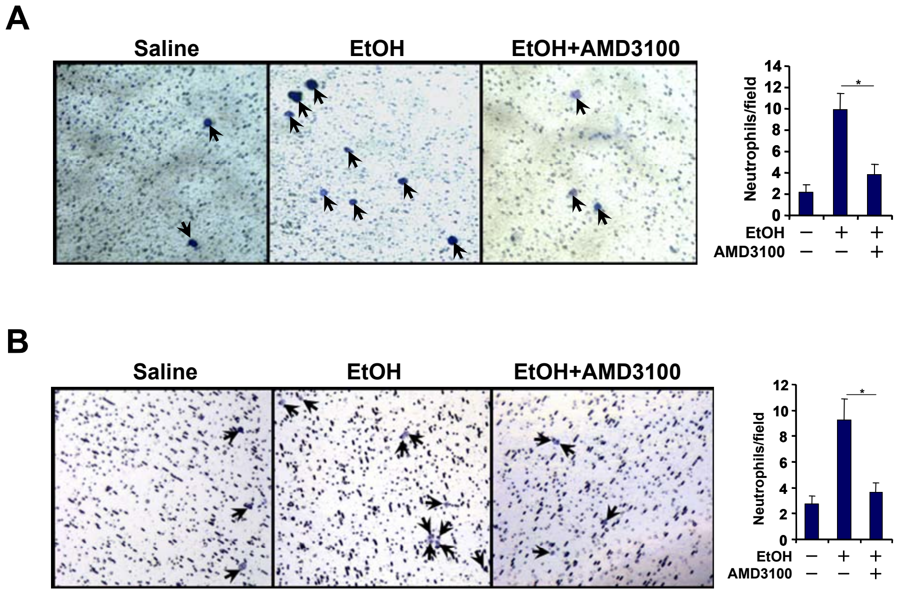

Appendix Figure S18: (**A**) Primary hepatocytes were treated with ethanol in the presence or absence of AMD3100. Transwell assay was performed as described in Methods. (**B**) Primary hepatocytes were treated with ethanol in the presence or absence of AMD3100. Transwell assay was performed as described in Methods.

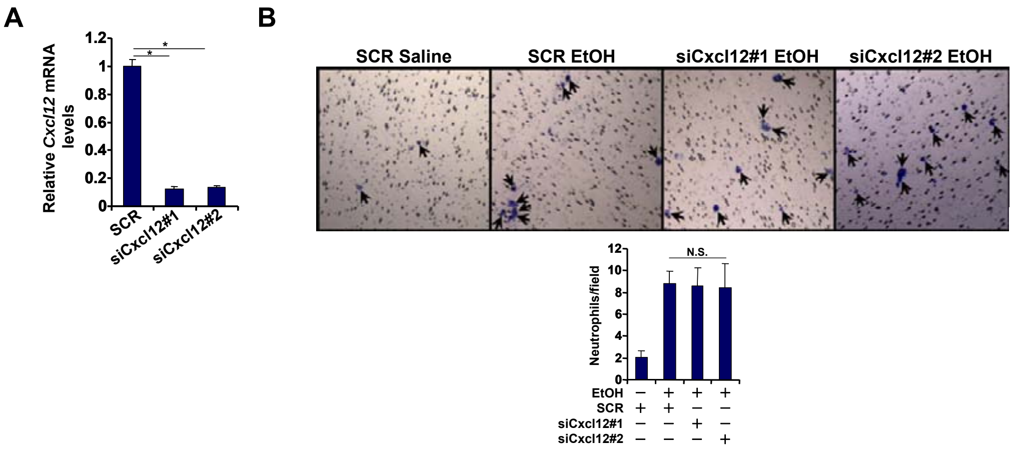

Appendix Figure S19: Primary hepatocytes were transfected with indicated siRNAs followed by treatment with ethanol. (**A**) CXCL12 levels were examined by qPCR. (**B**) Transwell assay was performed as described in Methods.

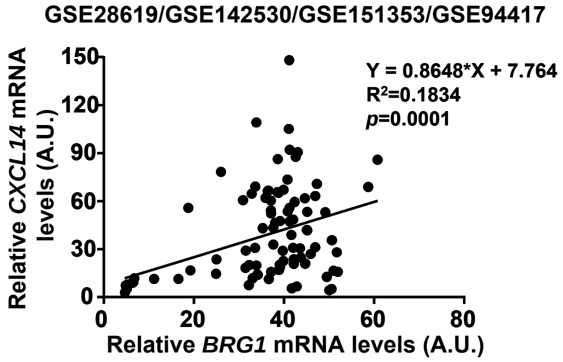

Appendix Figure S20: Correlation between BRG1 expression and CXCL14 expression in the livers of ALD patients was analyzed using four different GSE datasets.

Appendix Table S1: ALD Patient Information Sheet

Patient ID	Gender	Age (yr)	BT (^{o}C)	BP (mmHg)	ALT (U/dL)	AST (U/dL)	LDH (U/dL)	Triglyceride (mM)	Cholesterol (mM)
1	Male	65	36.2	119/72	146.9	386.1	345	1.1	1.92
2	Male	61	37.1	122/68	230.1	466.4	363	1.46	1.69
3	Male	66	36.6	149/78	69.5	195.6	333	1.1	1.47
4	Male	71	36.5	125/75	50.5	223.8	332	1.93	.31
5	Male	65	36.5	133/76	617.8	1726	428	1.08	1.6
6	Male	52	36.5	135/78	64.6	259.2	394	1.52	.28
7	Male	69	36.2	160/96	55.6	163.6	246	1.25	1.49
8	Male	52	36.2	144/80	67.8	193.6	459	1.78	1.83
9	Male	53	36.4	135/89	209.9	424.3	538	4.94	2.11
