# Supplementary figures and images for: Targetable Brg1‐CXCL14 axis contributes to alcoholic liver injury by driving neutrophil trafficking (part 1 of 2)
Source: EMBO Mol Med. 2023 Feb 1;15(3):e16592. doi: 10.15252/emmm.202216592 (PMC9994483; doi:10.15252/emmm.202216592)

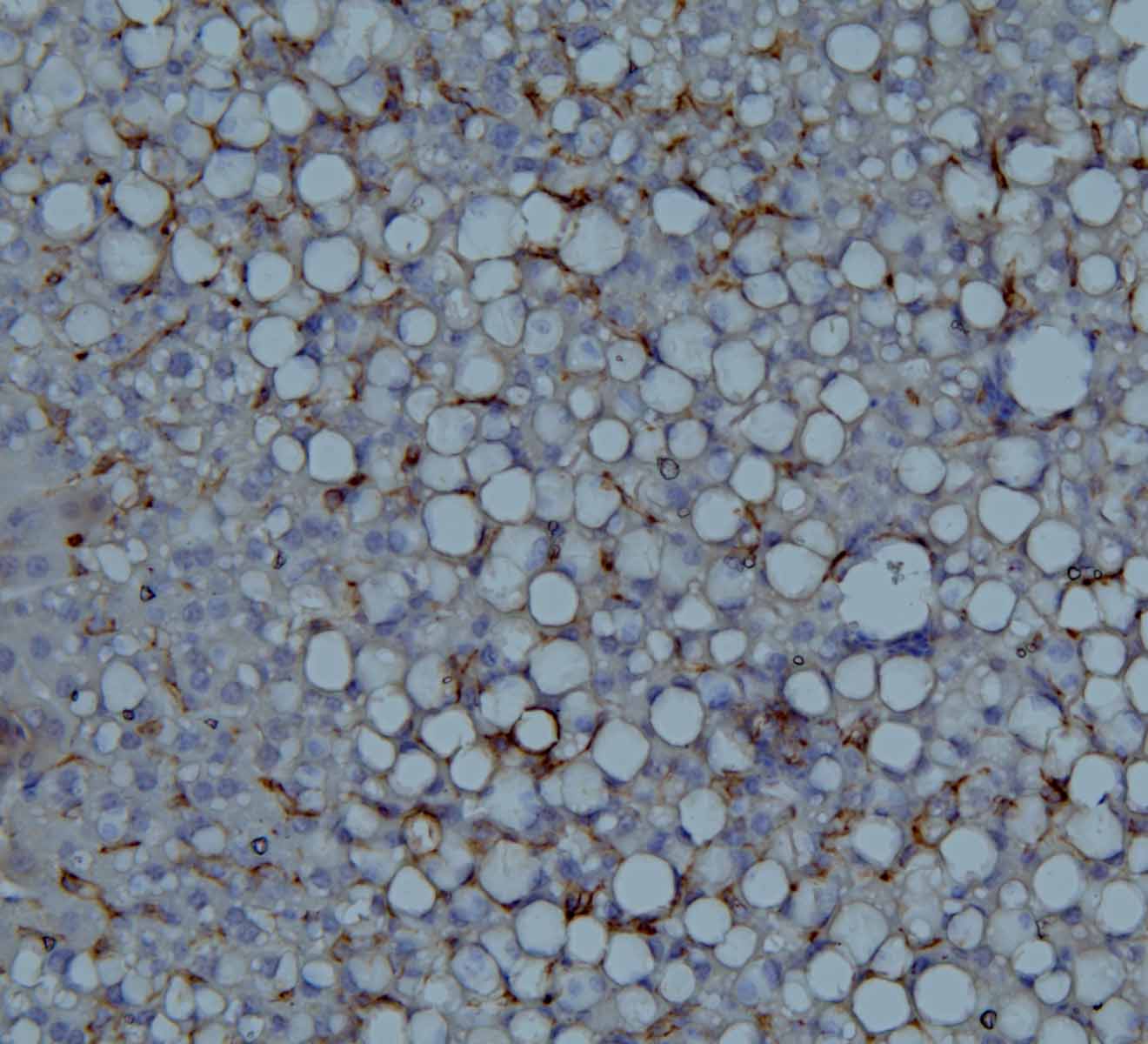

Supplement: Supplementary file 2 — Source Data for Appendix [file EMMM-15-e16592-s004.zip › Appendix/Figure S16/1.jpg]

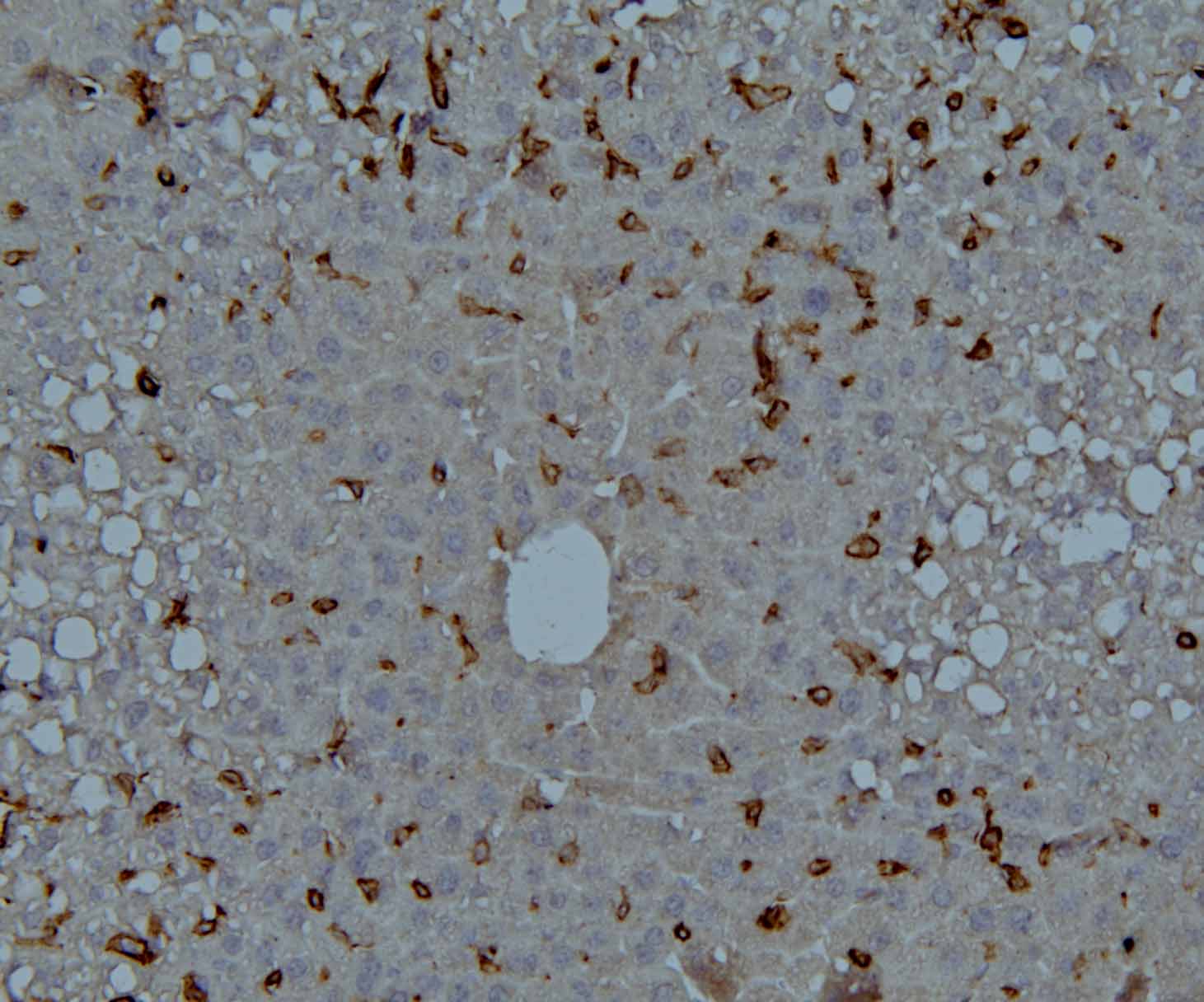

Supplement: Supplementary file 2 — Source Data for Appendix [file EMMM-15-e16592-s004.zip › Appendix/Figure S16/2.jpg]

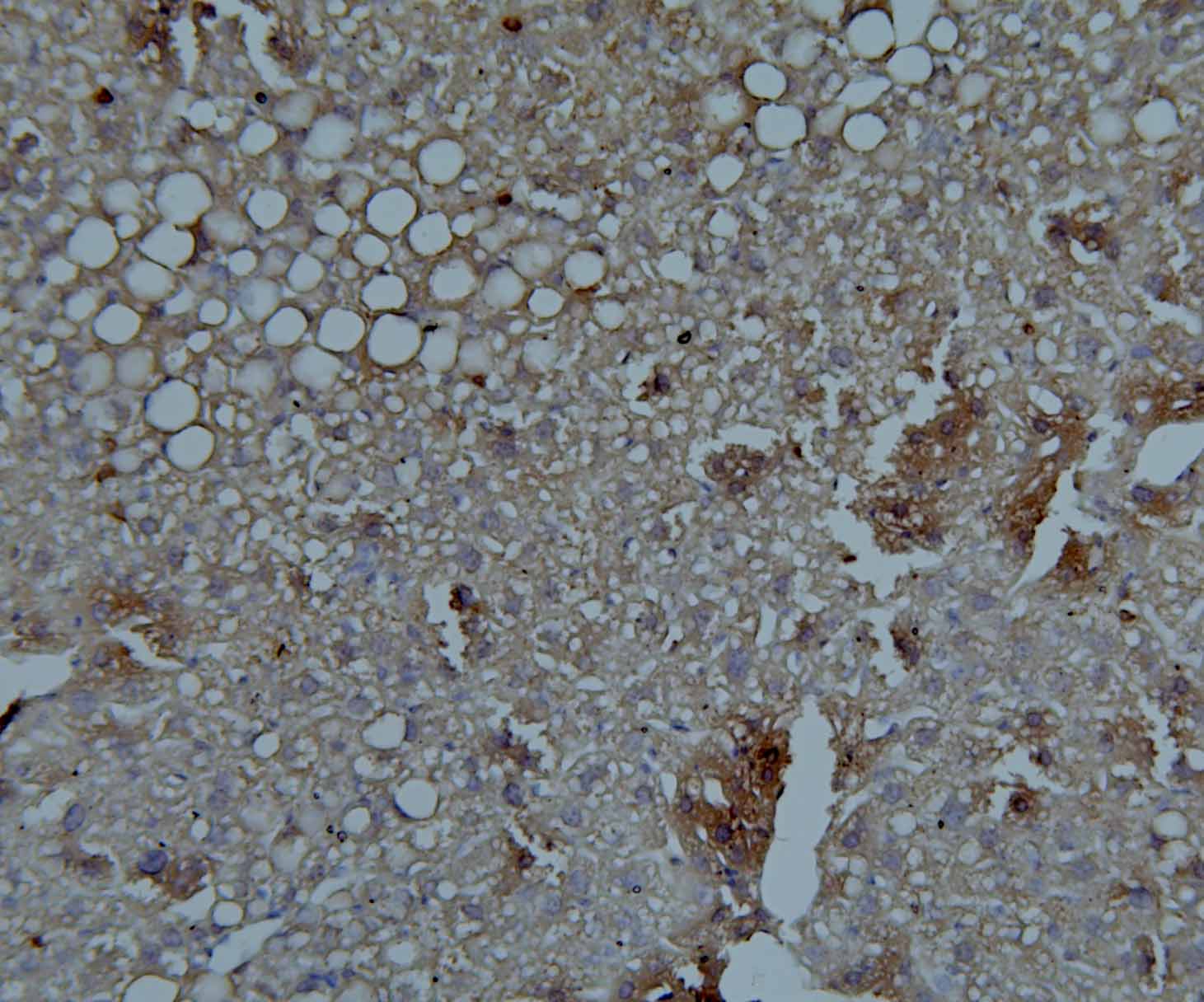

Supplement: Supplementary file 2 — Source Data for Appendix [file EMMM-15-e16592-s004.zip › Appendix/Figure S16/3.jpg]

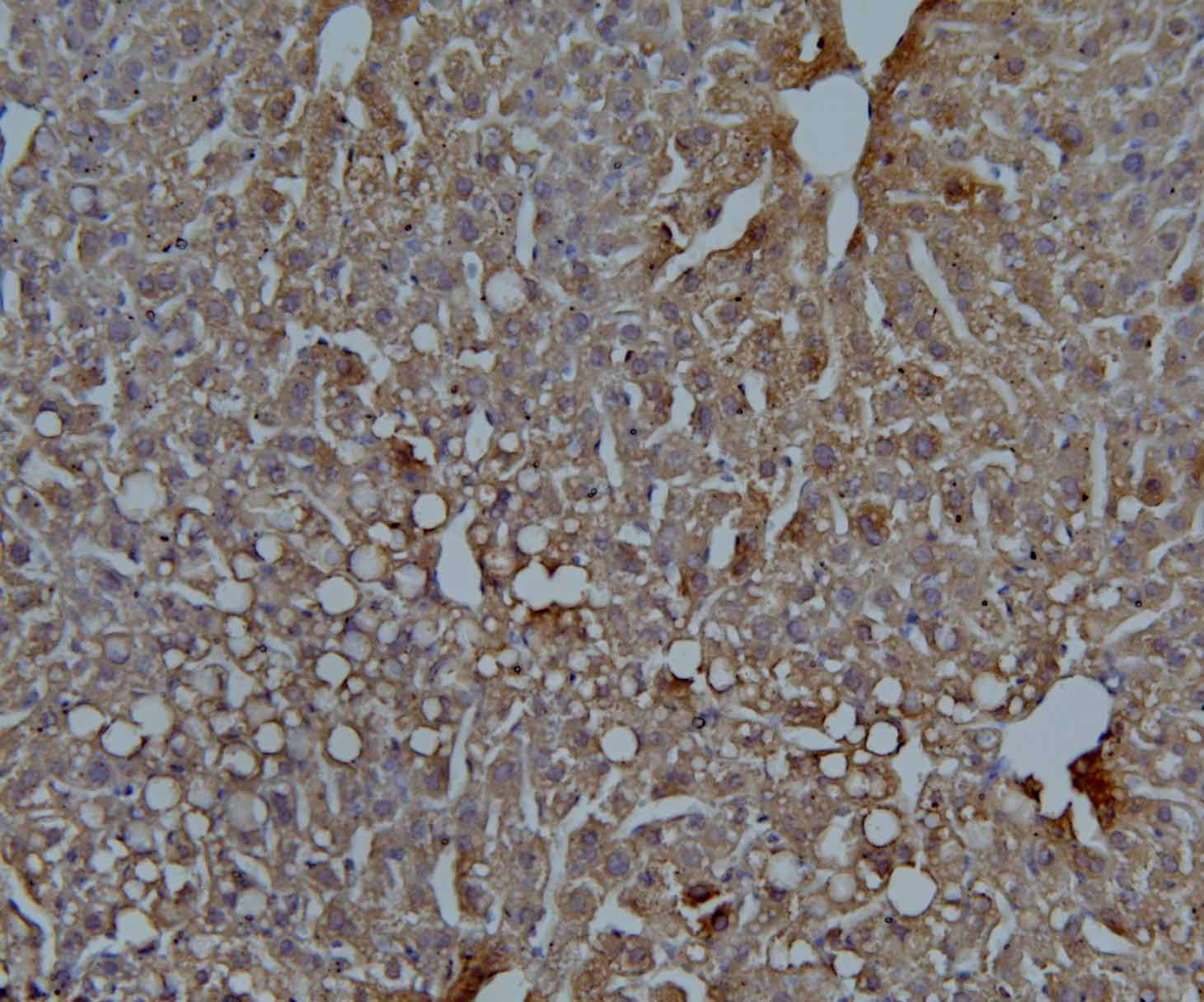

Supplement: Supplementary file 2 — Source Data for Appendix [file EMMM-15-e16592-s004.zip › Appendix/Figure S16/4.jpg]

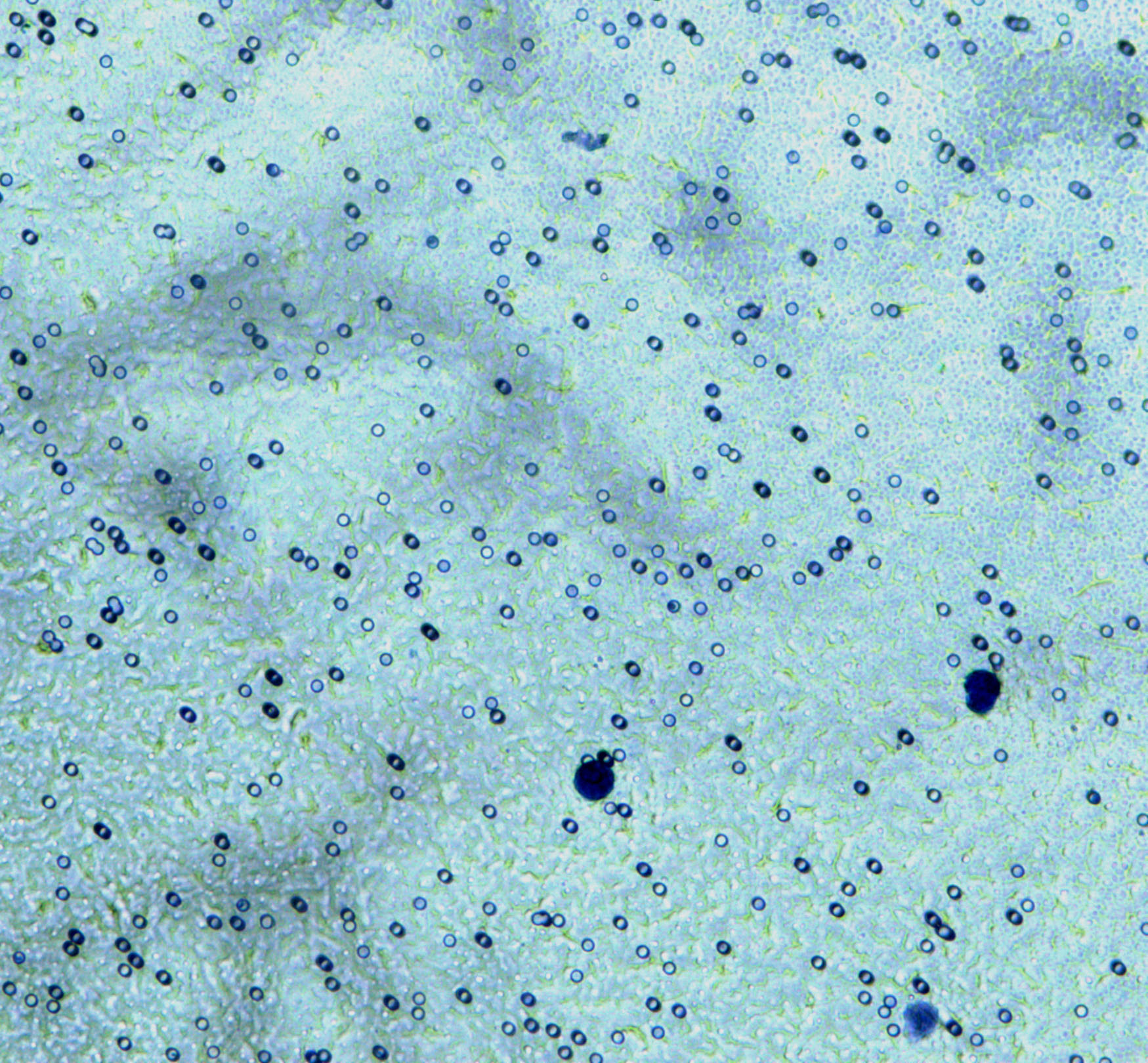

Supplement: Supplementary file 2 — Source Data for Appendix [file EMMM-15-e16592-s004.zip › Appendix/Figure S17/Fig.S17A/Primary murine hepatocyte PFI-3/1.jpg]

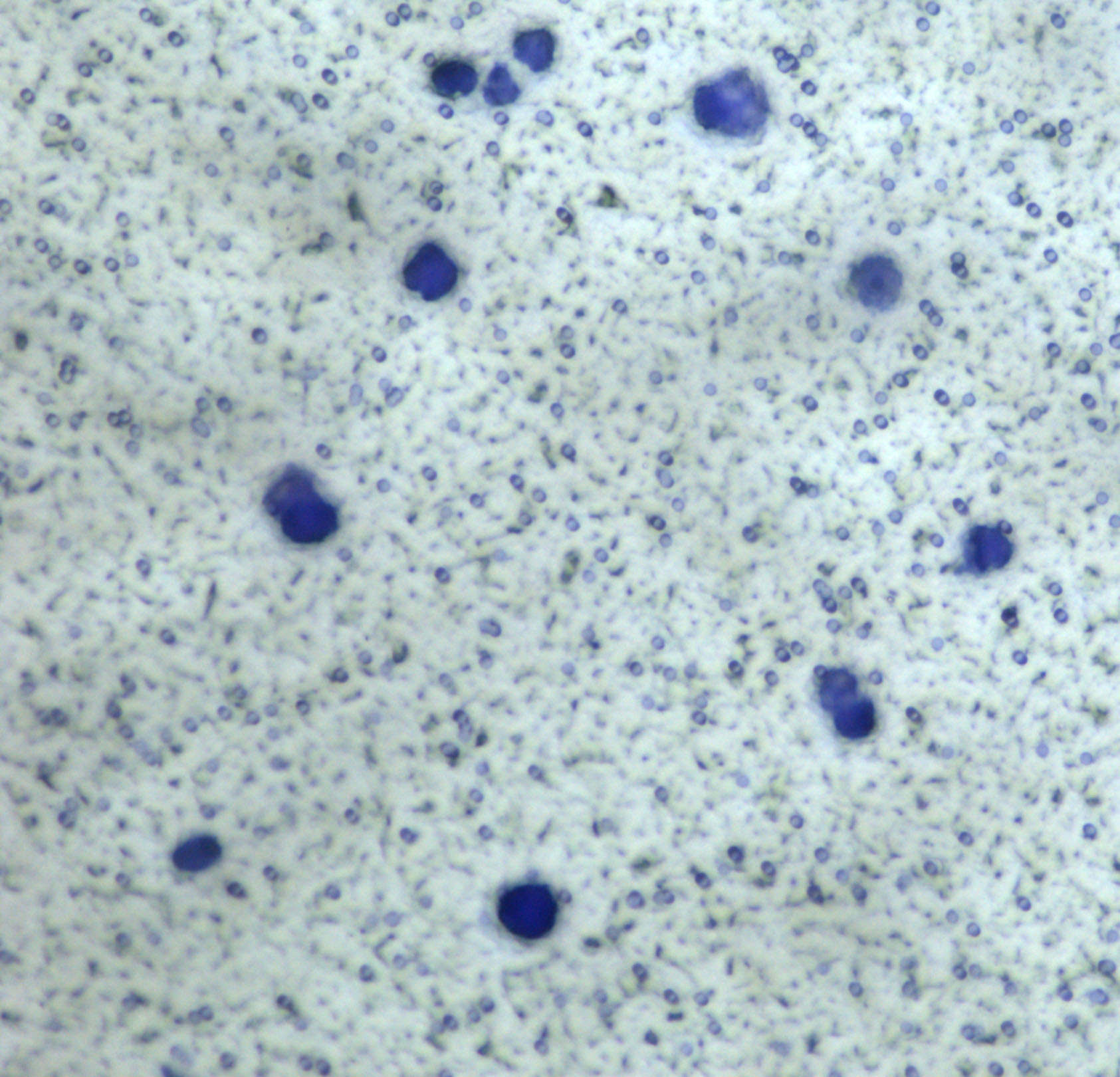

Supplement: Supplementary file 2 — Source Data for Appendix [file EMMM-15-e16592-s004.zip › Appendix/Figure S17/Fig.S17A/Primary murine hepatocyte PFI-3/2.jpg]

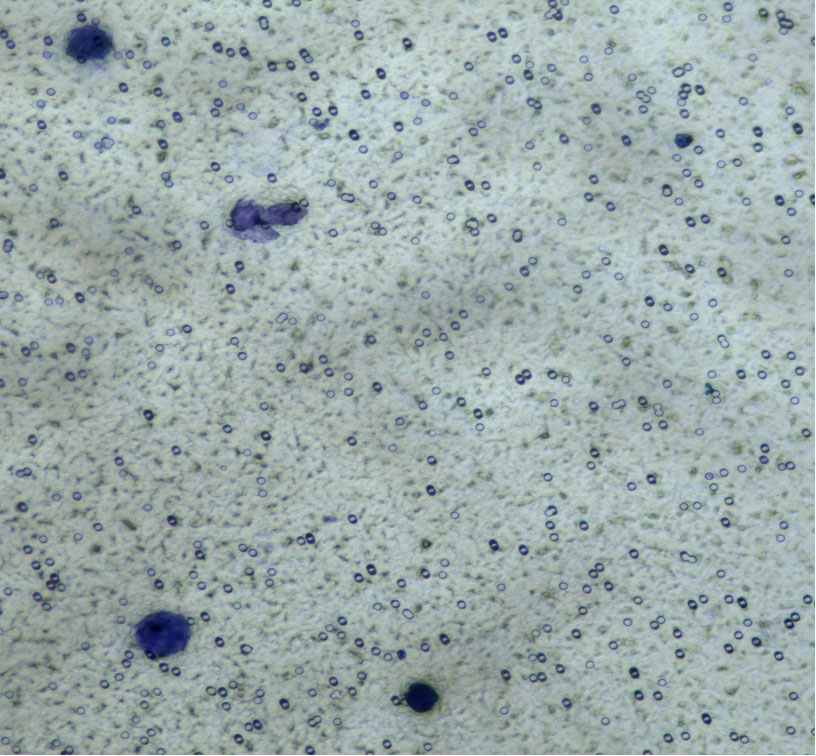

Supplement: Supplementary file 2 — Source Data for Appendix [file EMMM-15-e16592-s004.zip › Appendix/Figure S17/Fig.S17A/Primary murine hepatocyte PFI-3/3.jpg]

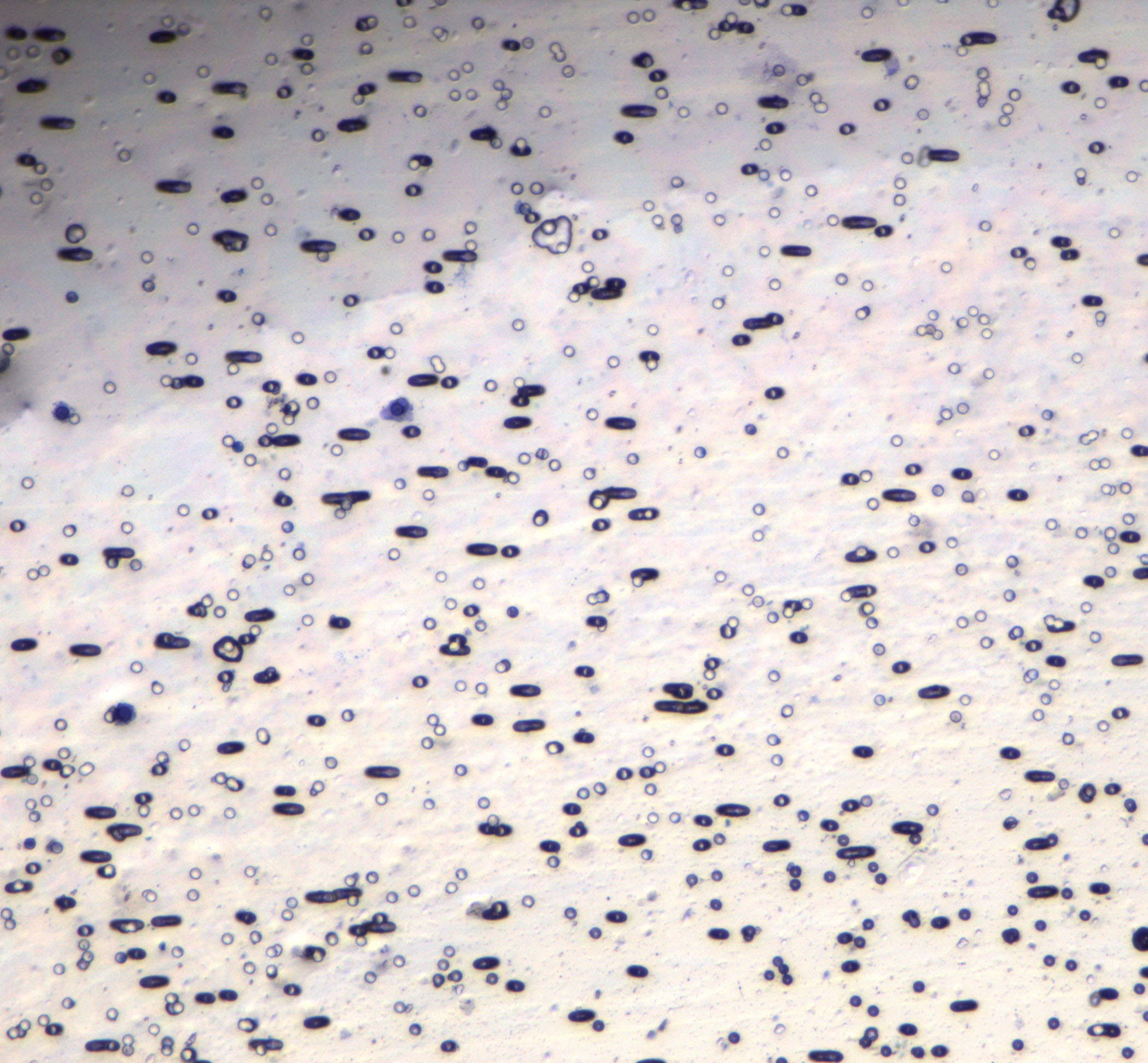

Supplement: Supplementary file 2 — Source Data for Appendix [file EMMM-15-e16592-s004.zip › Appendix/Figure S17/Fig.S17B/HepaRG PFI-3/1.jpg]

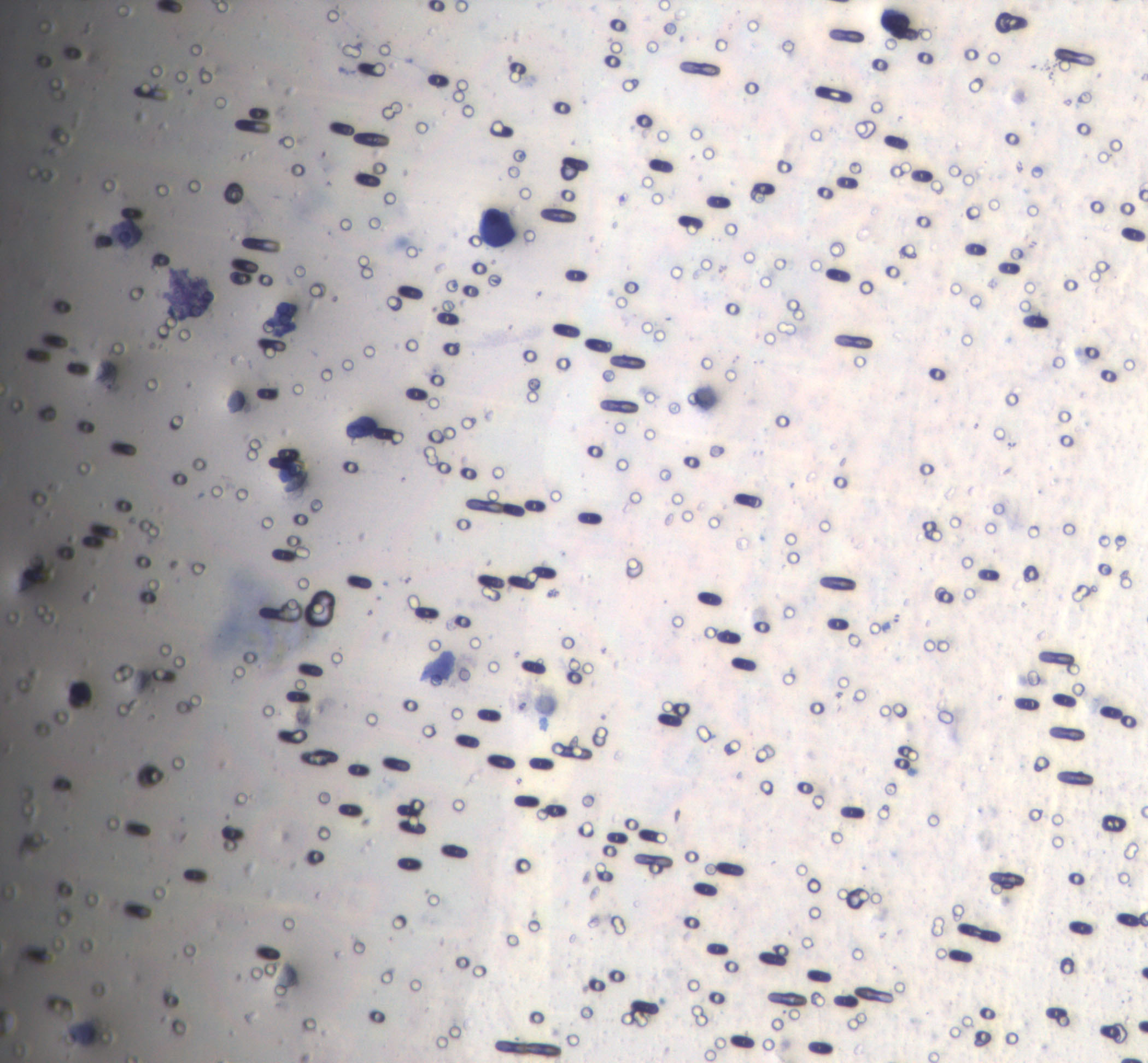

Supplement: Supplementary file 2 — Source Data for Appendix [file EMMM-15-e16592-s004.zip › Appendix/Figure S17/Fig.S17B/HepaRG PFI-3/2.jpg]

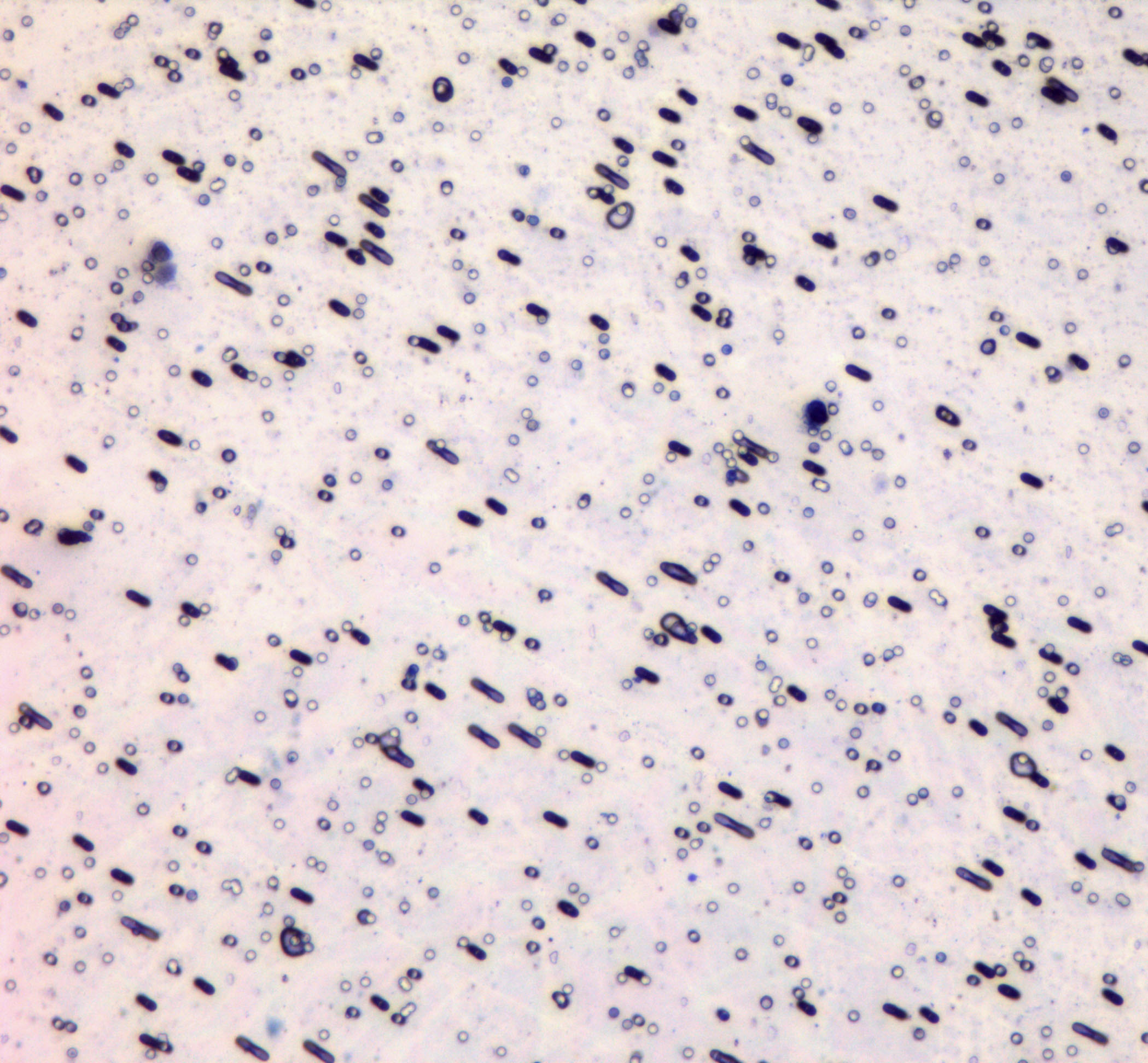

Supplement: Supplementary file 2 — Source Data for Appendix [file EMMM-15-e16592-s004.zip › Appendix/Figure S17/Fig.S17B/HepaRG PFI-3/3.jpg]

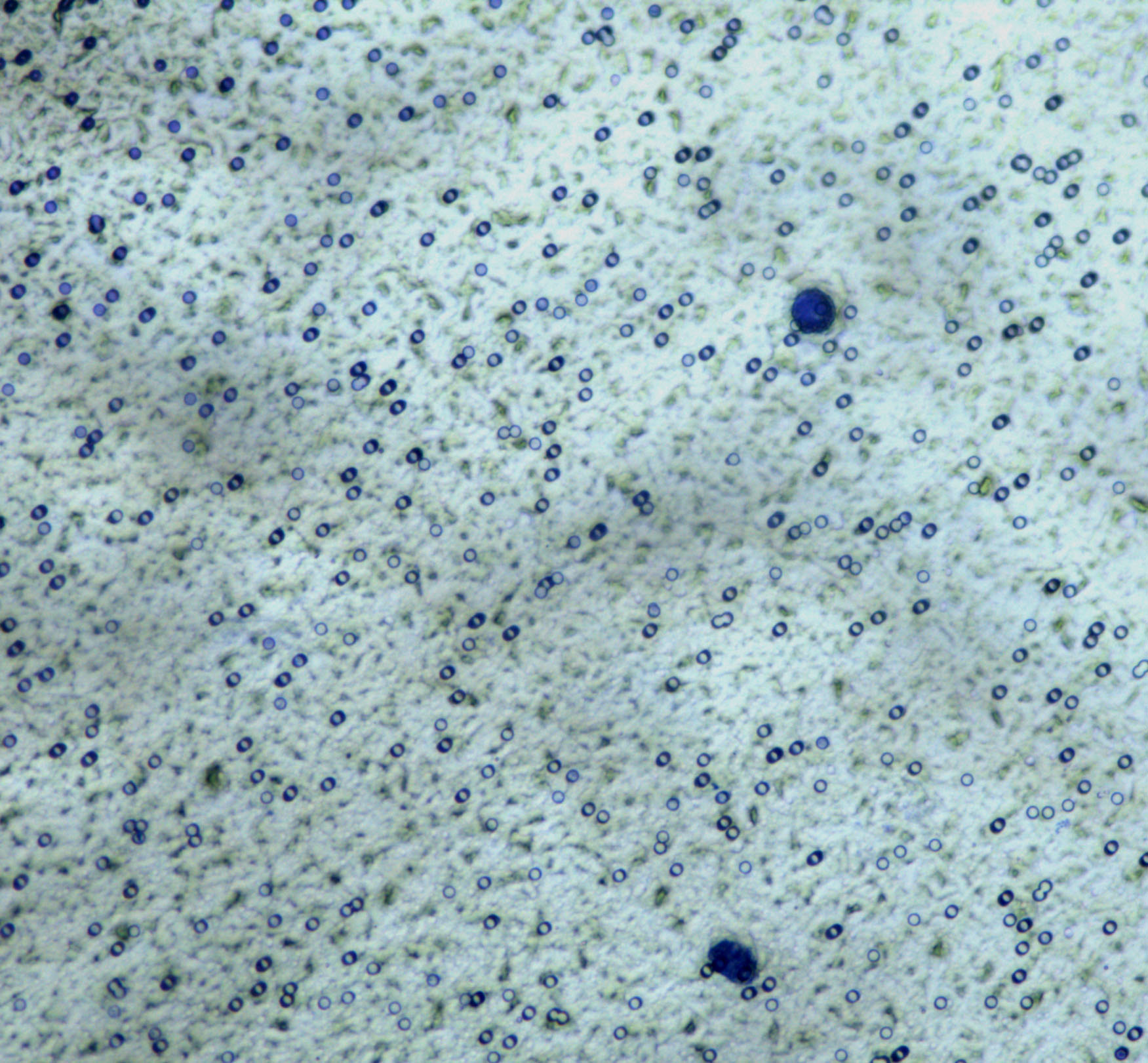

Supplement: Supplementary file 2 — Source Data for Appendix [file EMMM-15-e16592-s004.zip › Appendix/Figure S18/Fig.S18A/Primary murine hepatocyte AMD3100/1.jpg]

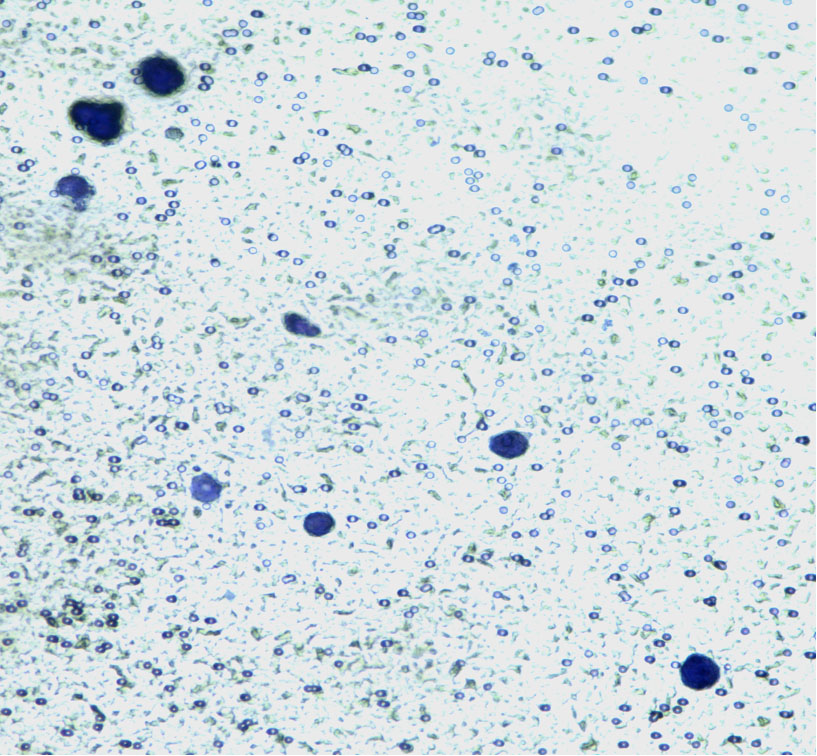

Supplement: Supplementary file 2 — Source Data for Appendix [file EMMM-15-e16592-s004.zip › Appendix/Figure S18/Fig.S18A/Primary murine hepatocyte AMD3100/2.jpg]

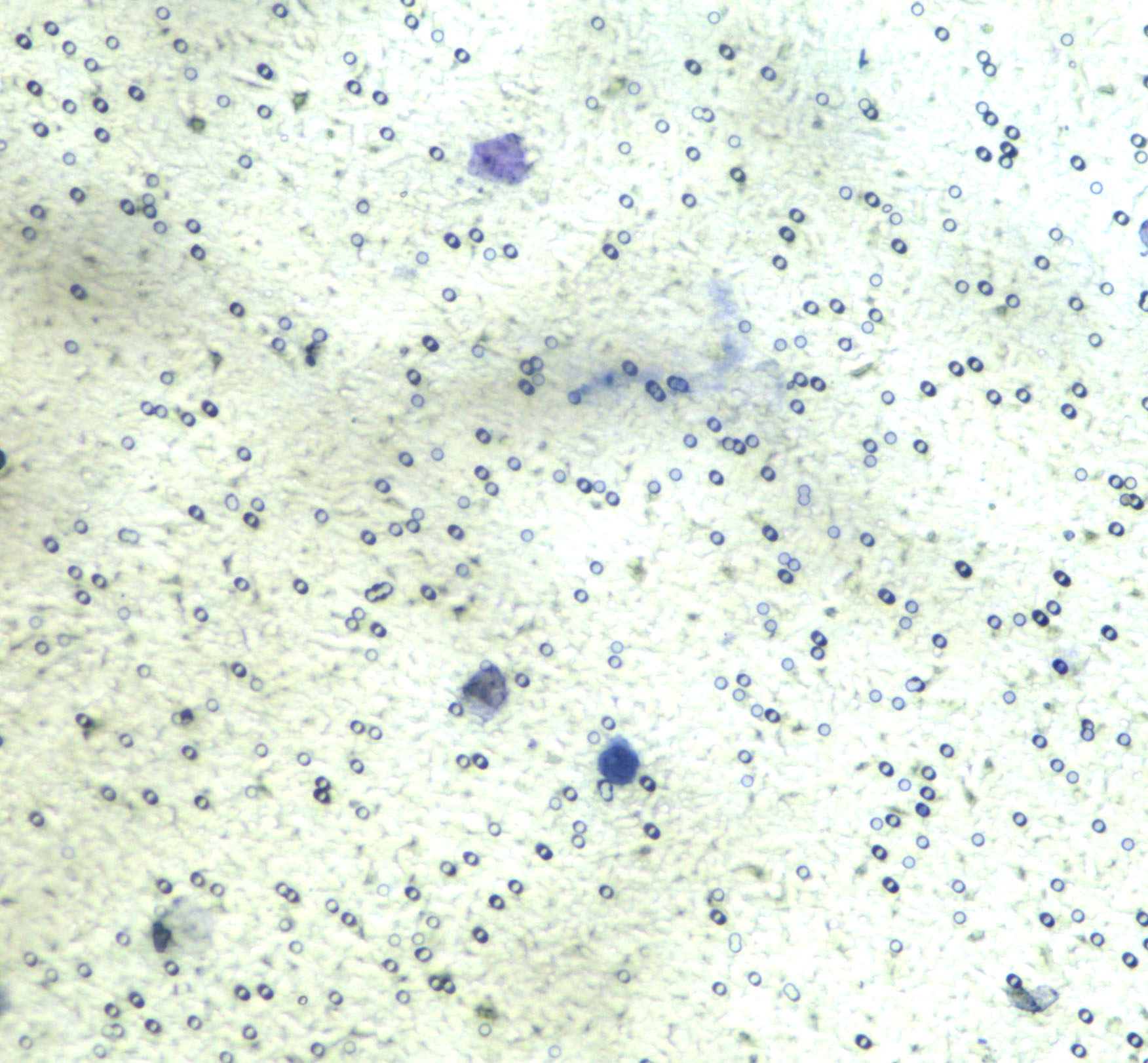

Supplement: Supplementary file 2 — Source Data for Appendix [file EMMM-15-e16592-s004.zip › Appendix/Figure S18/Fig.S18A/Primary murine hepatocyte AMD3100/3.jpg]

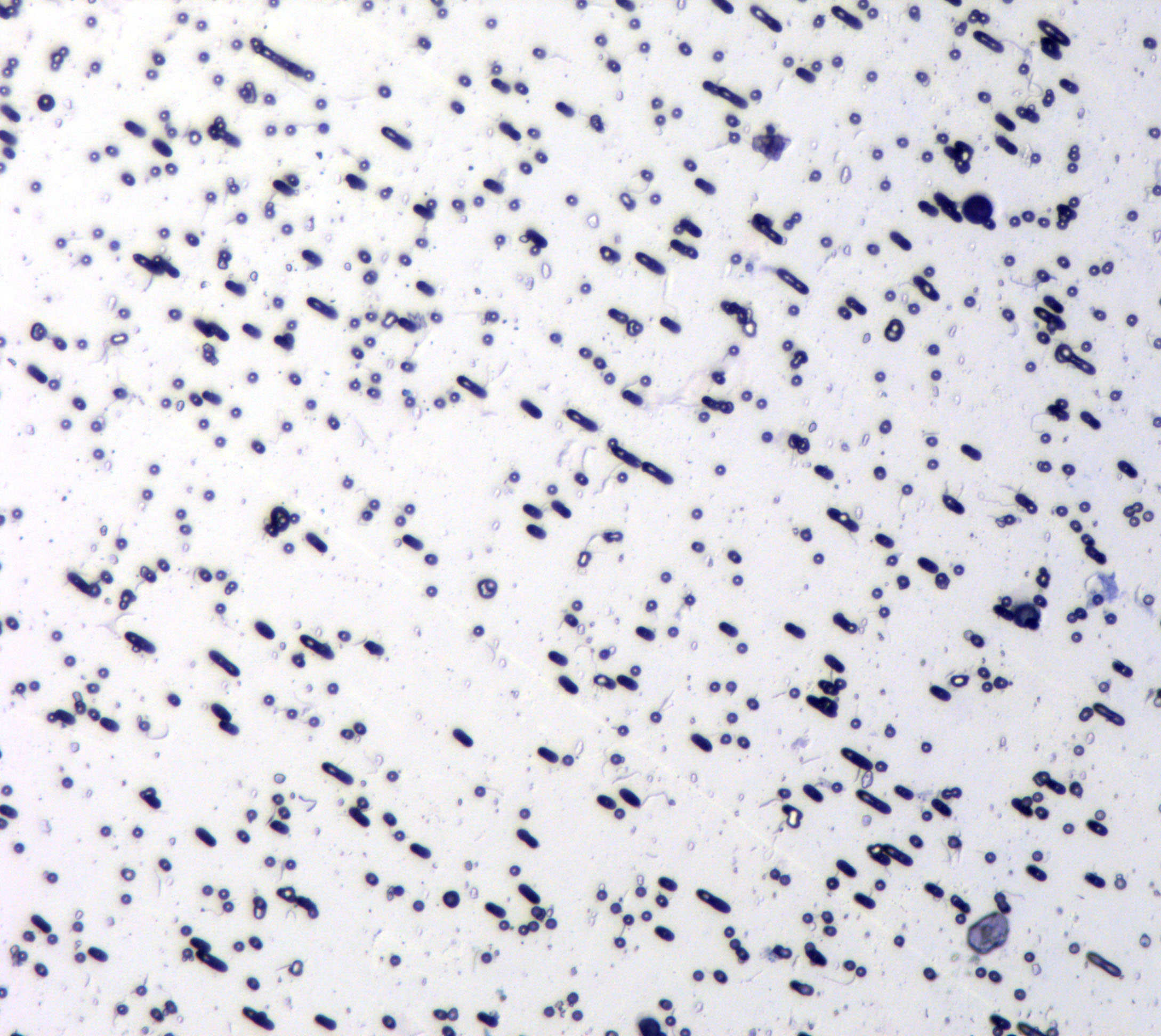

Supplement: Supplementary file 2 — Source Data for Appendix [file EMMM-15-e16592-s004.zip › Appendix/Figure S18/Fig.S18B/HepaRG AMD3100/1.jpg]

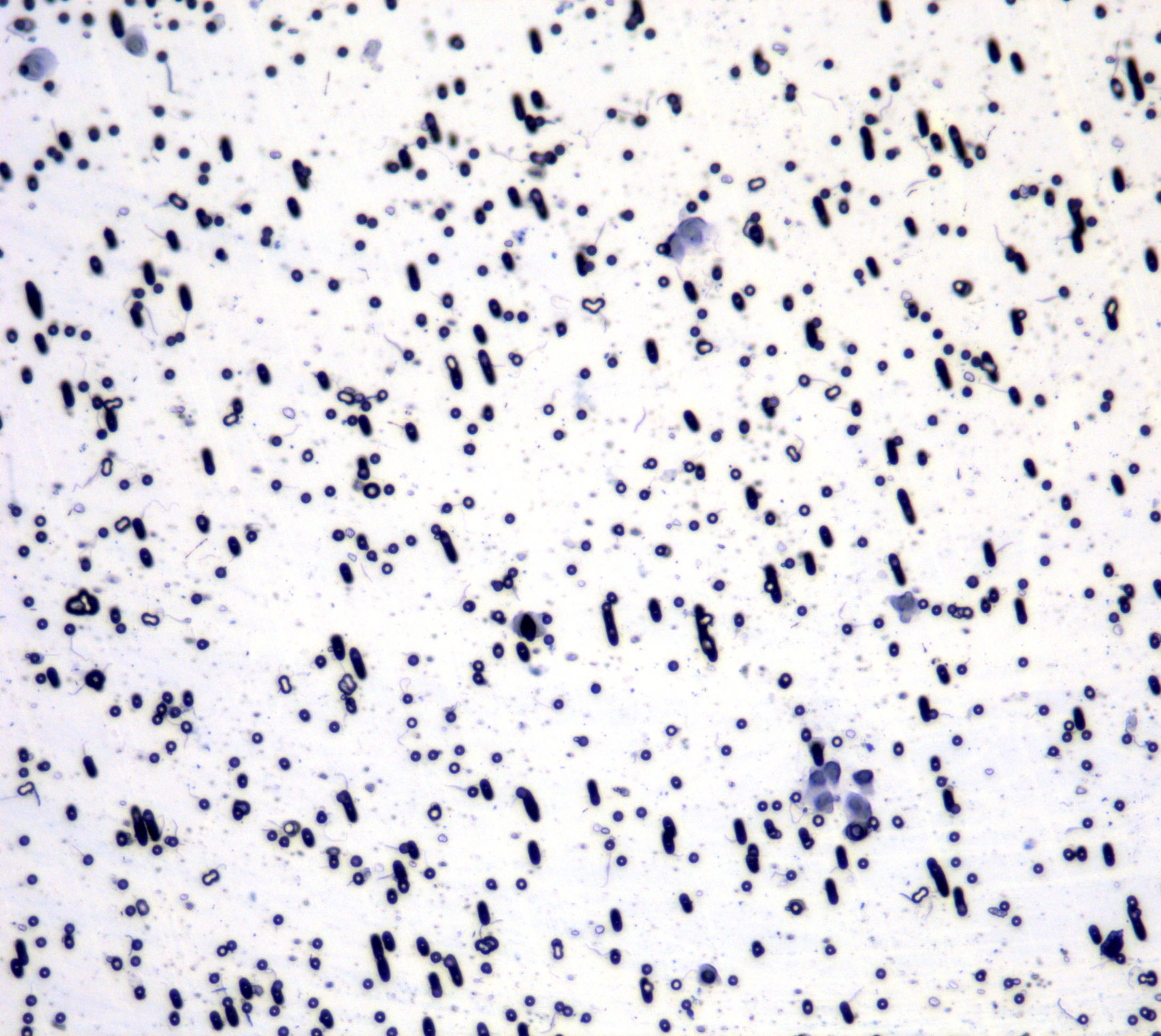

Supplement: Supplementary file 2 — Source Data for Appendix [file EMMM-15-e16592-s004.zip › Appendix/Figure S18/Fig.S18B/HepaRG AMD3100/2.jpg]

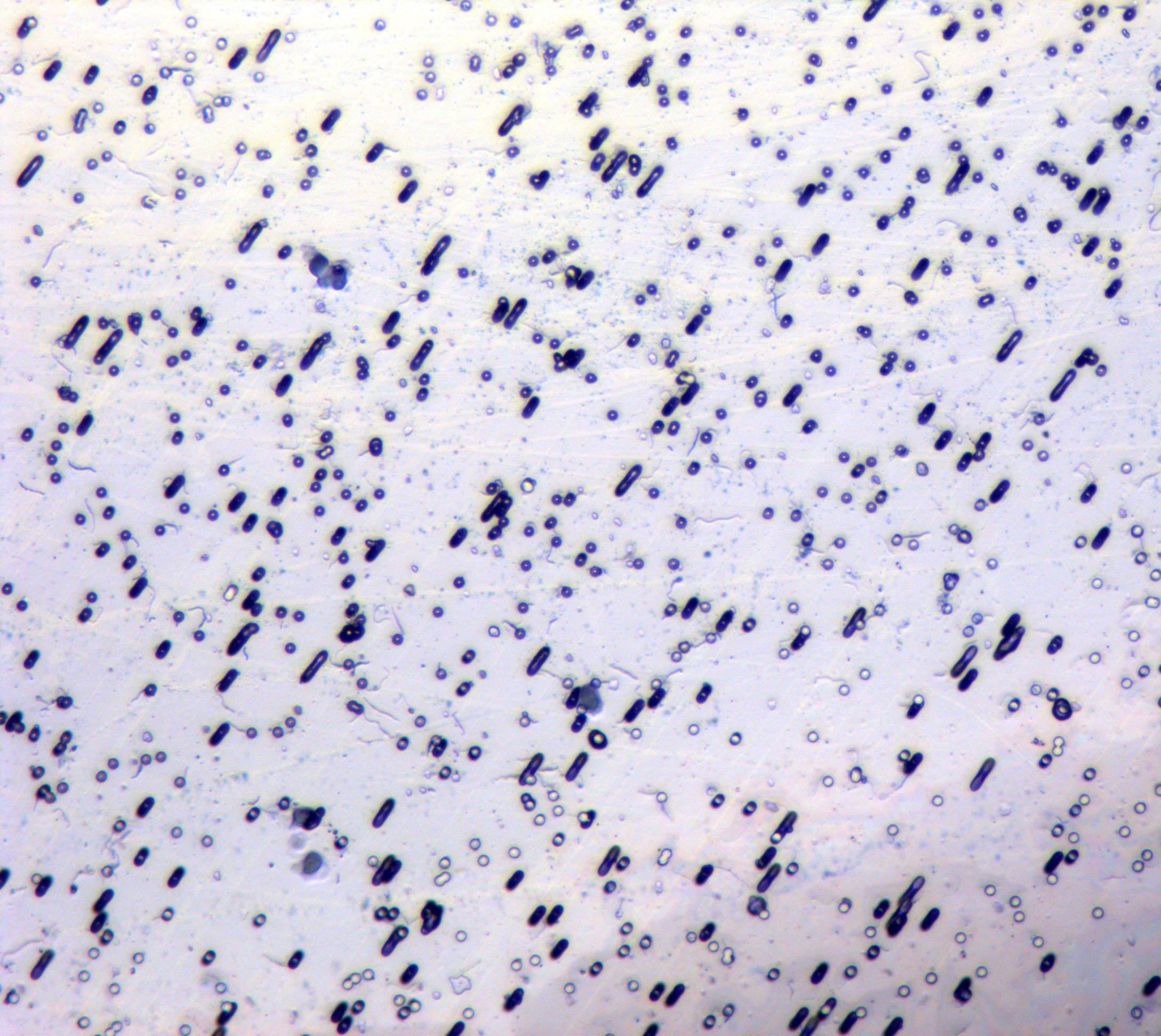

Supplement: Supplementary file 2 — Source Data for Appendix [file EMMM-15-e16592-s004.zip › Appendix/Figure S18/Fig.S18B/HepaRG AMD3100/3.jpg]

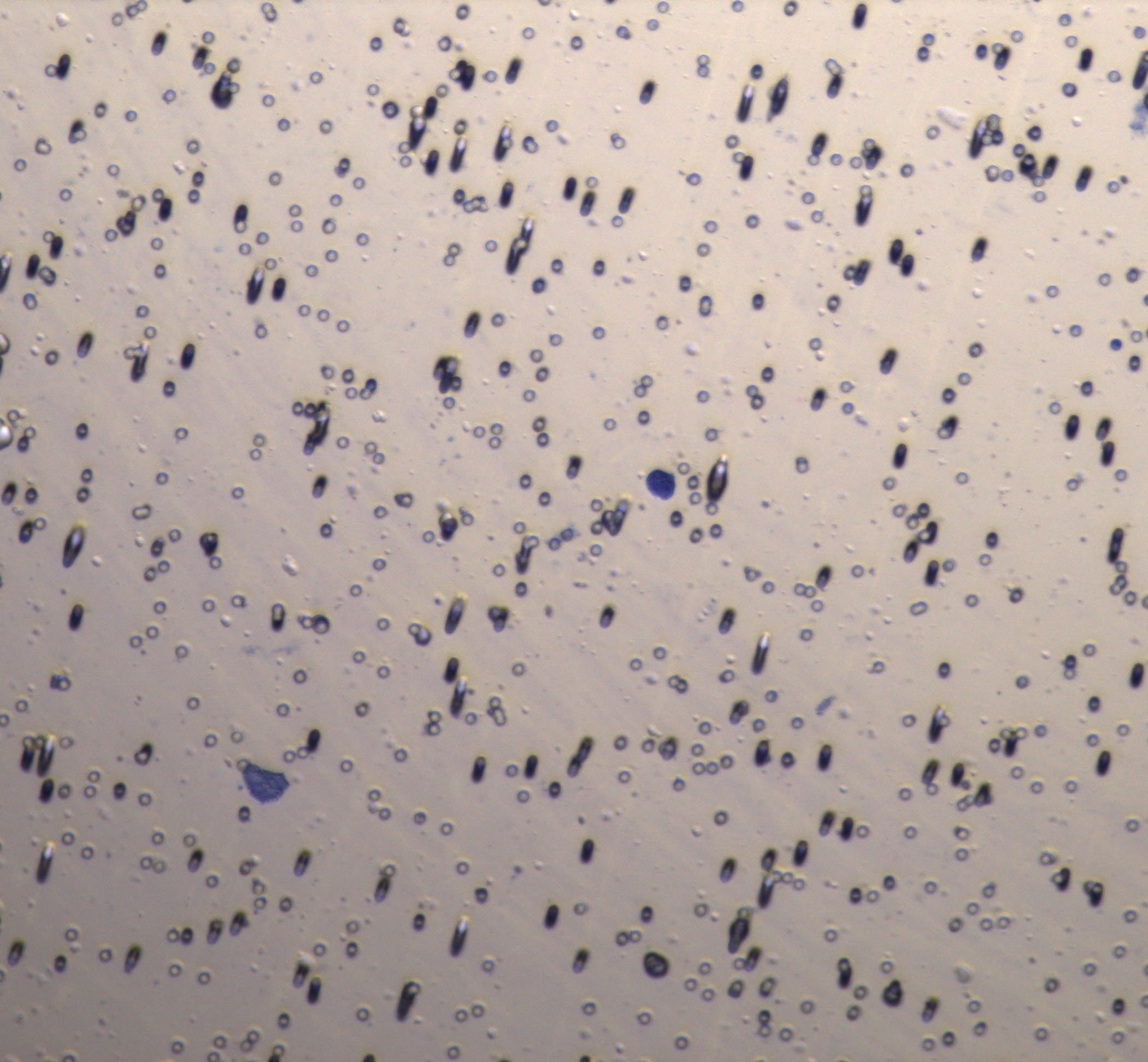

Supplement: Supplementary file 2 — Source Data for Appendix [file EMMM-15-e16592-s004.zip › Appendix/Figure S19/Fig.S19B/Primary murine hepatocyte siCxcl12/1.jpg]

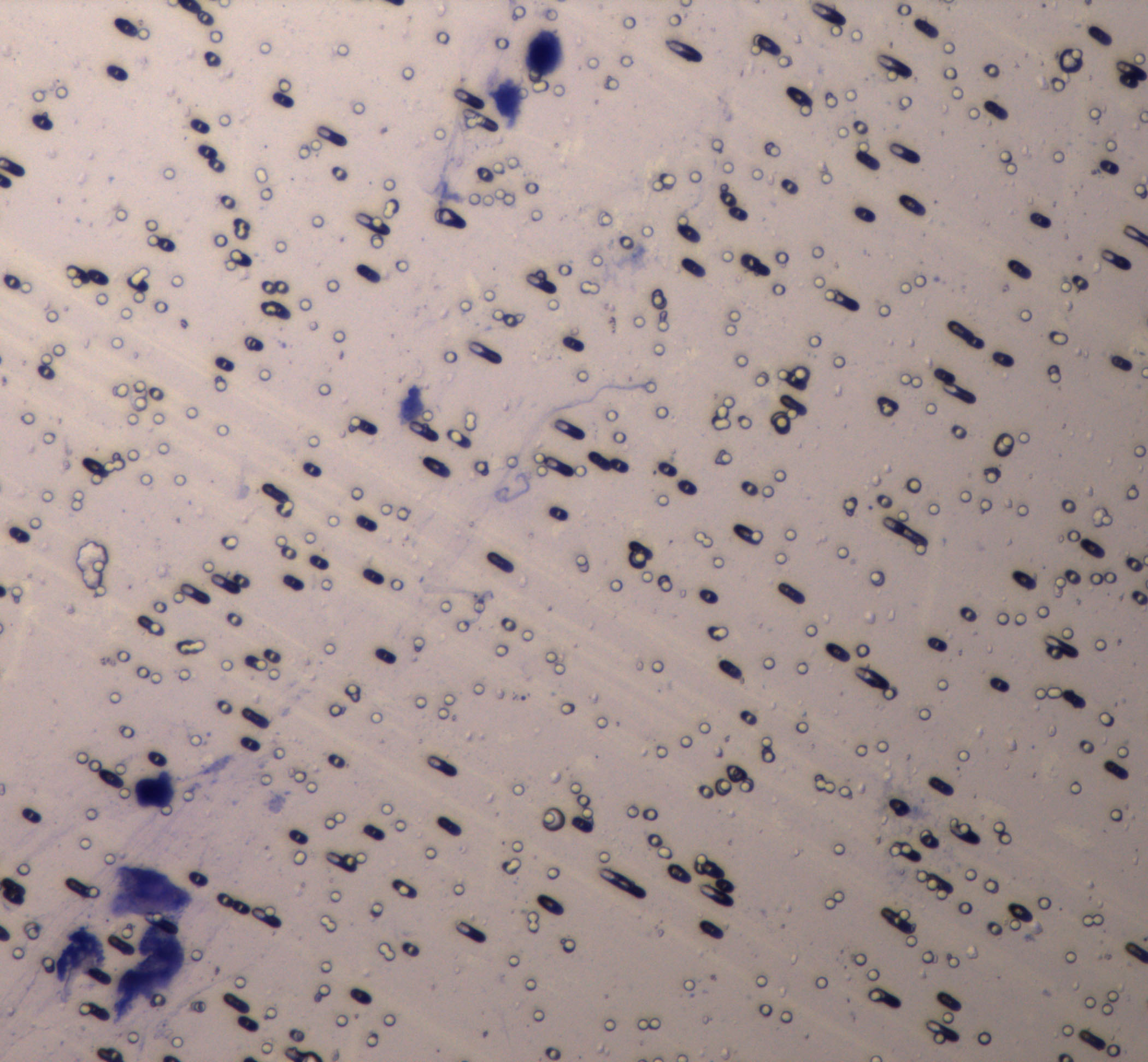

Supplement: Supplementary file 2 — Source Data for Appendix [file EMMM-15-e16592-s004.zip › Appendix/Figure S19/Fig.S19B/Primary murine hepatocyte siCxcl12/2.jpg]

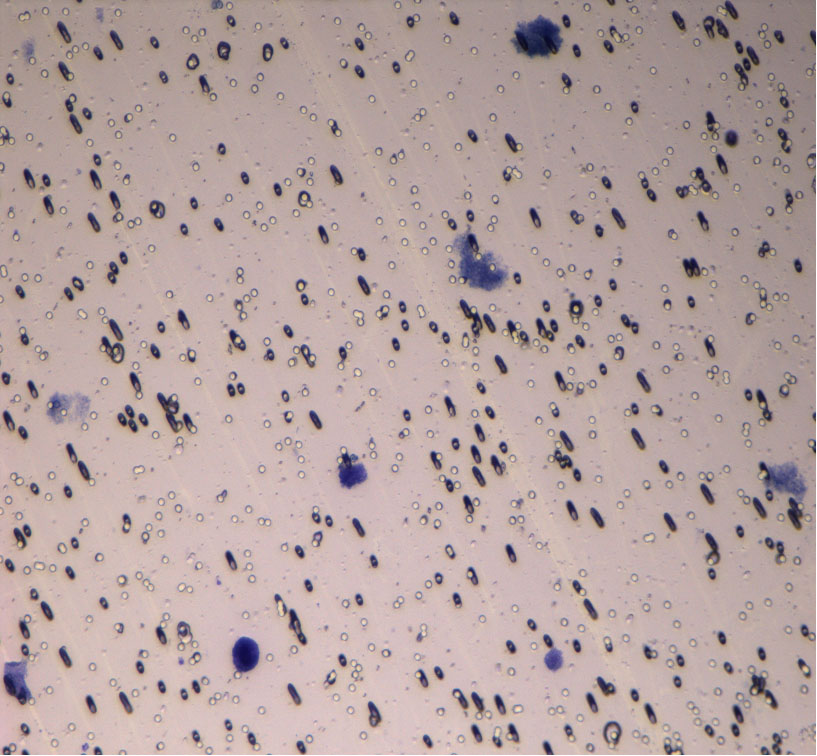

Supplement: Supplementary file 2 — Source Data for Appendix [file EMMM-15-e16592-s004.zip › Appendix/Figure S19/Fig.S19B/Primary murine hepatocyte siCxcl12/3.jpg]

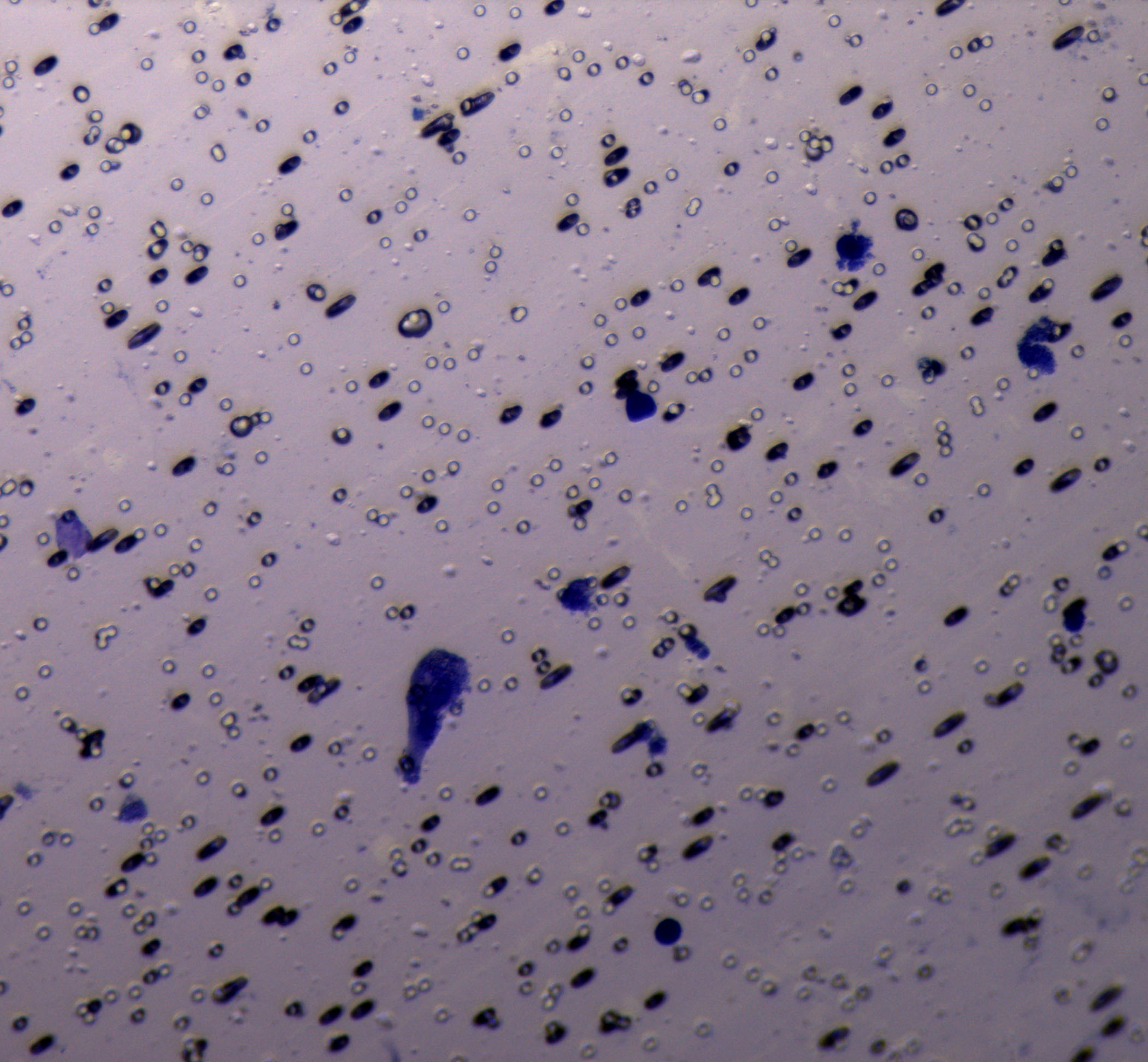

Supplement: Supplementary file 2 — Source Data for Appendix [file EMMM-15-e16592-s004.zip › Appendix/Figure S19/Fig.S19B/Primary murine hepatocyte siCxcl12/4.jpg]

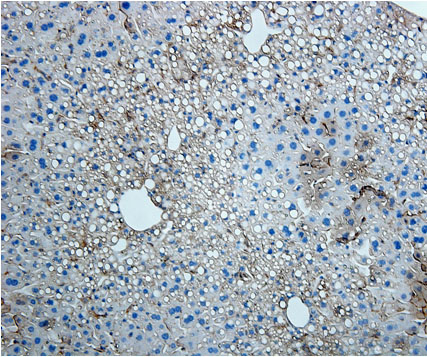

Supplement: Supplementary file 2 — Source Data for Appendix [file EMMM-15-e16592-s004.zip › Appendix/Figure S7/1.jpg]

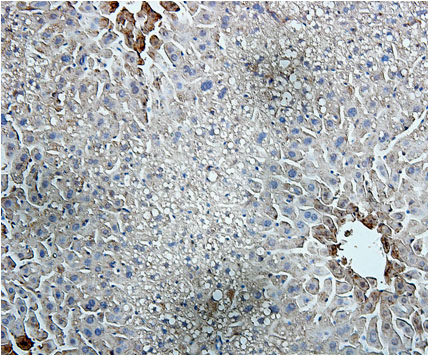

Supplement: Supplementary file 2 — Source Data for Appendix [file EMMM-15-e16592-s004.zip › Appendix/Figure S7/2.jpg]

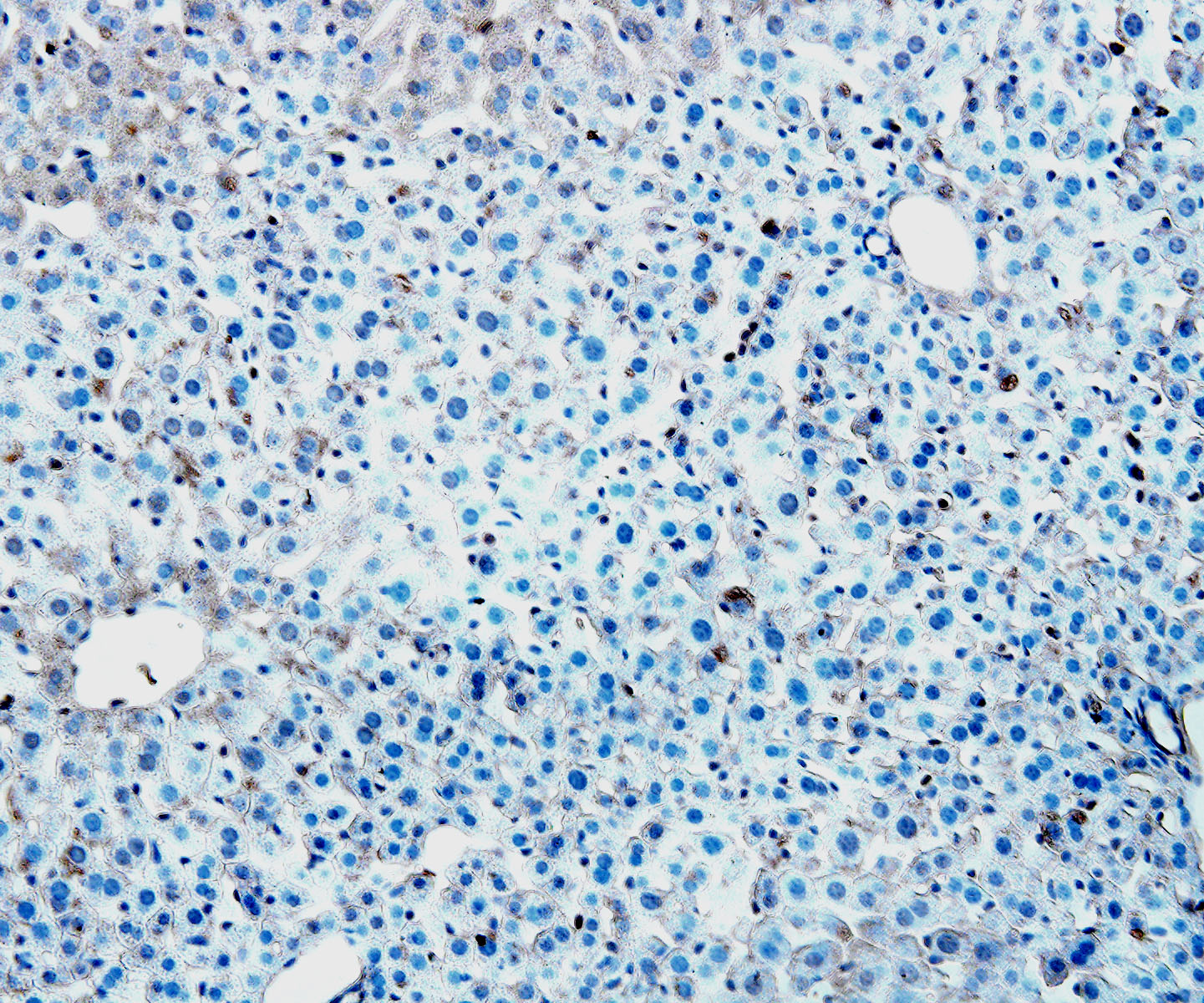

Supplement: Supplementary file 2 — Source Data for Appendix [file EMMM-15-e16592-s004.zip › Appendix/Figure S7/3.jpg]

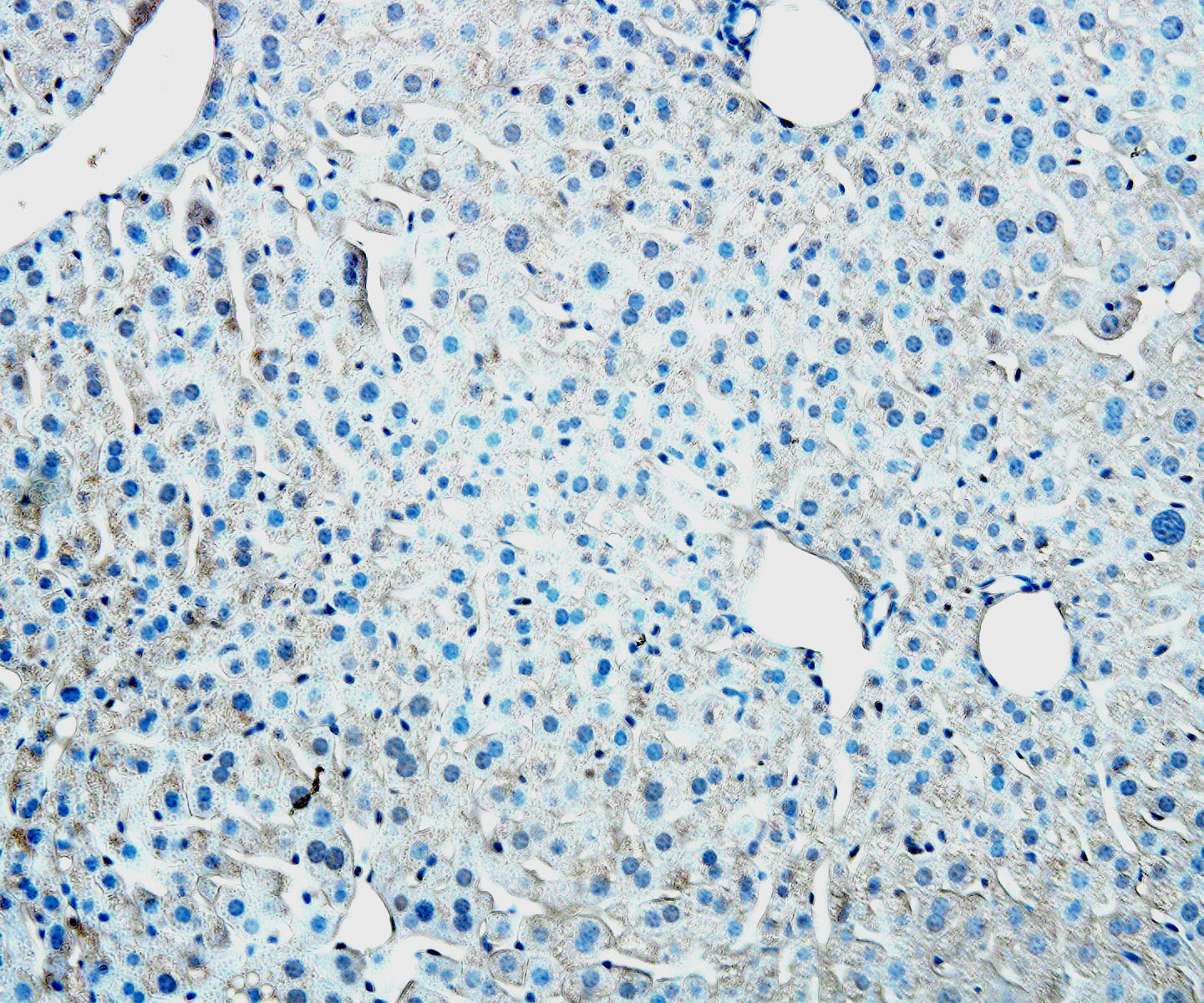

Supplement: Supplementary file 2 — Source Data for Appendix [file EMMM-15-e16592-s004.zip › Appendix/Figure S7/4.jpg]

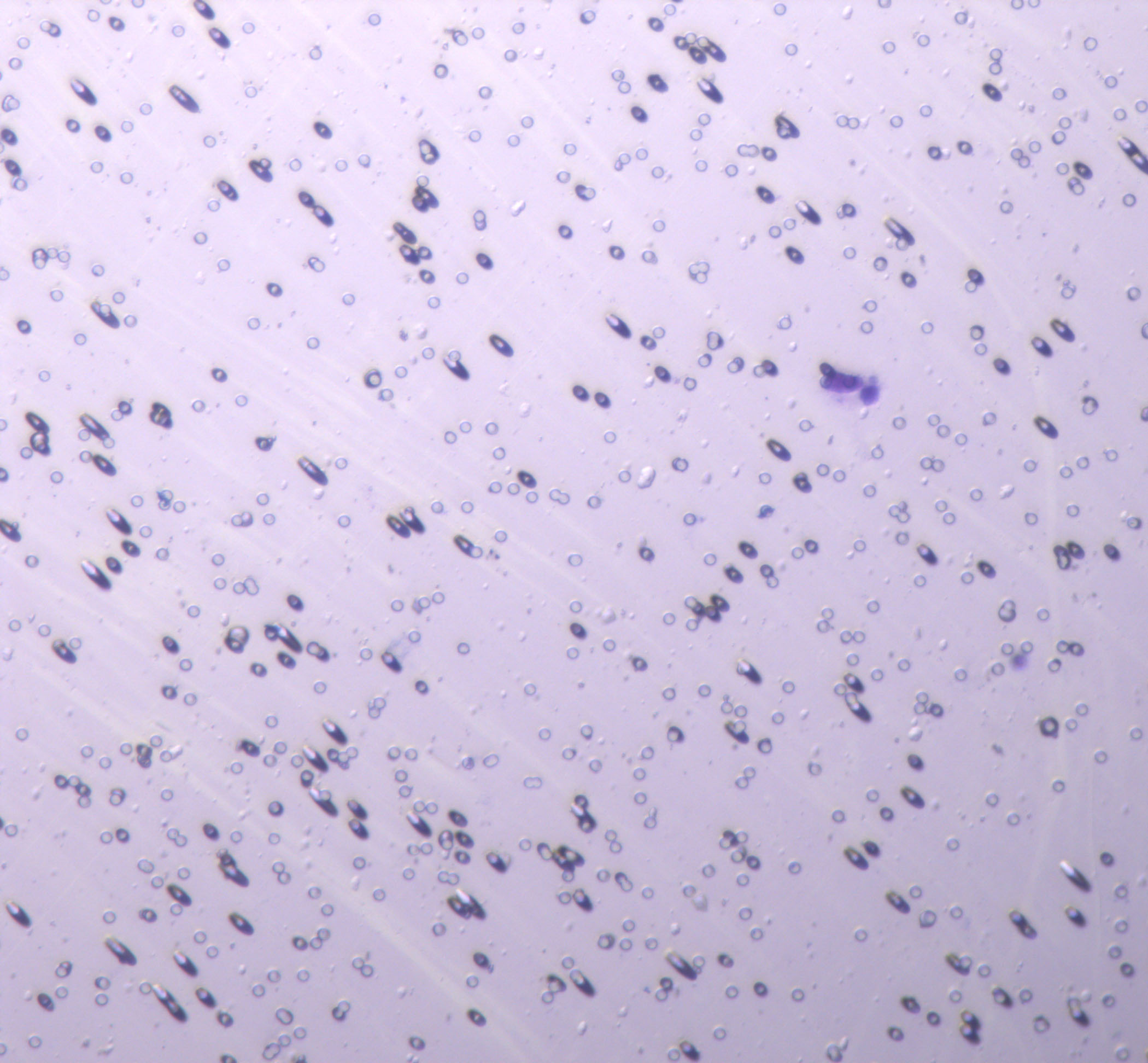

Supplement: Supplementary file 2 — Source Data for Appendix [file EMMM-15-e16592-s004.zip › Appendix/Figure S8/HepaRG siBRG1/1.jpg]

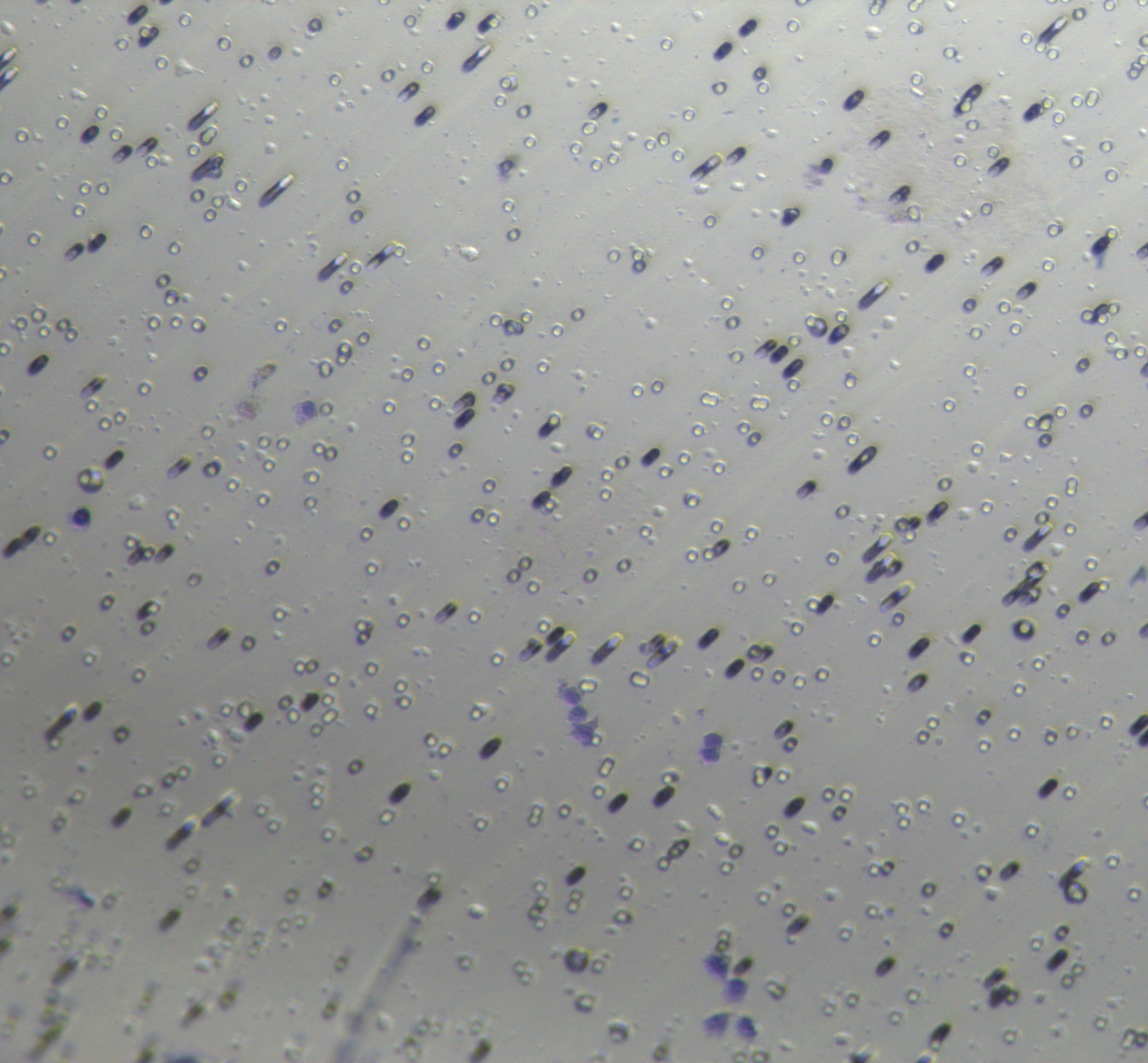

Supplement: Supplementary file 2 — Source Data for Appendix [file EMMM-15-e16592-s004.zip › Appendix/Figure S8/HepaRG siBRG1/2.jpg]

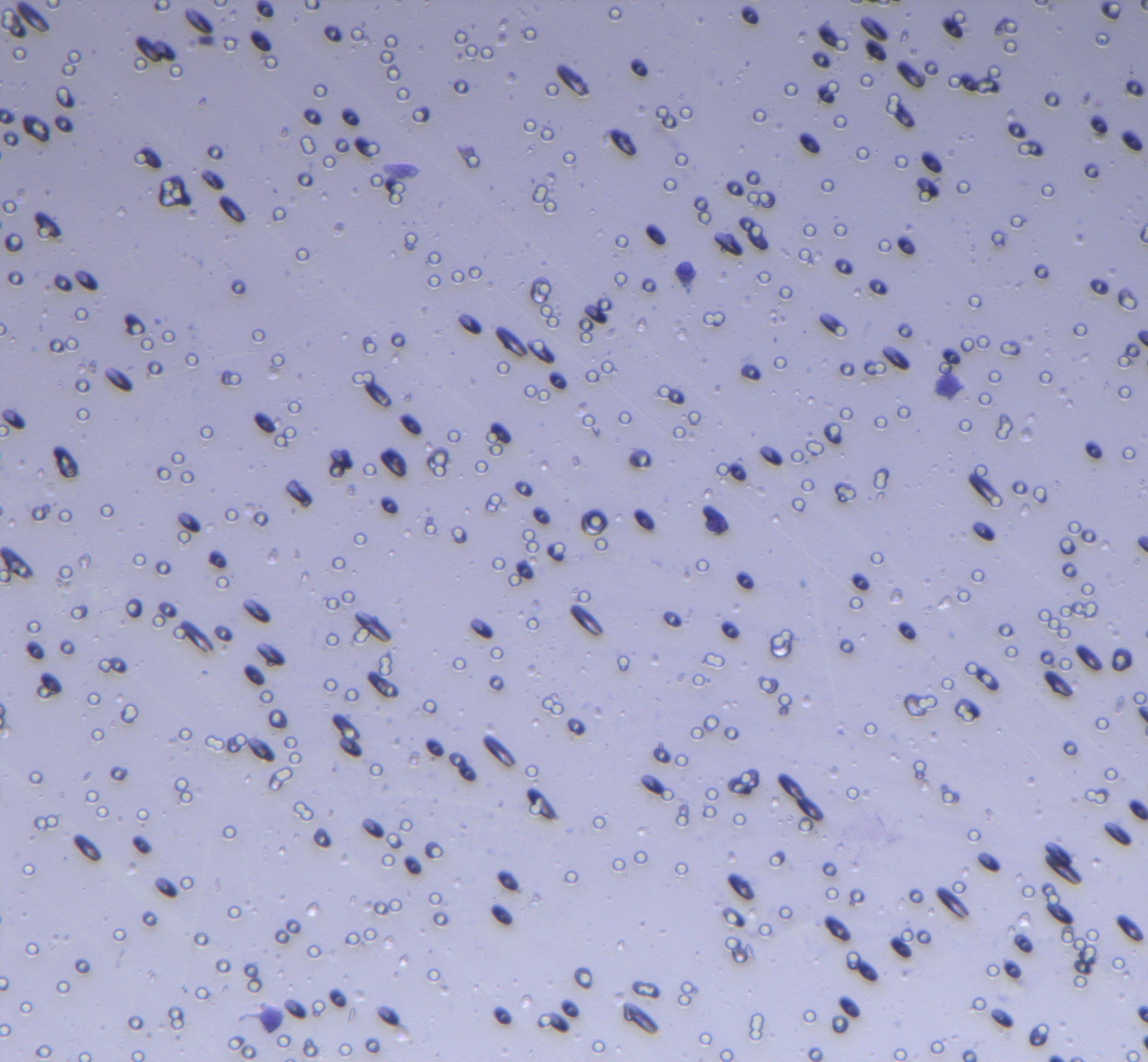

Supplement: Supplementary file 2 — Source Data for Appendix [file EMMM-15-e16592-s004.zip › Appendix/Figure S8/HepaRG siBRG1/3.jpg]

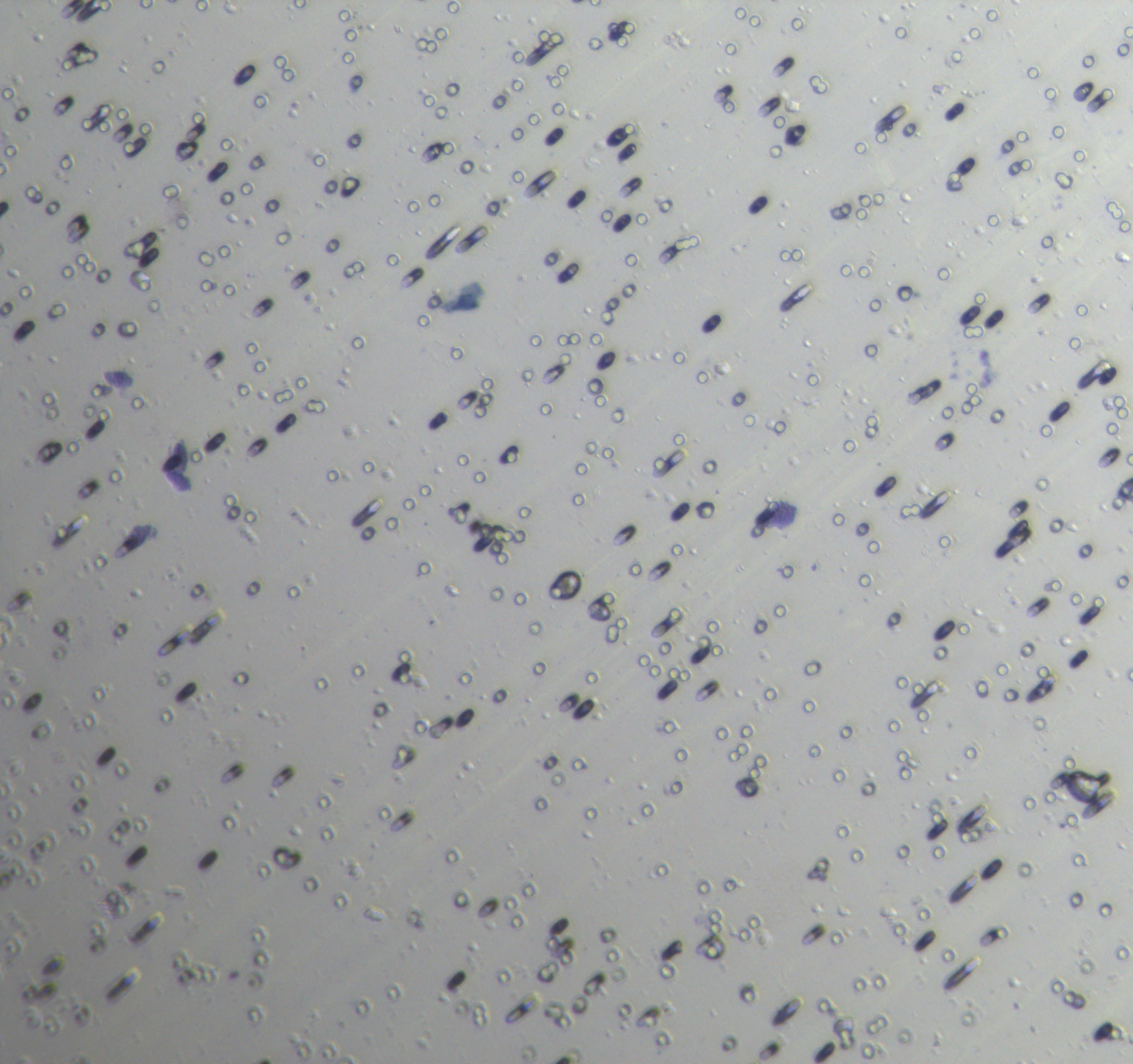

Supplement: Supplementary file 2 — Source Data for Appendix [file EMMM-15-e16592-s004.zip › Appendix/Figure S8/HepaRG siBRG1/4.jpg]

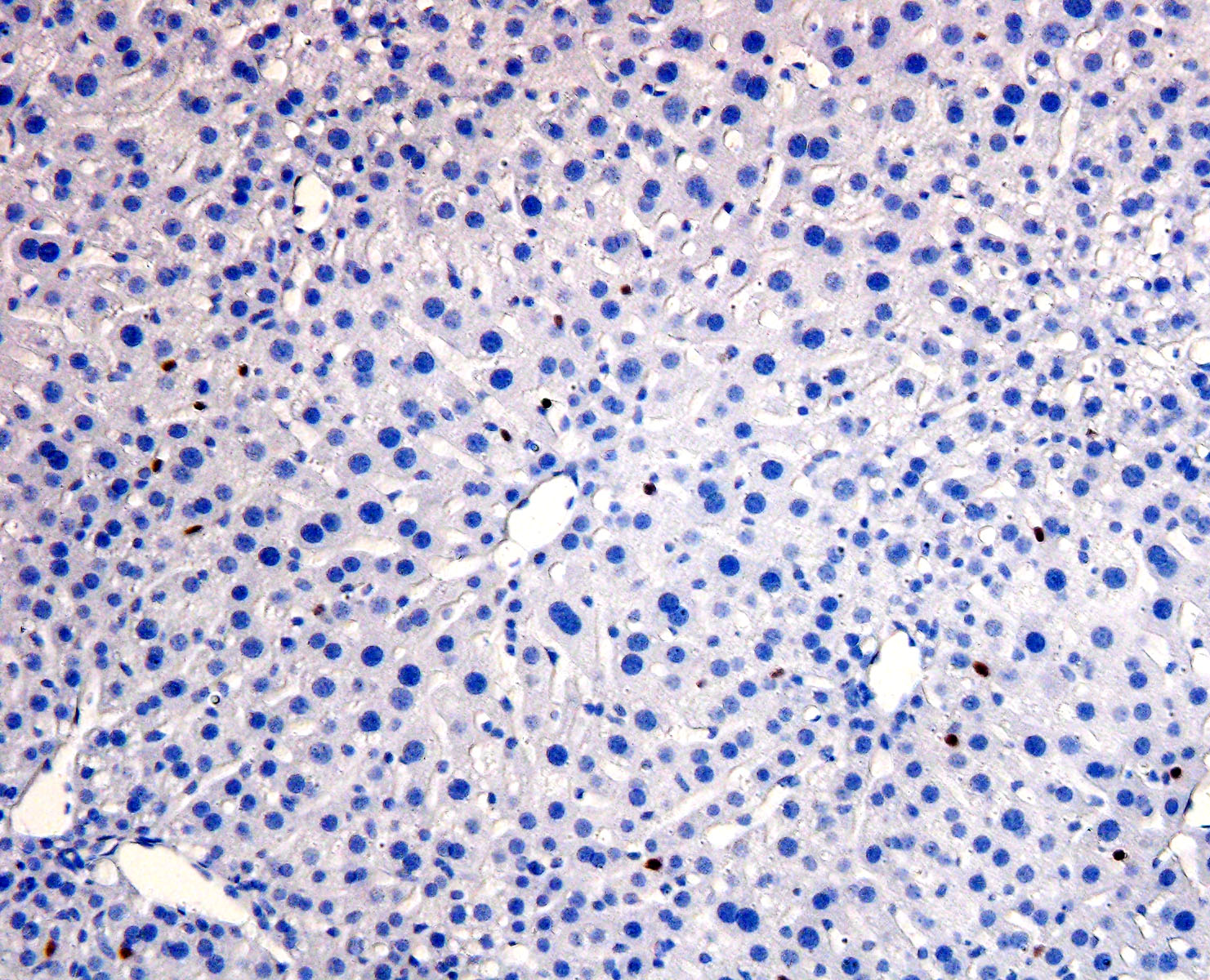

Supplement: Supplementary file 2 — Source Data for Appendix [file EMMM-15-e16592-s004.zip › Appendix/Figure S9/1.jpg]

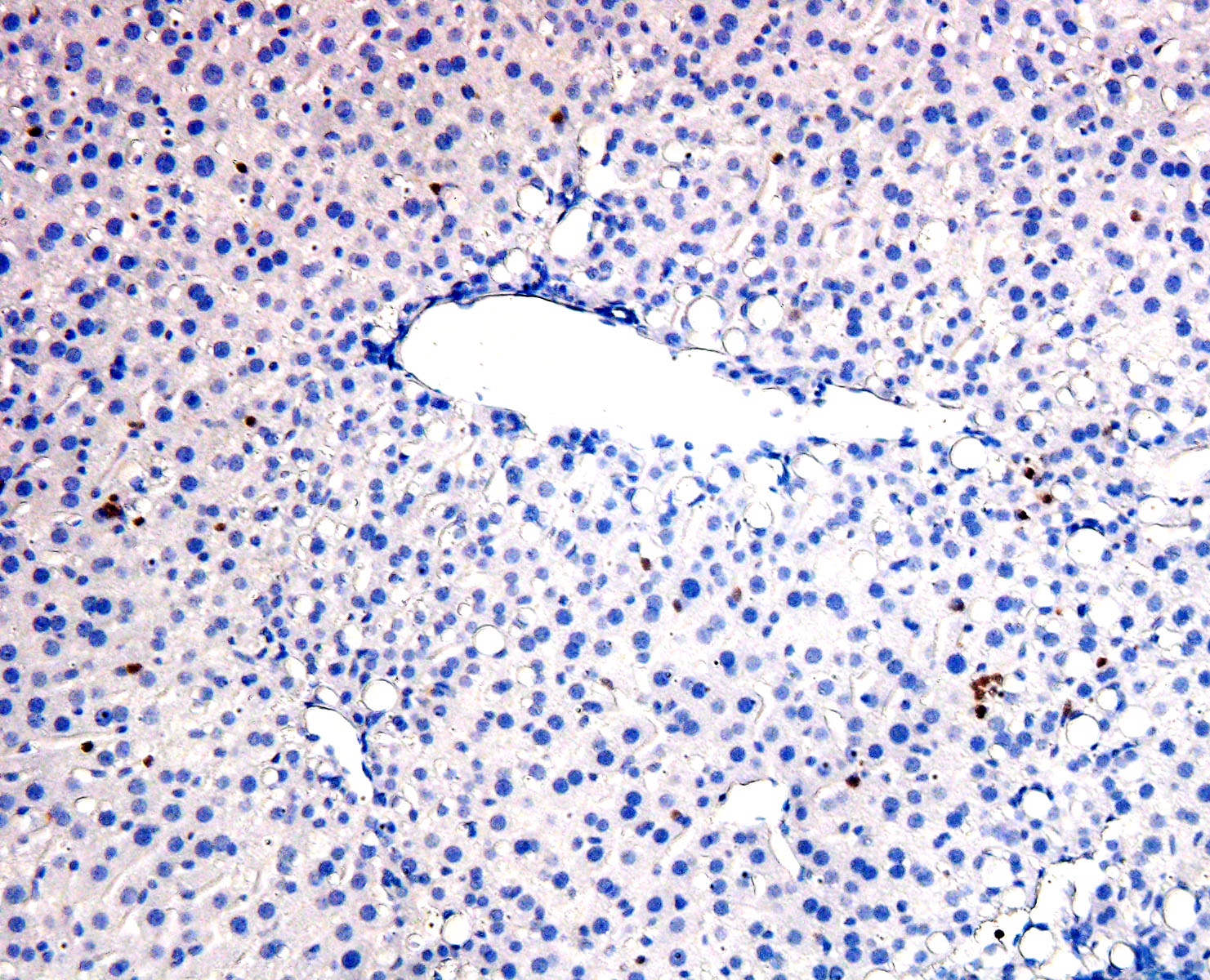

Supplement: Supplementary file 2 — Source Data for Appendix [file EMMM-15-e16592-s004.zip › Appendix/Figure S9/2.jpg]

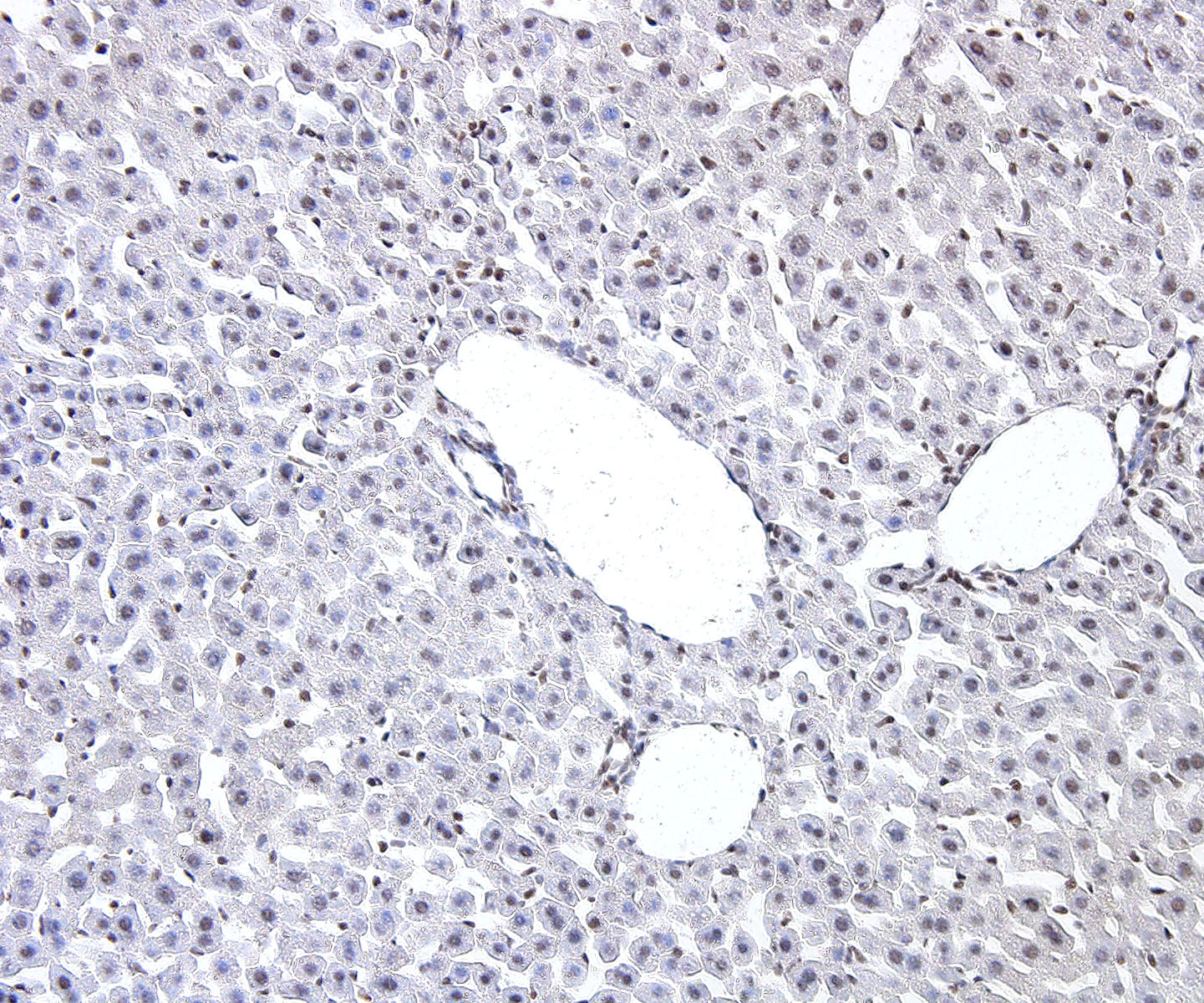

Supplement: Supplementary file 3 — Source Data for Figure 1 [file EMMM-15-e16592-s002.zip › Figure 1/Fig.1D/1.jpg]

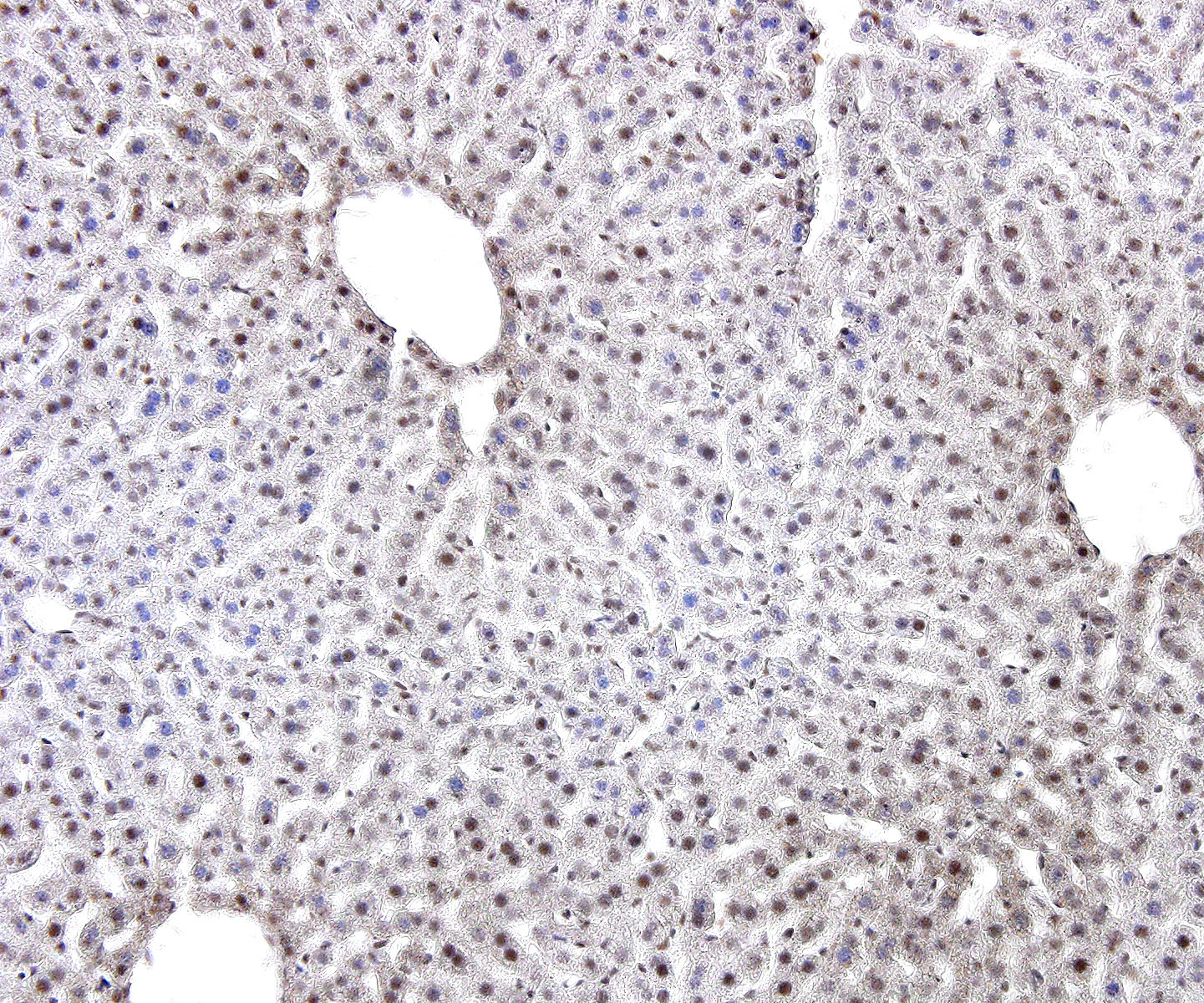

Supplement: Supplementary file 3 — Source Data for Figure 1 [file EMMM-15-e16592-s002.zip › Figure 1/Fig.1D/2.jpg]

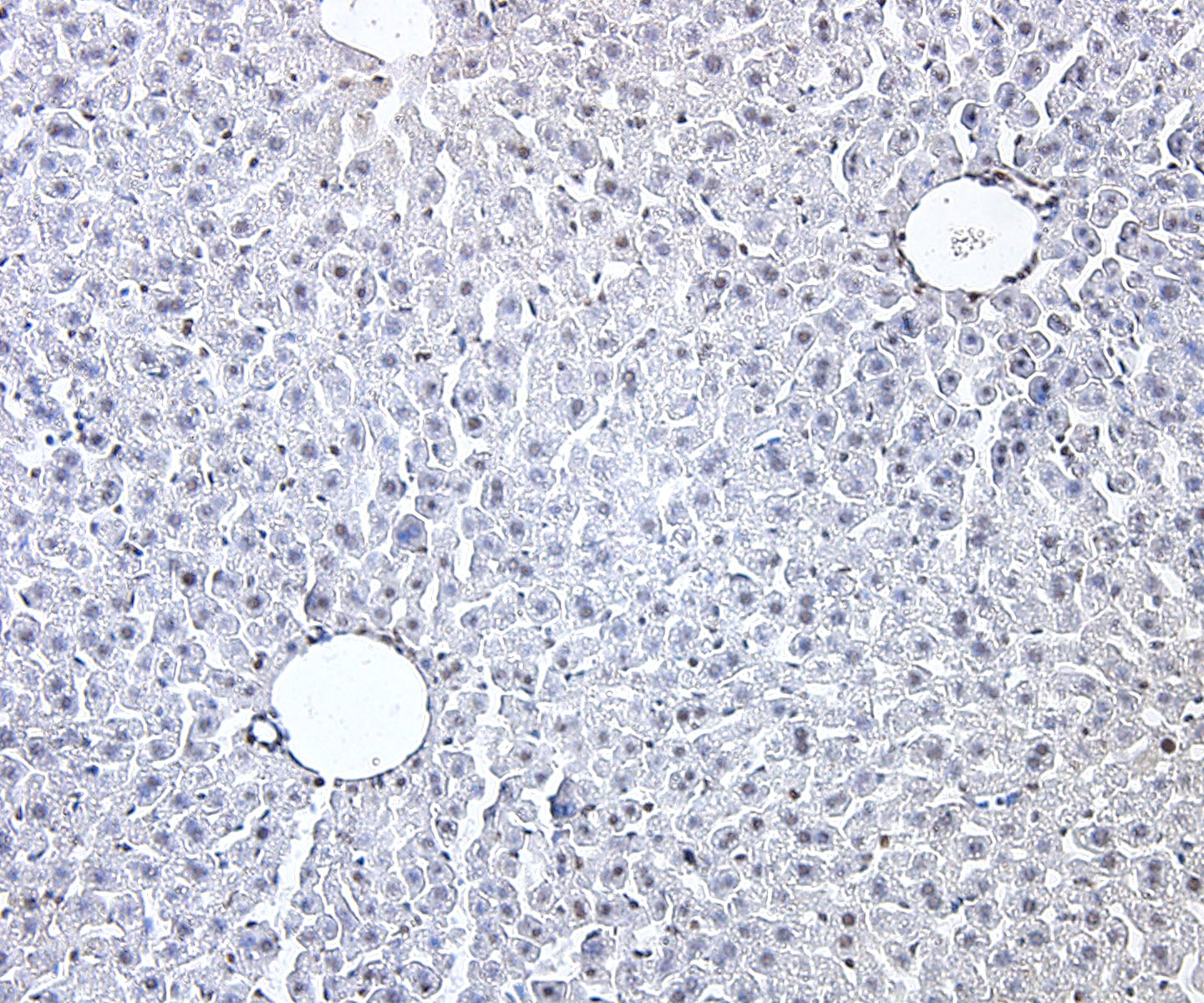

Supplement: Supplementary file 3 — Source Data for Figure 1 [file EMMM-15-e16592-s002.zip › Figure 1/Fig.1H/1.jpg]

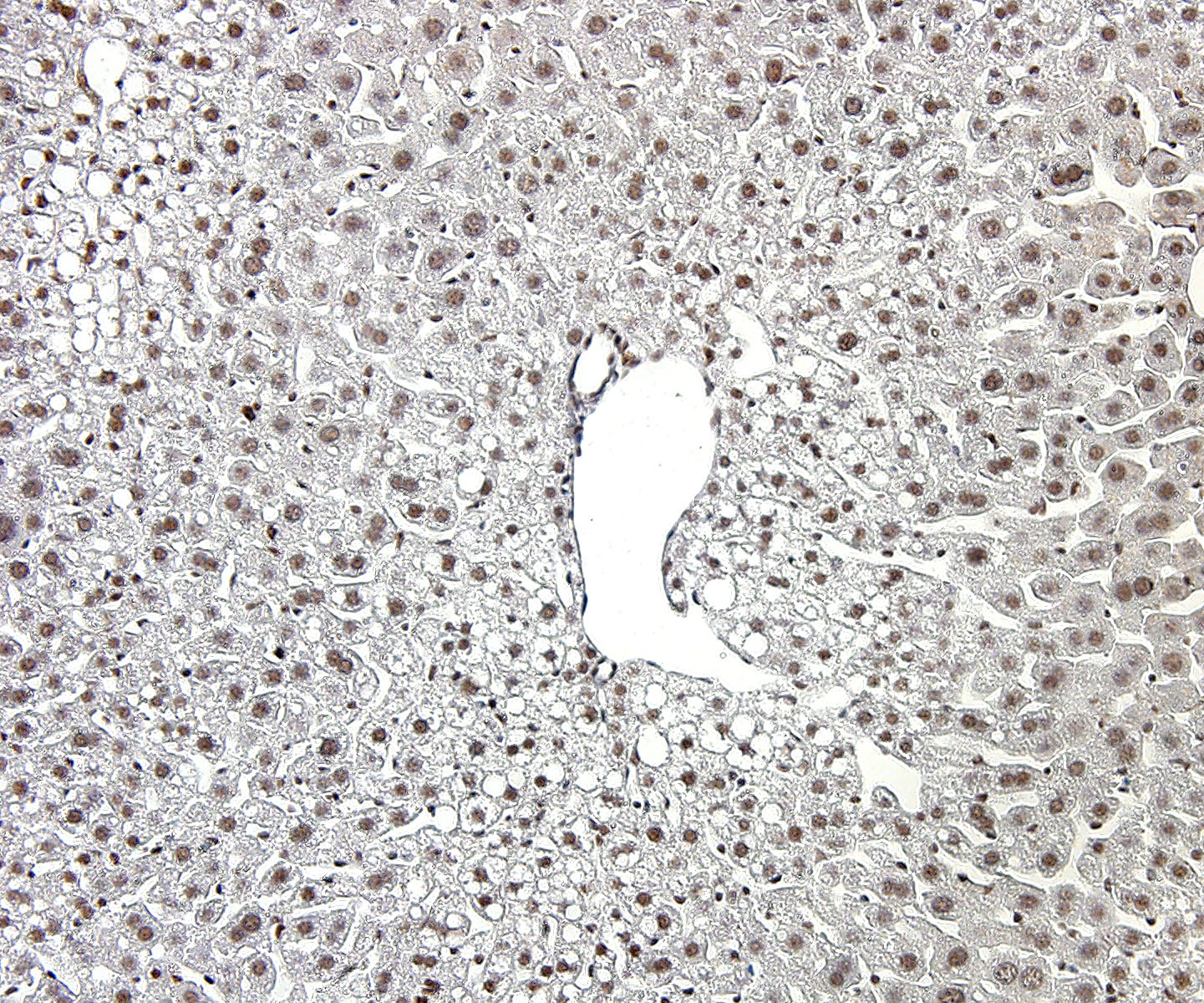

Supplement: Supplementary file 3 — Source Data for Figure 1 [file EMMM-15-e16592-s002.zip › Figure 1/Fig.1H/2.jpg]

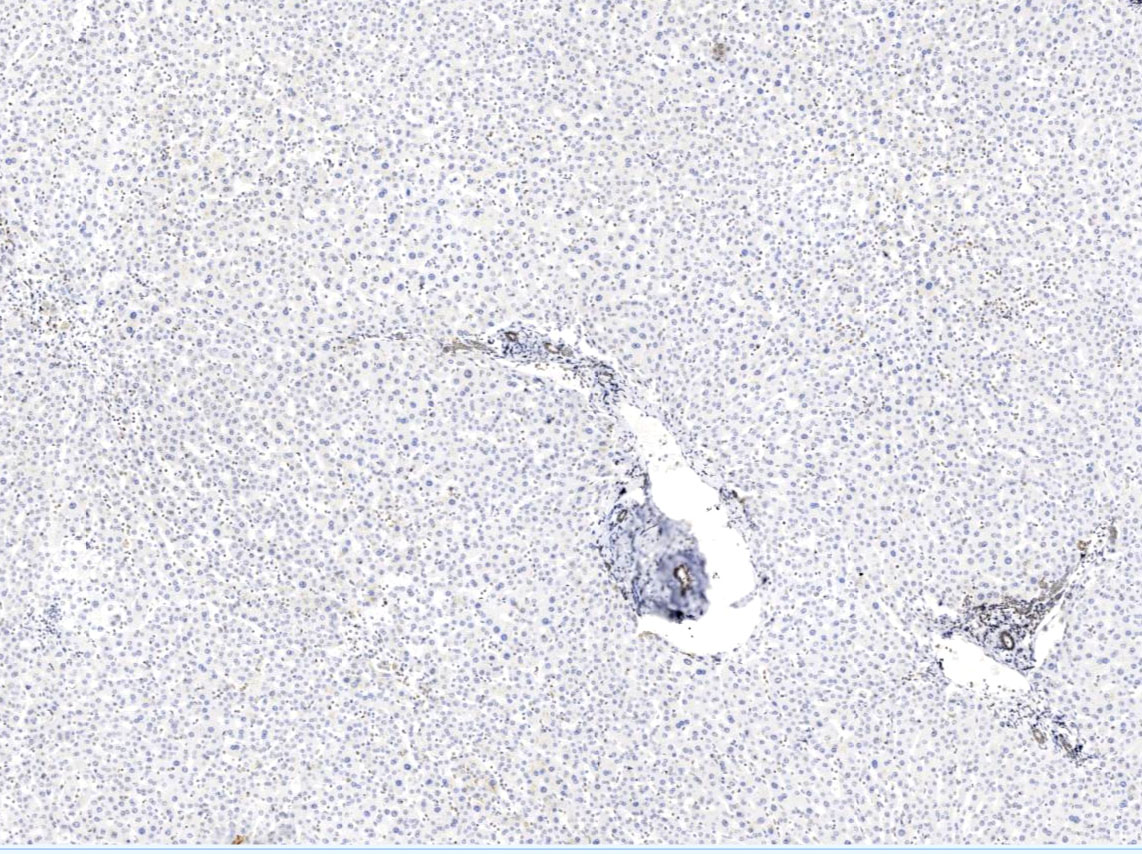

Supplement: Supplementary file 3 — Source Data for Figure 1 [file EMMM-15-e16592-s002.zip › Figure 1/Fig.1K/1.jpg]

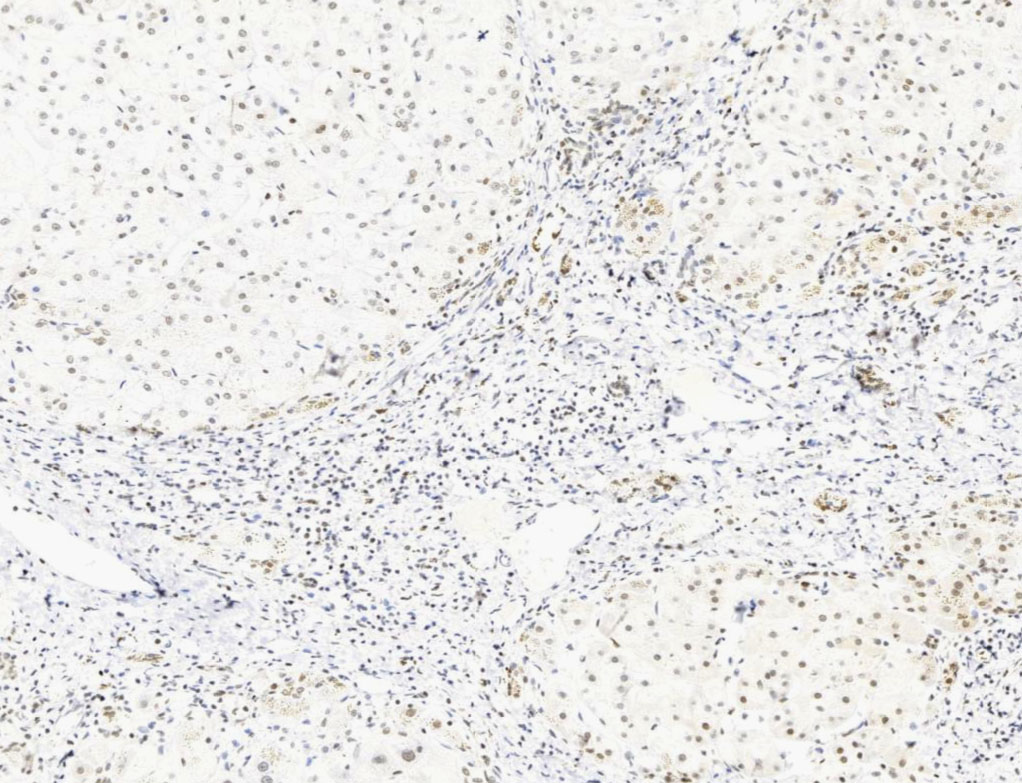

Supplement: Supplementary file 3 — Source Data for Figure 1 [file EMMM-15-e16592-s002.zip › Figure 1/Fig.1K/2.jpg]

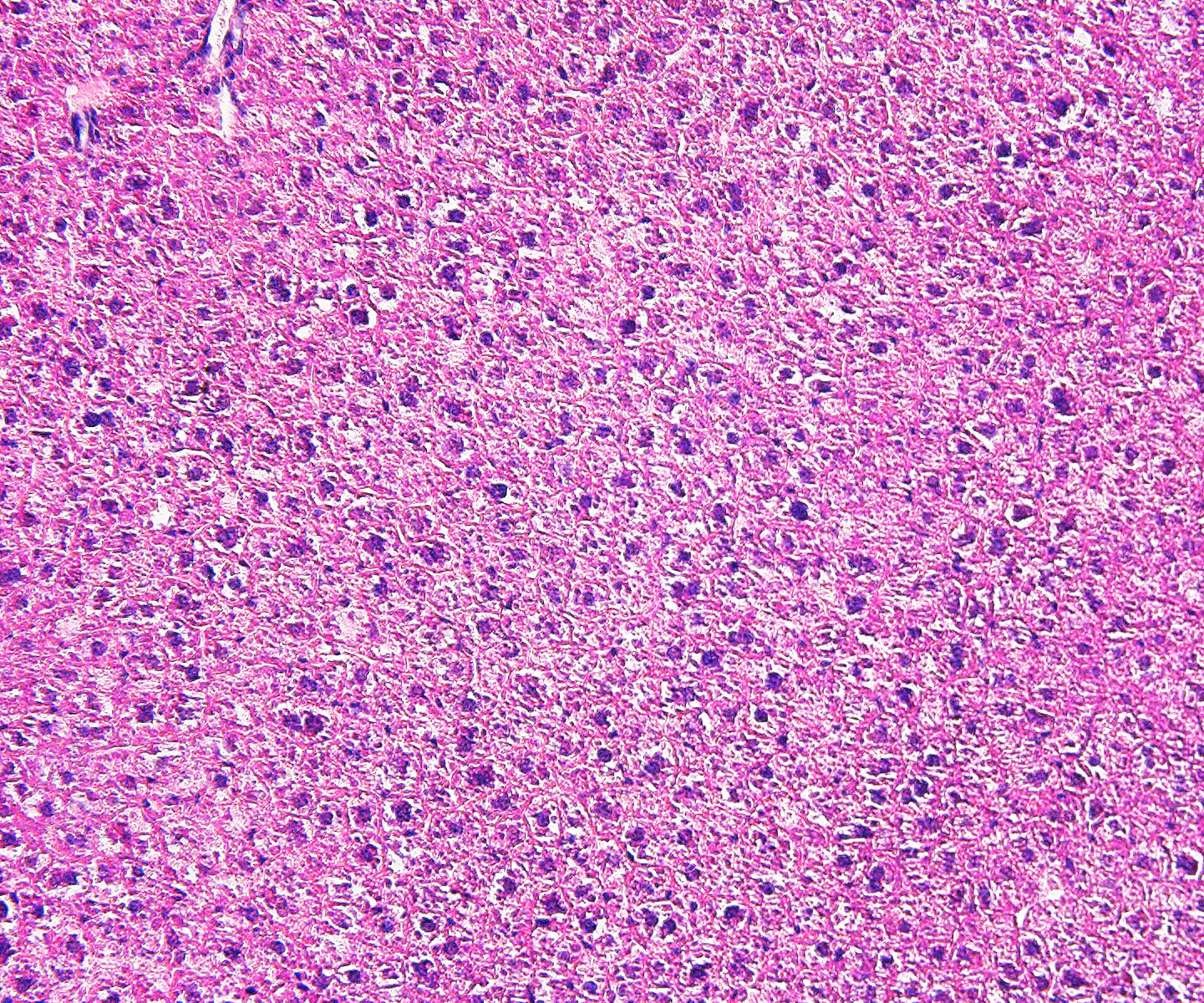

Supplement: Supplementary file 4 — Source Data for Figure 2 [file EMMM-15-e16592-s008.zip › Figure 2/Fig.2D/1.jpg]

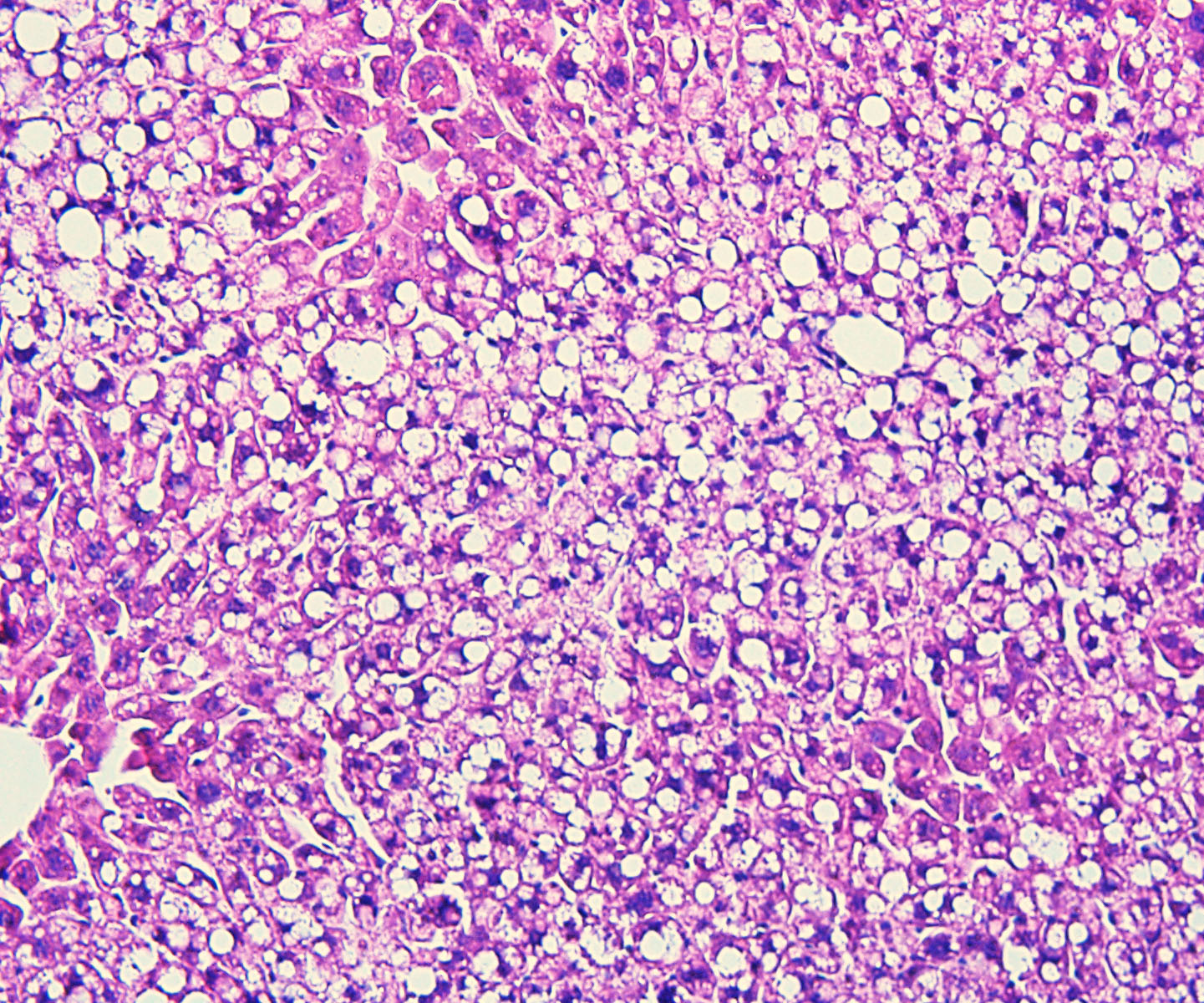

Supplement: Supplementary file 4 — Source Data for Figure 2 [file EMMM-15-e16592-s008.zip › Figure 2/Fig.2D/2.jpg]

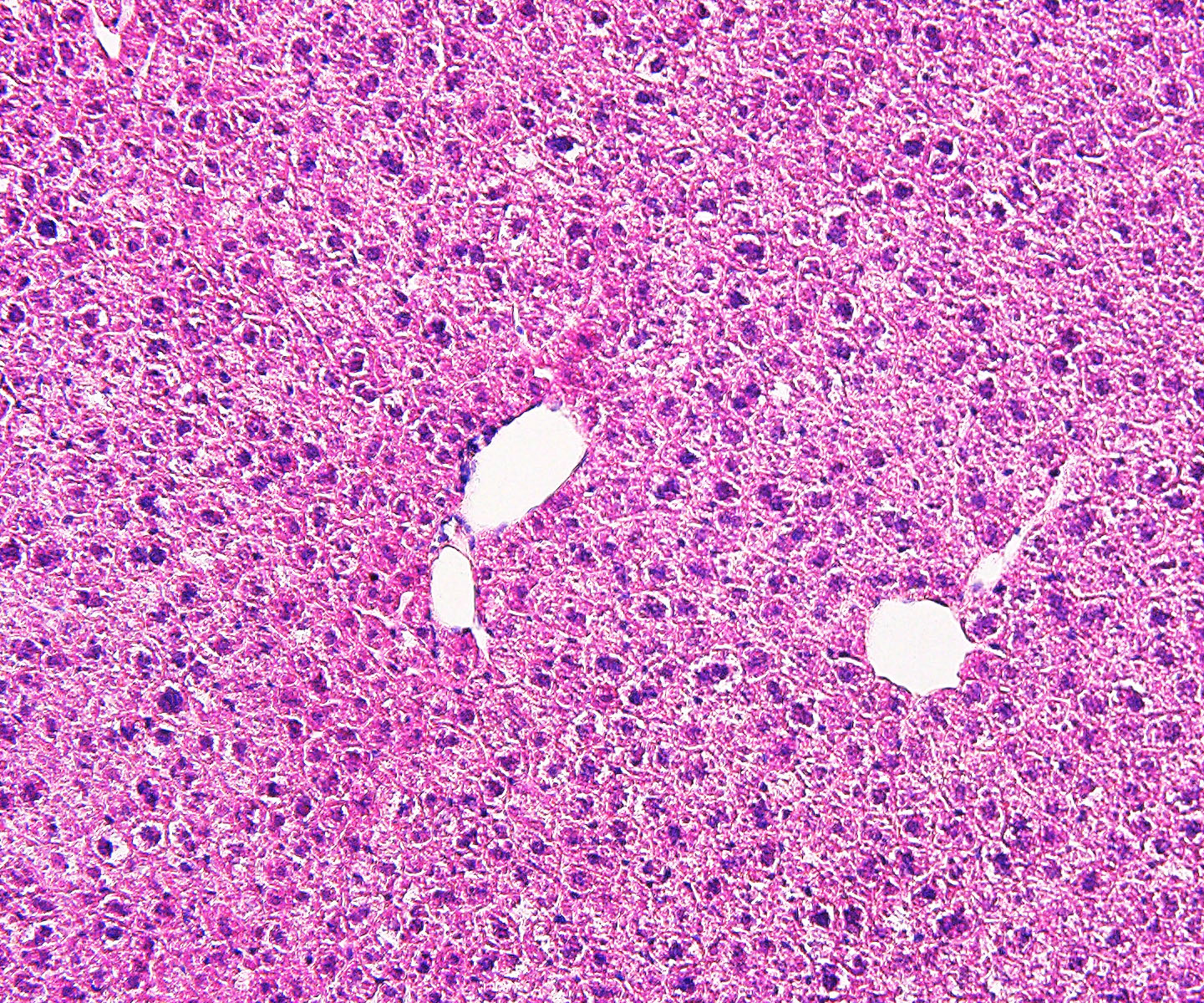

Supplement: Supplementary file 4 — Source Data for Figure 2 [file EMMM-15-e16592-s008.zip › Figure 2/Fig.2D/3.jpg]

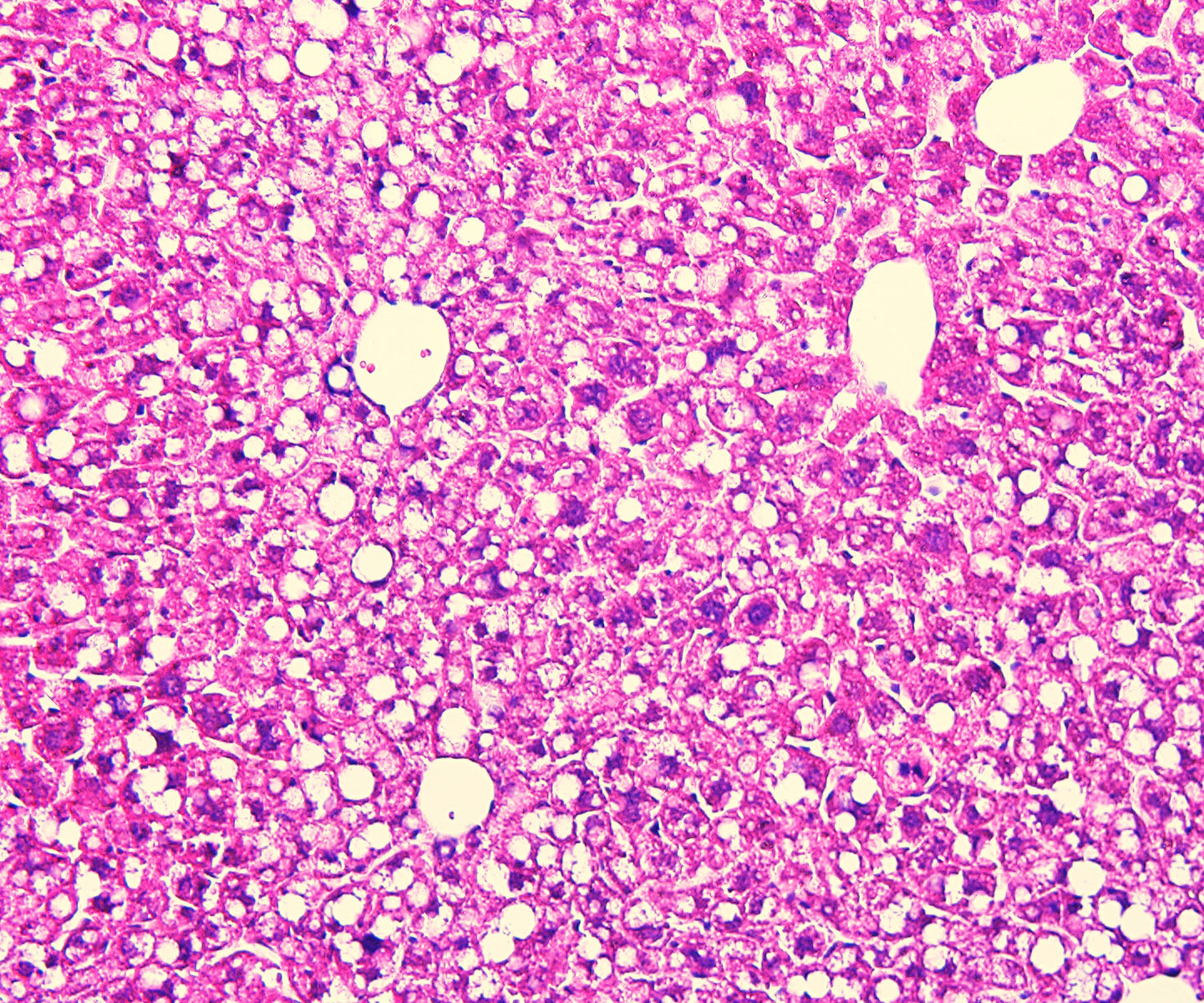

Supplement: Supplementary file 4 — Source Data for Figure 2 [file EMMM-15-e16592-s008.zip › Figure 2/Fig.2D/4.jpg]

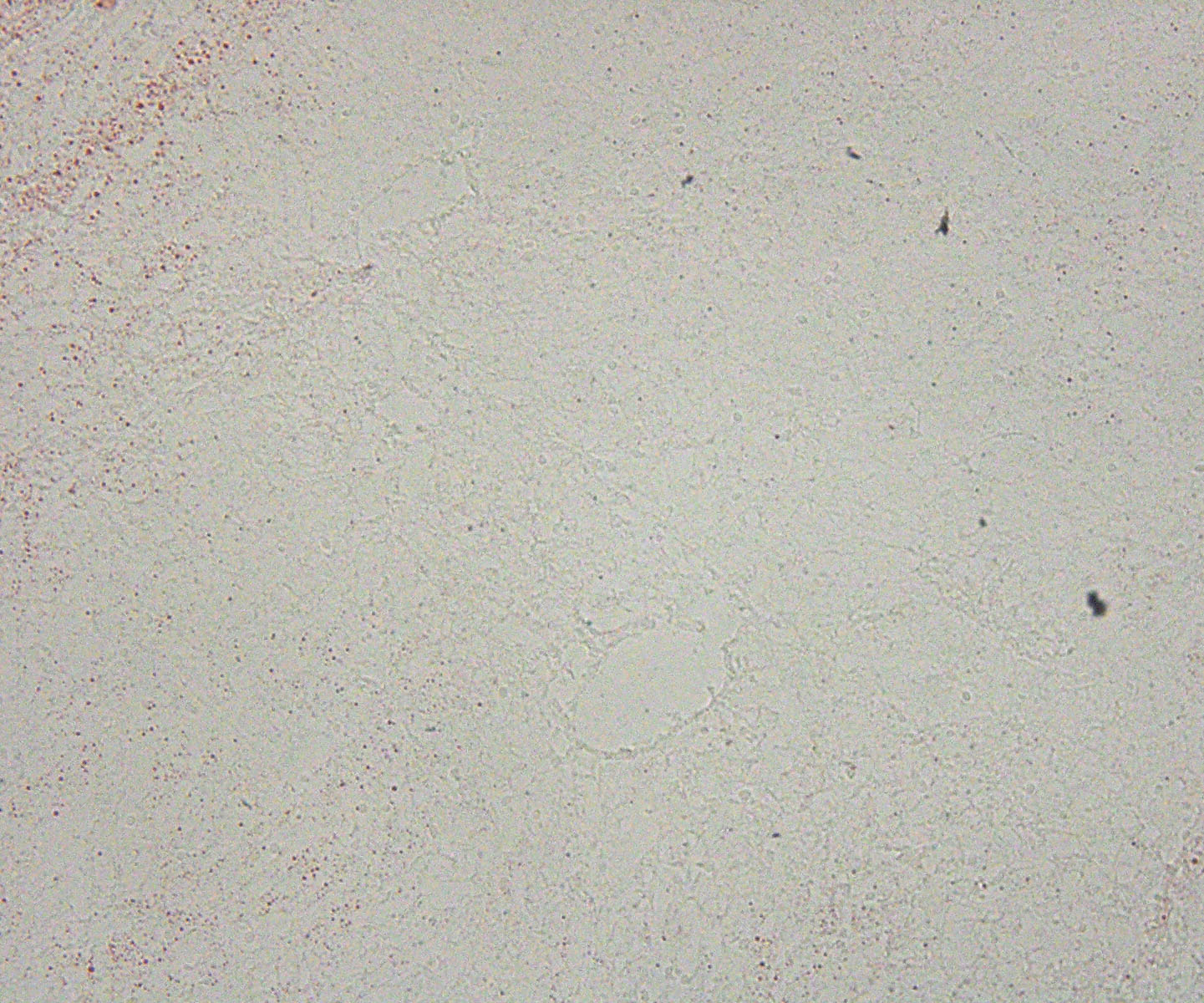

Supplement: Supplementary file 4 — Source Data for Figure 2 [file EMMM-15-e16592-s008.zip › Figure 2/Fig.2D/5.jpg]

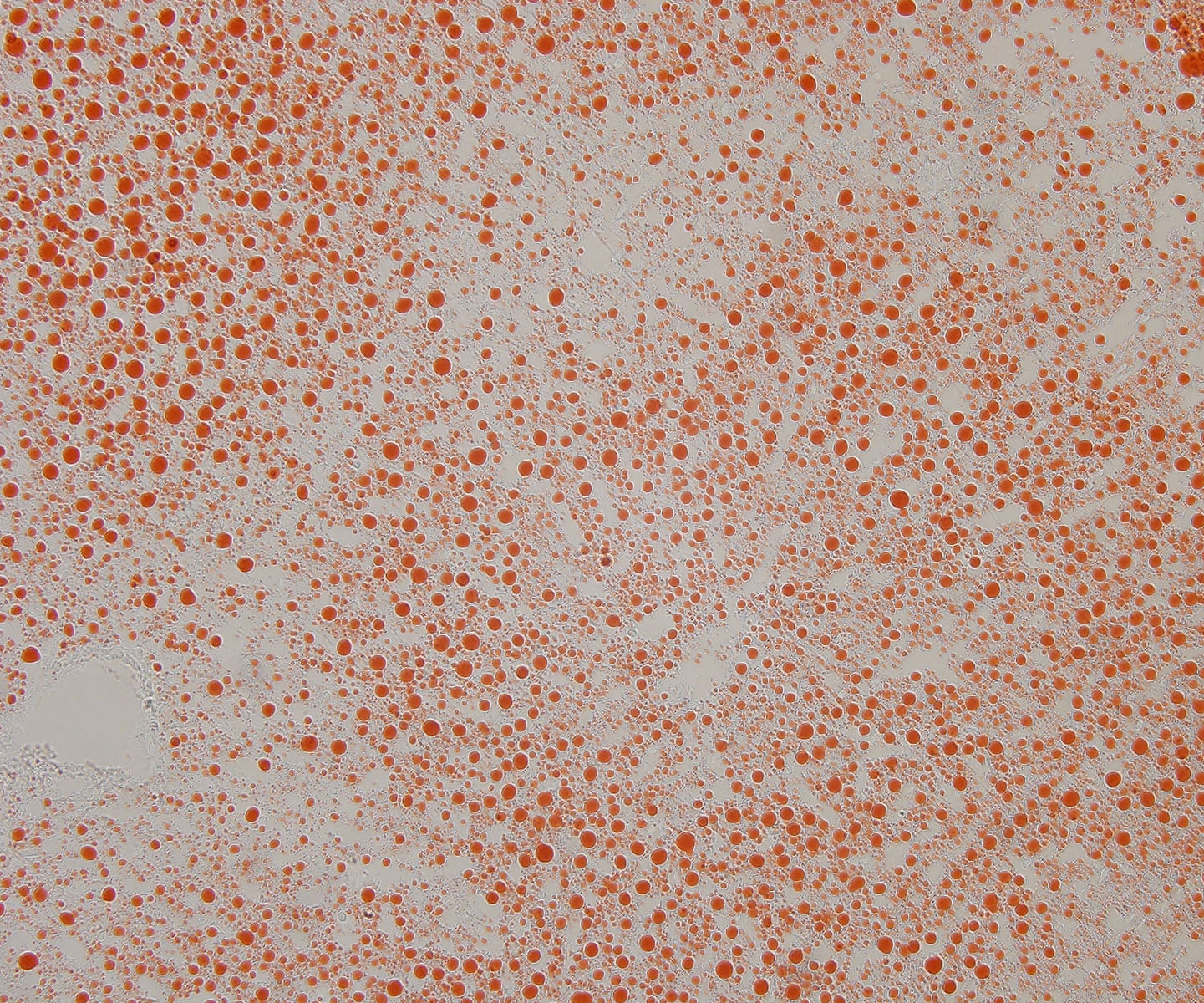

Supplement: Supplementary file 4 — Source Data for Figure 2 [file EMMM-15-e16592-s008.zip › Figure 2/Fig.2D/6.jpg]

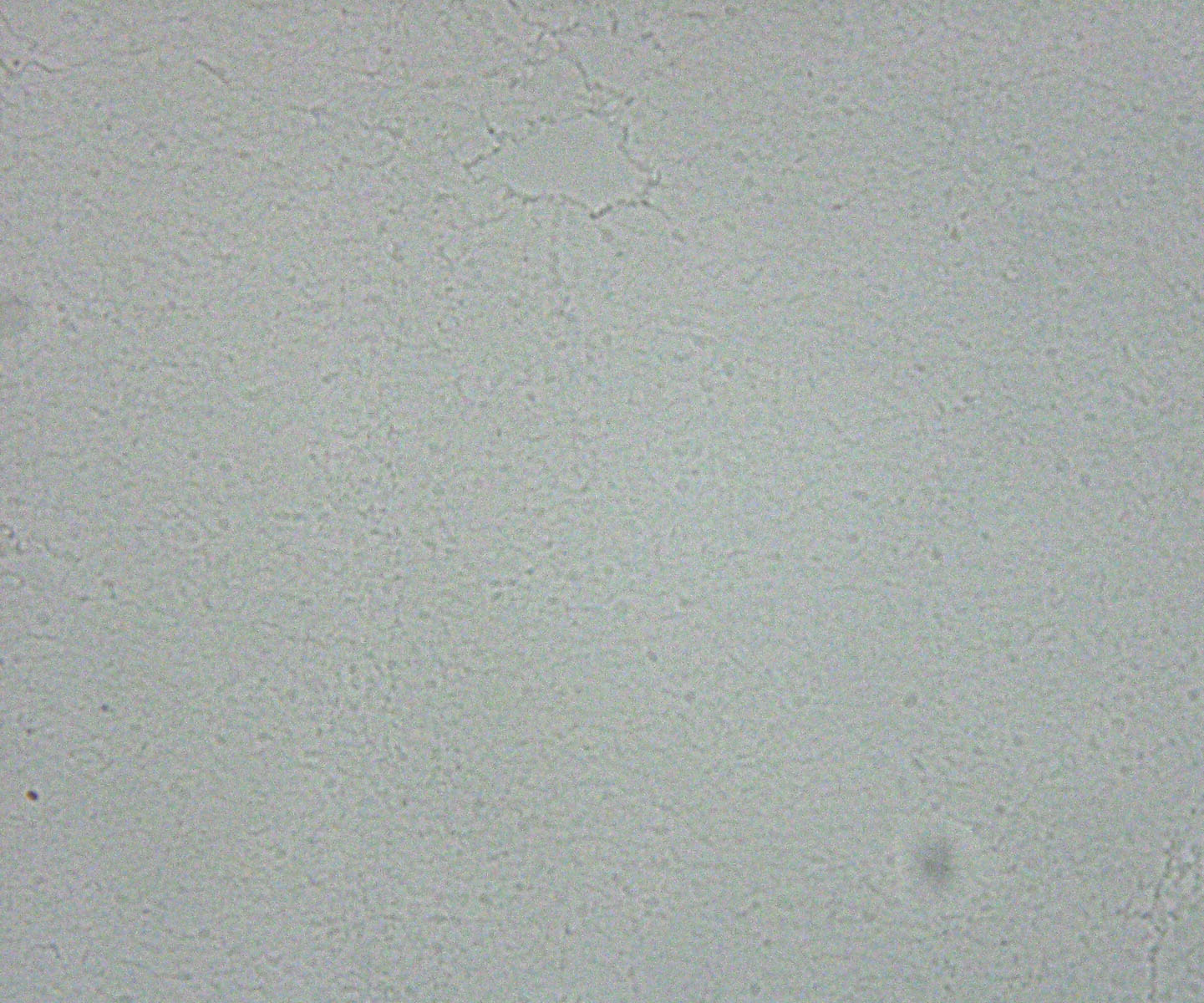

Supplement: Supplementary file 4 — Source Data for Figure 2 [file EMMM-15-e16592-s008.zip › Figure 2/Fig.2D/7.jpg]

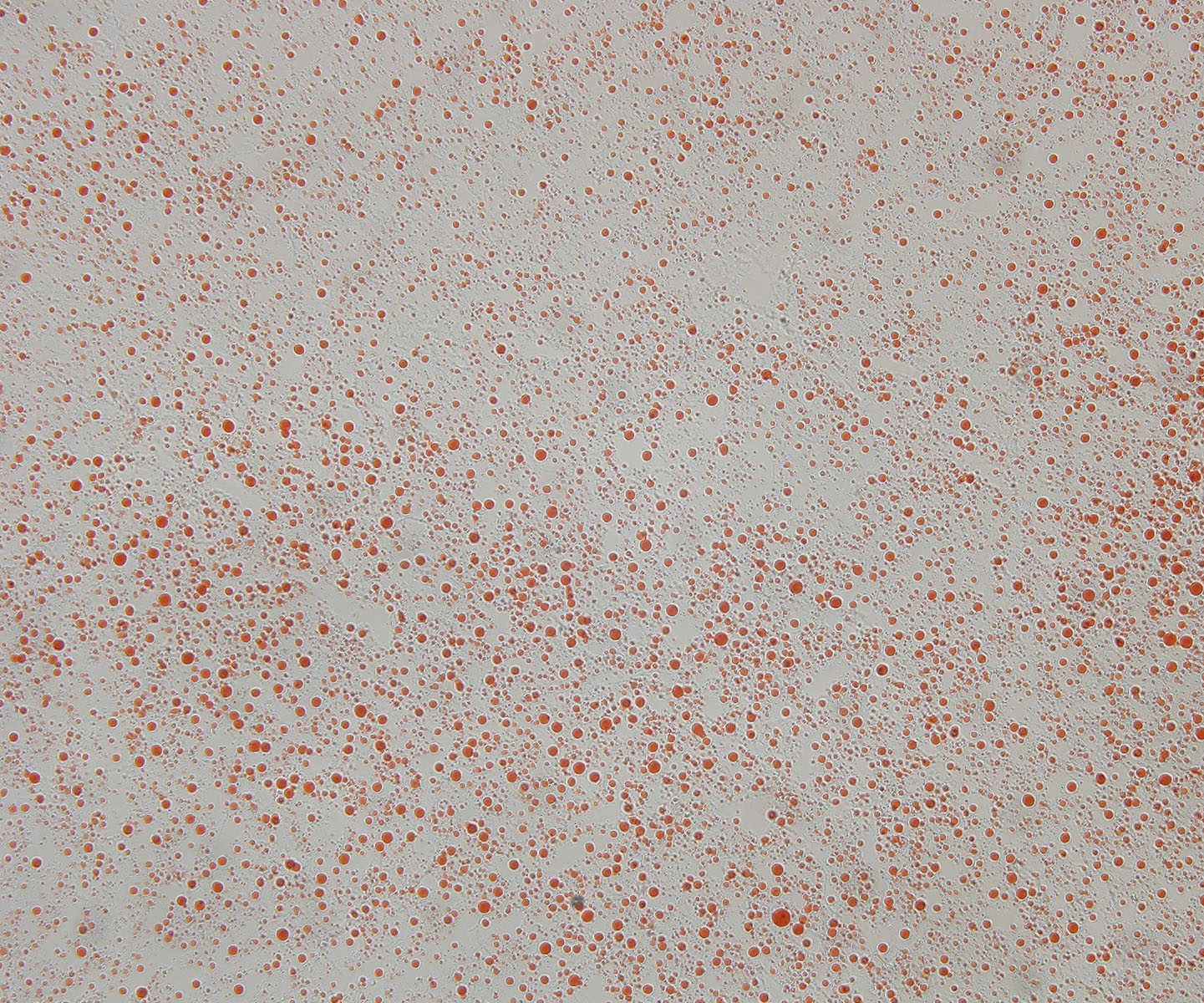

Supplement: Supplementary file 4 — Source Data for Figure 2 [file EMMM-15-e16592-s008.zip › Figure 2/Fig.2D/8.jpg]

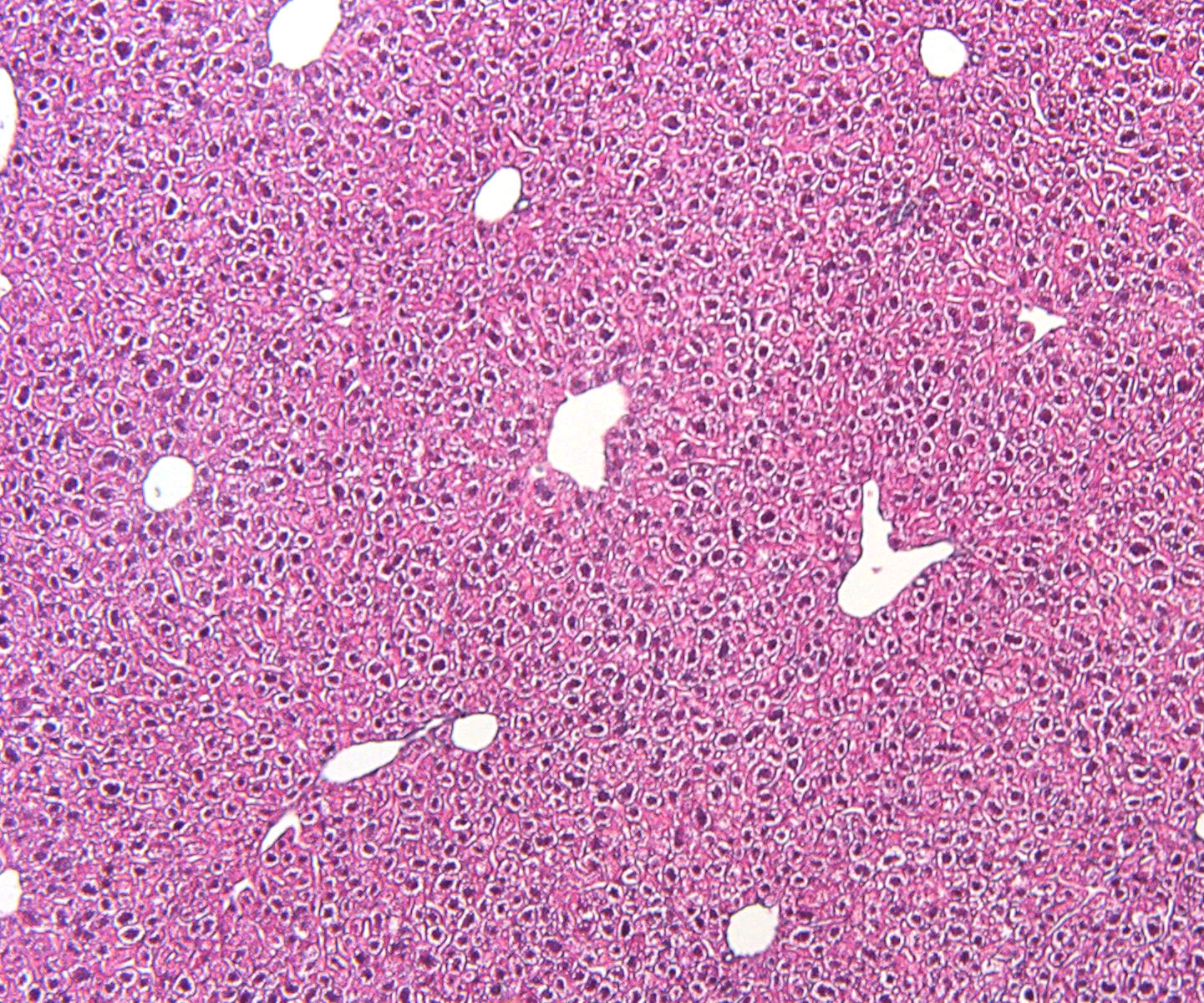

Supplement: Supplementary file 4 — Source Data for Figure 2 [file EMMM-15-e16592-s008.zip › Figure 2/Fig.2I/1.jpg]

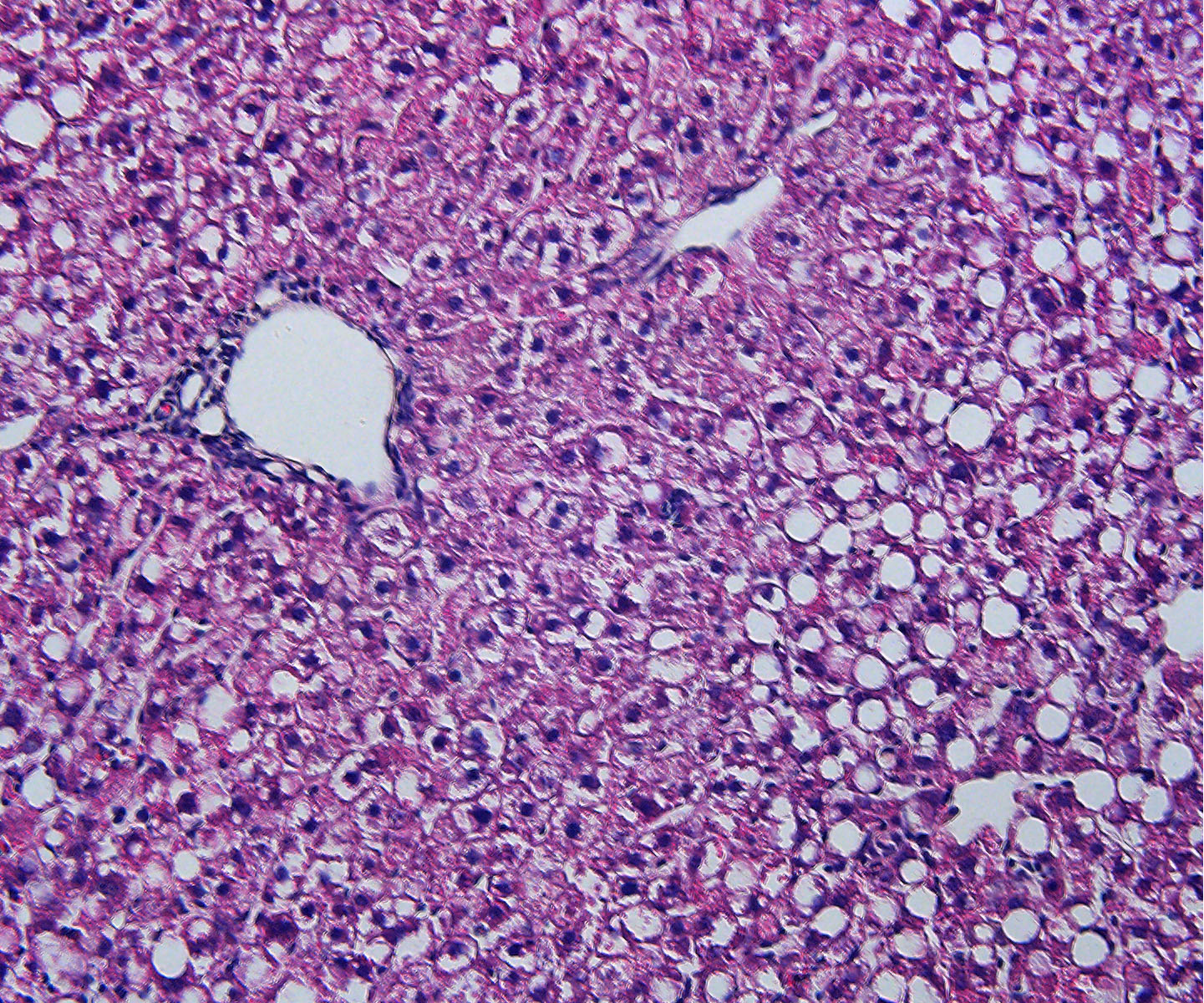

Supplement: Supplementary file 4 — Source Data for Figure 2 [file EMMM-15-e16592-s008.zip › Figure 2/Fig.2I/2.jpg]

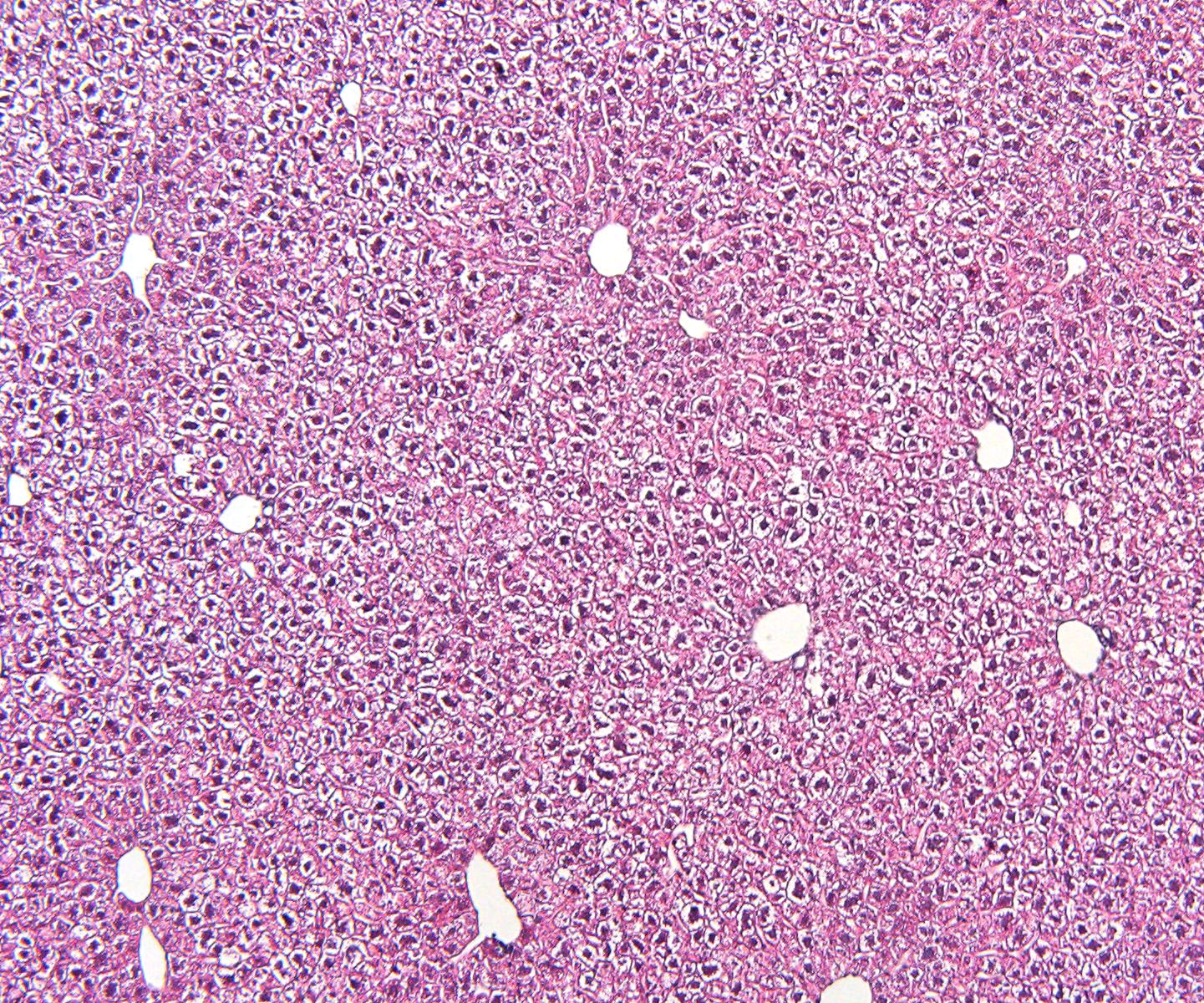

Supplement: Supplementary file 4 — Source Data for Figure 2 [file EMMM-15-e16592-s008.zip › Figure 2/Fig.2I/3.jpg]

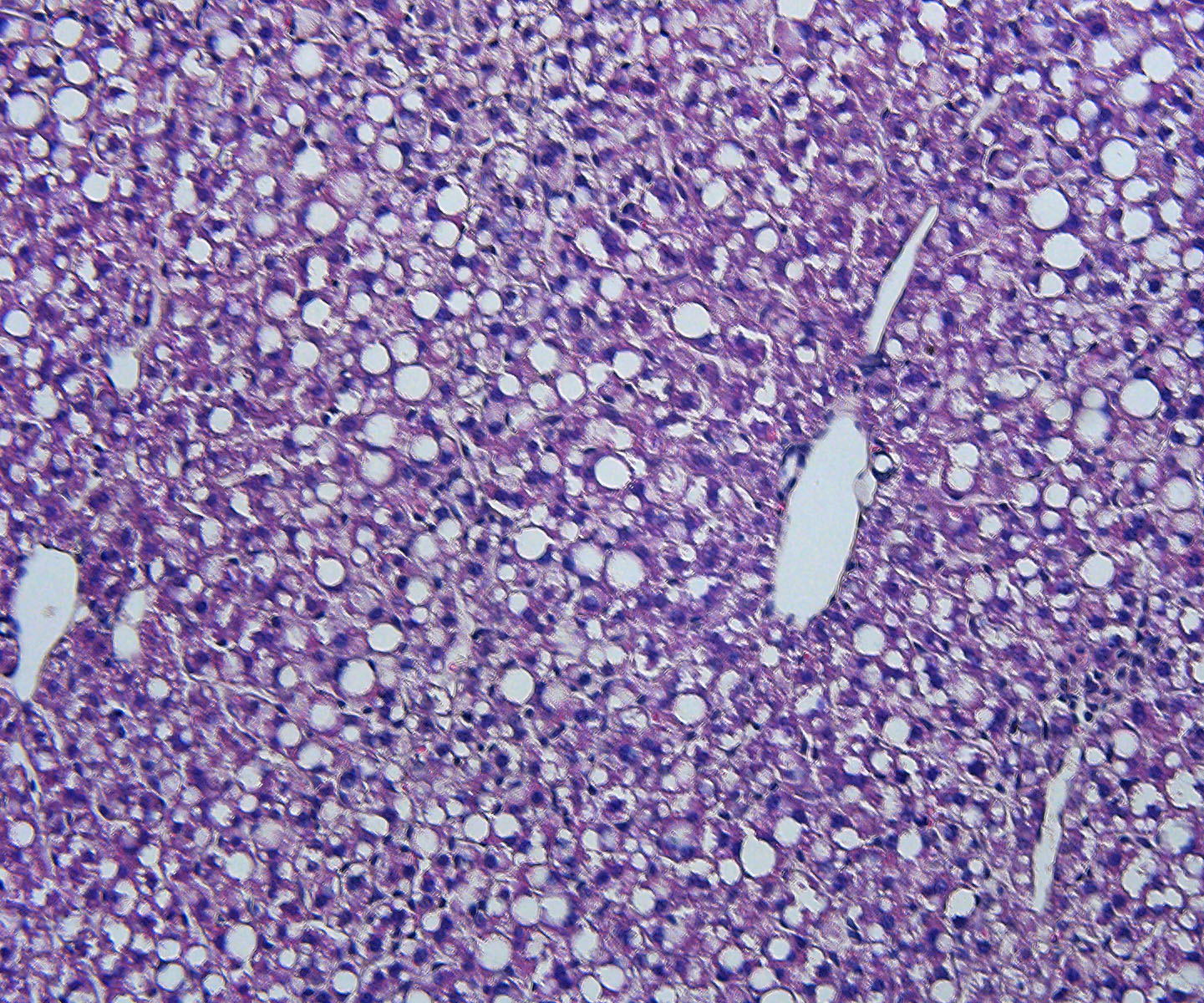

Supplement: Supplementary file 4 — Source Data for Figure 2 [file EMMM-15-e16592-s008.zip › Figure 2/Fig.2I/4.jpg]

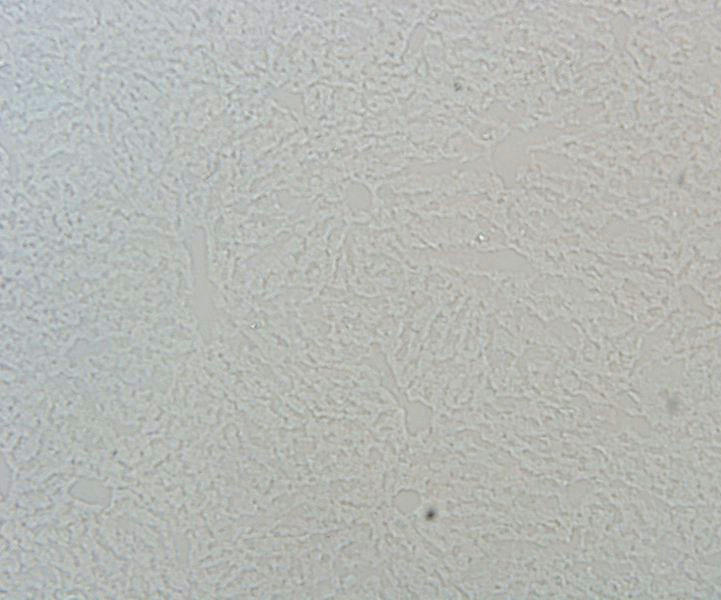

Supplement: Supplementary file 4 — Source Data for Figure 2 [file EMMM-15-e16592-s008.zip › Figure 2/Fig.2I/5.jpg]

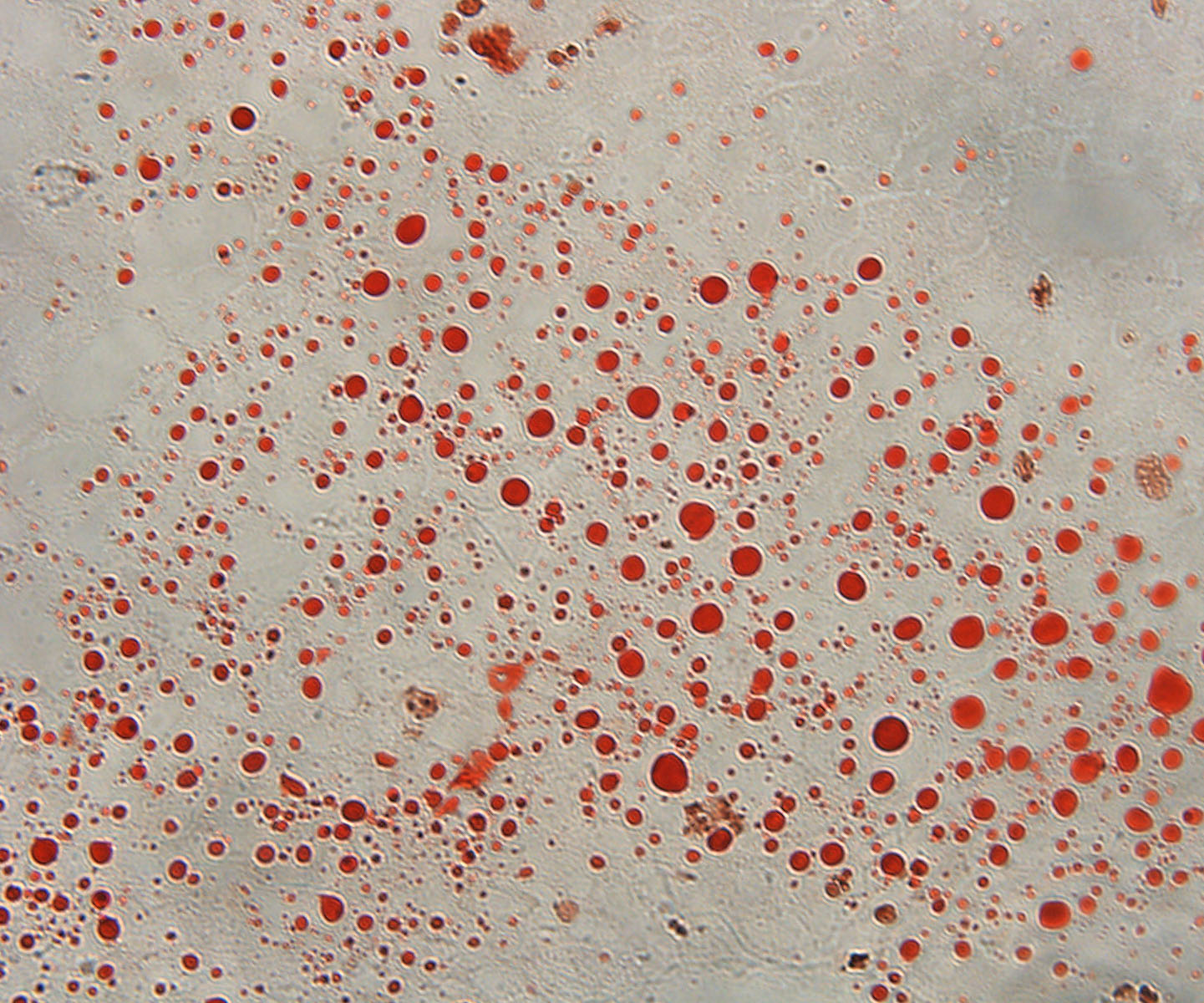

Supplement: Supplementary file 4 — Source Data for Figure 2 [file EMMM-15-e16592-s008.zip › Figure 2/Fig.2I/6.jpg]

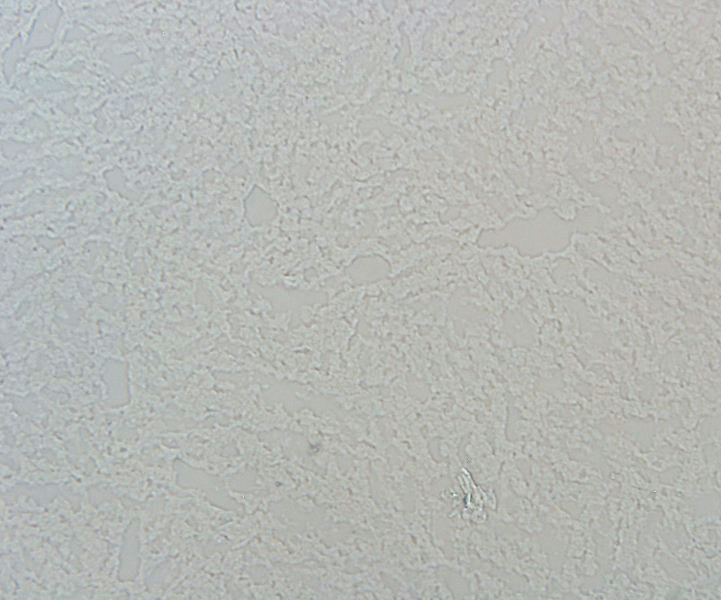

Supplement: Supplementary file 4 — Source Data for Figure 2 [file EMMM-15-e16592-s008.zip › Figure 2/Fig.2I/7.jpg]

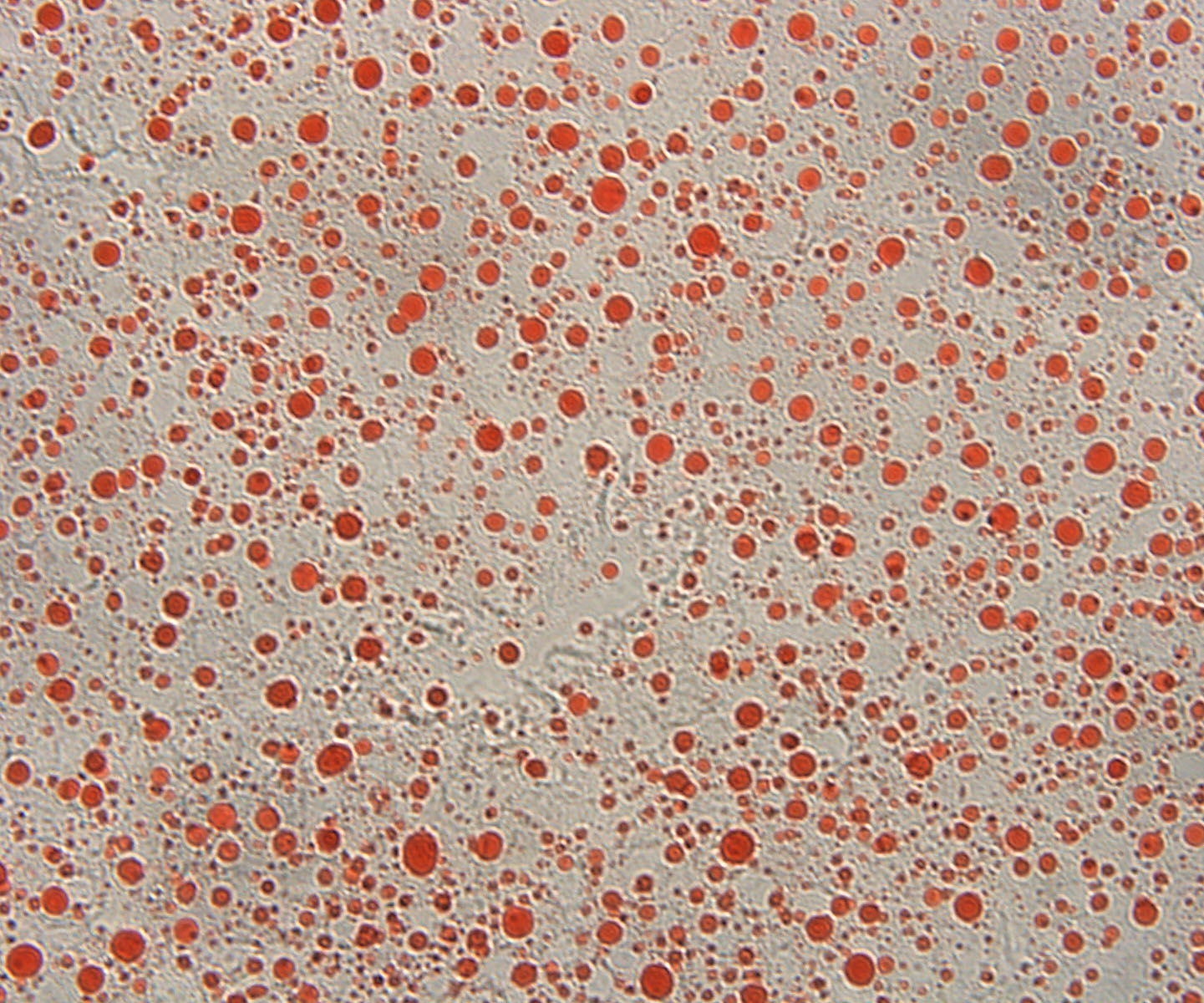

Supplement: Supplementary file 4 — Source Data for Figure 2 [file EMMM-15-e16592-s008.zip › Figure 2/Fig.2I/8.jpg]

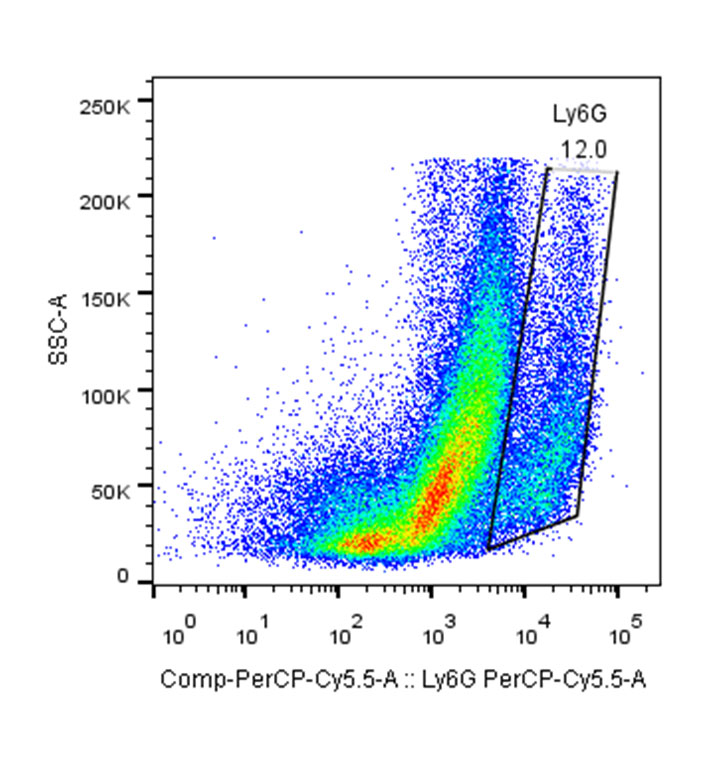

Supplement: Supplementary file 5 — Source Data for Figure 3 [file EMMM-15-e16592-s003.zip › Figure 3/Fig.3A/1.jpg]

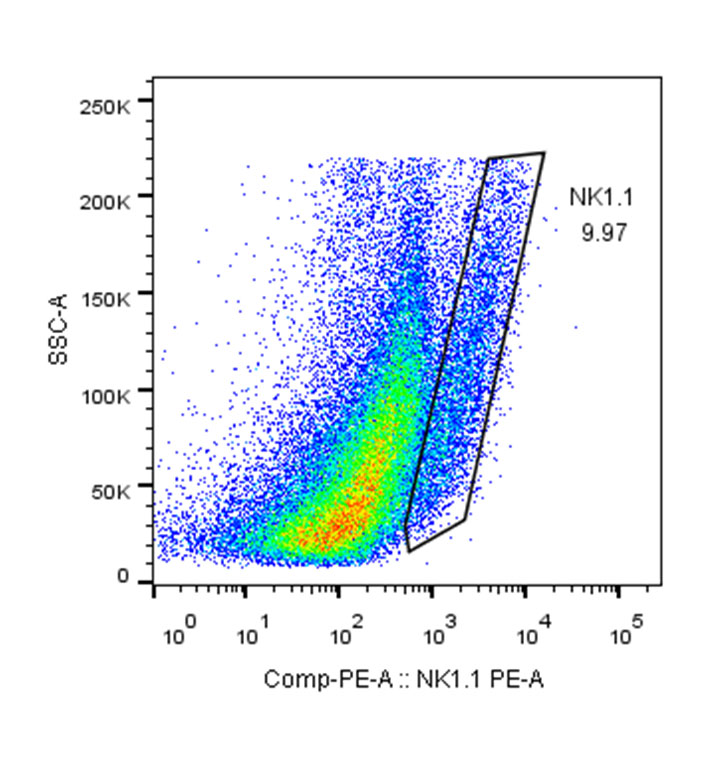

Supplement: Supplementary file 5 — Source Data for Figure 3 [file EMMM-15-e16592-s003.zip › Figure 3/Fig.3A/10.jpg]

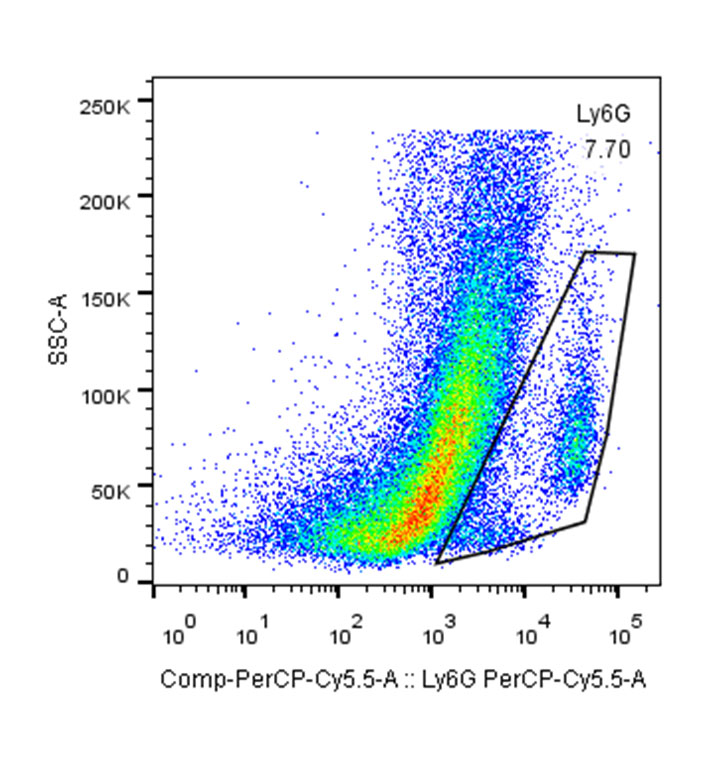

Supplement: Supplementary file 5 — Source Data for Figure 3 [file EMMM-15-e16592-s003.zip › Figure 3/Fig.3A/2.jpg]

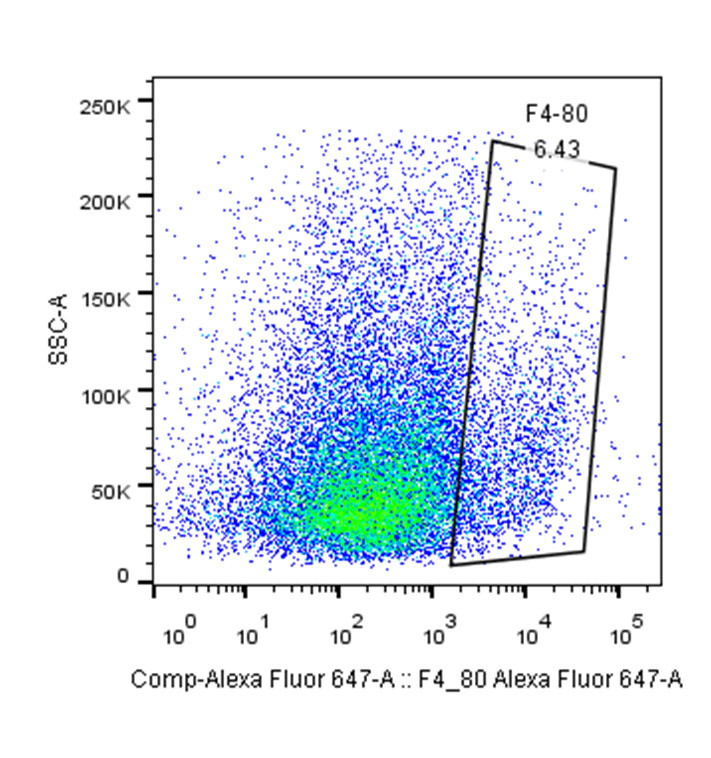

Supplement: Supplementary file 5 — Source Data for Figure 3 [file EMMM-15-e16592-s003.zip › Figure 3/Fig.3A/3.jpg]

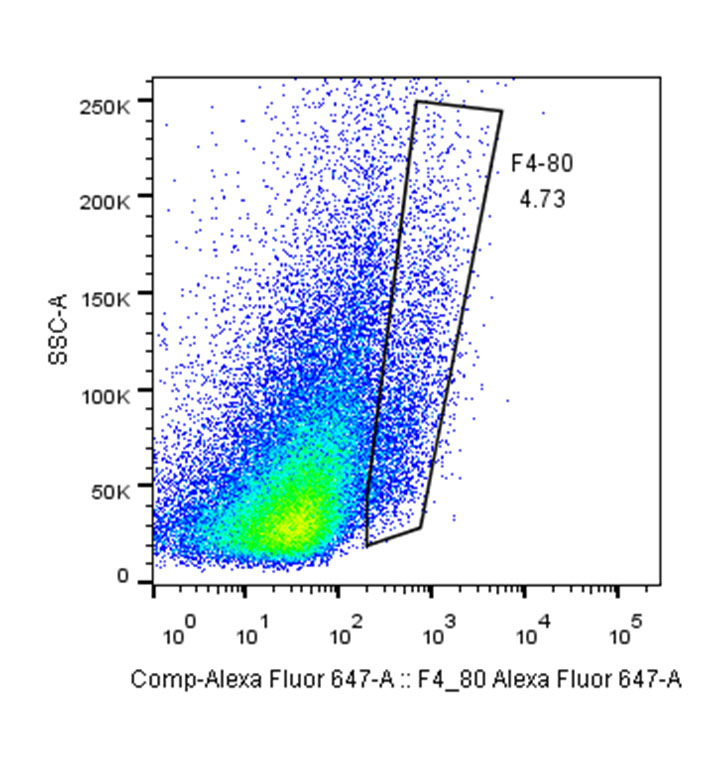

Supplement: Supplementary file 5 — Source Data for Figure 3 [file EMMM-15-e16592-s003.zip › Figure 3/Fig.3A/4.jpg]

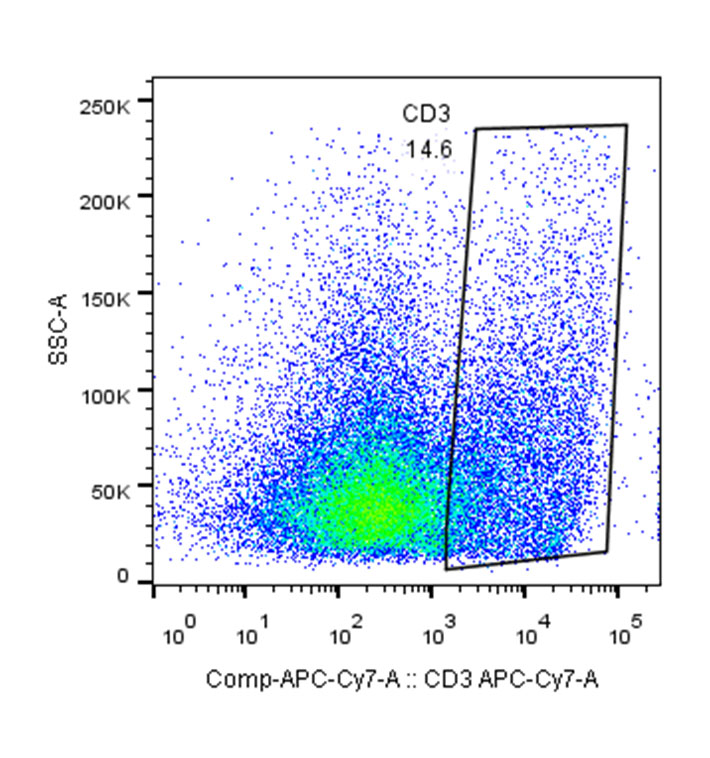

Supplement: Supplementary file 5 — Source Data for Figure 3 [file EMMM-15-e16592-s003.zip › Figure 3/Fig.3A/5.jpg]

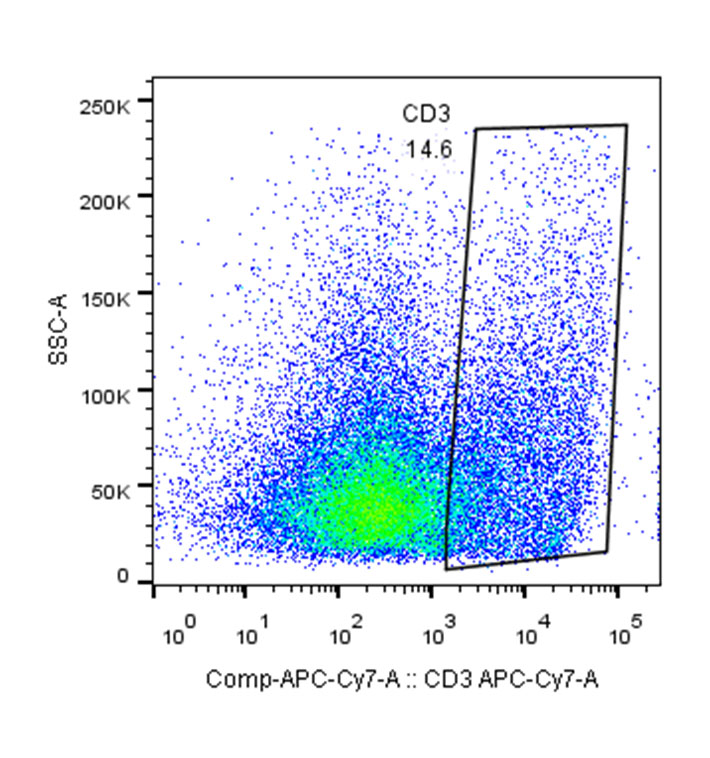

Supplement: Supplementary file 5 — Source Data for Figure 3 [file EMMM-15-e16592-s003.zip › Figure 3/Fig.3A/6.jpg]

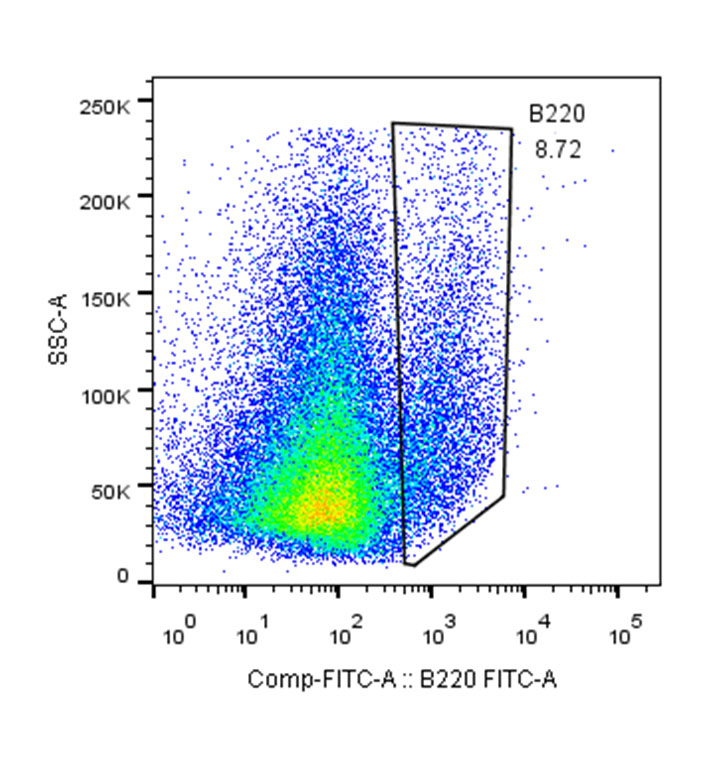

Supplement: Supplementary file 5 — Source Data for Figure 3 [file EMMM-15-e16592-s003.zip › Figure 3/Fig.3A/7.jpg]

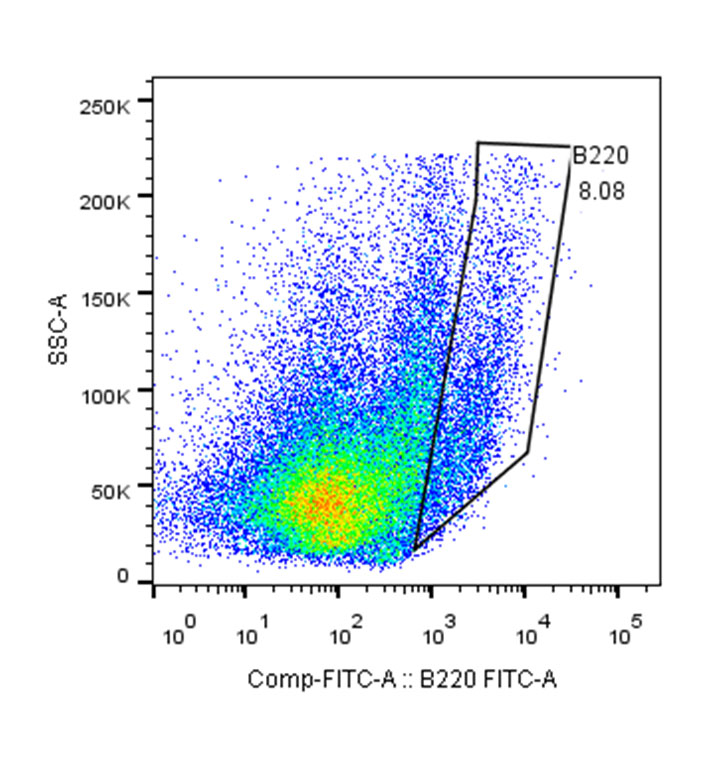

Supplement: Supplementary file 5 — Source Data for Figure 3 [file EMMM-15-e16592-s003.zip › Figure 3/Fig.3A/8.jpg]

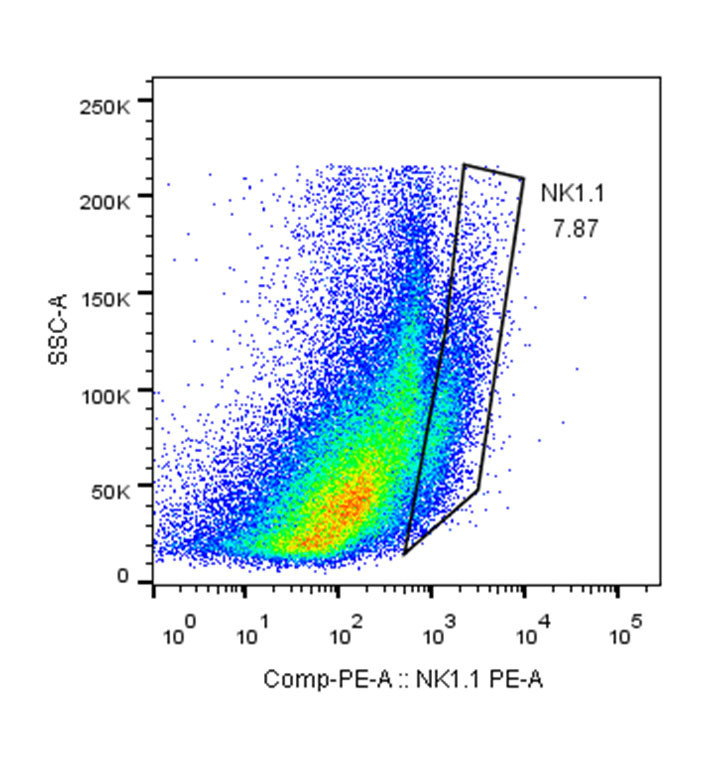

Supplement: Supplementary file 5 — Source Data for Figure 3 [file EMMM-15-e16592-s003.zip › Figure 3/Fig.3A/9.jpg]

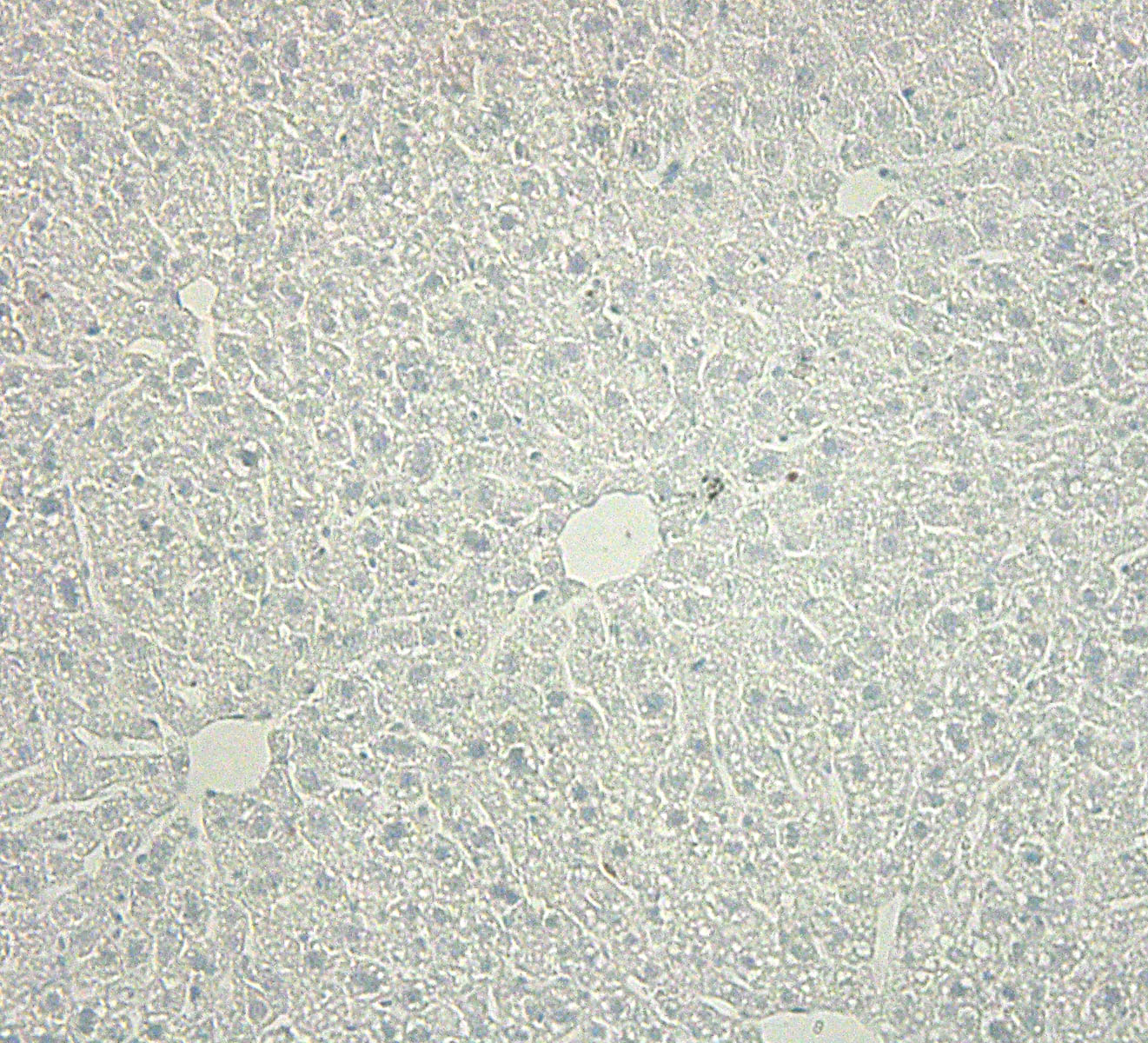

Supplement: Supplementary file 5 — Source Data for Figure 3 [file EMMM-15-e16592-s003.zip › Figure 3/Fig.3B/1.jpg]

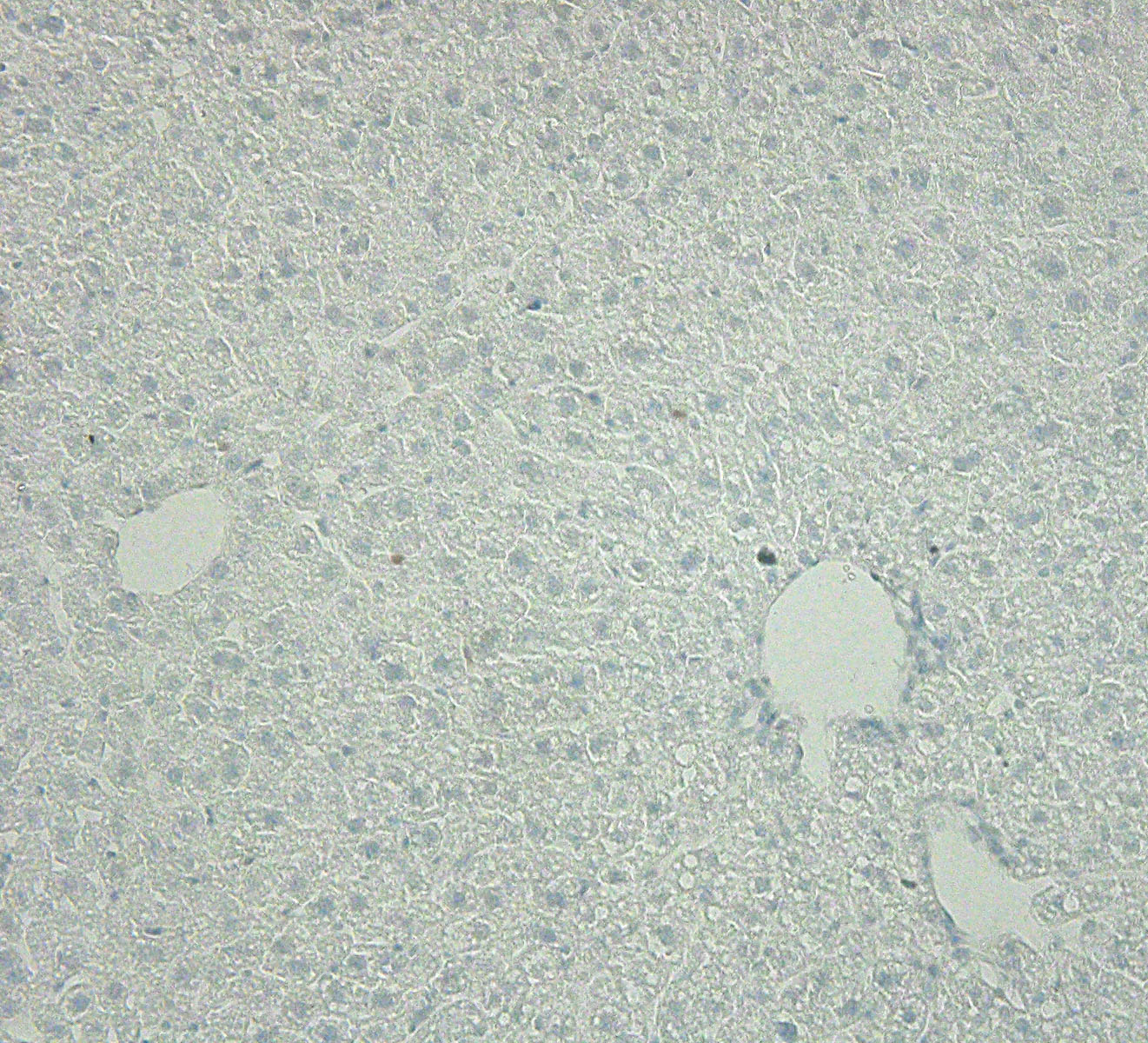

Supplement: Supplementary file 5 — Source Data for Figure 3 [file EMMM-15-e16592-s003.zip › Figure 3/Fig.3B/2.jpg]

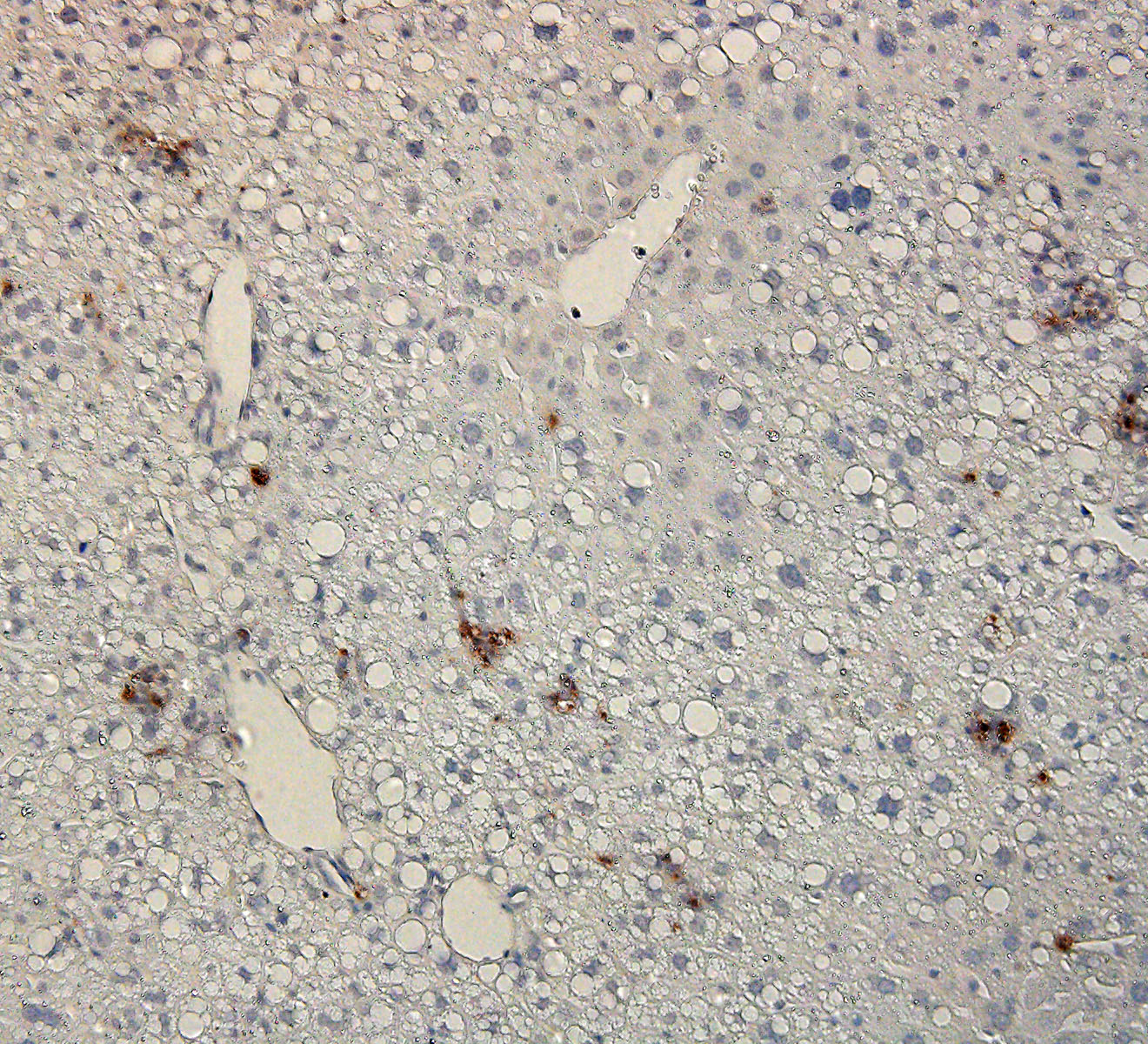

Supplement: Supplementary file 5 — Source Data for Figure 3 [file EMMM-15-e16592-s003.zip › Figure 3/Fig.3B/3.jpg]

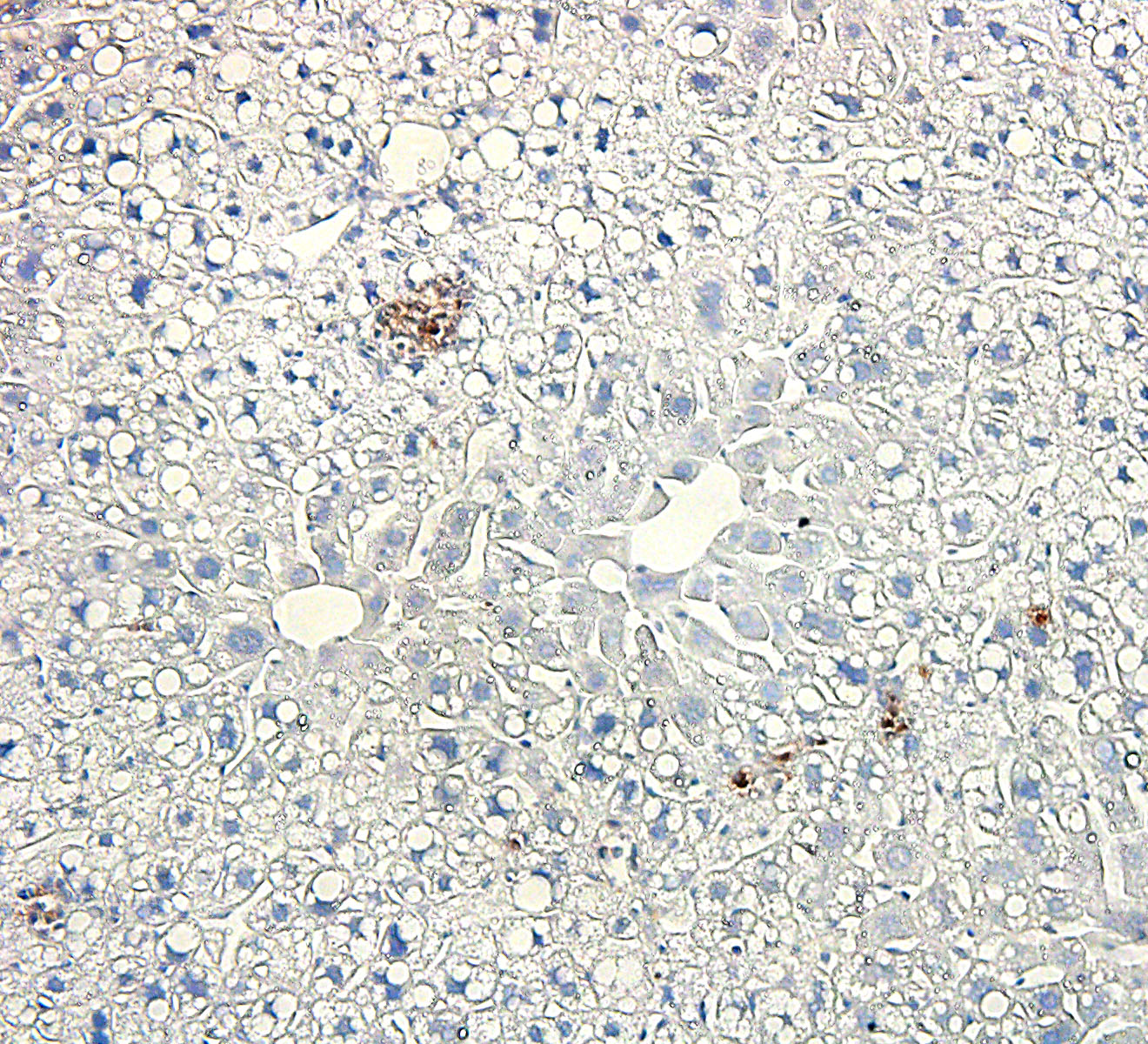

Supplement: Supplementary file 5 — Source Data for Figure 3 [file EMMM-15-e16592-s003.zip › Figure 3/Fig.3B/4.jpg]

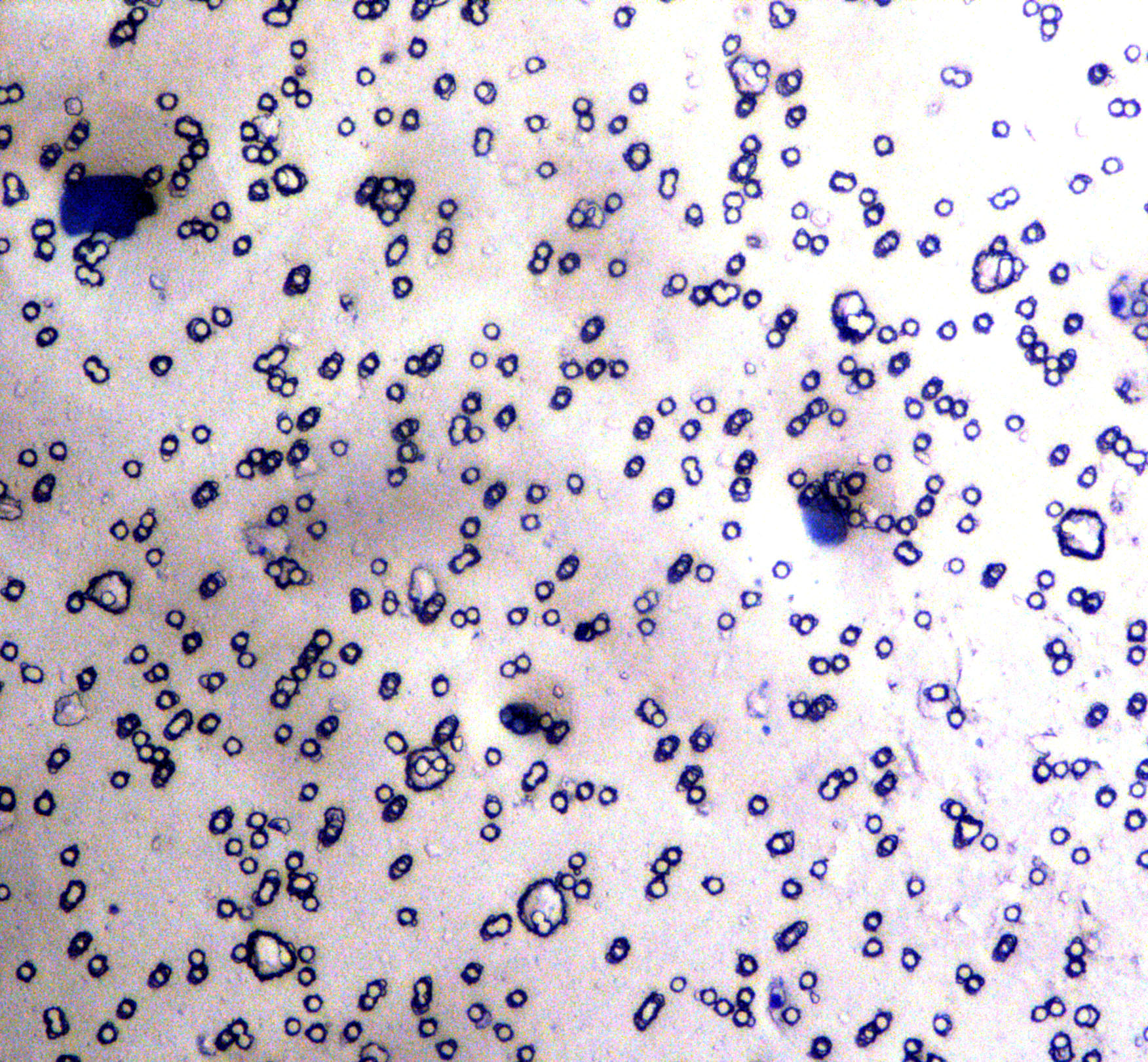

Supplement: Supplementary file 5 — Source Data for Figure 3 [file EMMM-15-e16592-s003.zip › Figure 3/Fig.3D/WT CKO hepatocyte/1.jpg]

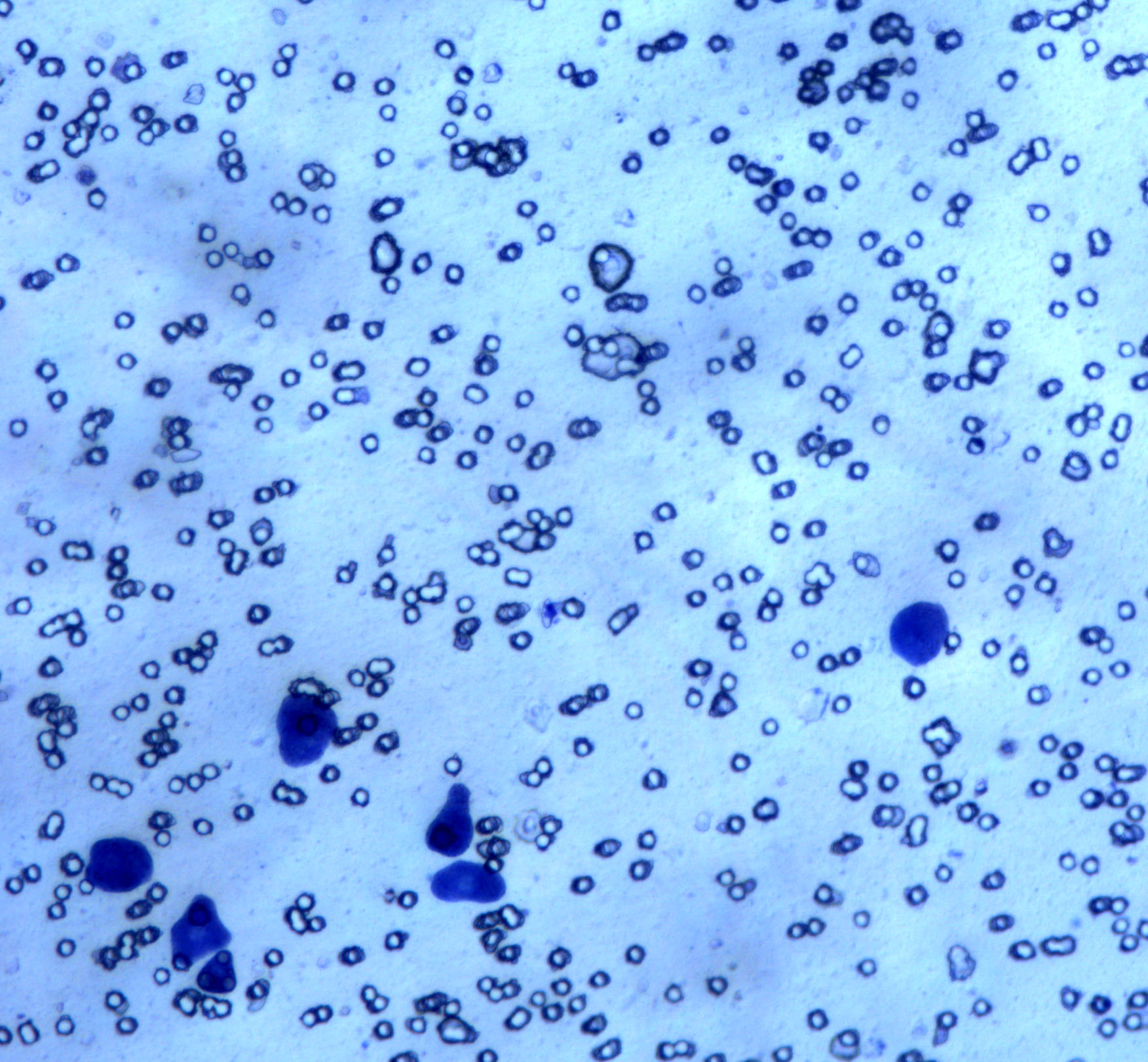

Supplement: Supplementary file 5 — Source Data for Figure 3 [file EMMM-15-e16592-s003.zip › Figure 3/Fig.3D/WT CKO hepatocyte/2.jpg]

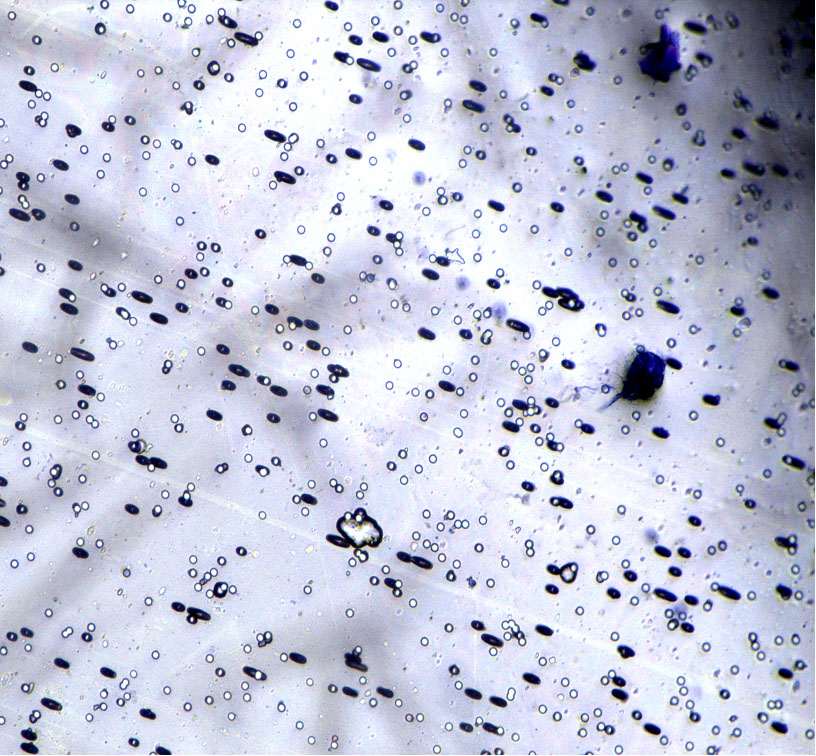

Supplement: Supplementary file 5 — Source Data for Figure 3 [file EMMM-15-e16592-s003.zip › Figure 3/Fig.3D/WT CKO hepatocyte/3.jpg]

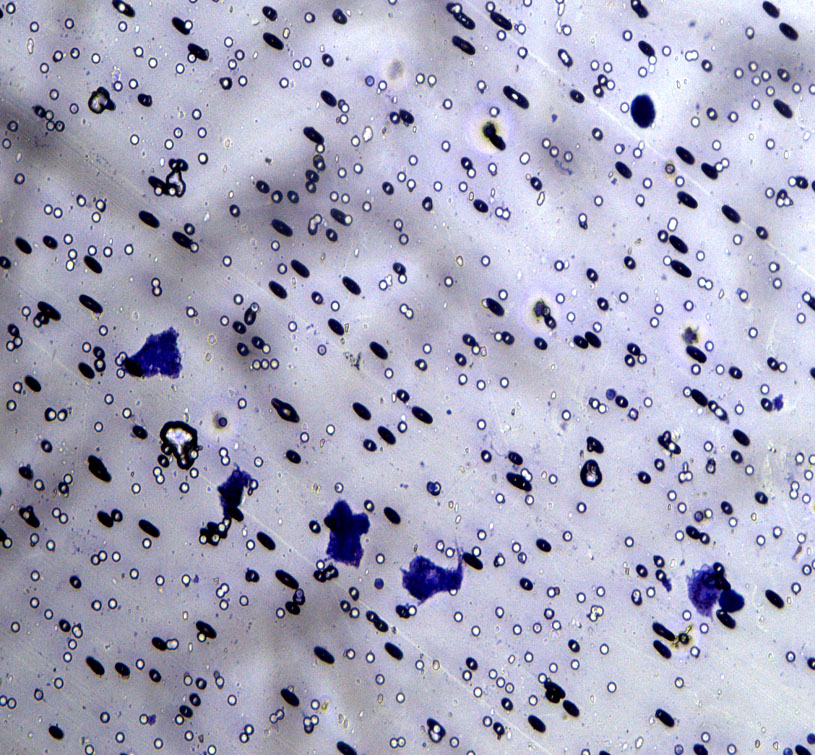

Supplement: Supplementary file 5 — Source Data for Figure 3 [file EMMM-15-e16592-s003.zip › Figure 3/Fig.3D/WT CKO hepatocyte/4.jpg]

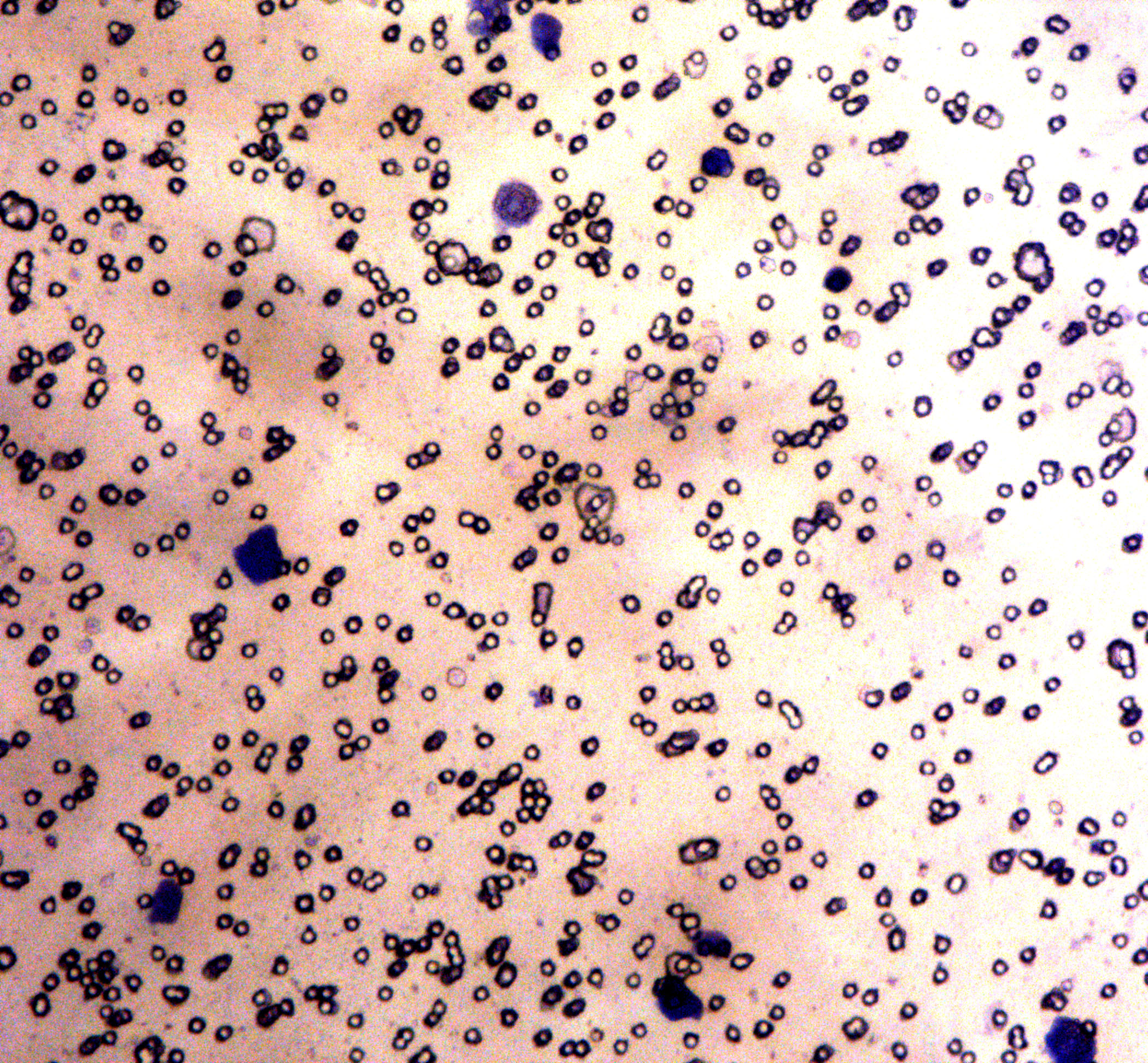

Supplement: Supplementary file 6 — Source Data for Figure 4 [file EMMM-15-e16592-s005.zip › Figure 4/Fig.4G/WT CKO hepatocytes rCXCL14/1.jpg]

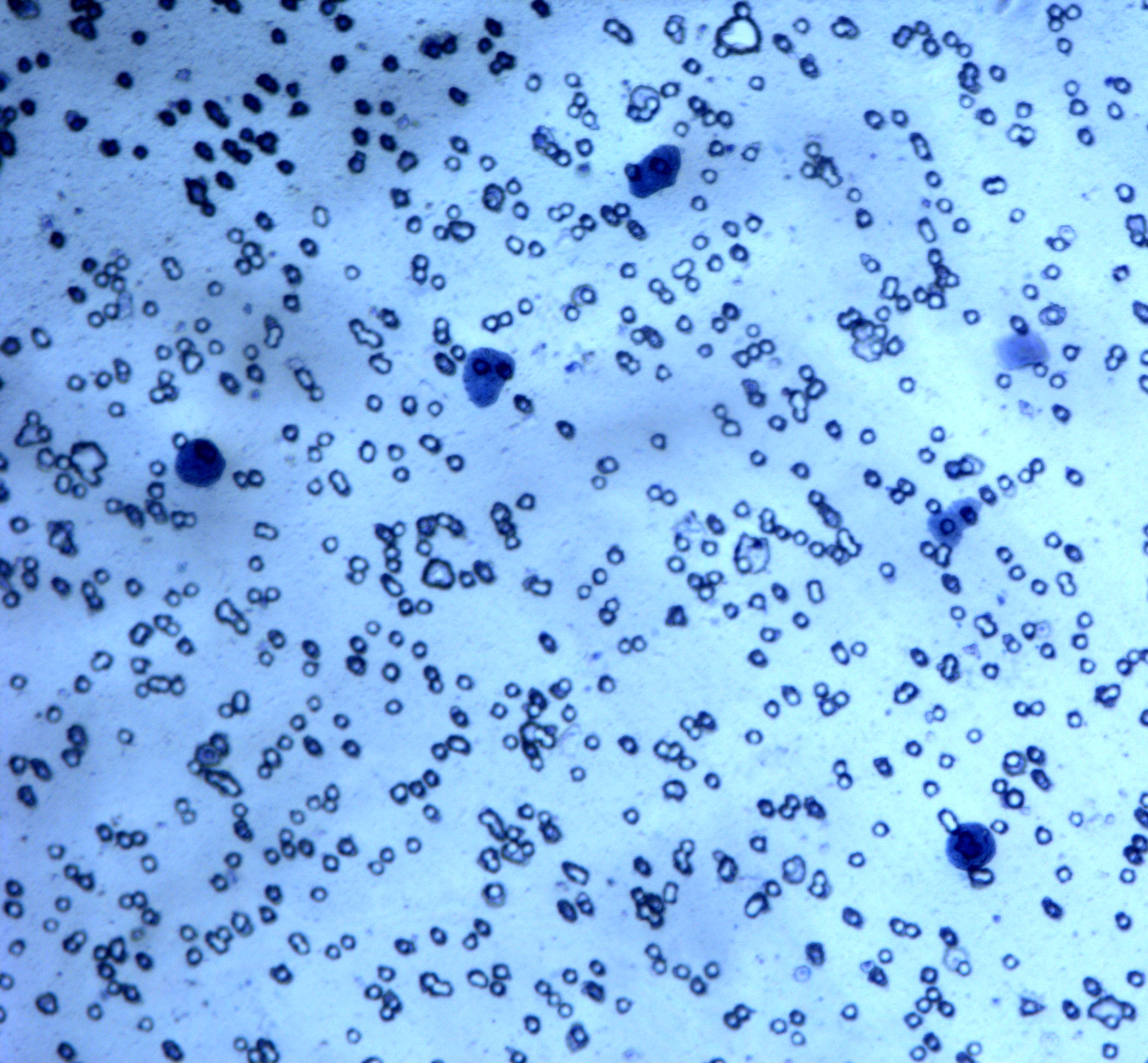

Supplement: Supplementary file 6 — Source Data for Figure 4 [file EMMM-15-e16592-s005.zip › Figure 4/Fig.4G/WT CKO hepatocytes rCXCL14/2.jpg]

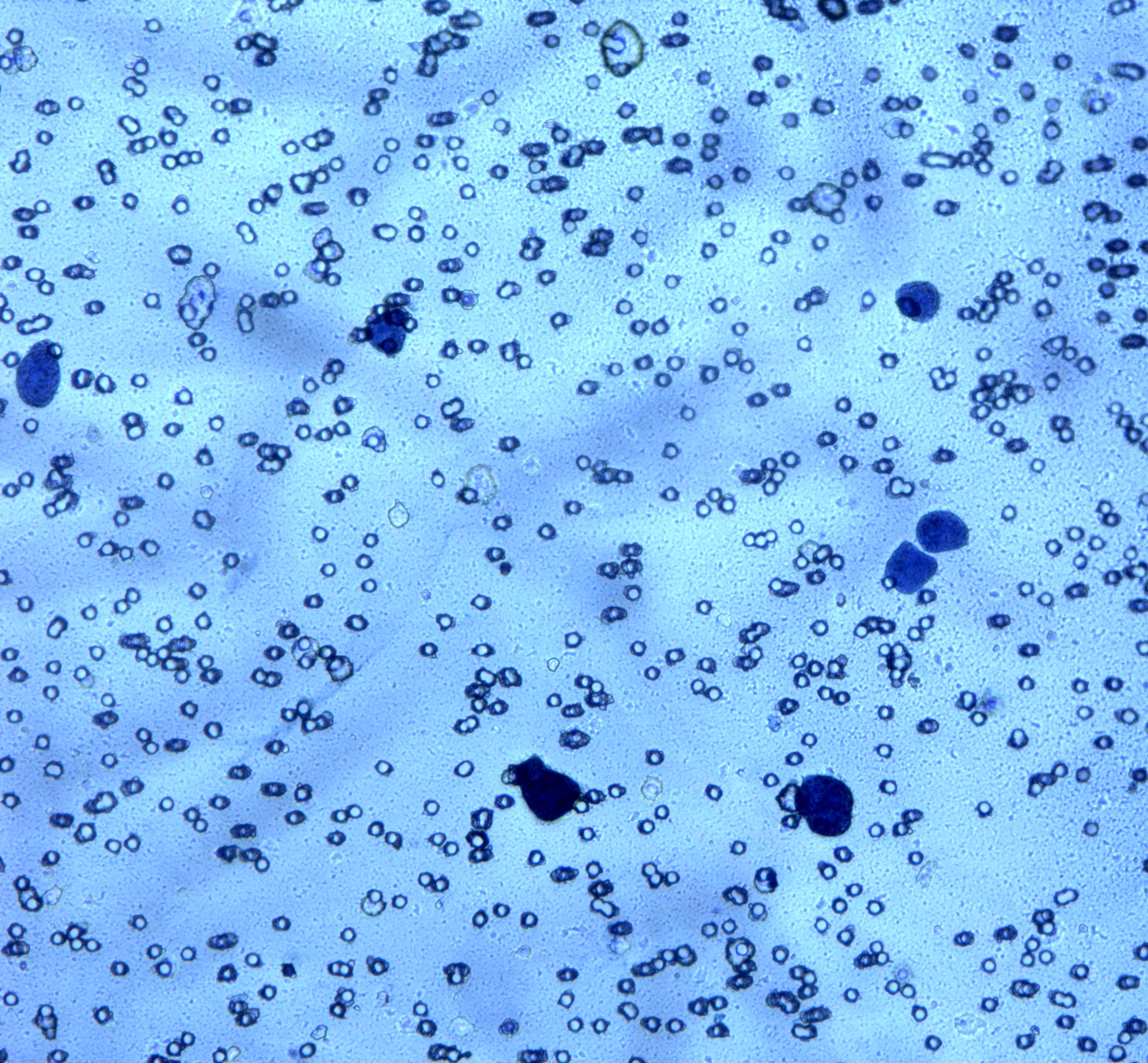

Supplement: Supplementary file 6 — Source Data for Figure 4 [file EMMM-15-e16592-s005.zip › Figure 4/Fig.4G/WT CKO hepatocytes rCXCL14/3.jpg]

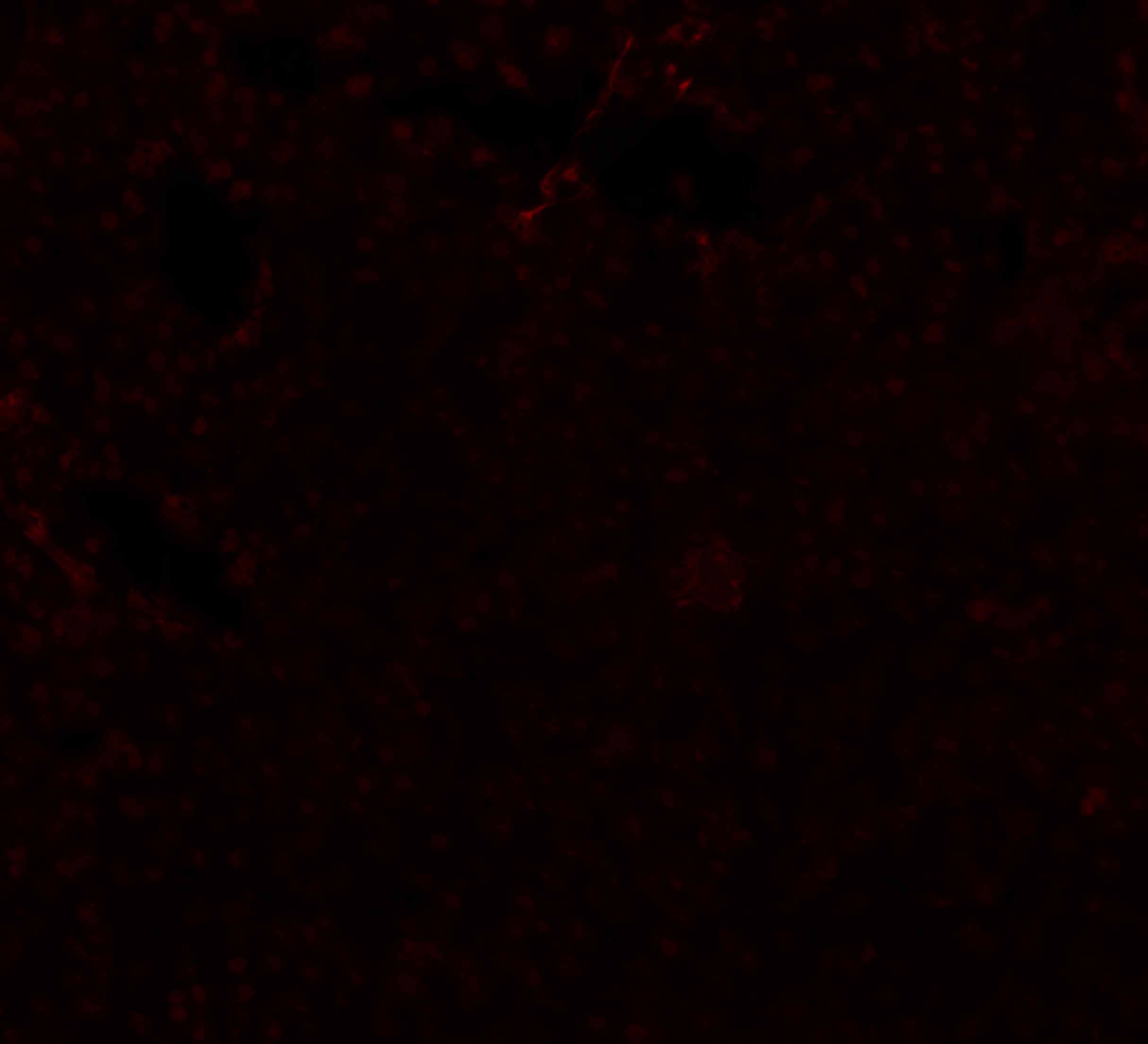

Supplement: Supplementary file 7 — Source Data for Figure 5 [file EMMM-15-e16592-s010.zip › Figure 5/Fig.5E/DHE/1.jpg]

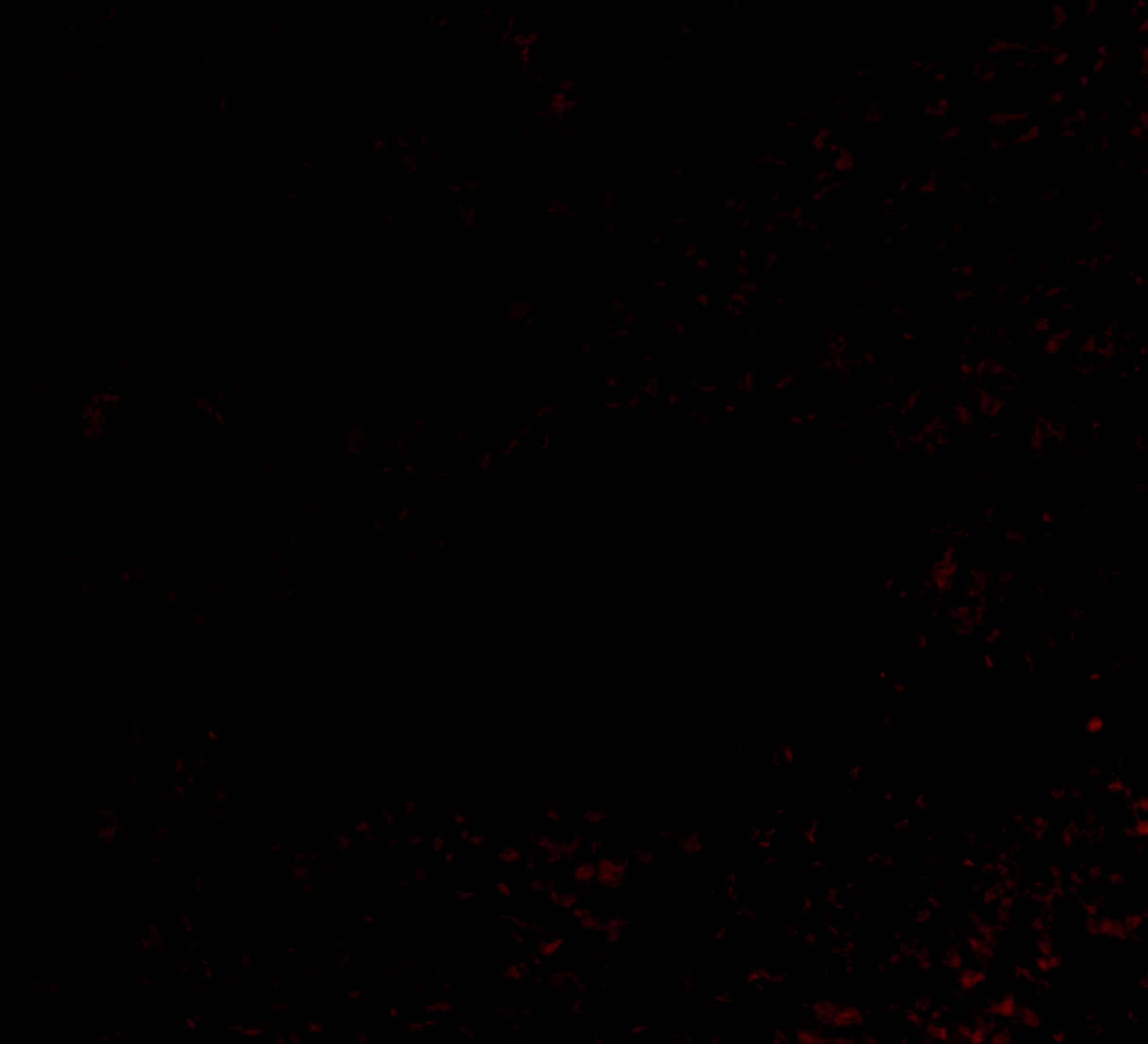

Supplement: Supplementary file 7 — Source Data for Figure 5 [file EMMM-15-e16592-s010.zip › Figure 5/Fig.5E/DHE/2.jpg]

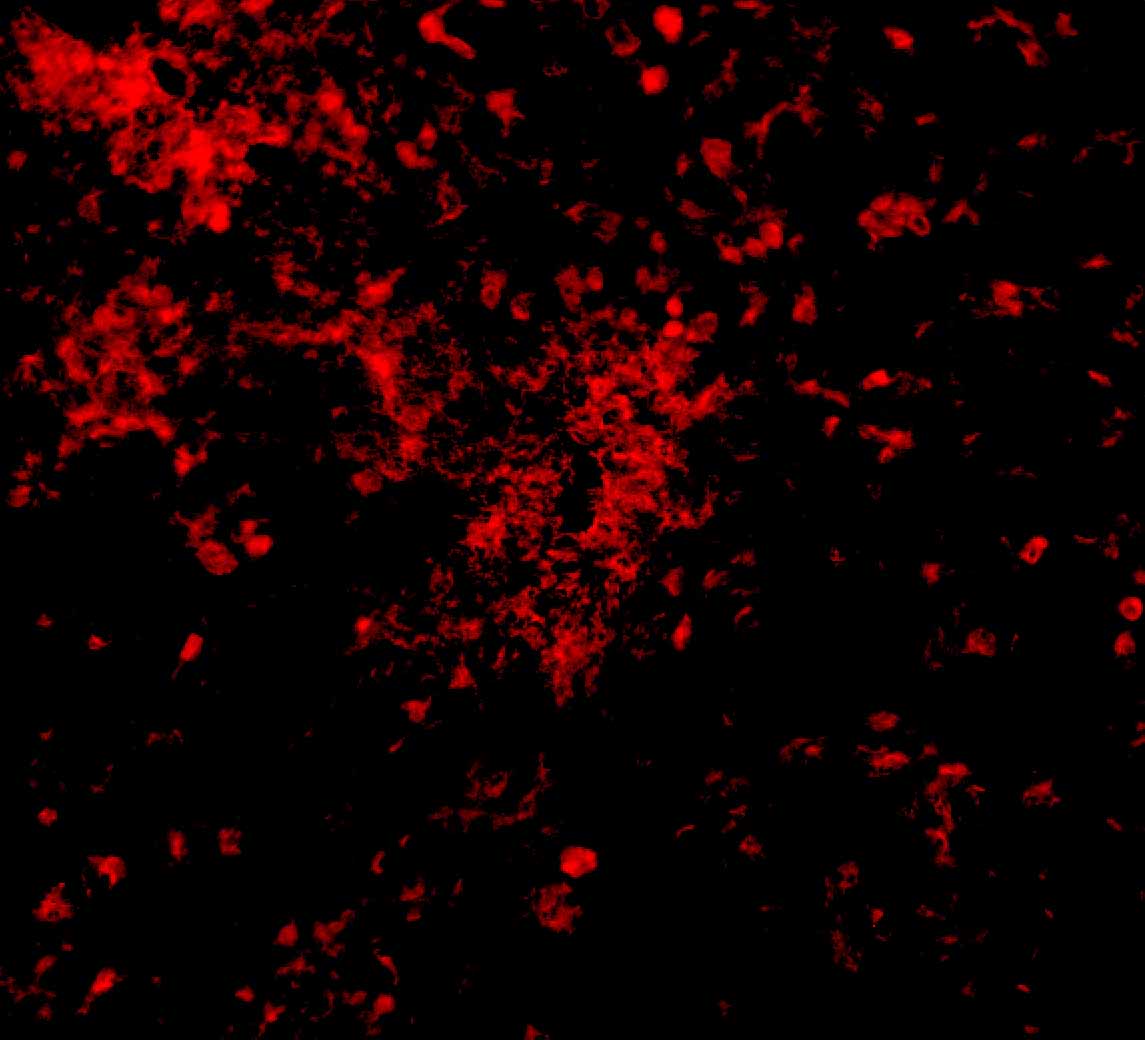

Supplement: Supplementary file 7 — Source Data for Figure 5 [file EMMM-15-e16592-s010.zip › Figure 5/Fig.5E/DHE/3.jpg]

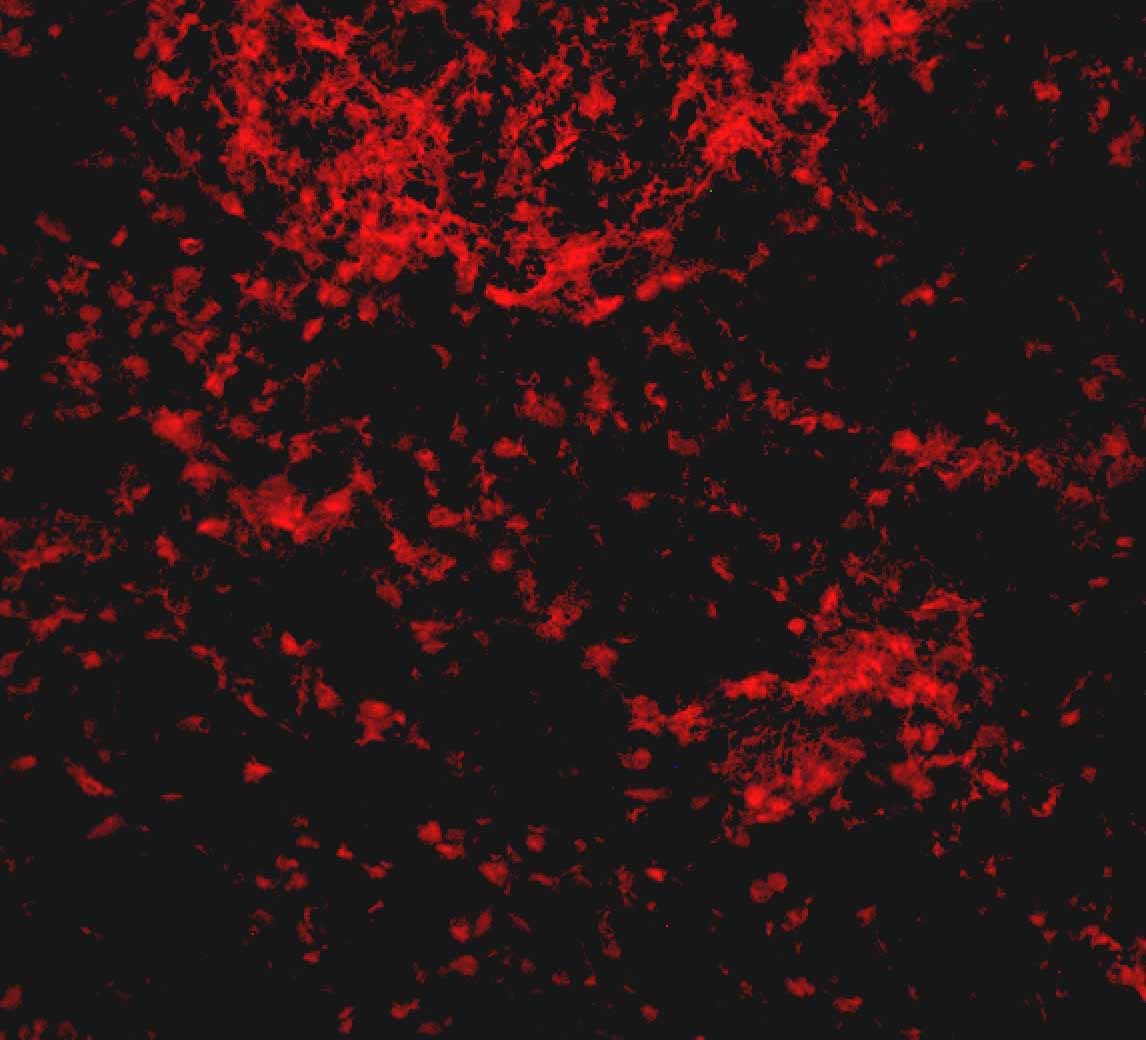

Supplement: Supplementary file 7 — Source Data for Figure 5 [file EMMM-15-e16592-s010.zip › Figure 5/Fig.5E/DHE/4.jpg]

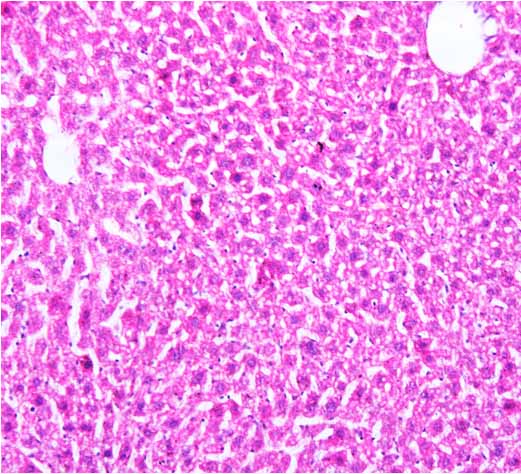

Supplement: Supplementary file 7 — Source Data for Figure 5 [file EMMM-15-e16592-s010.zip › Figure 5/Fig.5E/HE/1.jpg]

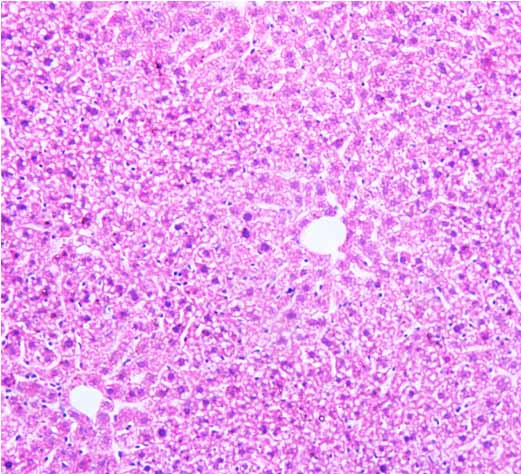

Supplement: Supplementary file 7 — Source Data for Figure 5 [file EMMM-15-e16592-s010.zip › Figure 5/Fig.5E/HE/2.jpg]

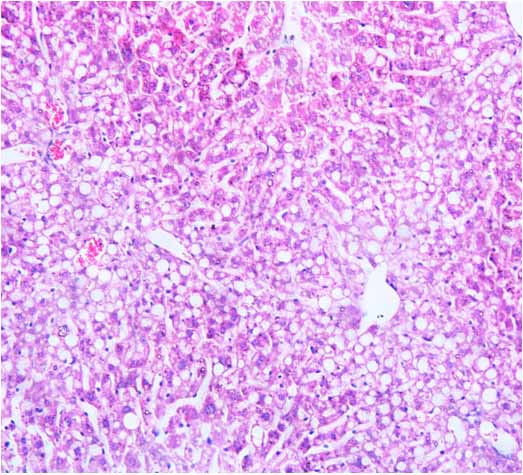

Supplement: Supplementary file 7 — Source Data for Figure 5 [file EMMM-15-e16592-s010.zip › Figure 5/Fig.5E/HE/3.jpg]

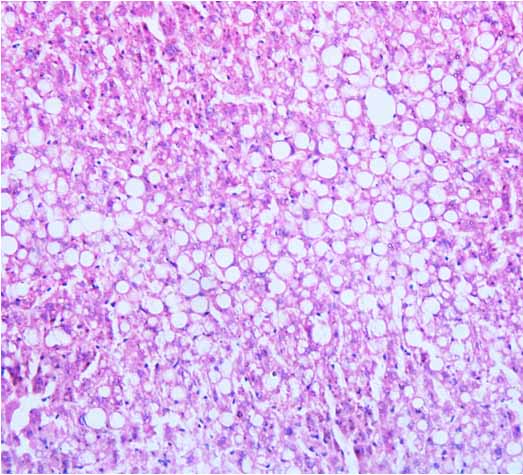

Supplement: Supplementary file 7 — Source Data for Figure 5 [file EMMM-15-e16592-s010.zip › Figure 5/Fig.5E/HE/4.jpg]

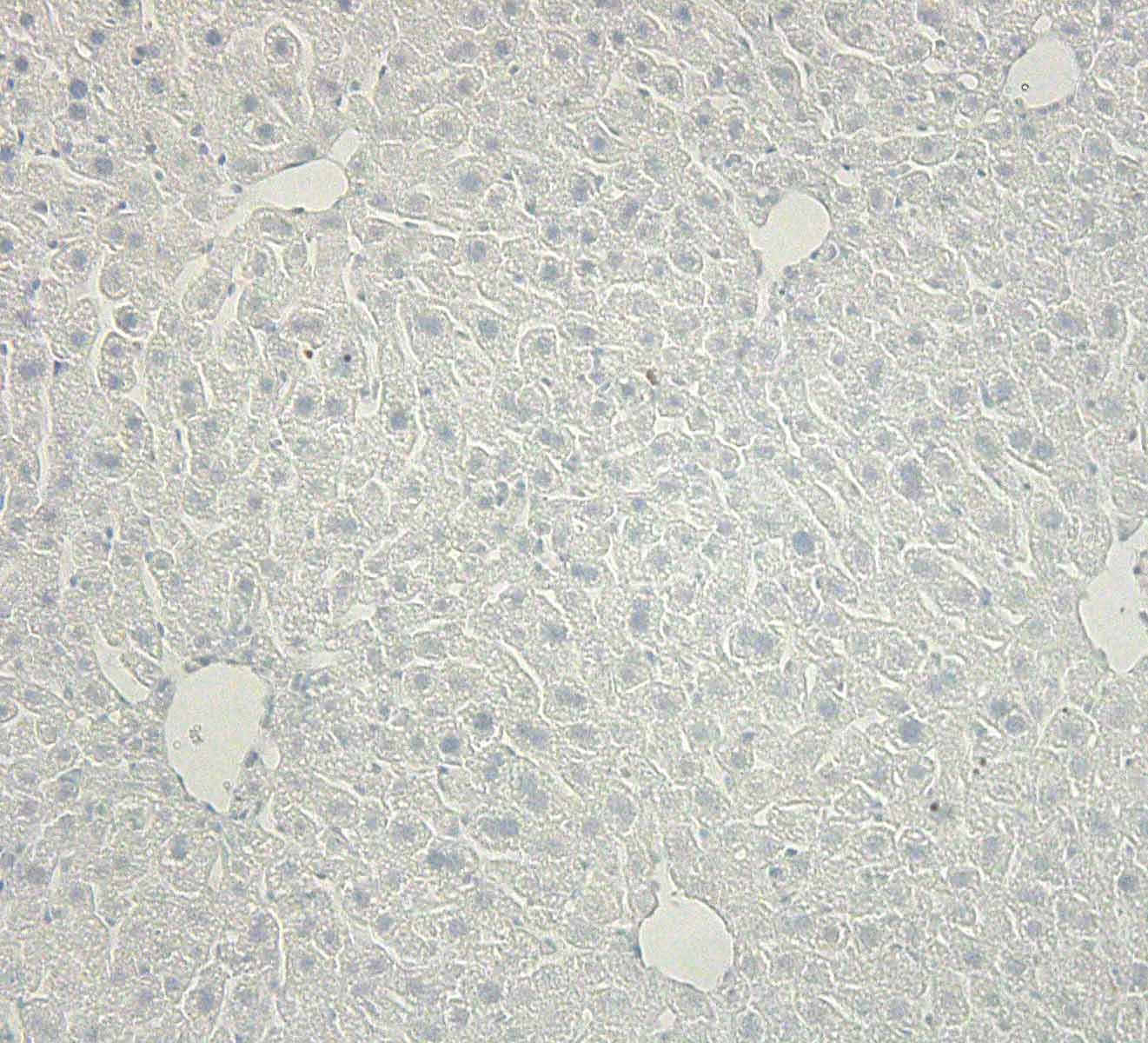

Supplement: Supplementary file 7 — Source Data for Figure 5 [file EMMM-15-e16592-s010.zip › Figure 5/Fig.5E/LY6G/1.jpg]

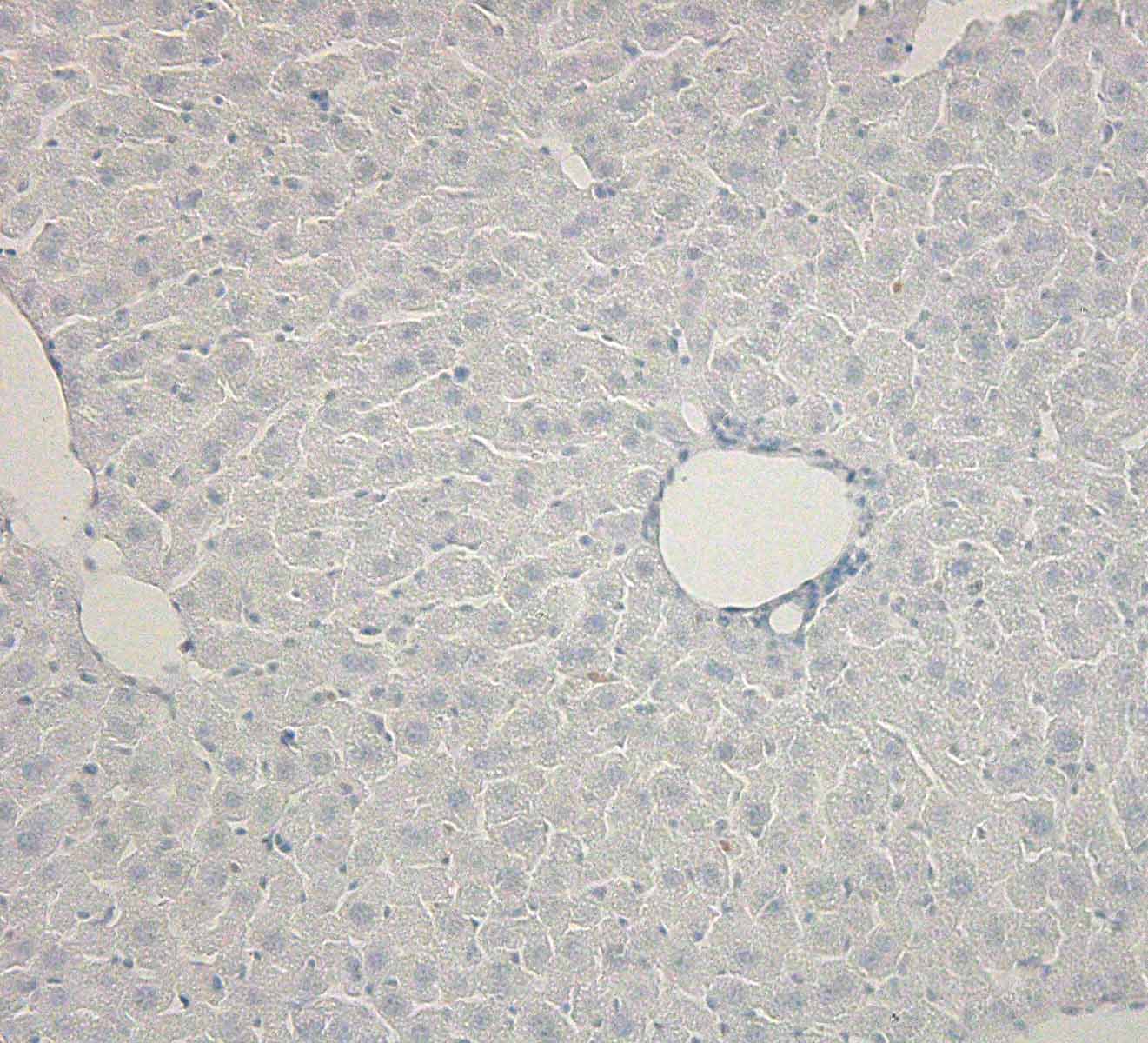

Supplement: Supplementary file 7 — Source Data for Figure 5 [file EMMM-15-e16592-s010.zip › Figure 5/Fig.5E/LY6G/2.jpg]

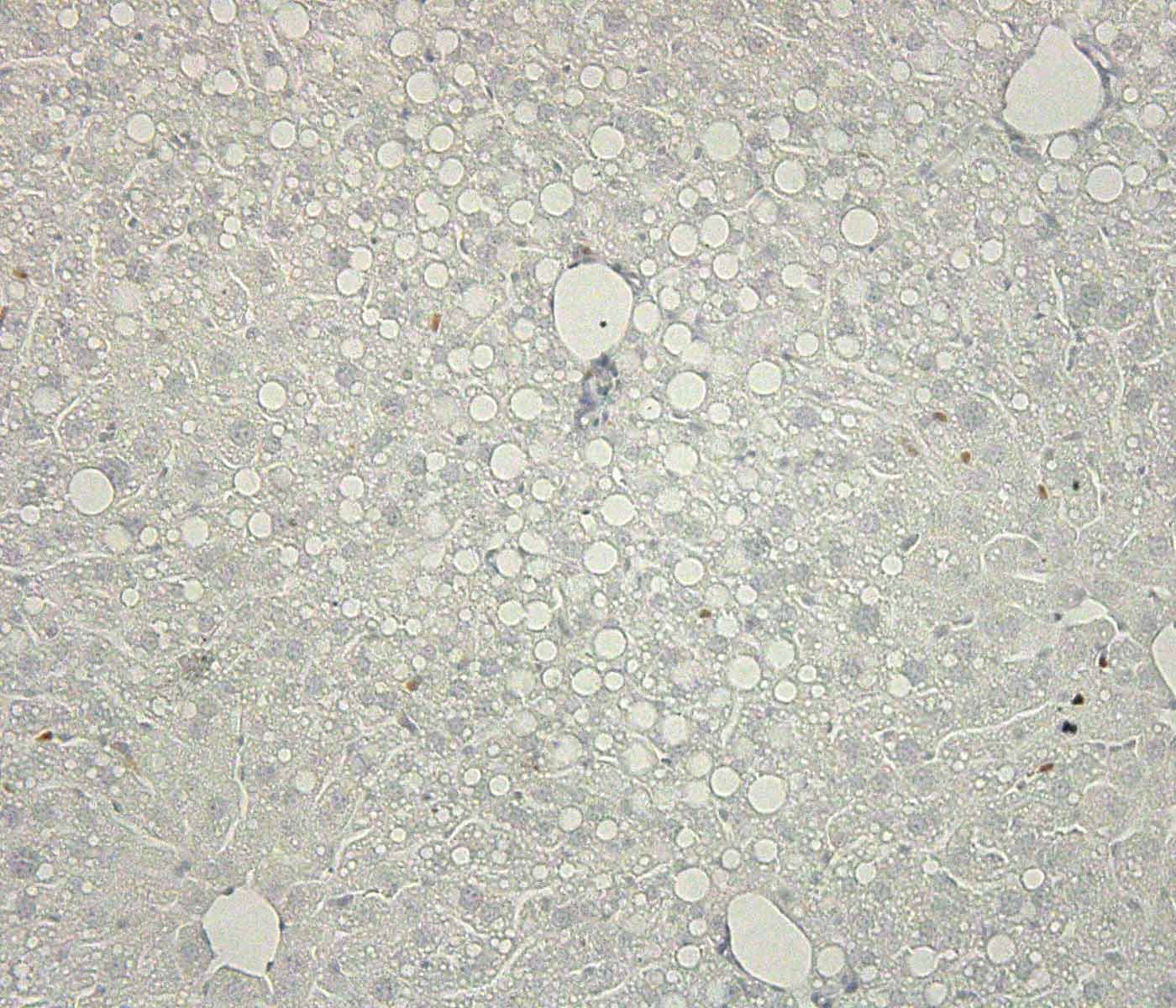

Supplement: Supplementary file 7 — Source Data for Figure 5 [file EMMM-15-e16592-s010.zip › Figure 5/Fig.5E/LY6G/3.jpg]

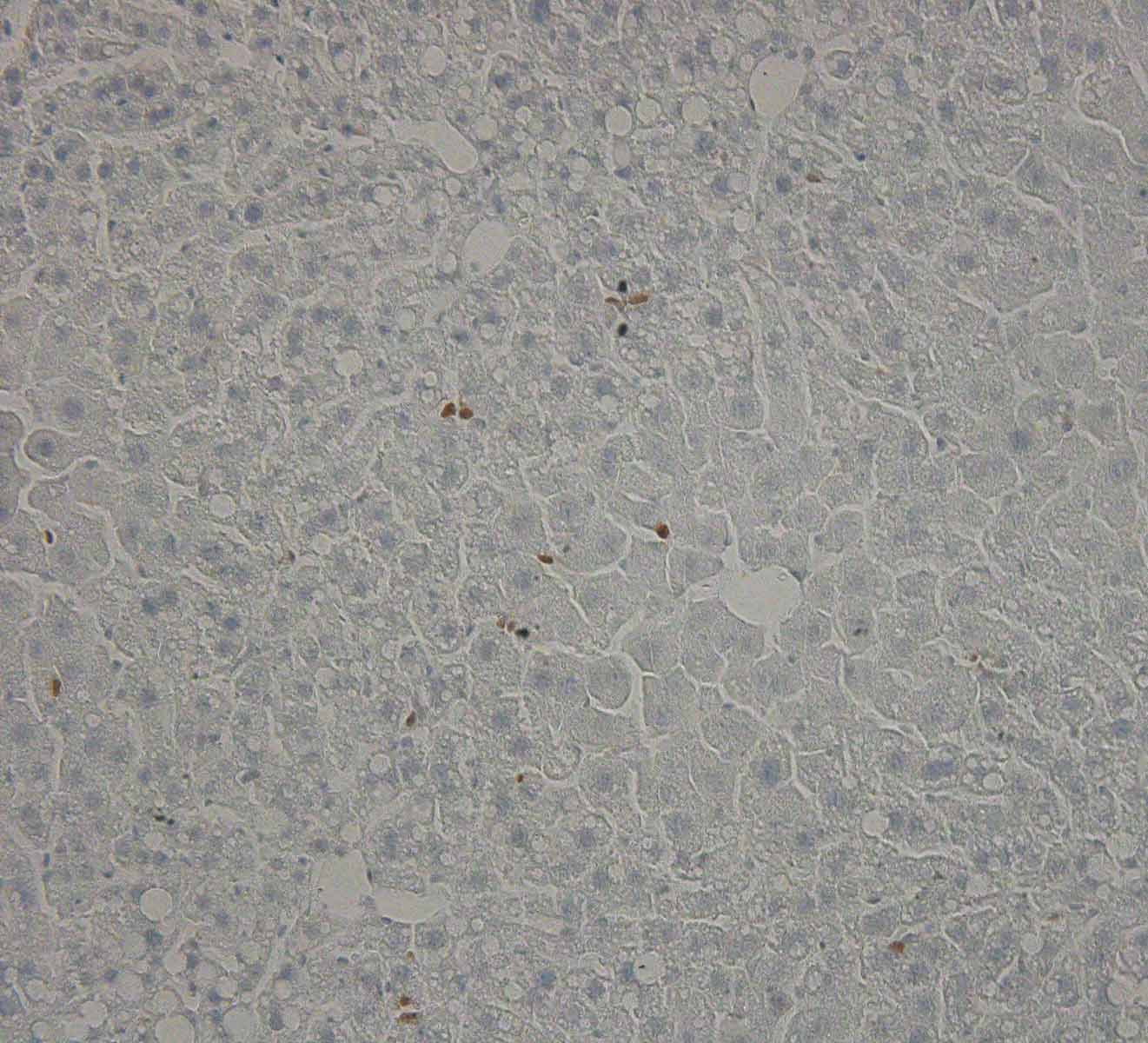

Supplement: Supplementary file 7 — Source Data for Figure 5 [file EMMM-15-e16592-s010.zip › Figure 5/Fig.5E/LY6G/4.jpg]

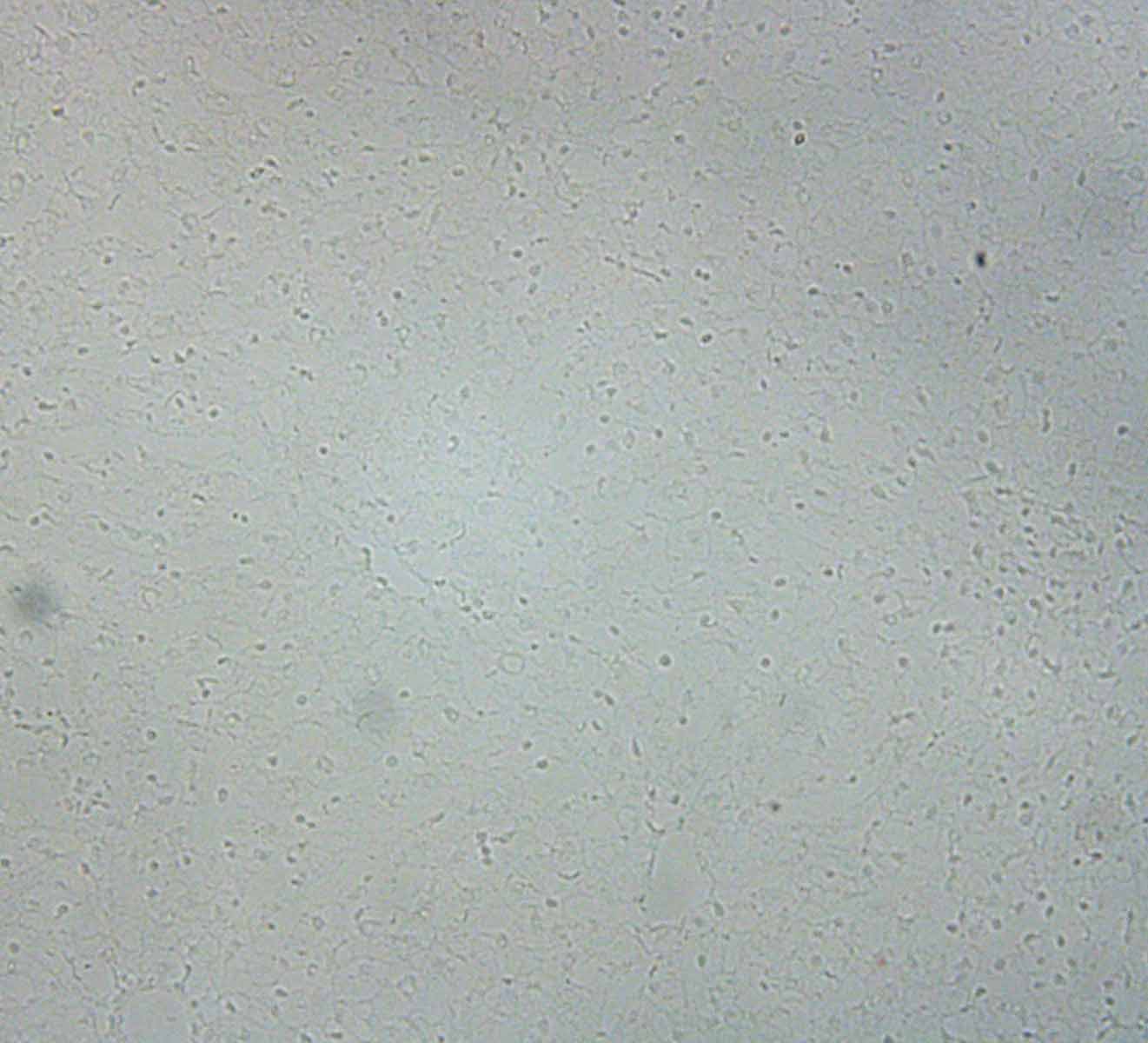

Supplement: Supplementary file 7 — Source Data for Figure 5 [file EMMM-15-e16592-s010.zip › Figure 5/Fig.5E/ORO/1.jpg]

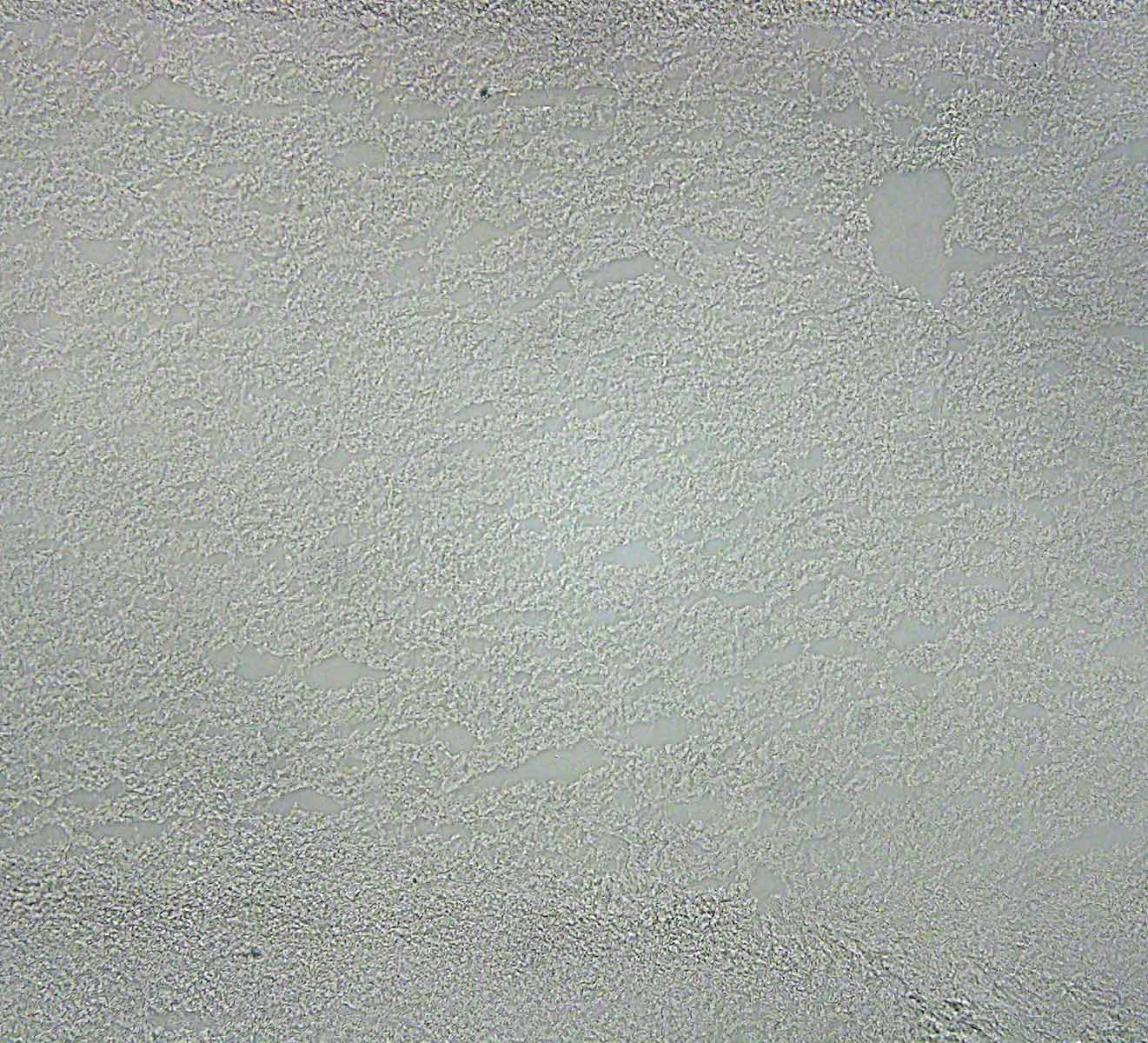

Supplement: Supplementary file 7 — Source Data for Figure 5 [file EMMM-15-e16592-s010.zip › Figure 5/Fig.5E/ORO/2.jpg]

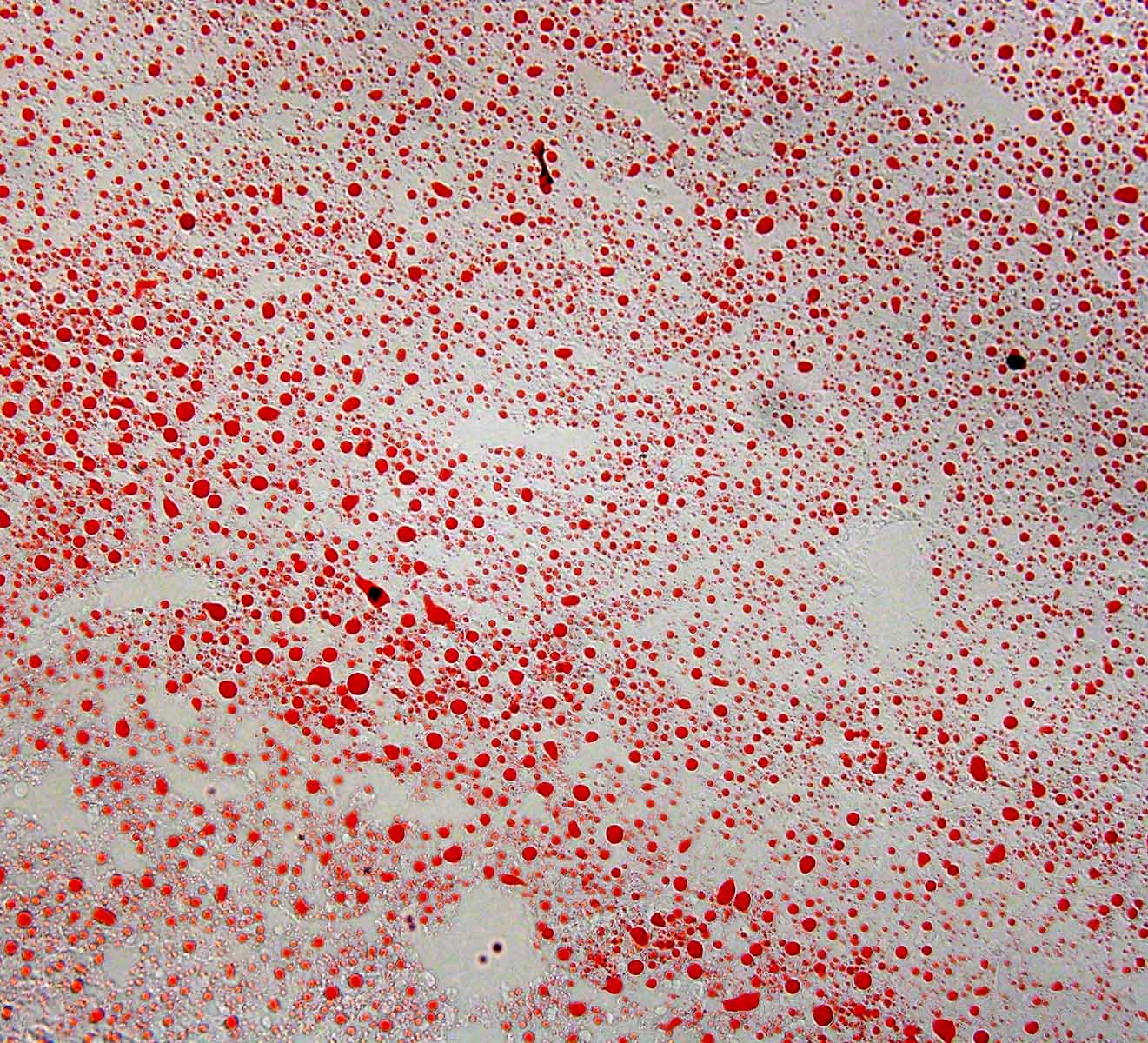

Supplement: Supplementary file 7 — Source Data for Figure 5 [file EMMM-15-e16592-s010.zip › Figure 5/Fig.5E/ORO/3.jpg]

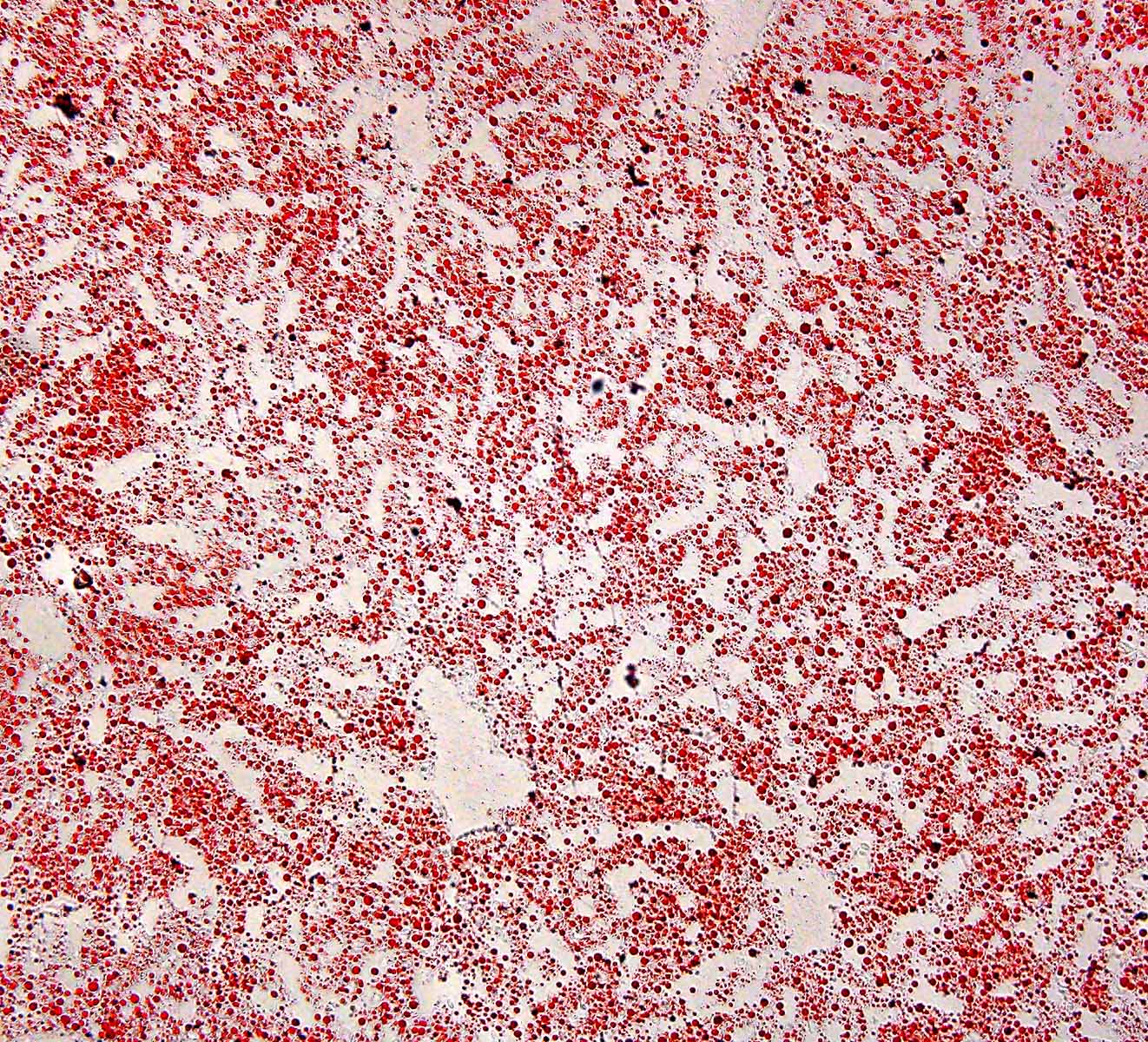

Supplement: Supplementary file 7 — Source Data for Figure 5 [file EMMM-15-e16592-s010.zip › Figure 5/Fig.5E/ORO/4.jpg]

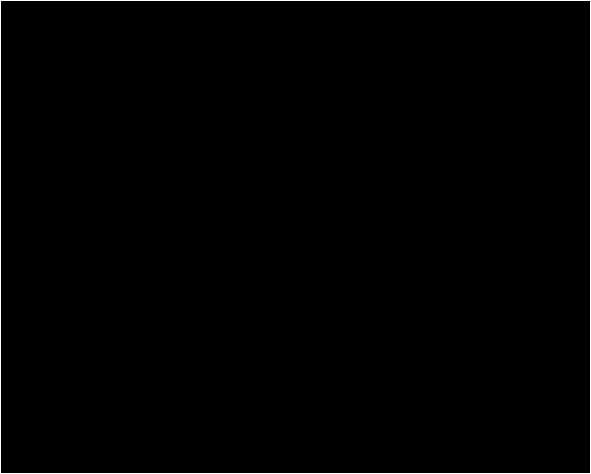

Supplement: Supplementary file 7 — Source Data for Figure 5 [file EMMM-15-e16592-s010.zip › Figure 5/Fig.5J/DHE/1.jpg]

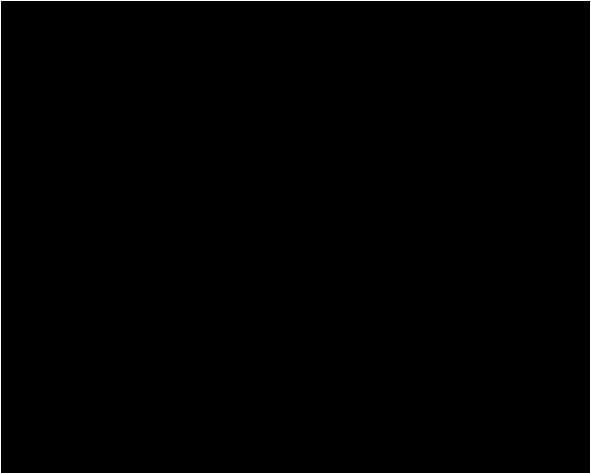

Supplement: Supplementary file 7 — Source Data for Figure 5 [file EMMM-15-e16592-s010.zip › Figure 5/Fig.5J/DHE/2.jpg]

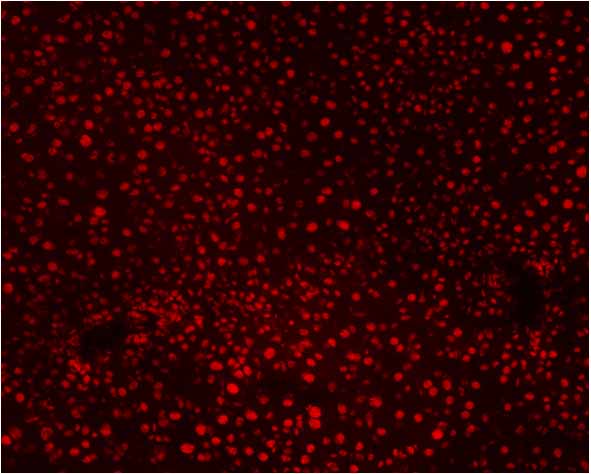

Supplement: Supplementary file 7 — Source Data for Figure 5 [file EMMM-15-e16592-s010.zip › Figure 5/Fig.5J/DHE/3.jpg]

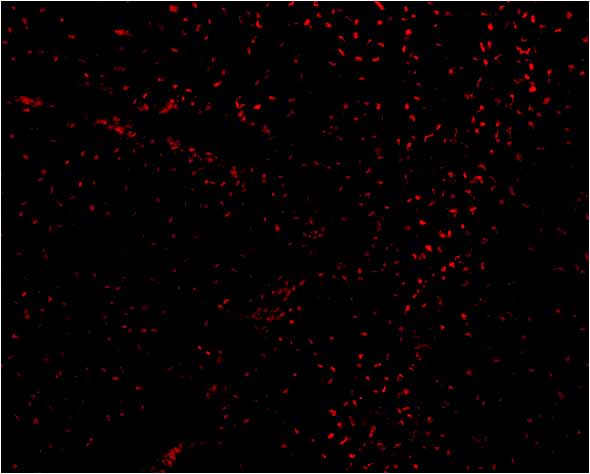

Supplement: Supplementary file 7 — Source Data for Figure 5 [file EMMM-15-e16592-s010.zip › Figure 5/Fig.5J/DHE/4.jpg]

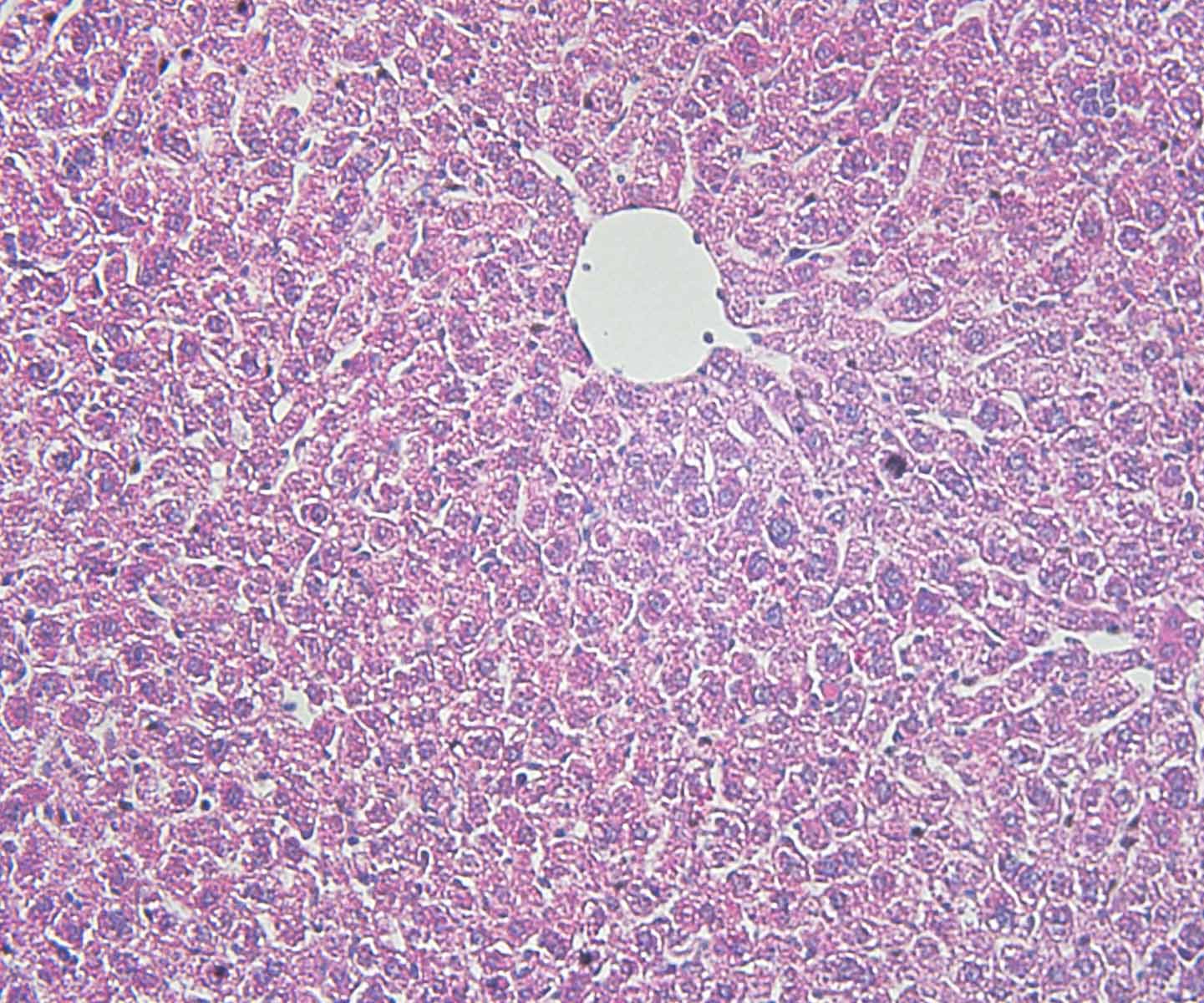

Supplement: Supplementary file 7 — Source Data for Figure 5 [file EMMM-15-e16592-s010.zip › Figure 5/Fig.5J/HE/1.jpg]

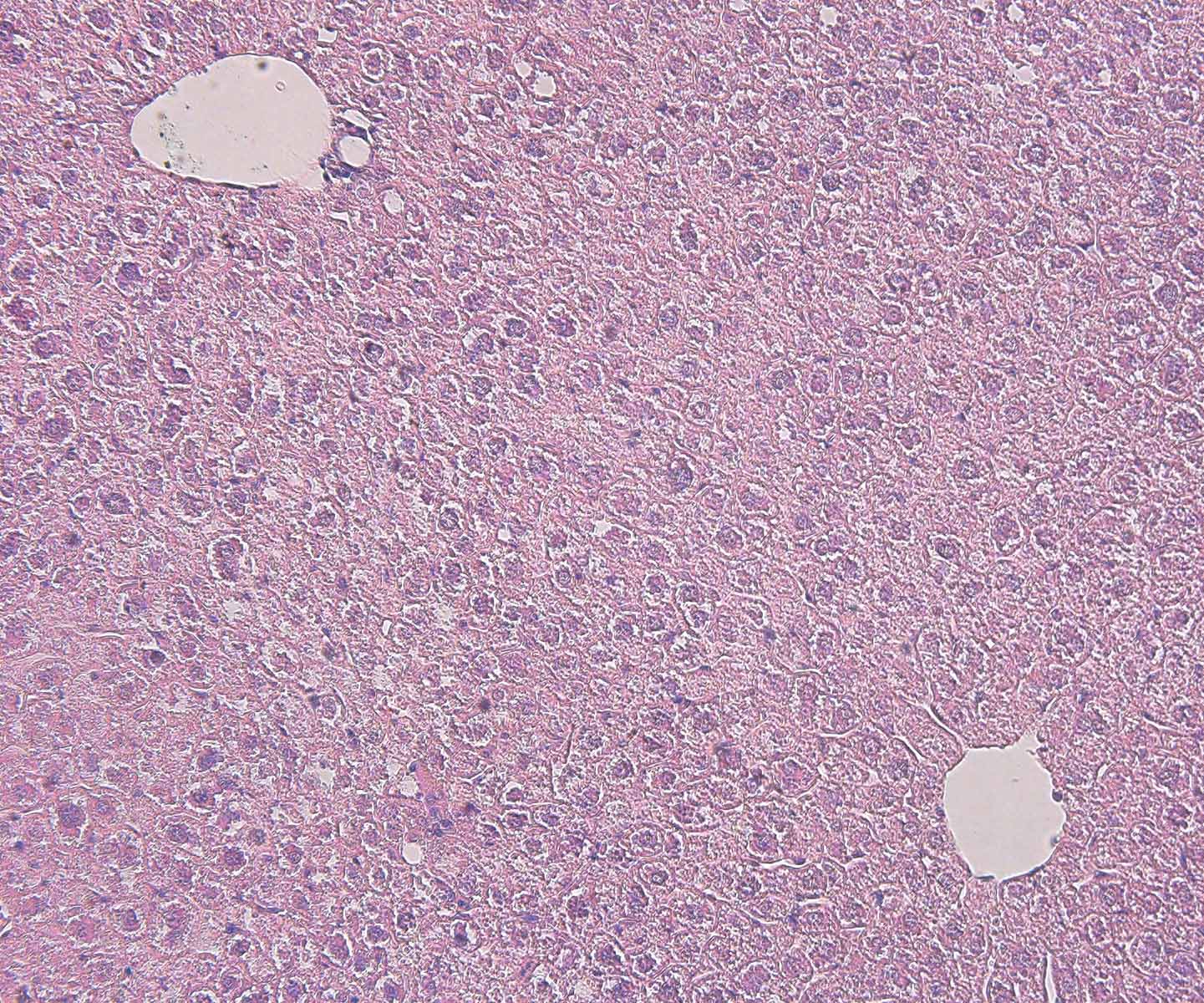

Supplement: Supplementary file 7 — Source Data for Figure 5 [file EMMM-15-e16592-s010.zip › Figure 5/Fig.5J/HE/2.jpg]

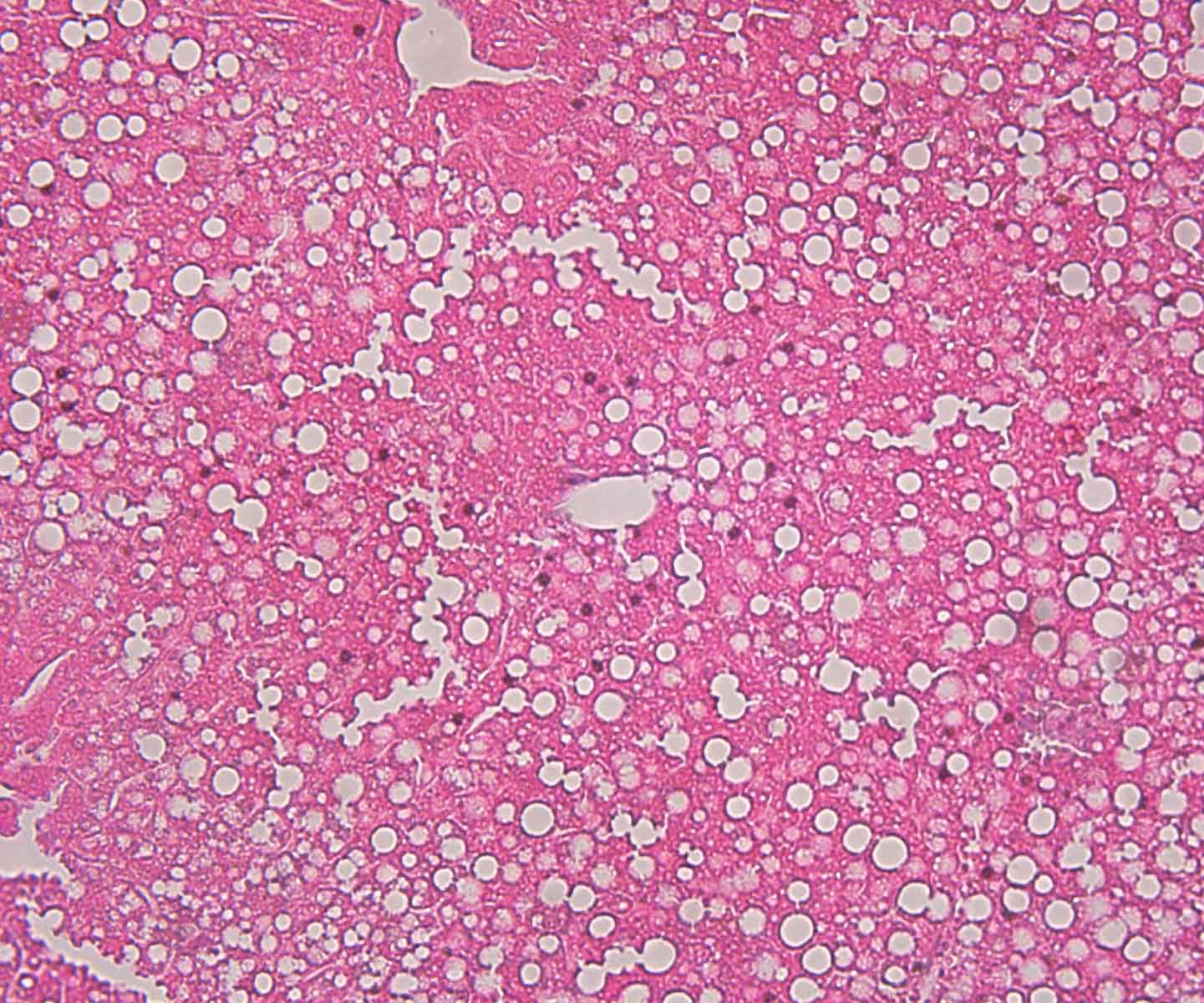

Supplement: Supplementary file 7 — Source Data for Figure 5 [file EMMM-15-e16592-s010.zip › Figure 5/Fig.5J/HE/3.jpg]

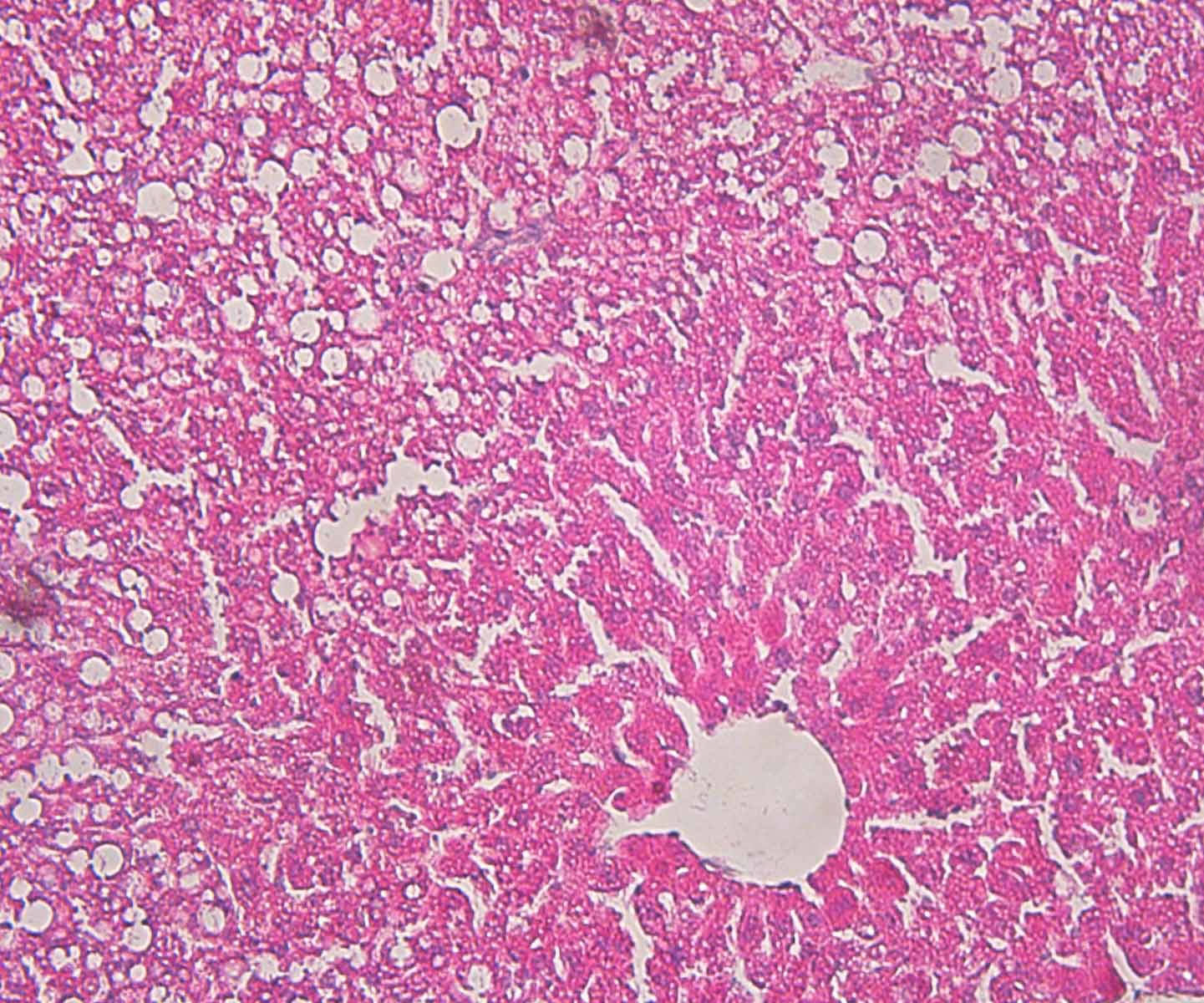

Supplement: Supplementary file 7 — Source Data for Figure 5 [file EMMM-15-e16592-s010.zip › Figure 5/Fig.5J/HE/4.jpg]

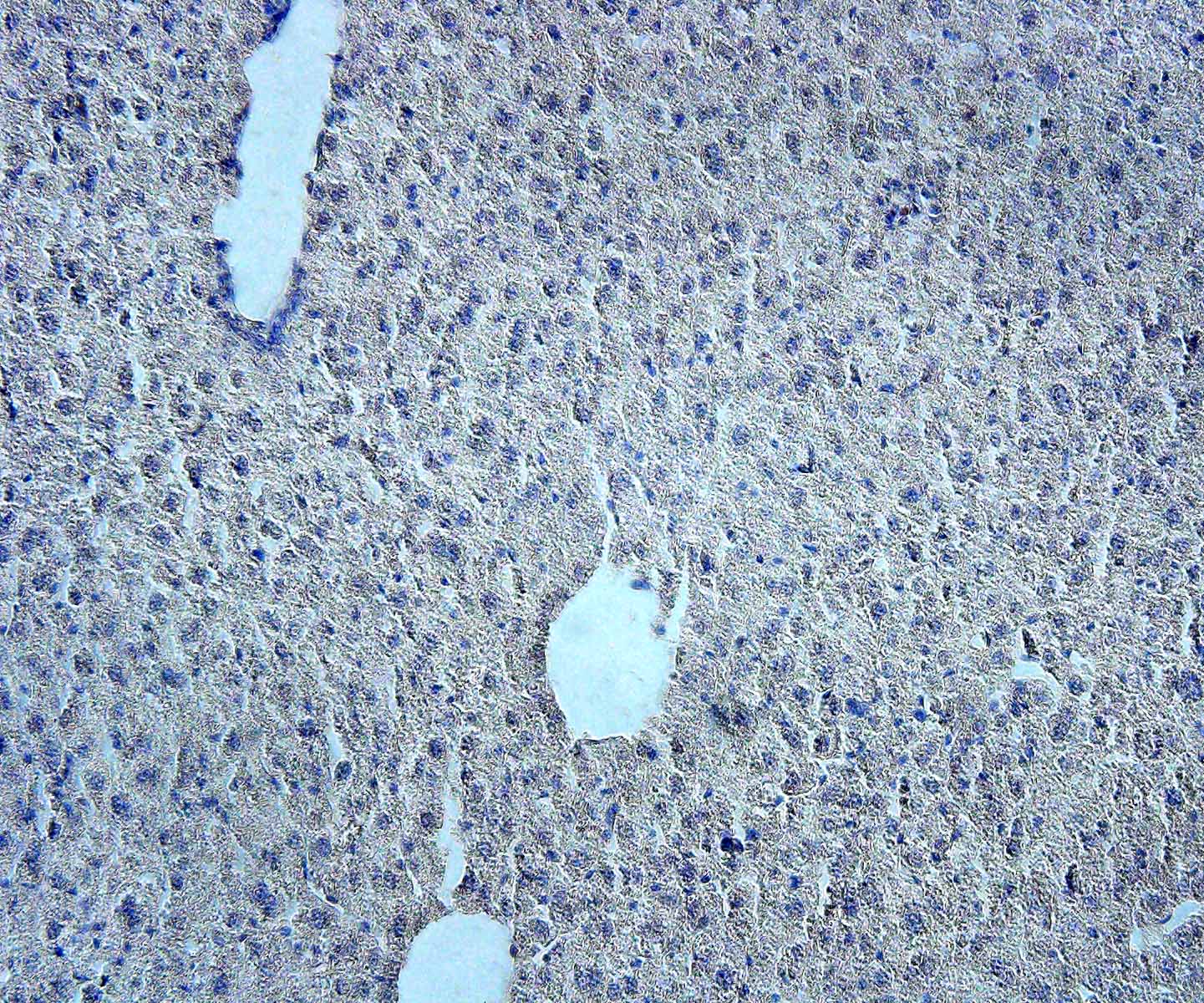

Supplement: Supplementary file 7 — Source Data for Figure 5 [file EMMM-15-e16592-s010.zip › Figure 5/Fig.5J/LY6G/1.jpg]

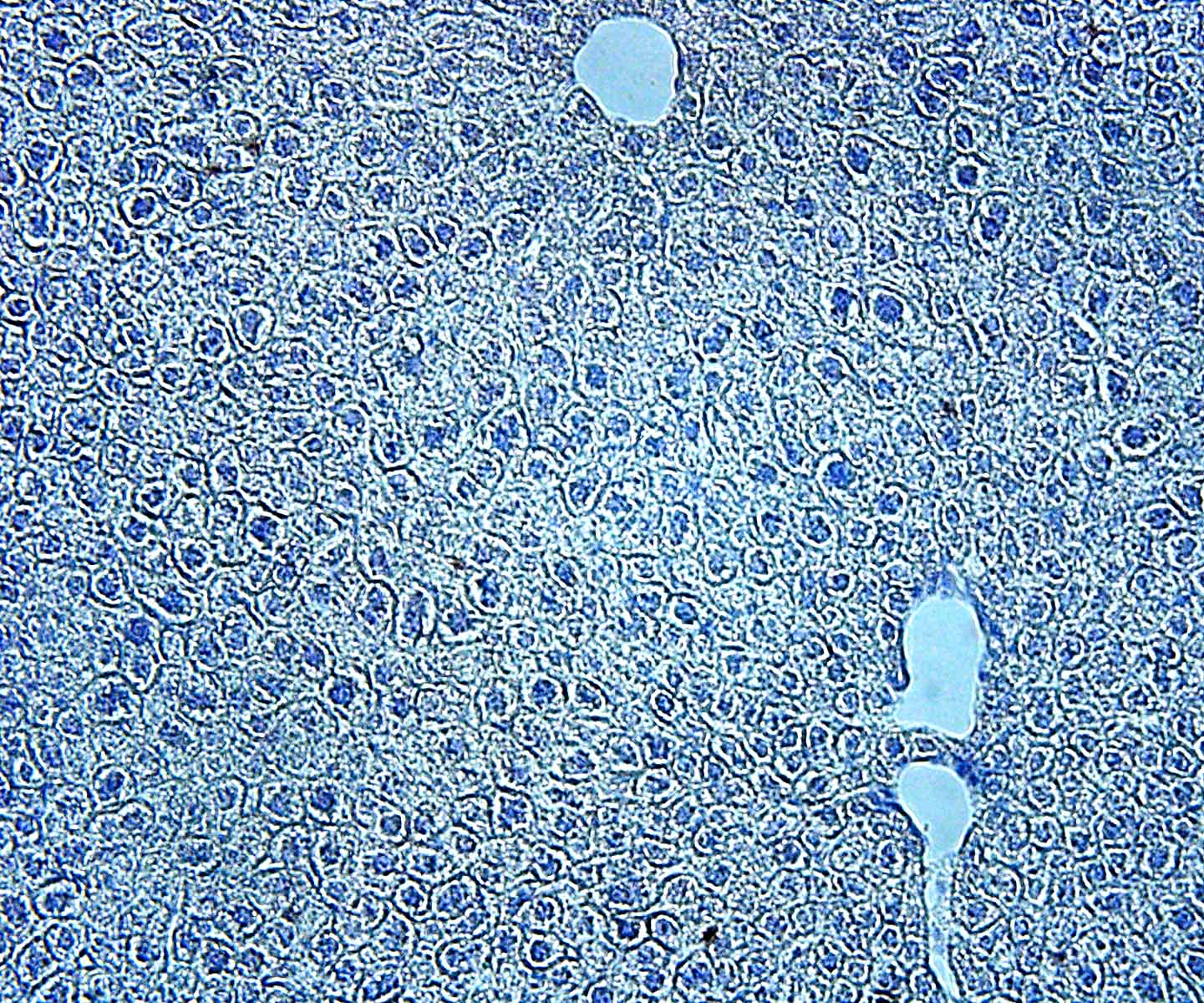

Supplement: Supplementary file 7 — Source Data for Figure 5 [file EMMM-15-e16592-s010.zip › Figure 5/Fig.5J/LY6G/2.jpg]

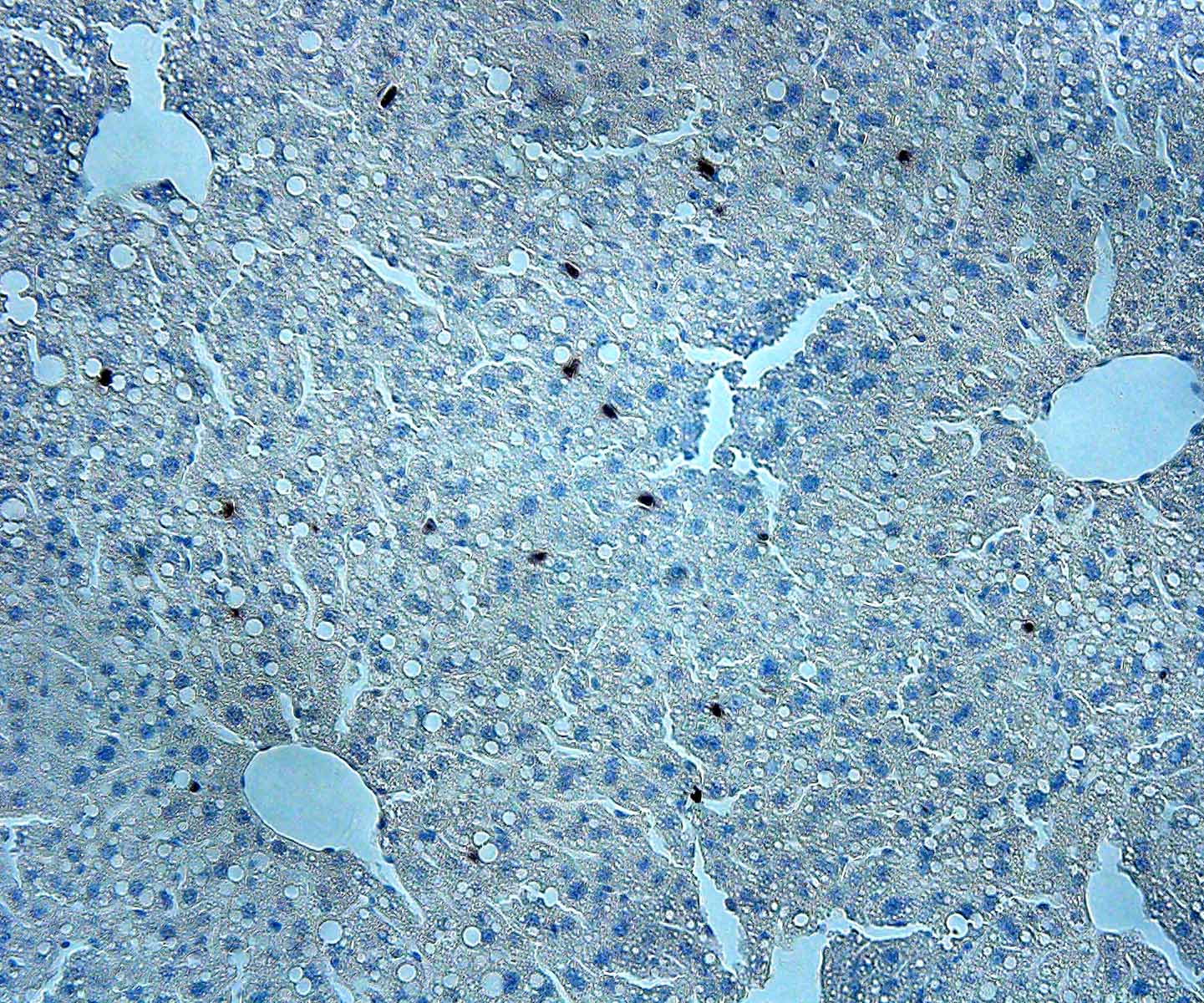

Supplement: Supplementary file 7 — Source Data for Figure 5 [file EMMM-15-e16592-s010.zip › Figure 5/Fig.5J/LY6G/3.jpg]
