# Supplementary figures and images for: Targetable Brg1‐CXCL14 axis contributes to alcoholic liver injury by driving neutrophil trafficking (part 2 of 2)
Source: EMBO Mol Med. 2023 Feb 1;15(3):e16592. doi: 10.15252/emmm.202216592 (PMC9994483; doi:10.15252/emmm.202216592)

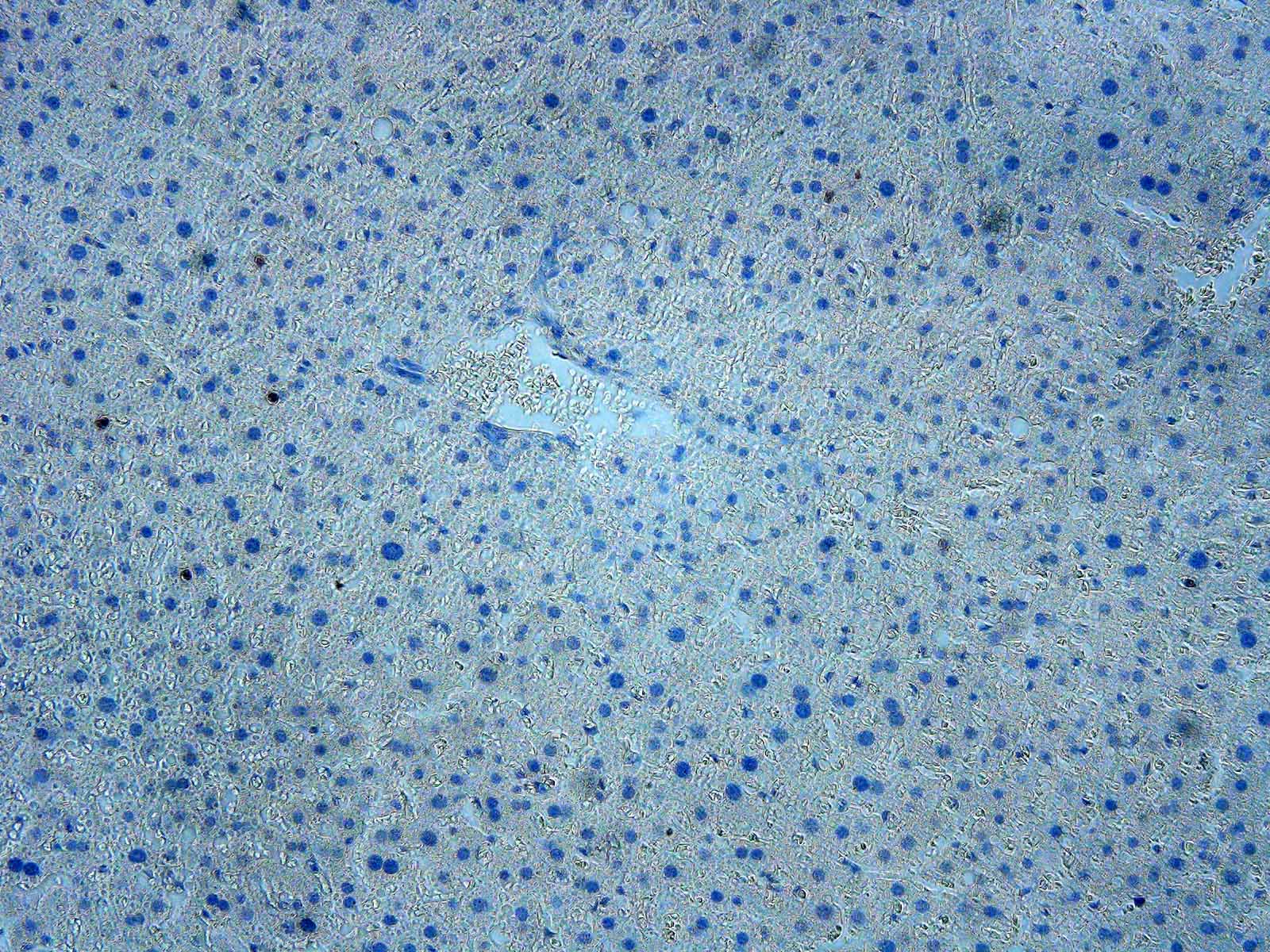

Supplement: Supplementary file 7 — Source Data for Figure 5 [file EMMM-15-e16592-s010.zip › Figure 5/Fig.5J/LY6G/4.jpg]

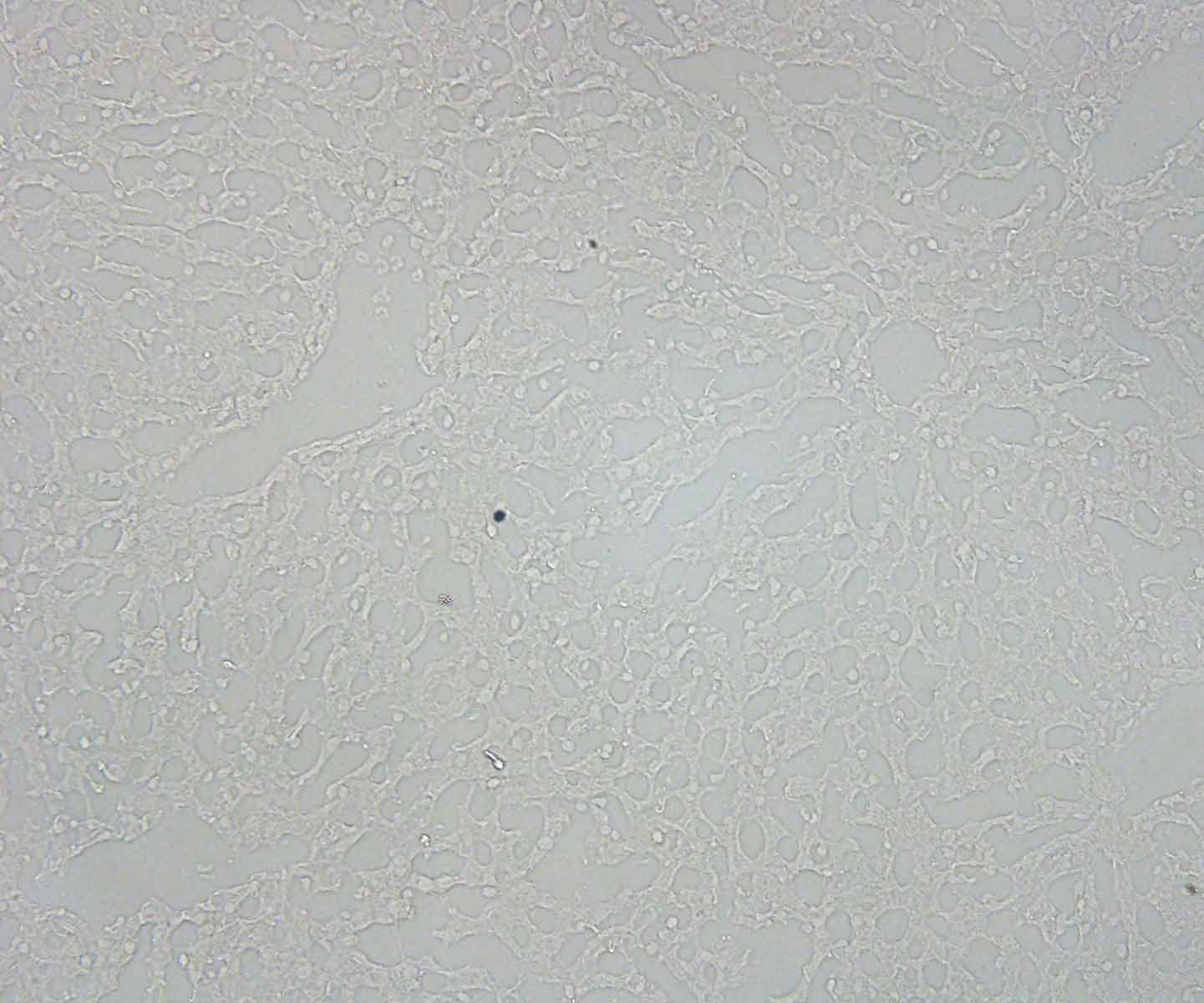

Supplement: Supplementary file 7 — Source Data for Figure 5 [file EMMM-15-e16592-s010.zip › Figure 5/Fig.5J/ORO/1.jpg]

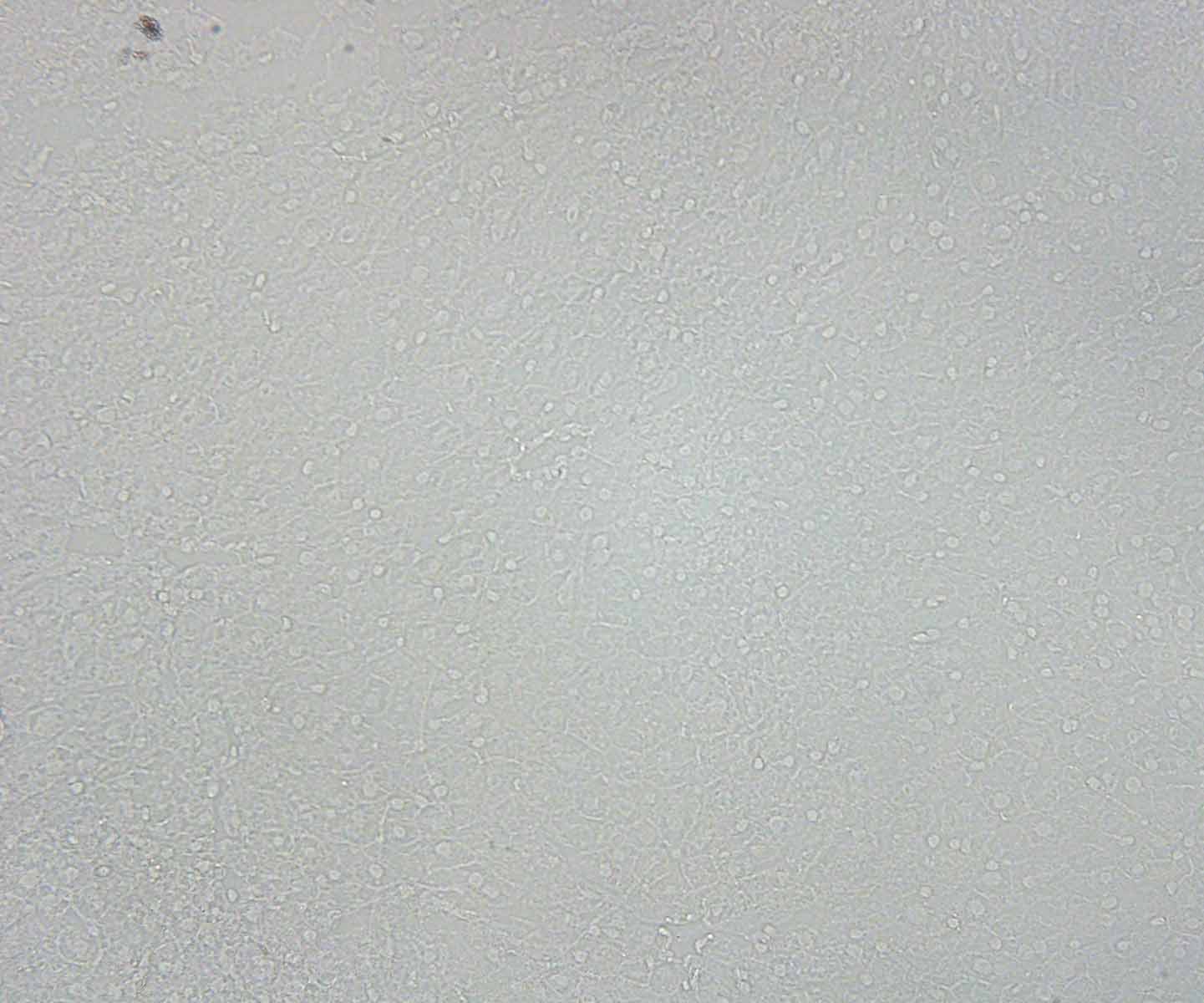

Supplement: Supplementary file 7 — Source Data for Figure 5 [file EMMM-15-e16592-s010.zip › Figure 5/Fig.5J/ORO/2.jpg]

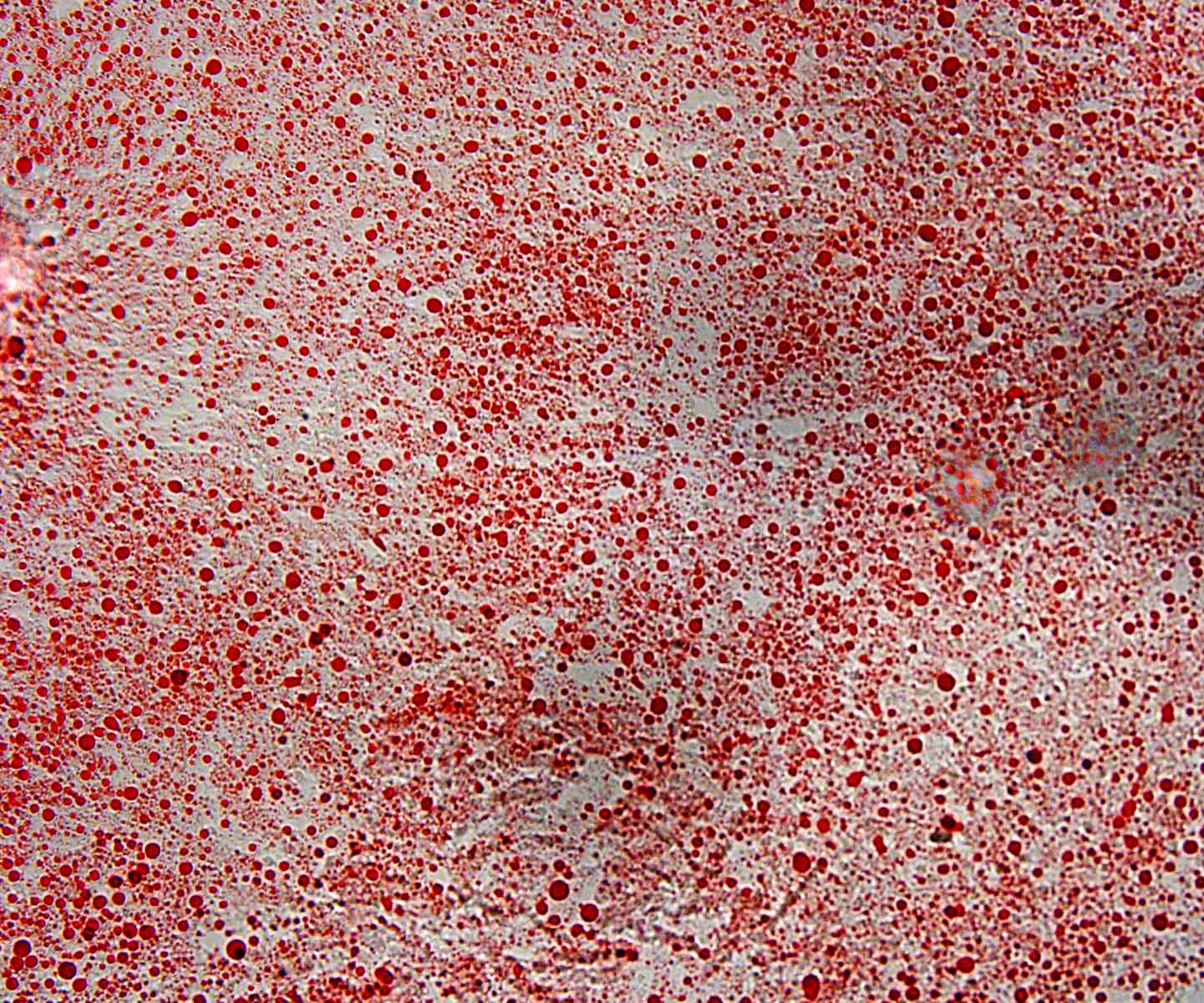

Supplement: Supplementary file 7 — Source Data for Figure 5 [file EMMM-15-e16592-s010.zip › Figure 5/Fig.5J/ORO/3.jpg]

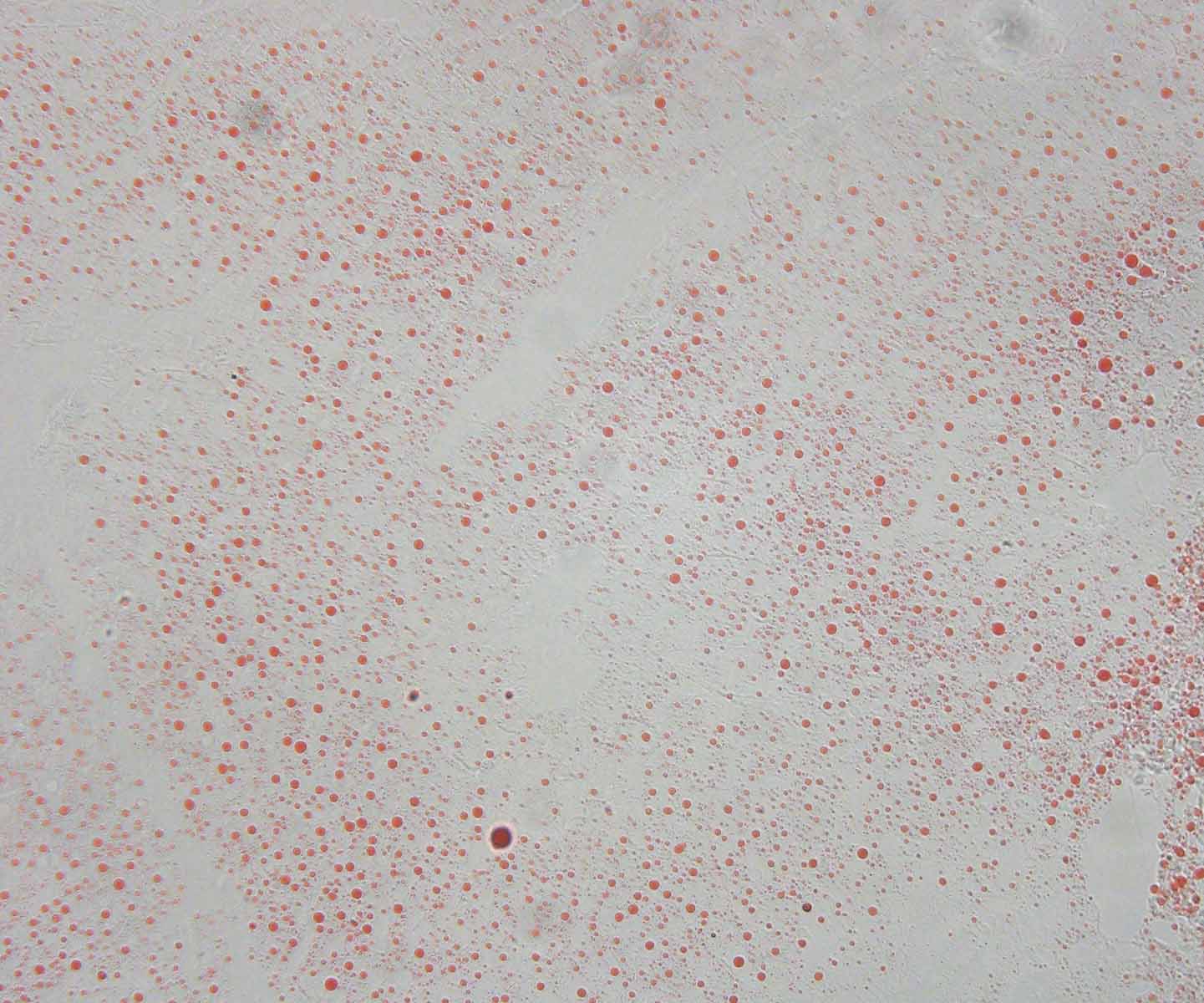

Supplement: Supplementary file 7 — Source Data for Figure 5 [file EMMM-15-e16592-s010.zip › Figure 5/Fig.5J/ORO/4.jpg]

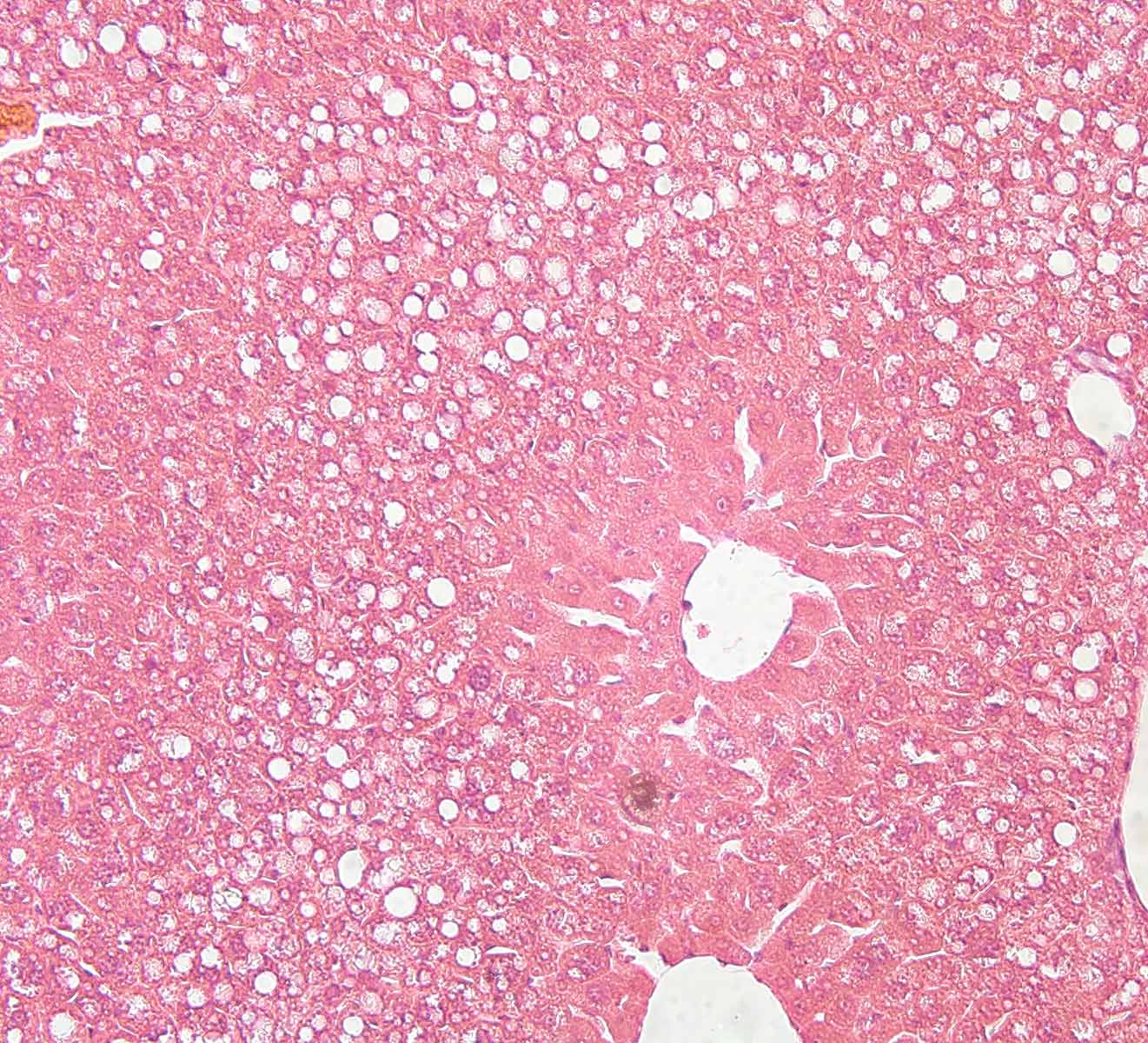

Supplement: Supplementary file 8 — Source Data for Figure 6 [file EMMM-15-e16592-s007.zip › Figure 6/Fig.6E/1.jpg]

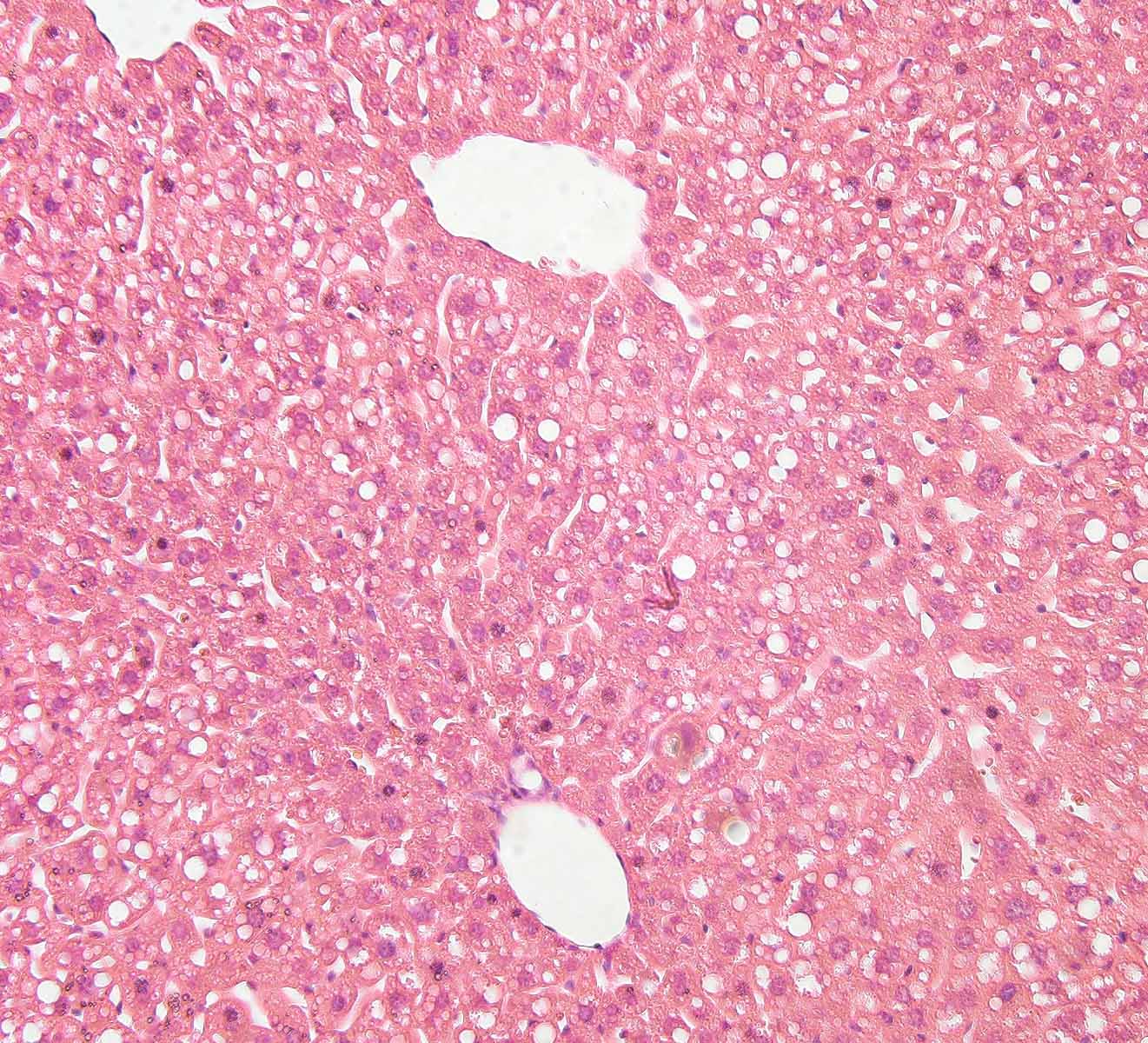

Supplement: Supplementary file 8 — Source Data for Figure 6 [file EMMM-15-e16592-s007.zip › Figure 6/Fig.6E/2.jpg]

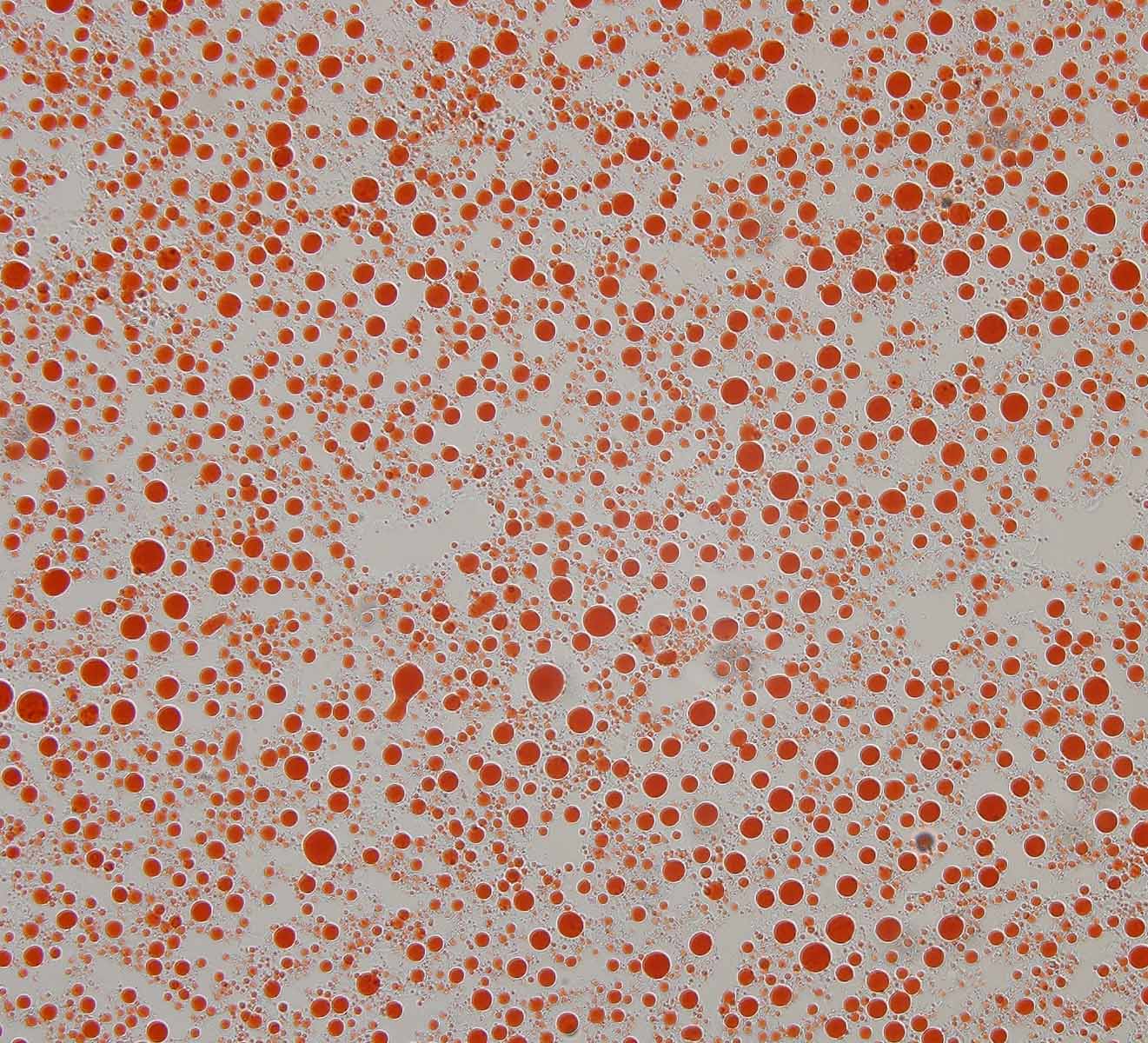

Supplement: Supplementary file 8 — Source Data for Figure 6 [file EMMM-15-e16592-s007.zip › Figure 6/Fig.6E/3.jpg]

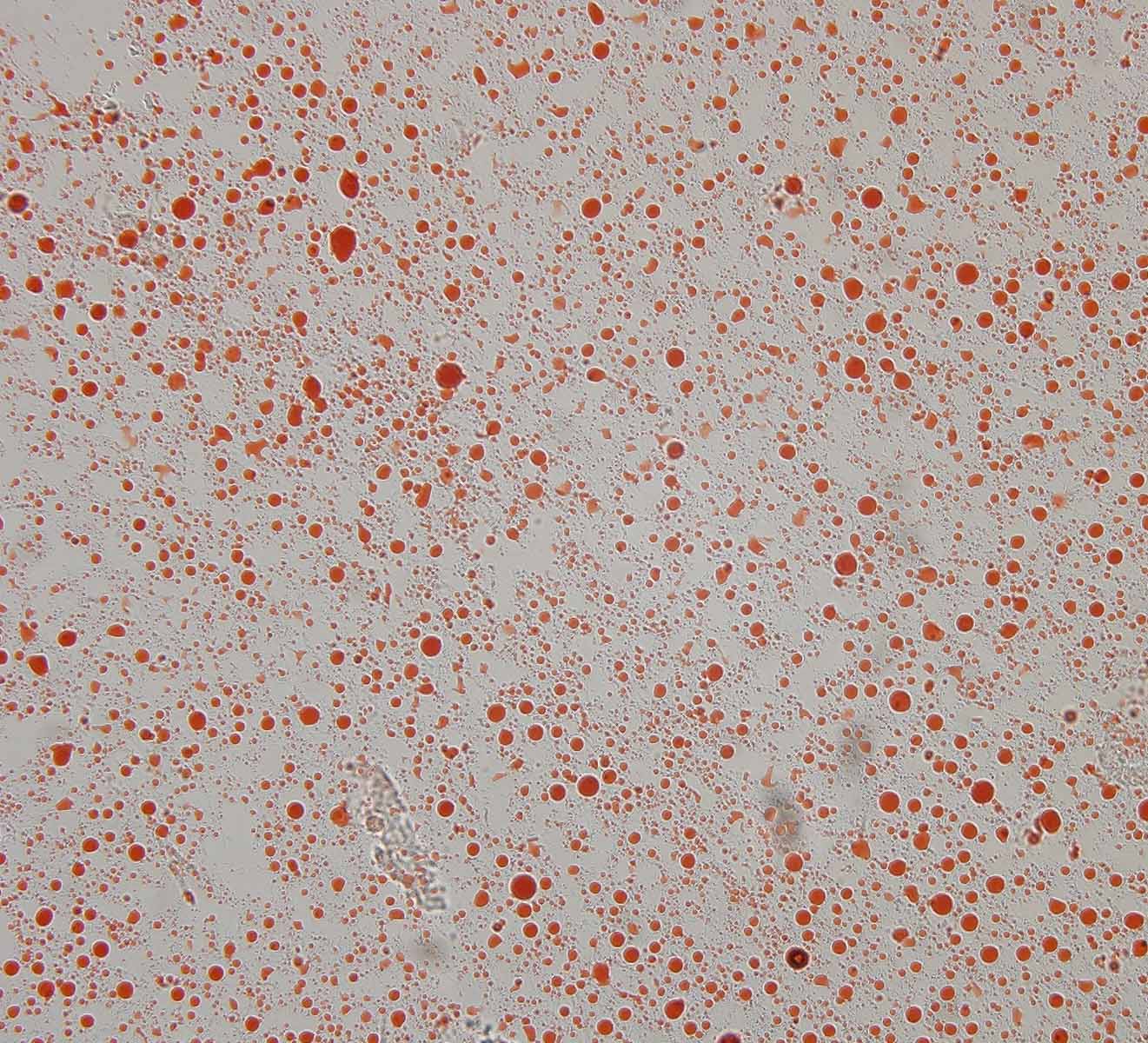

Supplement: Supplementary file 8 — Source Data for Figure 6 [file EMMM-15-e16592-s007.zip › Figure 6/Fig.6E/4.jpg]

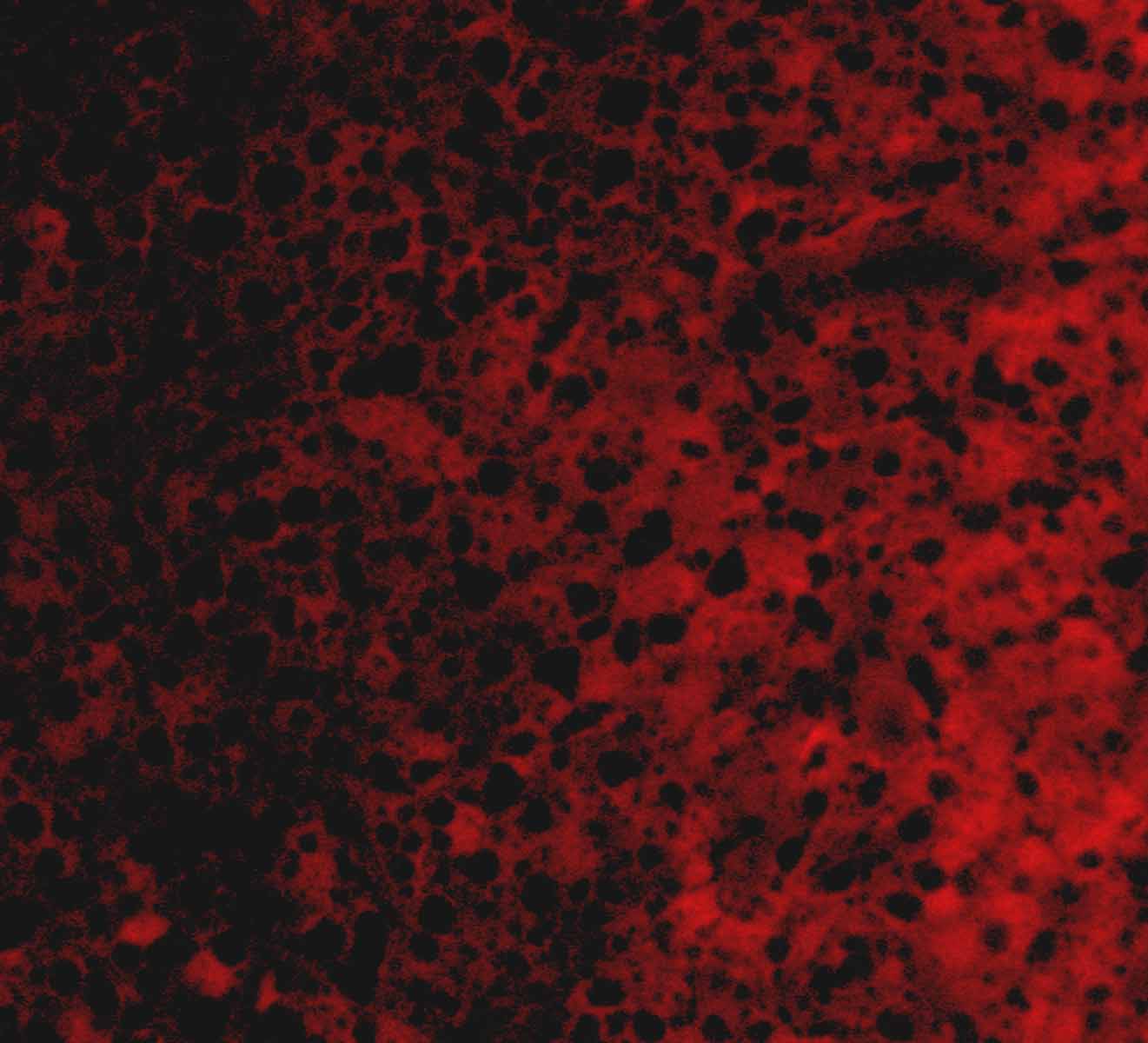

Supplement: Supplementary file 8 — Source Data for Figure 6 [file EMMM-15-e16592-s007.zip › Figure 6/Fig.6E/5.jpg]

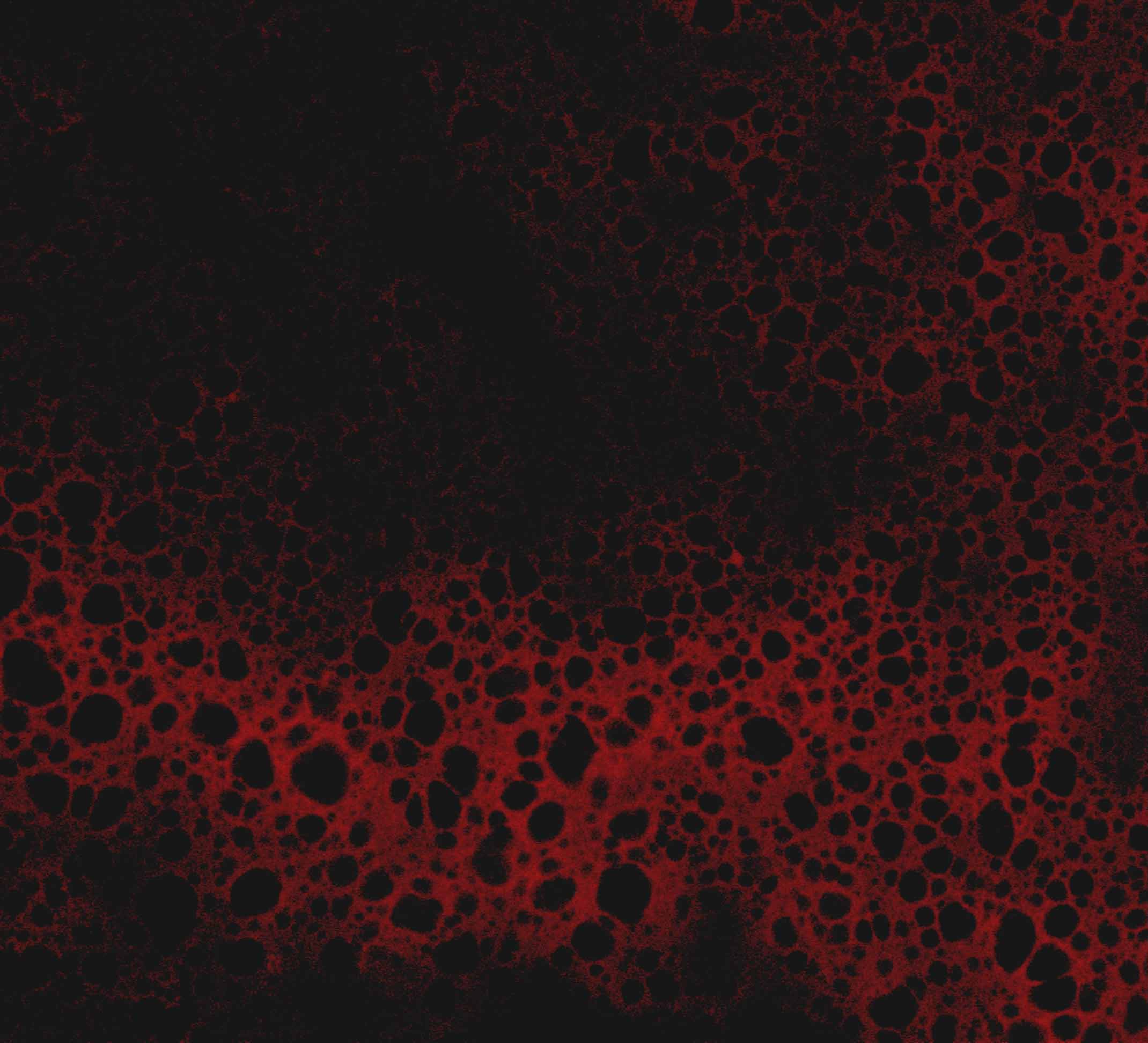

Supplement: Supplementary file 8 — Source Data for Figure 6 [file EMMM-15-e16592-s007.zip › Figure 6/Fig.6E/6.jpg]

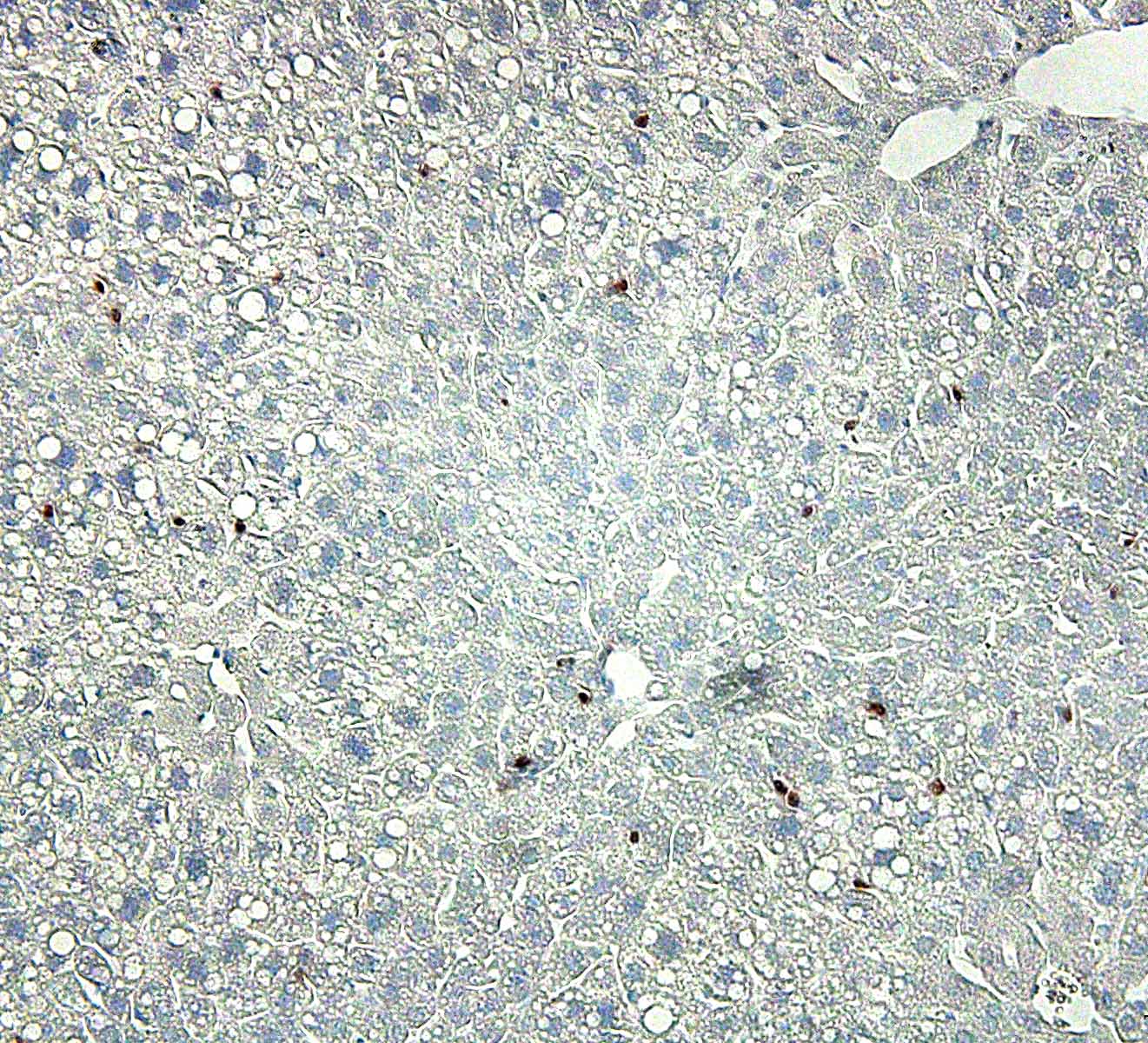

Supplement: Supplementary file 8 — Source Data for Figure 6 [file EMMM-15-e16592-s007.zip › Figure 6/Fig.6E/7.jpg]

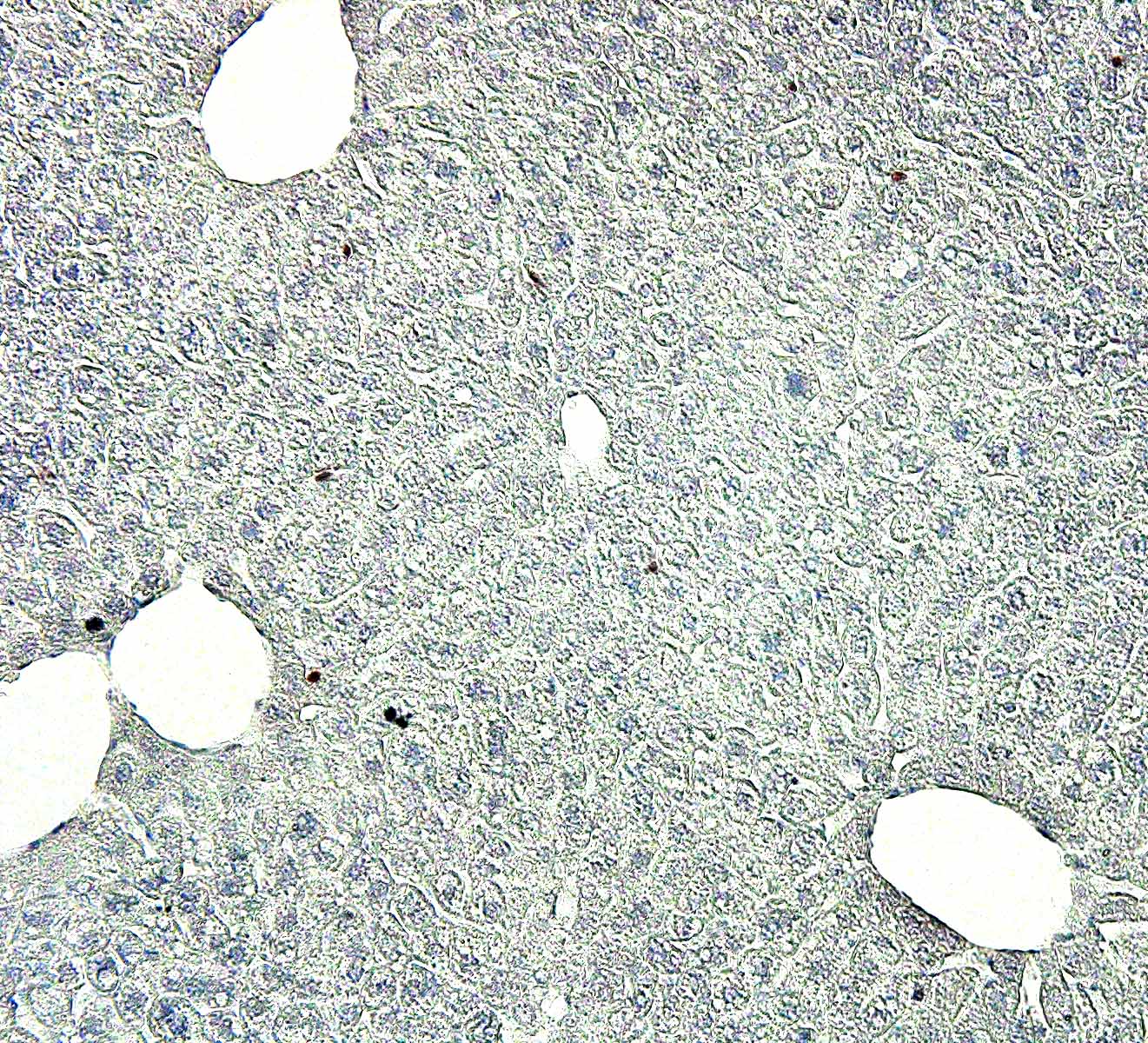

Supplement: Supplementary file 8 — Source Data for Figure 6 [file EMMM-15-e16592-s007.zip › Figure 6/Fig.6E/8.jpg]

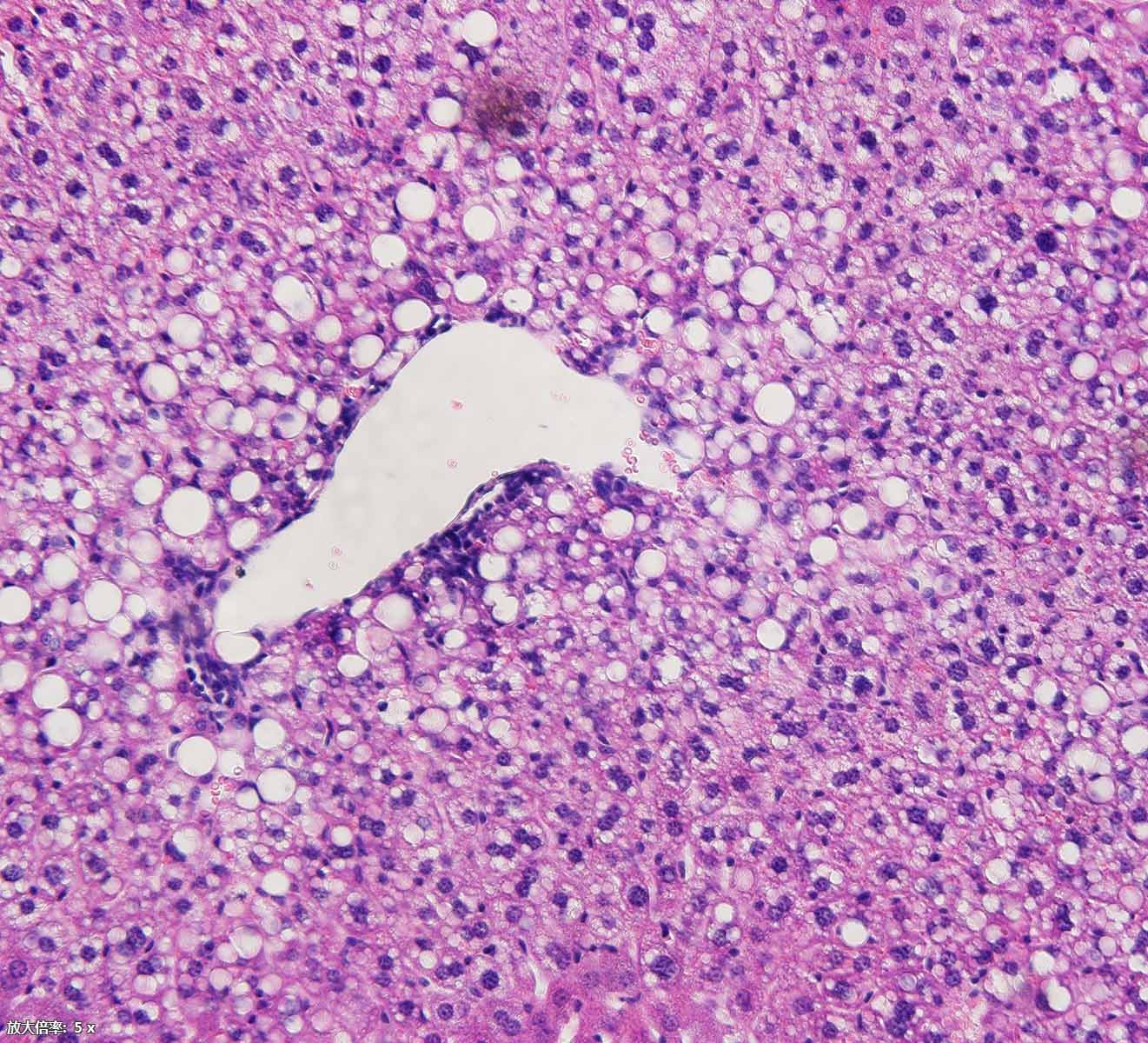

Supplement: Supplementary file 8 — Source Data for Figure 6 [file EMMM-15-e16592-s007.zip › Figure 6/Fig.6J/1.jpg]

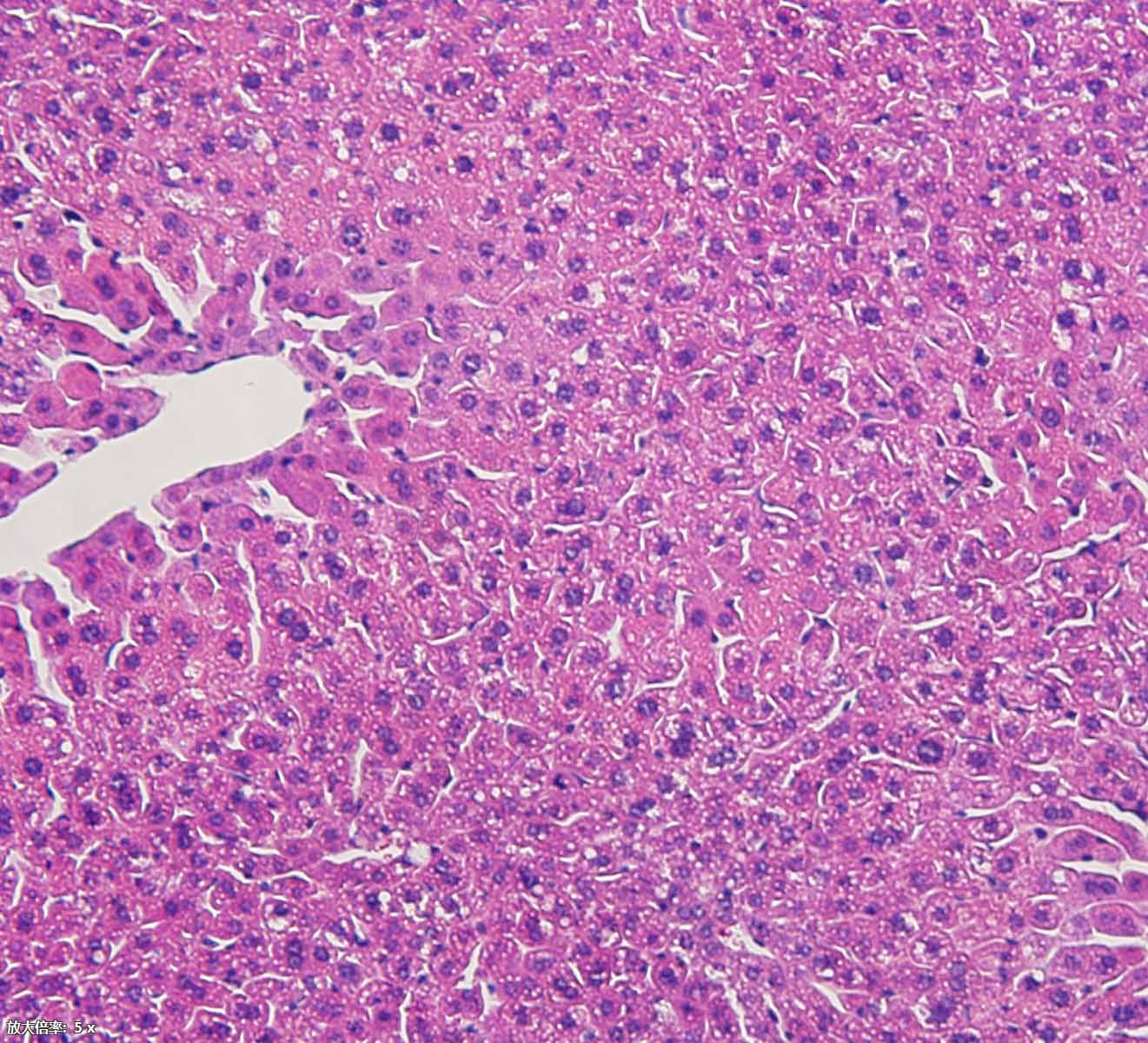

Supplement: Supplementary file 8 — Source Data for Figure 6 [file EMMM-15-e16592-s007.zip › Figure 6/Fig.6J/2.jpg]

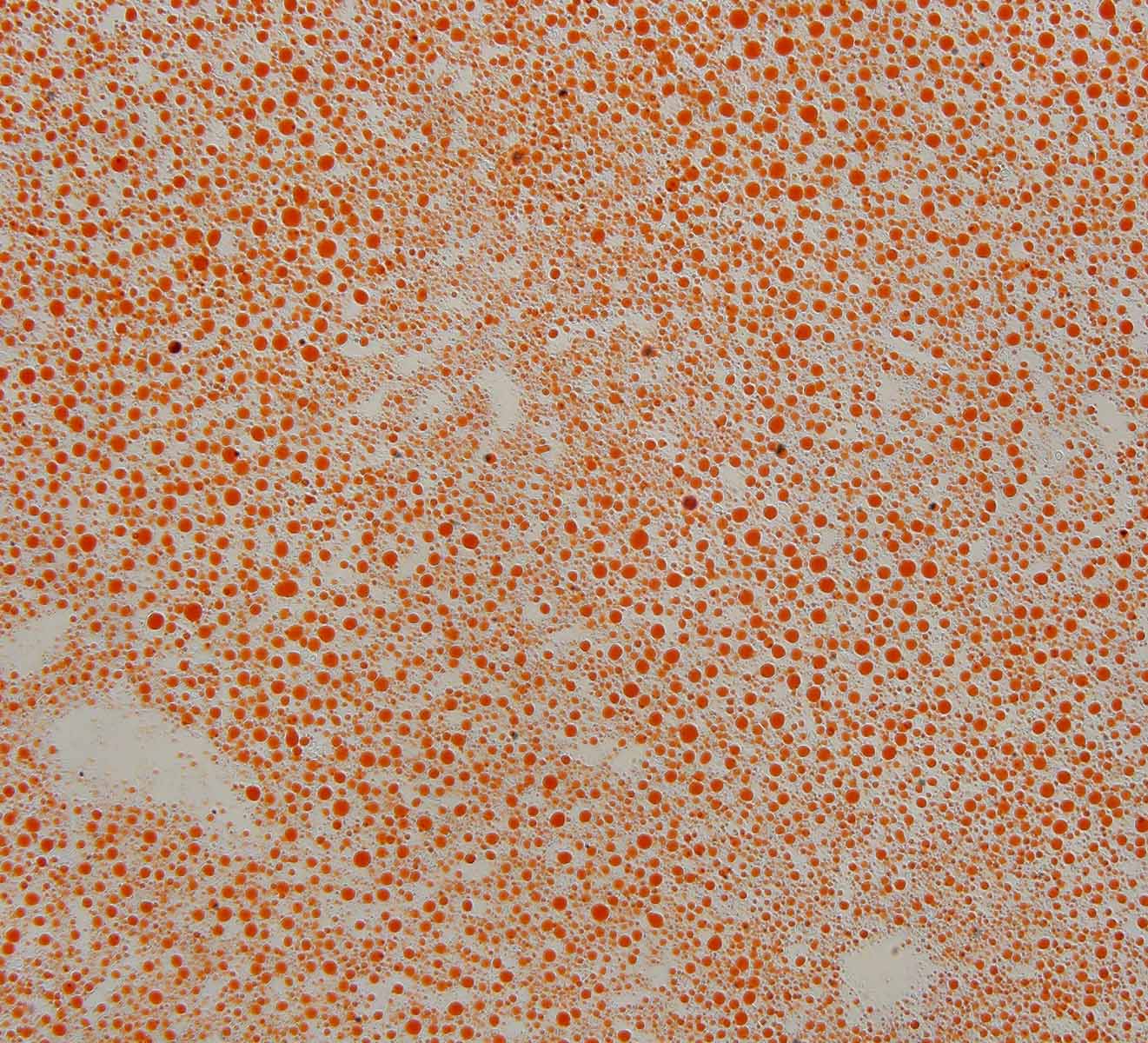

Supplement: Supplementary file 8 — Source Data for Figure 6 [file EMMM-15-e16592-s007.zip › Figure 6/Fig.6J/3.jpg]

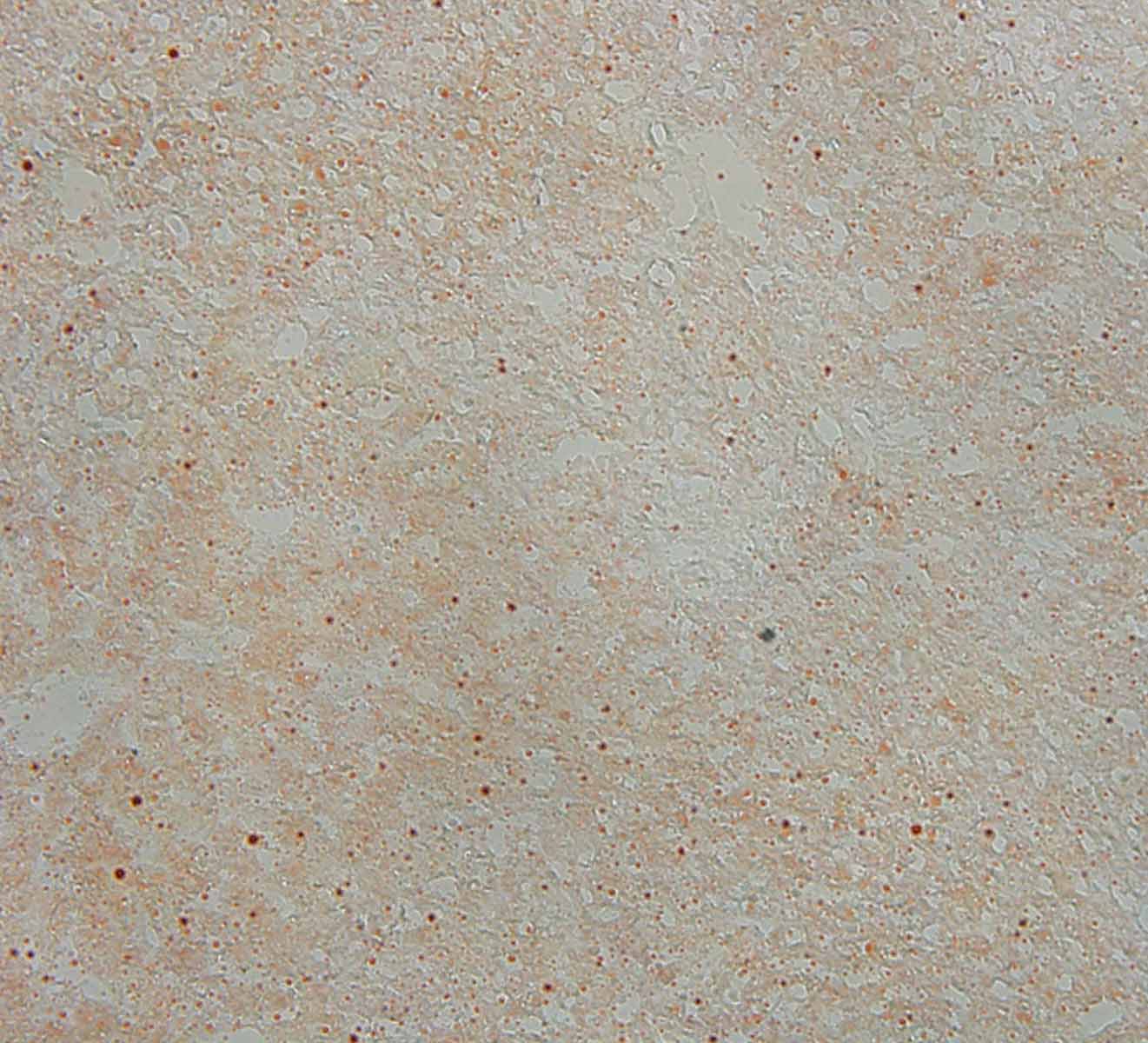

Supplement: Supplementary file 8 — Source Data for Figure 6 [file EMMM-15-e16592-s007.zip › Figure 6/Fig.6J/4.jpg]

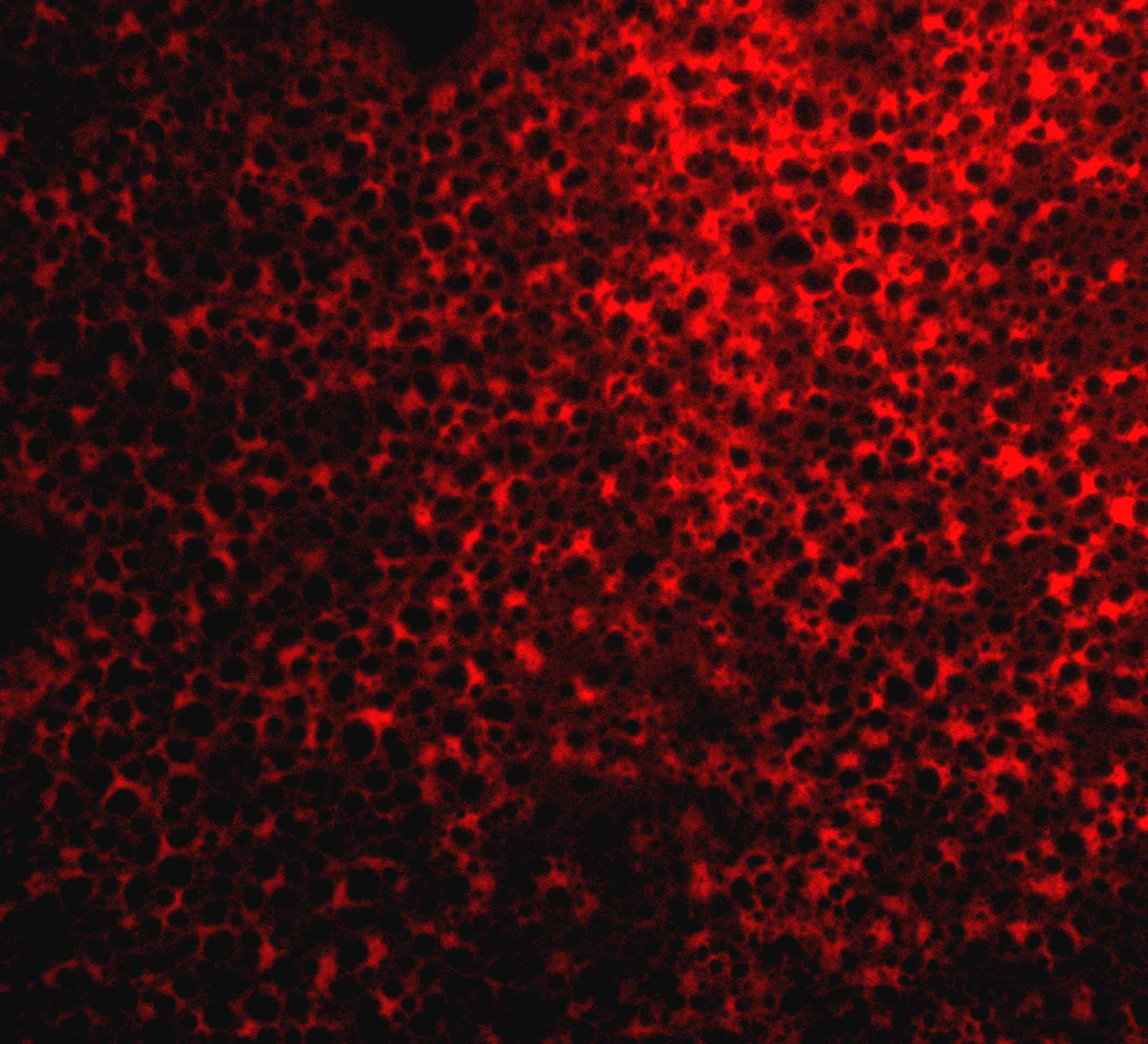

Supplement: Supplementary file 8 — Source Data for Figure 6 [file EMMM-15-e16592-s007.zip › Figure 6/Fig.6J/5.jpg]

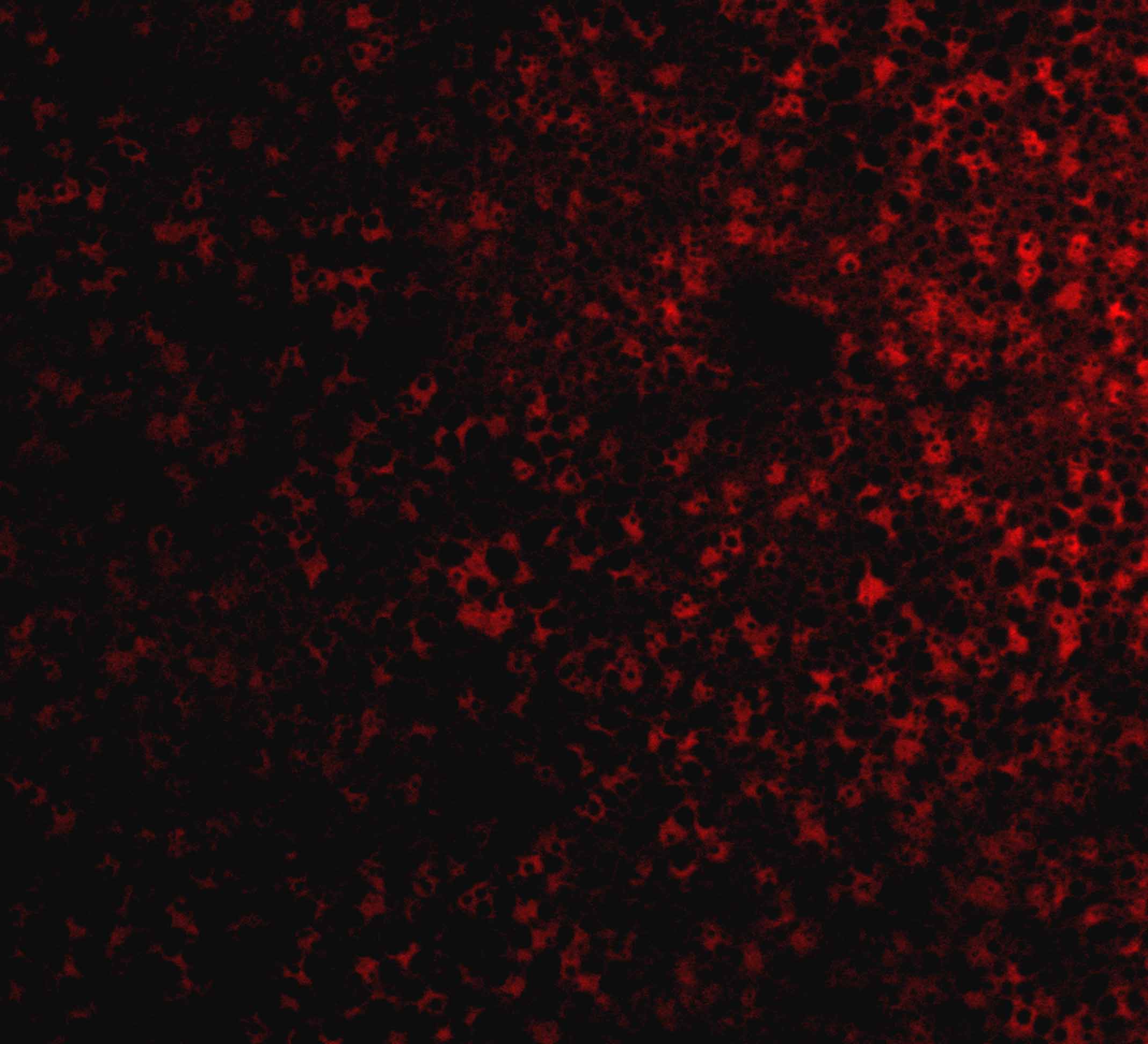

Supplement: Supplementary file 8 — Source Data for Figure 6 [file EMMM-15-e16592-s007.zip › Figure 6/Fig.6J/6.jpg]

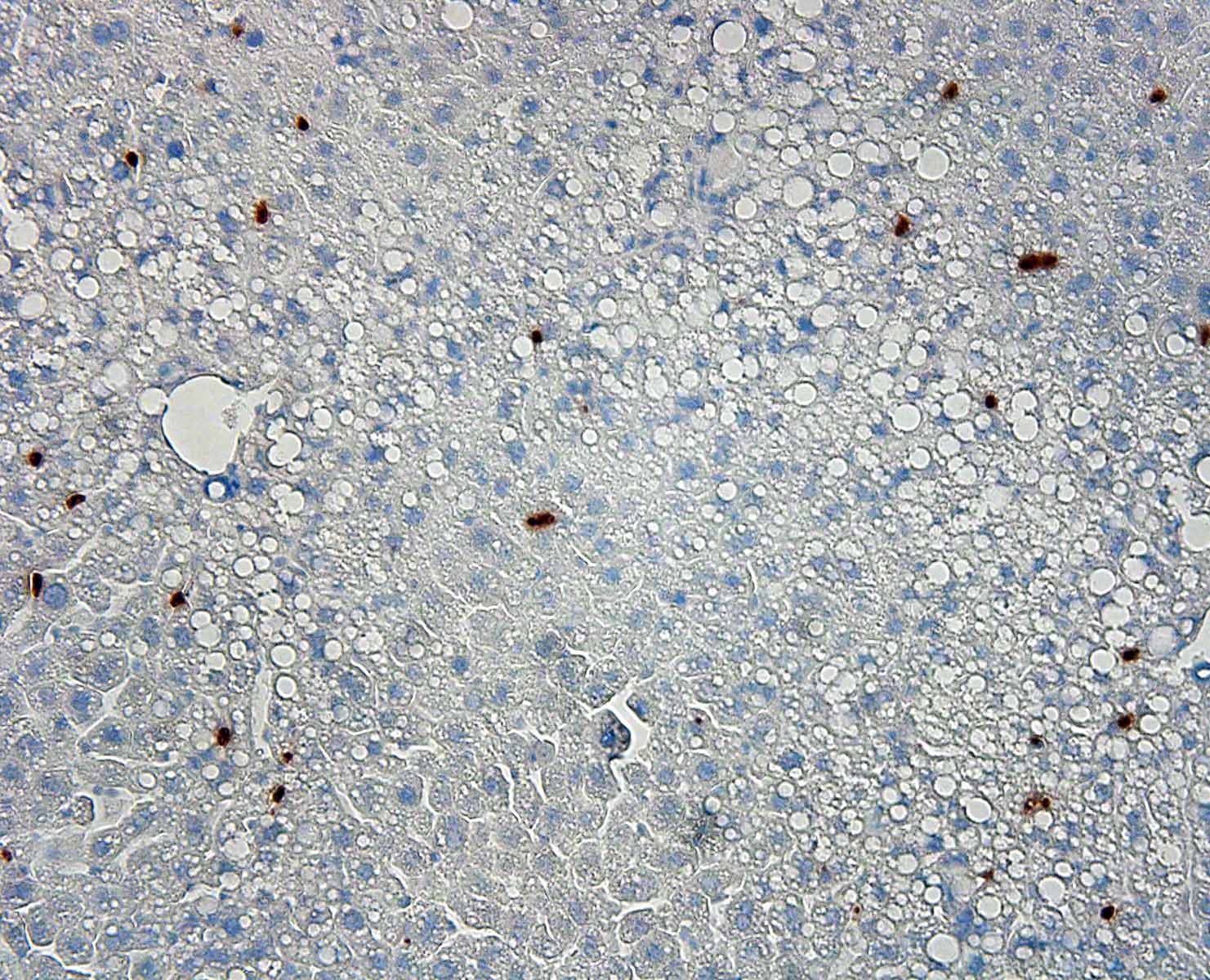

Supplement: Supplementary file 8 — Source Data for Figure 6 [file EMMM-15-e16592-s007.zip › Figure 6/Fig.6J/7.jpg]

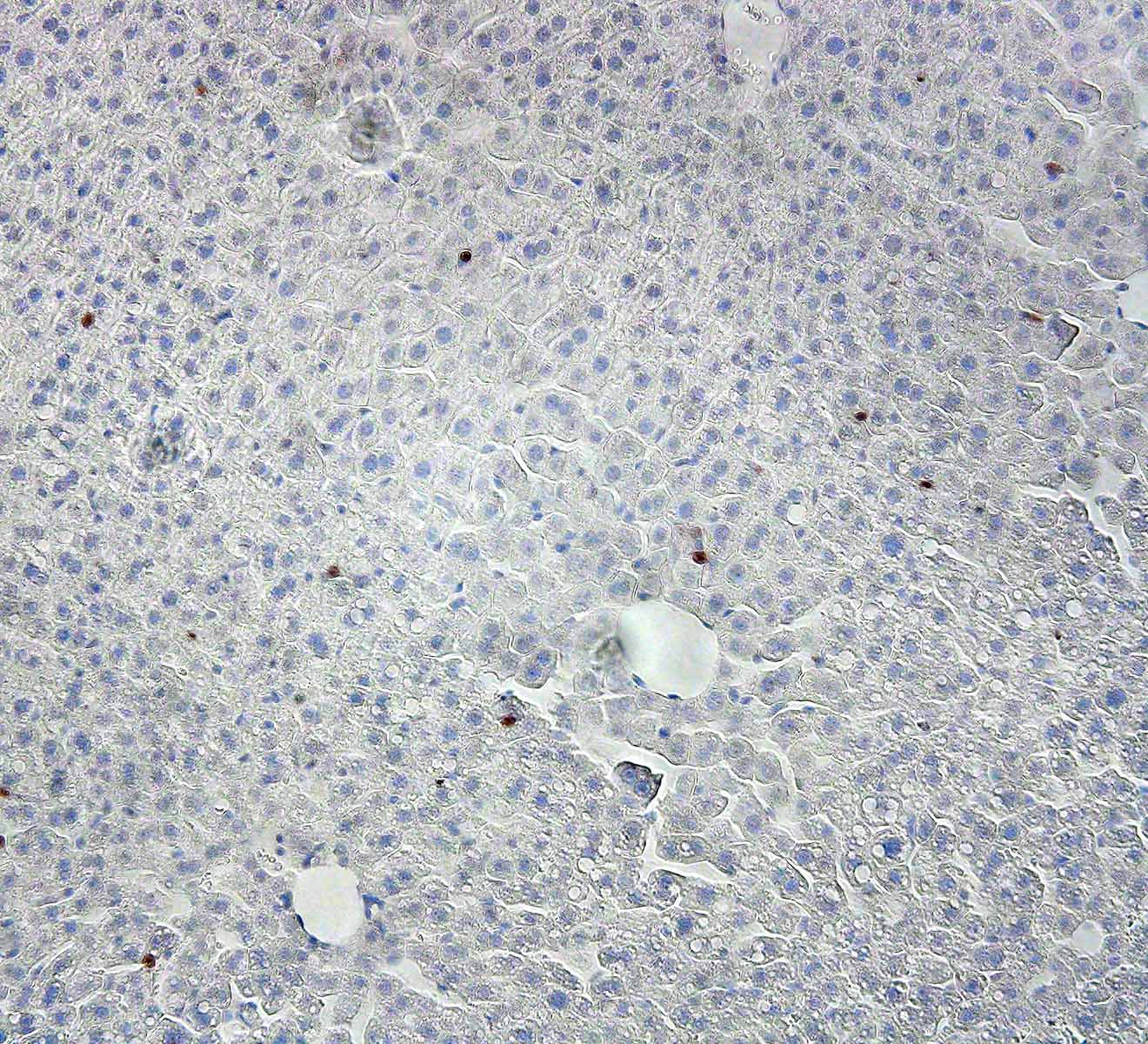

Supplement: Supplementary file 8 — Source Data for Figure 6 [file EMMM-15-e16592-s007.zip › Figure 6/Fig.6J/8.jpg]

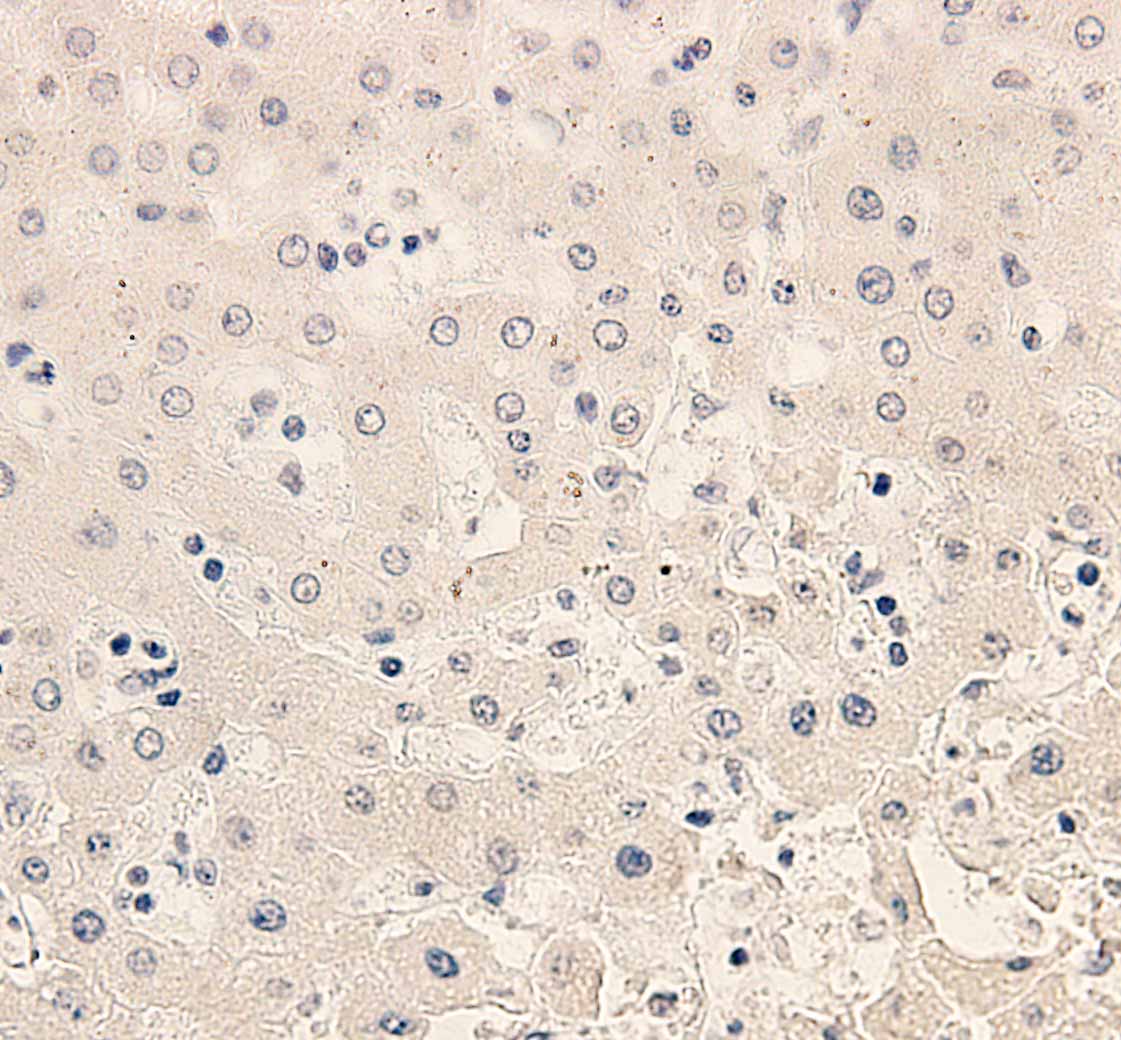

Supplement: Supplementary file 9 — Source Data for Figure 7 [file EMMM-15-e16592-s001.zip › Figure 7/Fig.7C/1.jpg]

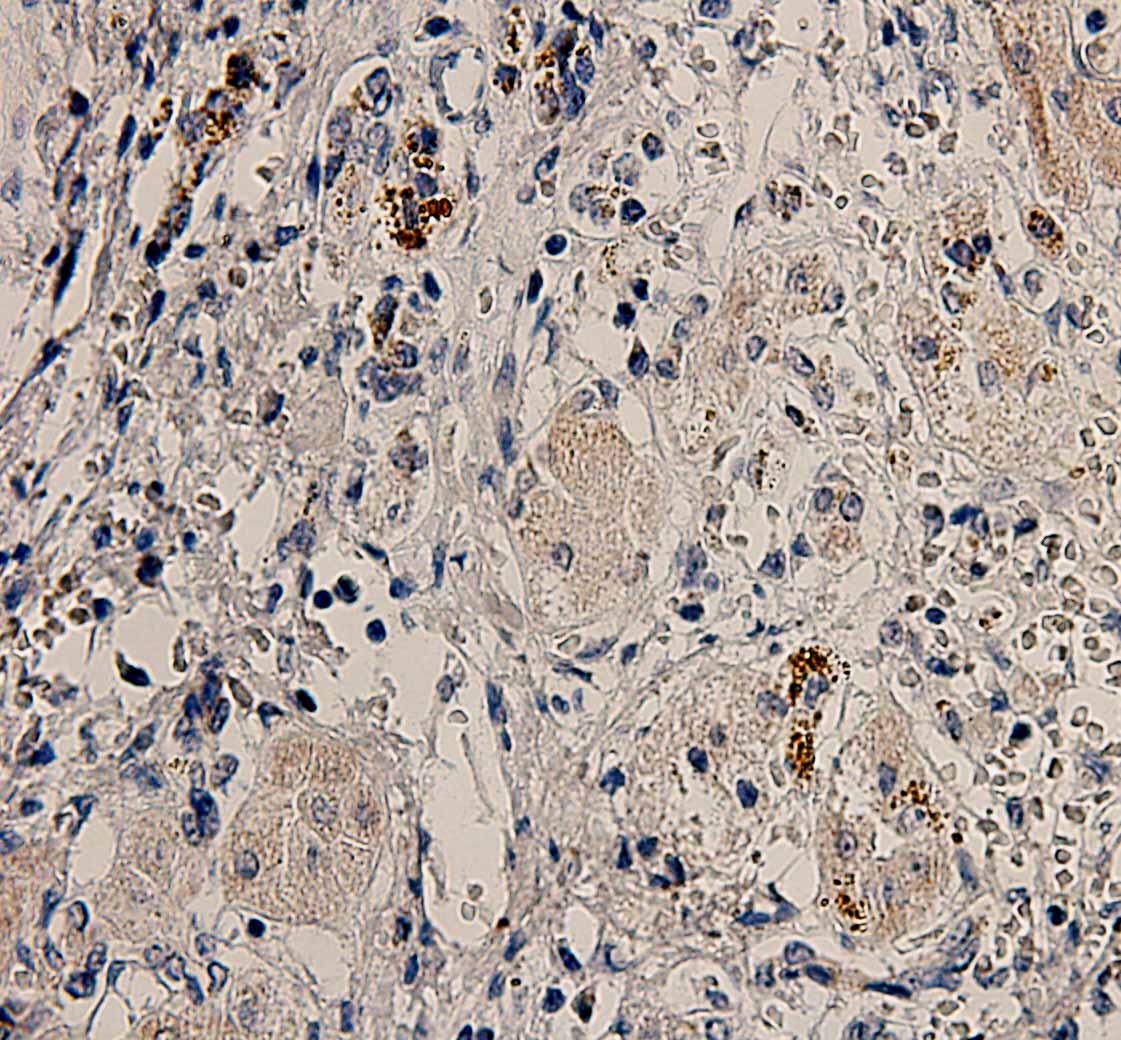

Supplement: Supplementary file 9 — Source Data for Figure 7 [file EMMM-15-e16592-s001.zip › Figure 7/Fig.7C/2.jpg]

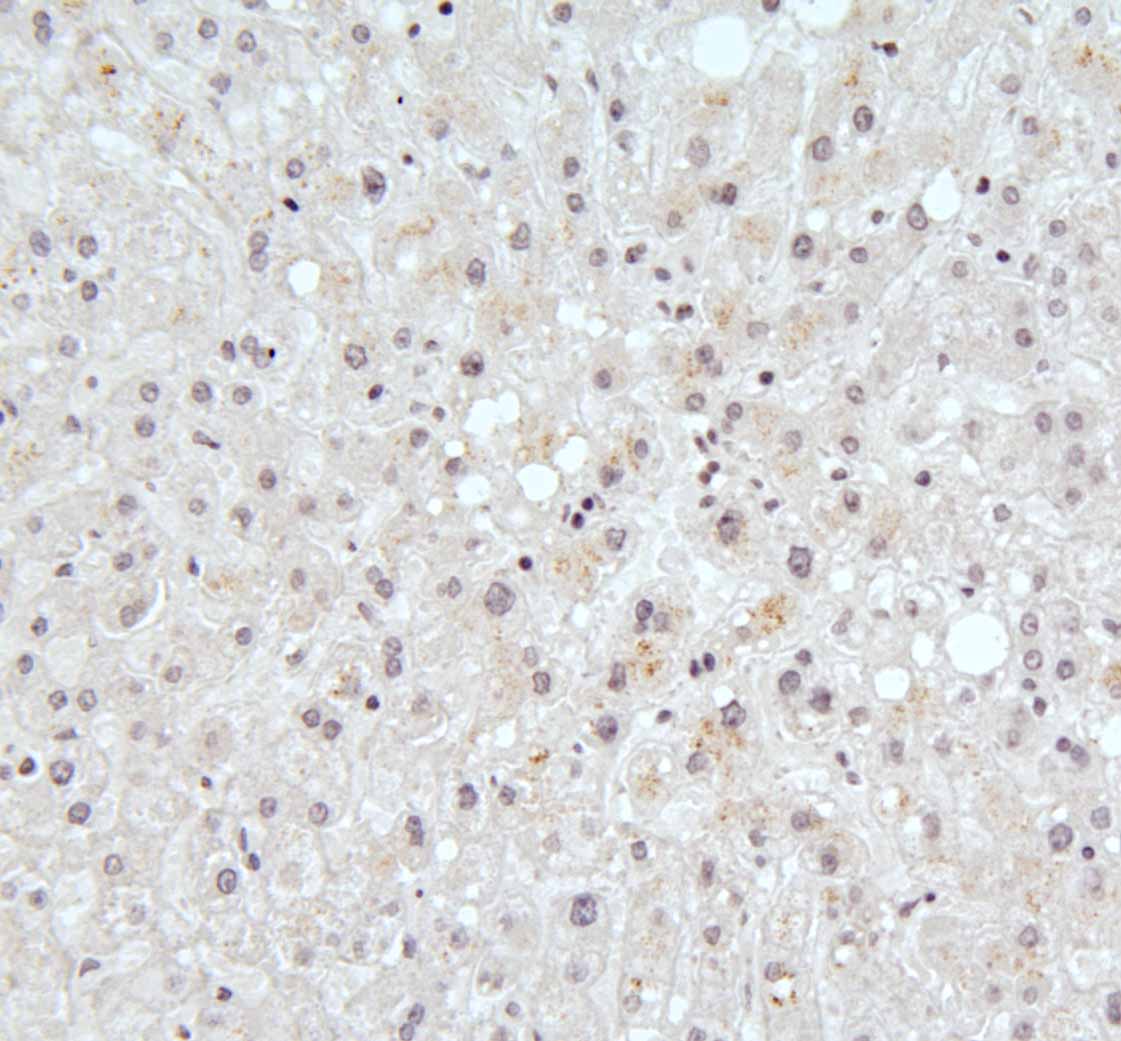

Supplement: Supplementary file 9 — Source Data for Figure 7 [file EMMM-15-e16592-s001.zip › Figure 7/Fig.7C/3.jpg]

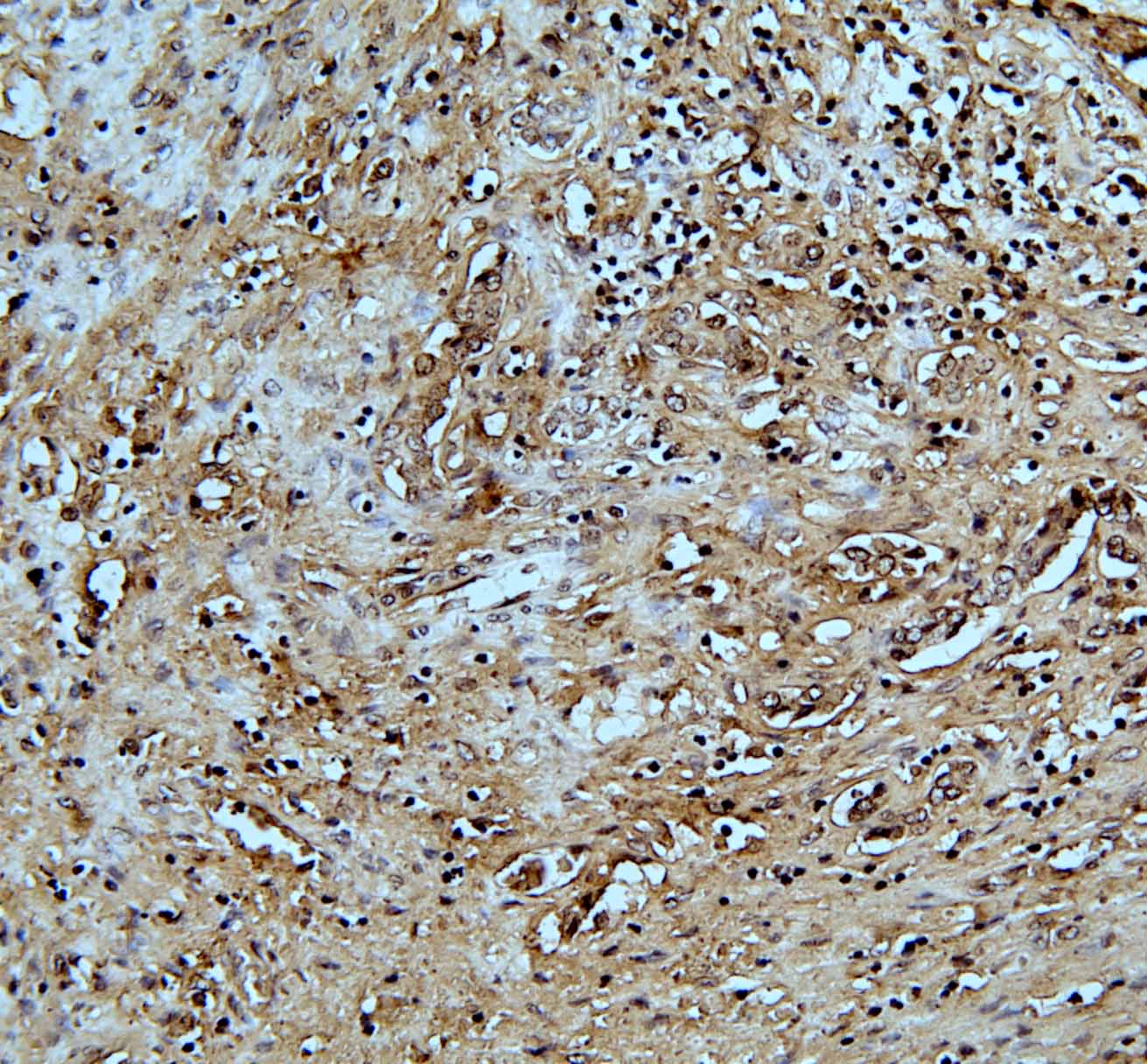

Supplement: Supplementary file 9 — Source Data for Figure 7 [file EMMM-15-e16592-s001.zip › Figure 7/Fig.7C/4.jpg]
