# Supplementary material for: Targetable Brg1‐CXCL14 axis contributes to alcoholic liver injury by driving neutrophil trafficking
Source: EMBO Mol Med. 2023 Feb 1;15(3):e16592. doi: 10.15252/emmm.202216592 (PMC9994483; doi:10.15252/emmm.202216592)
Supplement: Supplementary file 10 [file EMMM-15-e16592-s009.pdf]

EMBO Molecular Medicine

Targetable Brg1-CXCL14 axis contributes to alcoholic liver injury by driving neutrophil trafficking

Nan Li, Hong Liu, Yujia Xue, Zheng Xu, Xiulian Miao, Yan Guo, Zilong Li, Zhiwen Fan, and Yong Xu
DOI: 10.15252/emmm.202216592

Corresponding authors: Yong Xu (yjxu@njmu.edu.cn) , Zilong Li (zllnjmu@gmail.com), Zhiwen Fan (fzwdrumtower@hotmail.com)

Review Timeline:

Submission Date:	16th Jul 22
Editorial Decision:	18th Aug 22
Revision Received:	17th Dec 22
Editorial Decision:	10th Jan 23
Revision Received:	13th Jan 23
Accepted:	17th Jan 23

Editor: Zeljko Durdevic

Transaction Report:

(Note: With the exception of the correction of typographical or spelling errors that could be a source of ambiguity, letters and reports are not edited. Depending on transfer agreements, referee reports obtained elsewhere may or may not be included in this compilation. Referee reports are anonymous unless the Referee chooses to sign their reports.)

18th Aug 2022

Dear Dr. Xu,

Thank you for the submission of your manuscript to EMBO Molecular Medicine. We have now received feedback from the two reviewers who agreed to evaluate your manuscript. As you will see from the reports, both referees recognize potential interest of the study but also raise serious concerns that should be addressed in a major revision.

Further consideration of a revision that addresses reviewers' concerns in full will entail a second round of review. EMBO Molecular Medicine encourages a single round of revision only and therefore, acceptance or rejection of the manuscript will depend on the completeness of your responses included in the next, final version of the manuscript. For this reason, and to save you from any frustrations in the end, I would strongly advise against returning an incomplete revision.

We would welcome the submission of a revised version within three months for further consideration. Please let us know if you require longer to complete the revision.

I look forward to receiving your revised manuscript.

Yours sincerely,

Zeljko Durdevic

Zeljko Durdevic
Editor
EMBO Molecular Medicine

.

***** Reviewer's comments *****

Referee #1 (Comments on Novelty/Model System for Author):

It is okay.

Referee #1 (Remarks for Author):

Brahma-related gene 1 (Brg1) is the core component of the SWI/SNF chromatin remodeling complex, contributing to organ development and disease progression. Alcoholic liver diseases (ALD), including alcoholic steatosis, alcoholic steatohepatitis, alcoholic liver cirrhosis, and alcoholic-associated liver cancers, have caused significant economic burdens. Clarifying the molecular pathogenesis of ALD may identify potential therapy for ALD patients. The authors aimed to investigate the role of Brg1 in ALD pathogenesis and its potential application in treating ALD. Using two ALD mouse models and human ALD samples, they found that Brg1 is upregulated in rodent and human ALD liver tissues compared to the control ones. Besides, alcohol consumption significantly promoted alcoholic steatosis and steatohepatitis in male mice, but liver-specific knockout of Brg1 (Brg1LKO) reversed disease severity. Liver-specific overexpression of Brg1 (Brg1LKI) further exacerbated ALD phenotypes. Flow cytometry analysis found that only neutrophil infiltration is decreased in Brg1LKO mice. Through RNA Seq (n=2), the CXCL14 was identified to be likely involved in the Brg1-mediated decrease of neutrophil infiltration. Indeed, the re-introduction of CXCL14 reversed the impacts of liver Brg1 knockout on ALD development. Overexpression of CXCL14 also promoted ALD progression. More importantly, treating mice with CXCL14 and Brg1 inhibitors significantly alleviated ALD phenotypes. The authors also established the correlation of Brg1, CXCL14, and neutrophils in human ALD samples. Overall, the current study has comprehensively demonstrated the Brg1/CXCL14 axis in ALD progression. However, the impacts of Brg1 in non-alcoholic fatty liver and chronic liver injuries have been extensively studied by others and the author's group, thus rendering limited innovation of this study. Besides, several major concerns identified in this manuscript may dampen the current findings' impacts on the ALD society.

Major:

1. The authors measured cyp2e1 levels and found no difference between control and Brg1LKO mice. There are several enzymes involved in alcohol metabolism. Besides, alcohol consumption in the NIAAA model may significantly impact the study outcomes. Did the authors measure alcohol drinking amount (food consumption) and blood alcohol contents in these mice?
2. All immunohistochemical staining images for immune cells are of poor quality. The specific staining results are not distinguishable, making the authors' statement inconclusive.
3. It is surprising that several important inflammatory cytokines are down-regulated in Brg1LKO mice, but only neutrophil infiltration is decreased in the liver of ALD mice. How could down-regulated inflammatory cytokines, such as TNFα, IL1β, and MCP-1, not lead to reduced macrophages in ALD? Is there any compensated mechanism to promote hepatic Kupffer cells or circulating macrophage infiltration?
4. The neutrophil co-culture experiments were performed using mouse hepatocytes. Can the authors repeat the experiments using human hepatocytes to ensure human relevance? The stained neutrophils look like the background of transwell pores. You may want to replace the representative image with a suitable one. In the meantime, can the authors verify if the knockdown of Brg1 or CXCL14 will not cause the infiltration of other immune cell types to support their conclusion?
5. It is interesting to know that Brg1LKO could suppress alcoholic steatosis. What is the potential molecular mechanism by which down-regulated Brg1 suppresses alcoholic steatosis?
6. They should measure mouse body, fat, and liver weight changes to further illustrate how Brg1 impacts ALD progression. Without the information, it may not be able to conclude how Brg1 affects alcoholic steatosis.
Minor: 1. On page 6, they mentioned the RNA seq experiments were done using two Brg1LKI samples. Are they Brg1LKO or Brg1LKI samples?

2. The authors performed RNA Seq experiments using 2 samples per group. Is it statistically significant?
3. Did the authors test if knockout or overexpression of Brg1 affects ALD phenotypes in the chronic alcohol feeding model rather than the NIAAA model?

Referee #2 (Remarks for Author):

The authors investigated an involvement of Brahma-related gene 1 (Brg1) in animal models of alcoholic liver disease (ALD) and in ALD patients. The up- or down-regulation of Brg1 and small molecule inhibitors modulated liver injury and neutrophil recruitment through the chemokine CXCL14. The authors present a lot of supporting evidence for their hypothesis and conclusions. Several issues need to be clarified:

1. The authors compare ALD patients with decades of drinking to naïve animals exposed for a short time to alcohol producing an acute injury. The authors need to better emphasize that these are totally different stages in the pathophysiology of ALD. It may be possible to protect in the animal model by targeting Brg1 and CXCL14 during the development of the injury, it is unlikely that such an intervention will affect ALD in patients with cirrhosis.
2. P. 8, the discussion of the patient samples refers repeatedly to Figure 4B,C and D. However, this should be Figure 7. In addition, it would be helpful to show a higher magnification of Figure 7C (neutrophils) and an immunohistochemistry for CXCL14 staining.
3. The authors report ALT/AST activities in U/dL. The numbers of 150-250 U/dL appear very high for these models as well as in the patients (Table I) as this would translate to 1,500 - 2,000 U/L, which means that there should be extensive necrosis. This seems very unlikely. Thus, the authors need to re-calculate their ALT/AST values.
4. Figure 4G: Treatment with recombinant CXCL14 enhances hepatic PMN infiltration. Does it also increase injury? What are the ALT values for these experiments?
5. Figure 5E: If CXCL14 is a chemotactic factor for neutrophils and responsible for hepatic neutrophil recruitment, why are there no neutrophils in the CXCL14 gene overexpressing animals in the absence of alcohol treatment? Is there no CXCL14 formation in the absence of alcohol? The CXCL14 levels need to be measured to exclude that other factors also contribute to this effect.
6. Figure 5 and others: What is the connection of CXCL14-mediated neutrophil chemotaxis and steatosis? Again, there seem to be potential off-target effects that need to be considered.
7. Figure 5E: The authors show evidence for ROS formation by histology. Where is this ROS coming from? Is there an overlap between neutrophil staining and ROS to suggest that neutrophils may be the source of ROS?
8. Figure 6: The authors claim to use "a specific antagonist to the CXCL14 receptor (AMD3100)". However, AMD3100 is an antagonist of CXCR4, which is known to be the receptor for CXCL12. At the present time, the receptor for CXCL14 has not been definitively identified. Given the effect of AMD3100, is there a role of CXCL12 in addition to CXCL14?
9. Overall, it would be helpful to provide a graphical abstract to better illustrate the mechanisms investigated in this manuscript.

Referee #1 (Remarks for Author):

Brahma-related gene 1 (Brg1) is the core component of the SWI/SNF chromatin remodeling complex, contributing to organ development and disease progression. Alcoholic liver diseases (ALD), including alcoholic steatosis, alcoholic steatohepatitis, alcoholic liver cirrhosis, and alcoholic-associated liver cancers, have caused significant economic burdens. Clarifying the molecular pathogenesis of ALD may identify potential therapy for ALD patients. The authors aimed to investigate the role of Brg1 in ALD pathogenesis and its potential application in treating ALD. Using two ALD mouse models and human ALD samples, they found that Brg1 is upregulated in rodent and human ALD liver tissues compared to the control ones. Besides, alcohol consumption significantly promoted alcoholic steatosis and steatohepatitis in male mice, but liver-specific knockout of Brg1 (Brg1LKO) reversed disease severity. Liver-specific overexpression of Brg1 (Brg1LKI) further exacerbated ALD phenotypes. Flow cytometry analysis found that only neutrophil infiltration is decreased in Brg1LKO mice. Through RNA Seq (n=2), the CXCL14 was identified to be likely involved in the Brg1-mediated decrease of neutrophil infiltration. Indeed, the re-introduction of CXCL14 reversed the impacts of liver Brg1 knockout on ALD development. Overexpression of CXCL14 also promoted ALD progression. More importantly, treating mice with CXCL14 and Brg1 inhibitors significantly alleviated ALD phenotypes. The authors also established the correlation of Brg1, CXCL14, and neutrophils in human ALD samples.
Overall, the current study has comprehensively demonstrated the Brg1/CXCL14 axis in ALD progression. However, the impacts of Brg1 in non-alcoholic fatty liver and chronic liver injuries have been extensively studied by others and the author's group, thus rendering limited innovation of this study. Besides, several major concerns identified in this manuscript may dampen the current findings' impacts on the ALD society.
We thank the reviewer for his/her insightful critiques and helpful suggestions to improve our manuscript. Please see below our point-by-point response.

Major:
1. The authors measured cyp2e1 levels and found no difference between control and Brg1LKO mice. There are several enzymes involved in alcohol metabolism. Besides, alcohol consumption in the NIAAA model may significantly impact the study outcomes. Did the authors measure alcohol drinking amount (food consumption) and blood alcohol contents in these mice?
Response:
(1) We have examined the levels of other enzymes involved in alcohol metabolism including Adh1, Aldh2, Aldh3a, and Aldh9a. As shown in the new appendix supplementary figure 5C, none of these enzymes were altered by Brg1 deletion in hepatocytes.
(2) Alcohol intake (measured daily) and blood alcohol concentration (measured at the time of sacrifice) have been included as the new appendix supplementary figure 4A and 4B, respectively. No differences were detected for either parameter between the WT mice and the Brg1 LKO mice.

2. All immunohistochemical staining images for immune cells are of poor quality. The specific staining results are not distinguishable, making the authors' statement inconclusive.

Response: We appreciate the reviewer's concern. We have now provided enlarged images for all the Ly6G IHC stainings.

3. It is surprising that several important inflammatory cytokines are down-regulated in Brg1LKO mice, but only neutrophil infiltration is decreased in the liver of ALD mice. How could down-regulated inflammatory cytokines, such as TNFα, IL1β, and MCP-1, not lead to reduced macrophages in ALD? Is there any compensated mechanism to promote hepatic Kupffer cells or circulating macrophage infiltration?

Response: We thank the reviewer for raising this intriguing question. There are several possible interpretations for this apparent discrepancy. We agree with the reviewer that there might be a compensatory mechanism in the absence of hepatocyte Brg1 to promote macrophage infiltration despite low levels of chemokines. Alternatively, macrophage trafficking is regulated by not one but several different chemokines such that a threshold must be reached before discernable changes in cell migration could be detected. This "barcode" of chemokines has yet to be determined for macrophages in the context of ALD. Thirdly, an early surge followed by a decline of F4/80+ macrophages has been well documented for a wide range of organ injuries. We did not profile the dynamic changes in macrophage population in the liver during the entire course of ALD pathogenesis using the NIAAA protocol but instead focused on the end-point for analysis. Therefore, the possibility that Brg1 may contribute to regulate macrophage infiltration at earlier points during ALD development can not be conclusively excluded. Lastly, whole liver tissue was used for RNA extraction and qPCR analysis. Therefore, the data presented in figure 2E should be interpreted as overall inflammation in the liver instead of "chemoattractive prowess" emitting from hepatocytes. After all, many of these cytokines/chemokines can be produced by neutrophils (PMID: 25374568, 29772063). Down-regulation of these cytokines/chemokines may be interpreted as deficient neutrophil recruitment. The reviewer is absolutely right that the role of Brg1 in regulating immune cell trafficking in the context of ALD pathogenesis deserves more attention and we hope to address these lingering issues in future studies. We have expanded the Discussion section to reflect on this point.

4. The neutrophil co-culture experiments were performed using mouse hepatocytes. Can the authors repeat the experiments using human hepatocytes to ensure human relevance? The stained neutrophils look like the background of transwell pores. You may want to replace the representative image with a suitable one. In the meantime, can the authors verify if the knockdown of Brg1 or CXCL14 will not cause the infiltration of other immune cell types to support their conclusion?

Response: We thank the reviewer for his/her suggestion.

(1) We have since performed additional Boyden chamber trans-well experiments using HepaRG cells, considered ideal for investigating liver metabolism in lieu of primary human hepatocytes (PMID: 20645056). As shown in the new appendix supplementary figure 8,

figure 17, and figure 18, Brg1 knockdown, or Brg1 inhibition, or CXCL14 antagonism attenuated neutrophil migration.
(2) We respectfully refer the reviewer to the appendix supplementary figure 7 showing that Brg1 deficiency did not influence the infiltration of either macrophages or T lymphocytes. We have also performed additional IHC stainings to assess the effect of CXCL14 on macrophage/T cell infiltration in the liver. As shown in the new appendix supplementary figure 16, CXCL14 knockdown did not alter the infiltration of macrophages or T lymphocytes.

5. It is interesting to know that $Brg1^{LKO}$ could suppress alcoholic steatosis. What is the potential molecular mechanism by which down-regulated Brg1 suppresses alcoholic steatosis?
Response: We thank the reviewer for raising this critical question. There are several possible mechanisms, both direct and indirect, whereby Brg1 in hepatocytes may contribute to steatosis, some of which have been alluded to in the Discussion section. Based on our prior observations that Brg1 interacts with SREBPs to promote de novo lipogenesis in hepatocytes, we speculate that Brg1 deficiency may dampen SREBP activity and therefore directly influence lipid accumulation in the liver. Alternatively, dampened inflammation as a result of Brg1-dependent recruitment of neutrophils may alter the immune microenvironment in the liver thereby skewing the hepatic metabolism and lipid accumulation. Of note, some of the cytokines/chemokines could directly act on hepatocytes to alter metabolism. For instance, Clement et al have previously reported that adipose tissue derived MCP-1, a classic chemokine, can induce lipid accumulation when added to hepatocytes in vitro (PMID: 18570214).

6. They should measure mouse body, fat, and liver weight changes to further illustrate how Brg1 impacts ALD progression. Without the information, it may not be able to conclude how Brg1 affects alcoholic steatosis.
Response: We appreciate the reviewer's concern. As requested, body weight, liver weight, and gonadal WAT weight have been measured. As shown in the new appendix supplementary figure 3, Brg1 deficiency attenuated liver weight and liver weight/body weight ratio without influencing body weight, WAT weight, or WAT weight/body weight ratio in mice.

Minor:
1. On page 6, they mentioned the RNA seq experiments were done using two Brg1LKI samples. Are they Brg1LKO or Brg1LKI samples?
Response: This was a typo and has been corrected. $Brg1^{LKO}$ livers were used for RNA-seq. We apologize for the confusion.

2. The authors performed RNA Seq experiments using 2 samples per group. Is it statistically significant?
Response: We thank the reviewer for his/her comment. Because 2 samples were used for each group, statistical analysis was not performed. We would like to add that the differentially expressed genes including CXCL14 have been validated by qPCR.

3. Did the authors test if knockout or overexpression of Brg1 affects ALD phenotypes in the chronic alcohol feeding model rather than the NIAAA model?

Response: We thank the reviewer for his/her suggestion. We have not established a chronic alcohol feeding model in our laboratory. Both the NIAAA model and the chronic alcohol feeding model have pros and cons in terms of recapitulating the human ALD pathology; neither can faithfully encapsulate the entire spectrum of ALD development and progression in humans. We have added a few sentences in the Discussion section to reflect on the limitation of our study.

Referee #2 (Remarks for Author):

The authors investigated an involvement of Brahma-related gene 1 (Brg1) in animal models of alcoholic liver disease (ALD) and in ALD patients. The up- or down-regulation of Brg1 and small molecule inhibitors modulated liver injury and neutrophil recruitment through the chemokine CXCL14. The authors present a lot of supporting evidence for their hypothesis and conclusions. Several issues need to be clarified:

We thank the reviewer for his/her insightful critiques and helpful suggestions to improve our manuscript. Please see below our point-by-point response.

1. The authors compare ALD patients with decades of drinking to naïve animals exposed for a short time to alcohol producing an acute injury. The authors need to better emphasize that these are totally different stages in the pathophysiology of ALD. It may be possible to protect in the animal model by targeting Brg1 and CXCL14 during the development of the injury, it is unlikely that such an intervention will affect ALD in patients with cirrhosis.

Response: We thank the reviewer for his/her critique. We agree with the reviewer that ALD develop over a much longer time than and thus cannot be faithfully recapitulated by the current model. This limitation is now discussed in the Discussion section.

2. P. 8, the discussion of the patient samples refers repeatedly to Figure 4B, C and D. However, this should be Figure 7. In addition, it would be helpful to show a higher magnification of Figure 7C (neutrophils) and an immunohistochemistry for CXCL14 staining.

Response:

(1) The referral to Figure 4B, C, and D should have been to Figure 7B, C, and D. This has been corrected and we apologize for the confusion.

(2) A magnified field of neutrophil staining is now included for Figure 7C.

(3) CXCL14 staining is now included in the revised Figure 7C.

3. The authors report ALT/AST activities in U/dL. The numbers of 150-250 U/dL appear very high for these models as well as in the patients (Table I) as this would translate to 1,500 - 2,000 U/L, which means that there should be extensive necrosis. This seems very unlikely. Thus, the authors need to re-calculate their ALT/AST values.

Response: We thank the reviewer for his/her comment. This indeed was a mislabeling. The unit should have been U/L instead of U/dL. The labels have been corrected throughout the manuscript. We apologize for the confusion.

4. Figure 4G: Treatment with recombinant CXCL14 enhances hepatic PMN infiltration. Does it also increase injury? What are the ALT values for these experiments?

Response: We thank the reviewer for his/her comment. We want to clarify that the aim of this experiment was to add recombinant CXCL14 as a supplement for the decrease of endogenous CXCL14 as a result of Brg1 deletion. It was not intended for as a "treatment" *per se*. As

shown below in the Reviewer Figure 1, the addition of recombinant CXCL14 did not alter ALT levels.

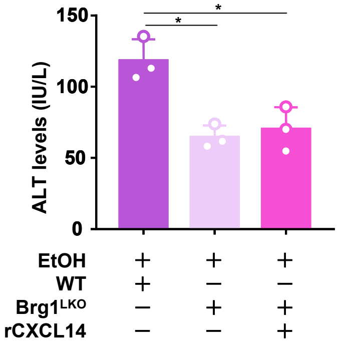

Reviewer Figure 1: Primary hepatocytes isolated from WT and Brg1 LKO mice were exposed to ethanol (50mM) in the presence or absence of recombinant CXCL14. ALT levels in the culture media were measured by a colorimetric kit.

5. Figure 5E: If CXCL14 is a chemotactic factor for neutrophils and responsible for hepatic neutrophil recruitment, why are there no neutrophils in the CXCL14 gene overexpressing animals in the absence of alcohol treatment? Is there no CXCL14 formation in the absence of alcohol? The CXCL14 levels need to be measured to exclude that other factors also contribute to this effect.

Response: We appreciate the reviewer's concern. One possible interpretation for the Ly6G staining shown in Figure 5E is that CXCL14 functions as an essential cue for neutrophil recruitment under pathological conditions (e.g., excessive alcohol intake) but may be insufficient when acting alone under physiological conditions even with large quantities. We have measured the efficiency of CXCL14 over-expression under physiological conditions. As shown in the modified appendix supplementary figure 14, the efficiency of CXCL14 over-expression appears to be similar in the pair-fed mice and the NIAAA-fed mice.

6. Figure 5 and others: What is the connection of CXCL14-mediated neutrophil chemotaxis and steatosis? Again, there seem to be potential off-target effects that need to be considered.

Response: We appreciate the reviewer's concern. Increased neutrophil infiltration in the liver may promote steatosis by producing reactive oxygen species and pro-inflammatory mediators to alter metabolism in hepatocytes. Neutrophil depletion by anti-Ly6G antibody has been shown to attenuate both hepatic inflammation AND steatosis in a model of non-alcoholic fatty liver disease (PMID: 26843485). A similar strategy has been exploited by Szabo et al in a model of binge alcohol intake to show that neutrophil depletion alleviates liver inflammation and injury; it is not clear whether steatosis was altered in this model (PMID: 30030149). A bolder but not unfounded hypothesis would be that CXCL14 may act on hepatocytes directly to skew metabolism. Clement et al have previously reported that adipose tissue derived MCP-1, a classic chemokine, can induce lipid accumulation when added to hepatocytes in vitro (PMID: 18570214). Therefore, while we respectfully disagree with the reviewer that the observation that CXCL14 appears to simultaneously regulate neutrophil trafficking and

steatosis may be off-target it needs further analysis in future studies. We have expanded the Discussion section to reflect on this point.

7. Figure 5E: The authors show evidence for ROS formation by histology. Where is this ROS coming from? Is there an overlap between neutrophil staining and ROS to suggest that neutrophils may be the source of ROS?

Response: We thank the reviewer for his/her intriguing question. Per his/her suggestion, we have performed double fluorescence staining with DHE (to detect ROS) and anti-Ly6G (to detect neutrophil). As shown below in the Reviewer Figure 2, there is some overlapping of DHE staining and Ly6G staining. We can only conclude, based on this information, that neutrophils can produce ROS in the liver. However, it remains to be determined whether neutrophils are the sole or even primary source of intrahepatic ROS in the course of ALD. Although our data seem to suggest that there might be a positive correlation between neutrophil infiltration and intrahepatic ROS levels, the effect may or may not be direct. The reviewer would probably agree that this issue, while certainly germane, is a little bit out of the scope of the present study, which we hope to address in future studies.

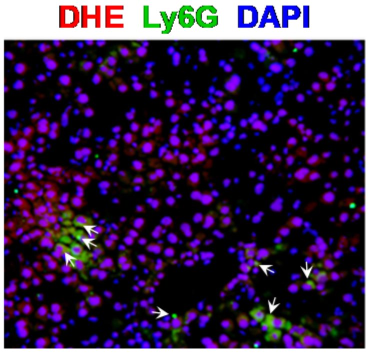

Reviewer Figure 2: C57/B6 mice were fed a NIAAA diet and cryosections were stained with anti-Ly6G and DHE.

8. Figure 6: The authors claim to use "a specific antagonist to the CXCL14 receptor (AMD3100)". However, AMD3100 is an antagonist of CXCR4, which is known to be the receptor for CXCL12. At the present time, the receptor for CXCL14 has not been definitively identified. Given the effect of AMD3100, is there a role of CXCL12 in addition to CXCL14?

Response: We thank the reviewer for his/her comment. We have examined the involvement of CXCL12 in macrophage migration. As shown in the new appendix supplementary Figure 19, depletion of CXCL12 in primary hepatocytes did not alter macrophage migration when co-cultured.

9. Overall, it would be helpful to provide a graphical abstract to better illustrate the mechanisms investigated in this manuscript.

Response: We thank the reviewer for his/her suggestion. A schematic diagram is now presented as the new Figure 7E to illustrate the mechanisms investigated in this manuscript.

10th Jan 2023

Dear Dr. Xu,

Thank you for the submission of your revised manuscript to EMBO Molecular Medicine. I am pleased to inform you that we will be able to accept your manuscript pending the following final amendments:

1) Please address the referee #1 points by discussion in the manuscript text.
2) Authors: E-mail correspondence to Zheng Xu and Nan Li could not be delivered. Please update their e-mail addresses and make sure to enter correct e-mail addresses for all authors in our submission system. Also, we note that you currently have together with you, a total of 3 co-corresponding authors. Is that correct? Do you confirm equal contribution of these 3 people, able to take full responsibility for the paper and its content? While there is no limit per se to the number of co-corresponding authors, 3 is rare and may not reflect as intended to the community.
3) Please carefully check and submit a complete Author Checklist. https://www.embopress.org/pb-assets/embo-site/EMBO%20Press%20Author%20Checklist-1642513524327.xlsx
4) Figures: Please add zoom highlight boxes to the enlarged image in all respective figures e.g. Figure 1D, H and K, Figure 3B, Figure 5E and J, Figure 6E and J and to several Appendix figures.
5) In the main manuscript file, please do the following:
- Correct/answer the track changes suggested by our data editors by working from the attached document.
- Add callouts for Figure 5K and 7E.
- In M&M, please include statement that in addition to WMA Declaration of Helsinki the experiments also conformed the Department of Health and Human Services Belmont Report.
- In M&M, provide the antibody dilutions that were used for each antibody.
- Please rename "Competing Interest" to "Disclosure Statement & Competing Interests". We updated our journal's competing interests policy in January 2022 and request authors to consider both actual and perceived competing interests. Please review the policy https://www.embopress.org/competing-interests and update your competing interests if necessary.
- Author contributions: Please remove it from the manuscript and specify author contributions in our submission system. CRediT has replaced the traditional author contributions section because it offers a systematic machine-readable author contributions format that allows for more effective research assessment. You are encouraged to use the free text boxes beneath each contributing author's name to add specific details on the author's contribution. More information is available in our guide to authors:
https://www.embopress.org/page/journal/17574684/authorguide#authorshipguidelines
- In data availability statement in addition to the accession number please provide URL for the deposited dataset in this case in NCBI (not PubMed). Use the following format to report the accession number of your data:

The datasets produced in this study are available in the following databases:
[data type]: [full name of the resource] [accession number/identifier] ([doi or URL or identifiers.org/DATABASE:ACCESSION])

Please check "Author Guidelines" for more information.
https://www.embopress.org/page/journal/17574684/authorguide#availabilityofpublishedmaterial
6) Appendix: Please upload it as .pdf file. All figures and tables should be listed separatly in the Table of Content. Please rename all supplementary figures and tables to "Appendix Figure S1" etc and "Appendix Table S1" etc and update their callouts in the main manuscript text.
7) Funding. Please make sure that information about all sources of funding are complete in both our submission system and in the manuscript.
8) The Paper Explained: Please provide "The Paper Explained" and add it to the main manuscript text. "The Paper Explained" consists of 3 sections: Problem, Results, and Impact. Please refer to any recently published manuscript on our website and please check "Author Guidelines" for more information.
https://www.embopress.org/page/journal/17574684/authorguide#researcharticleguide
9) Synopsis: Every published paper now includes a 'Synopsis' to further enhance discoverability. Synopses are displayed on the journal webpage and are freely accessible to all readers. They include separate synopsis image and synopsis text.
- Synopsis image: Please provide a striking image or visual abstract as a high-resolution jpeg file 550 px-wide x (250-400)-px high to illustrate your article.
- Synopsis text: Please provide a short standfirst (maximum of 300 characters, including space) as well as 2-5 one sentence bullet points that summarise the paper as a .doc file. Please write the bullet points to summarise the key NEW findings. They should be designed to be complementary to the abstract - i.e. not repeat the same text. We encourage inclusion of key acronyms and quantitative information (maximum of 30 words / bullet point). Please use the passive voice.
- Please check your synopsis text and image before submission with your revised manuscript. Please be aware that in the proof stage minor corrections only are allowed (e.g., typos).
10) For more information: This space should be used to list relevant web links for further consultation by our readers. Could you identify some relevant ones and provide such information as well? Some examples are patient associations, relevant databases, OMIM/proteins/genes links, author's websites, etc...

11) Source data: Please upload one file per figure together with completed checklist.
12) As part of the EMBO Publications transparent editorial process initiative (see our Editorial at http://embomolmed.embopress.org/content/2/9/329), EMBO Molecular Medicine will publish online a Review Process File (RPF) to accompany accepted manuscripts. This file will be published in conjunction with your paper and will include the anonymous referee reports, your point-by-point response and all pertinent correspondence relating to the manuscript. Let us know whether you agree with the publication of the RPF and as here, if you want to remove or not any figures from it prior to publication. Please note that the Authors checklist will be published at the end of the RPF.
13) Please provide a point-by-point letter INCLUDING my comments as well as the reviewer's reports and your detailed responses (as Word file).

I look forward to reading a new revised version of your manuscript as soon as possible.

Yours sincerely,

Zeljko Durdevic

Zeljko Durdevic
Editor
EMBO Molecular Medicine

***** Reviewer's comments *****

Referee #1 (Remarks for Author):

3.It is surprising that several important inflammatory cytokines are down-regulated in Brg1LKO mice, but only neutrophil infiltration is decreased in the liver of ALD mice. How could down-regulated inflammatory cytokines, such as TNFα, IL1β, and MCP-1, not lead to reduced macrophages in ALD? Is there any compensated mechanism to promote hepatic Kupffer cells or circulating macrophage infiltration?
Response: We thank the reviewer for raising this intriguing question. There are several possible interpretations for this apparent

discrepancy. We agree with the reviewer that there might be a compensatory mechanism in the absence of hepatocyte Brg1 to promote macrophage infiltration despite low levels of chemokines. Alternatively, macrophage trafficking is regulated by not one but several different chemokines such that a threshold must be reached before discernable changes in cell migration could be detected. This "barcode" of chemokines has yet to be determined for macrophages in the context of ALD. Thirdly, an early surge followed by a decline of F4/80+ macrophages has been well documented for a wide range of organ injuries. We did not profile the dynamic changes in macrophage population in the liver during the entire course of ALD pathogenesis using the NIAAA protocol but instead focused on the end-point for analysis. Therefore, the possibility that Brg1 may contribute to regulate macrophage infiltration at earlier points during ALD development can not be conclusively excluded. Lastly, whole liver tissue was used for RNA extraction and qPCR analysis. Therefore, the data presented in figure 2E should be interpreted as overall inflammation in the liver instead of "chemoattractive prowess" emitting from hepatocytes. After all, many of these cytokines/chemokines can be produced by neutrophils (PMID: 25374568, 29772063). Down-regulation of these cytokines/chemokines may be interpreted as deficient neutrophil recruitment. The reviewer is absolutely right that the role of Brg1 in regulating immune cell trafficking in the context of ALD pathogenesis deserves more attention and we hope to address these lingering issues in future studies. We have expanded the Discussion section to reflect on this point.

Ans: Thanks for the authors to address my concerns. However, the issues remain unresolved. It is well documented that elevated inflammatory cytokines (IL1b and TNFa) could lead to macrophage infiltration. A review article also mentioned the role of CXCL14 in promoting macrophage infiltration (PMC4700668). Based on the data you presented, it is expected that macrophage numbers should be decreased in the Brg1 liver specific KO (Brg1 LKO) mice. However, you did not observe the difference in macrophage numbers between control and Brg1 LKO mice. You should carefully and reasonably discuss the issue with a possible hypothesis (or a potential molecular mechanism) that can explain the discrepancy. 1. The "barcode" explanation is not possible since you have observed macrophage infiltration in all mice and found decreased IL1b and TNFa in Brg1 LKO mice. 2. Both control and Brg1 LKO mice challenged with the NIAAA model are with liver injury. How could macrophage surge not occur in control mice?

4.The neutrophil co-culture experiments were performed using mouse hepatocytes. Can the authors repeat the experiments using human hepatocytes to ensure human relevance? The stained neutrophils look like the background of transwell pores. You may want to replace the representative image with a suitable one. In the meantime, can the authors verify if the knockdown of Brg1 or CXCL14 will not cause the infiltration of other immune cell types to support their conclusion?
Response: We thank the reviewer for his/her suggestion.
(1) We have since performed additional Boyden chamber trans-well experiments using HepaRG cells, considered ideal for investigating liver metabolism in lieu of primary human hepatocytes (PMID: 20645056). As shown in the new appendix supplementary figure 8, figure 17, and figure 18, Brg1 knockdown, or Brg1 inhibition, or CXCL14 antagonism attenuated neutrophil migration.
(2) We respectfully refer the reviewer to the appendix supplementary figure 7 showing that Brg1 deficiency did not influence the infiltration of either macrophages or T lymphocytes. We have also performed additional IHC stainings to assess the effect of CXCL14 on macrophage/T cell infiltration in the liver. As shown in the new appendix supplementary figure 16, CXCL14 knockdown did not alter the infiltration of macrophages or T lymphocytes.

Ans: Thanks for your clarification. The reviewer evaluated supplementary Fig. S7 and S16. It was noted that the CD3 and F4/80 positive areas are higher in Brg1 deficient mice than the control ones based on the representative images. These findings contradict to decreased inflammatory cytokines found in the Brg1 LKO mice.

Referee #2 (Remarks for Author):

The authors satisfactorily addressed the questions and concerns and significantly improved the manuscript.

Reviewer's comments
Referee #1 (Remarks for Author):

1. Thanks for the authors to address my concerns. However, the issues remain unresolved. It is well documented that elevated inflammatory cytokines (IL1b and TNFa) could lead to macrophage infiltration. A review article also mentioned the role of CXCL14 in promoting macrophage infiltration (PMC4700668). Based on the data you presented, it is expected that macrophage numbers should be decreased in the Brg1 liver specific KO (Brg1 LKO) mice. However, you did not observe the difference in macrophage numbers between control and Brg1 LKO mice. You should carefully and reasonably discuss the issue with a possible hypothesis (or a potential molecular mechanism) that can explain the discrepancy. 1. The "barcode" explanation is not possible since you have observed macrophage infiltration in all mice and found decreased IL1b and TNFa in Brg1 LKO mice. 2. Both control and Brg1 LKO mice challenged with the NIAAA model are with liver injury. How could macrophage surge not occur in control mice?

Response: We appreciate the reviewer's concern but respectfully disagree with his/her reasoning and we would like to explain why.

(1) We have carefully read the review article referred to by the reviewer (PMC4700668) and come to the conclusion that the literature, as summarized in this review, supports a role for CXCL14 in the chemotaxis of B cells, monocytes, neutrophils, dendritic cells, and NK cells but not that of macrophages or T cells, which is consistent with our observation.

(2) We agree with the reviewer that some of the cytokines, including IL-1β and TNF-α, have a demonstrated role in promoting macrophage trafficking. As explained in our rebuttal during the last round of revision, several possible scenarios may account for this apparent discrepancy. We stand by our proposals.

2. Thanks for your clarification. The reviewer evaluated supplementary Fig. S7 and S16. It was noted that the CD3 and F4/80 positive areas are higher in Brg1 deficient mice than the control ones based on the representative images. These findings contradict to decreased inflammatory cytokines found in the Brg1 LKO mice.

Response: We appreciate the reviewer's concern. The histological stainings were evaluated and quantified by two pathologists independently. We stand by our observations. Please see our response to comment #1.

Referee #2 (Remarks for Author):

The authors satisfactorily addressed the questions and concerns and significantly improved the manuscript.

Response: We are happy to hear that the reviewer is satisfied with our revision. We are also gratified for his/her endorsement.

We are pleased to inform you that your manuscript is accepted for publication and is now being sent to our publisher to be included in the next available issue of EMBO Molecular Medicine.

EMBO Press Author Checklist

Corresponding Author Name: YONG XU	
Journal Submitted to: EMBO Mol Med	
Manuscript Number: EMM-2022-16592V3	

USEFUL LINKS FOR COMPLETING THIS FORM
The EMBO Journal - Author Guidelines
EMBO Reports - Author Guidelines
Molecular Systems Biology - Author Guidelines
EMBO Molecular Medicine - Author Guidelines

Reporting Checklist for Life Science Articles (updated January 2022)
This checklist is adapted from Materials Design Analysis Reporting (MDAR) Checklist for Authors. MDAR establishes a minimum set of requirements in transparent reporting in the life sciences (see Statement of Task: 10.31222/osf.io/9sm4x). Please follow the journal's guidelines in preparing your manuscript.
Please note that a copy of this checklist will be published alongside your article.

Abridged guidelines for figures
1. Data
The data shown in figures should satisfy the following conditions:
- the data were obtained and processed according to the field's best practice and are presented to reflect the results of the experiments in an accurate and unbiased manner.
- ideally, figure panels should include only measurements that are directly comparable to each other and obtained with the same assay.
- plots include clearly labeled error bars for independent experiments and sample sizes. Unless justified, error bars should not be shown for technical replicates.
- if n<5, the individual data points from each experiment should be plotted. Any statistical test employed should be justified.
- Source Data should be included to report the data underlying figures according to the guidelines set out in the authorship guidelines on Data Presentation.

2. Captions
Each figure caption should contain the following information, for each panel where they are relevant:
- a specification of the experimental system investigated (eg cell line, species name).
- the assay(s) and method(s) used to carry out the reported observations and measurements.
- an explicit mention of the biological and chemical entity(ies) that are being measured.
- an explicit mention of the biological and chemical entity(ies) that are altered/varied/perturbed in a controlled manner.
- the exact sample size (n) for each experimental group/condition, given as a number, not a range;
- a description of the sample collection allowing the reader to understand whether the samples represent technical or biological replicates (including how many animals, litters, cultures, etc.).
- a statement of how many times the experiment shown was independently replicated in the laboratory.
- definitions of statistical methods and measures:
  - common tests, such as t-test (please specify whether paired vs. unpaired), simple χ2 tests, Wilcoxon and Mann-Whitney tests, can be unambiguously identified by name only, but more complex techniques should be described in the methods section;
  - are tests one-sided or two-sided?
  - are there adjustments for multiple comparisons?
  - exact statistical test results, e.g., P values = x but not P values < x;
  - definition of 'center values' as median or average;
  - definition of error bars as s.d. or s.e.m.

Please complete ALL of the questions below.
Select "Not Applicable" only when the requested information is not relevant for your study.

Materials

Newly Created Materials	Information included in the manuscript?	In which section is the information available? (Reagents and Tools Table, Materials and Methods, Figures, Data Availability Section)
New materials and reagents need to be available; do any restrictions apply?	Not Applicable	

Antibodies	Information included in the manuscript?	In which section is the information available? (Reagents and Tools Table, Materials and Methods, Figures, Data Availability Section)
For **antibodies** provide the following information:<br>- Commercial antibodies: RRID (if possible) or supplier name, catalogue number and or/clone number<br>- Non-commercial: RRID or citation	Yes	Materials and Methods

DNA and RNA sequences	Information included in the manuscript?	In which section is the information available? (Reagents and Tools Table, Materials and Methods, Figures, Data Availability Section)
**Short novel DNA or RNA including primers, probes**: provide the sequences.	Yes	Materials and Methods

Cell materials	Information included in the manuscript?	In which section is the information available? (Reagents and Tools Table, Materials and Methods, Figures, Data Availability Section)
**Cell lines:** Provide species information, strain. Provide accession number in repository **OR** supplier name, catalog number, clone number, and/**OR** RRID.	Yes	Materials and Methods
**Primary cultures:** Provide species, strain, sex of origin, genetic modification status.	Yes	Materials and Methods
Report if the cell lines were recently **authenticated** (e.g., by STR profiling) and tested for mycoplasma contamination.	Not Applicable	

Experimental animals	Information included in the manuscript?	In which section is the information available? (Reagents and Tools Table, Materials and Methods, Figures, Data Availability Section)
**Laboratory animals or Model organisms:** Provide species, strain, sex, age, genetic modification status. Provide accession number in repository **OR** supplier name, catalog number, clone number, **OR** RRID.	Yes	Materials and Methods
**Animal observed in or captured from the field:** Provide species, sex, and age where possible.	Not Applicable	
Please detail **housing and husbandry conditions**.	Yes	Materials and Methods

Plants and microbes	Information included in the manuscript?	In which section is the information available? (Reagents and Tools Table, Materials and Methods, Figures, Data Availability Section)
**Plants:** provide species and strain, ecotype and cultivar where relevant, unique accession number if available, and source (including location for collected wild specimens).	Not Applicable	
**Microbes:** provide species and strain, unique accession number if available, and source.	Not Applicable	

Human research participants	Information included in the manuscript?	In which section is the information available? (Reagents and Tools Table, Materials and Methods, Figures, Data Availability Section)
If collected and within the bounds of privacy constraints report on age, sex and gender or ethnicity for all study participants.	Yes	Materials and Methods

Core facilities	Information included in the manuscript?	In which section is the information available? (Reagents and Tools Table, Materials and Methods, Figures, Data Availability Section)
If your work benefited from core facilities, was their service mentioned in the acknowledgments section?	Not Applicable	

Design

Study protocol	Information included in the manuscript?	In which section is the information available? (Reagents and Tools Table, Materials and Methods, Figures, Data Availability Section)
If study protocol has been **pre-registered, provide DOI in the manuscript**. For clinical trials, provide the trial registration number **OR** cite DOI.	Not Applicable	
Report the **clinical trial registration number** (at ClinicalTrials.gov or equivalent), where applicable.	Not Applicable	

Laboratory protocol	Information included in the manuscript?	In which section is the information available? (Reagents and Tools Table, Materials and Methods, Figures, Data Availability Section)
Provide DOI OR other citation details if **external detailed step-by-step protocols** are available.	Yes	Materials and Methods

Experimental study design and statistics	Information included in the manuscript?	In which section is the information available? (Reagents and Tools Table, Materials and Methods, Figures, Data Availability Section)
Include a statement about **sample size** estimate even if no statistical methods were used.	Yes	Materials and Methods
Were any steps taken to minimize the effects of subjective bias when allocating animals/samples to treatment (e.g. **randomization procedure**)? If yes, have they been described?	Not Applicable	
Include a statement about **blinding** even if no blinding was done.	Yes	Materials and Methods
Describe **inclusion/exclusion criteria** if samples or animals were excluded from the analysis. Were the criteria pre-established?<br><br>If sample or data points were omitted from analysis, report if this was due to attrition or intentional exclusion and provide justification.	Not Applicable	
For every figure, are **statistical tests** justified as appropriate? Do the data meet the assumptions of the tests (e.g., normal distribution)? Describe any methods used to assess it. Is there an estimate of variation within each group of data? Is the variance similar between the groups that are being statistically compared?	Yes	Materials and Methods

Sample definition and in-laboratory replication	Information included in the manuscript?	In which section is the information available? (Reagents and Tools Table, Materials and Methods, Figures, Data Availability Section)
In the figure legends: state number of times the experiment was **replicated** in laboratory.	Yes	Figure legends
In the figure legends: define whether data describe **technical or biological replicates**.	Yes	Figure legends

Ethics

Ethics	Information included in the manuscript?	In which section is the information available? (Reagents and Tools Table, Materials and Methods, Figures, Data Availability Section)
Studies involving **human participants**: State details of **authority granting ethics approval** (IRB or equivalent committee(s), provide reference number for approval.	Yes	Materials and Methods
Studies involving **human participants**: Include a statement confirming that **informed consent** was obtained from all subjects and that the experiments conformed to the principles set out in the WMA Declaration of Helsinki and the Department of Health and Human Services Belmont Report.	Yes	Materials and Methods
Studies involving **human participants:** For publication of **patient photos**, include a statement confirming that consent to publish was obtained.	Not Applicable	
Studies involving experimental **animals**: State details of **authority granting ethics approval** (IRB or equivalent committee(s), provide reference number for approval. Include a statement of compliance with ethical regulations.	Yes	Materials and Methods
Studies involving **specimen and field samples:** State if relevant **permits** obtained, provide details of authority approving study; if none were required, explain why.	Yes	Materials and Methods

Dual Use Research of Concern (DURC)	Information included in the manuscript?	In which section is the information available? (Reagents and Tools Table, Materials and Methods, Figures, Data Availability Section)
Could your study fall under dual use research restrictions? Please check biosecurity documents and list of **select agents and toxins** (CDC): https://www.selectagents.gov/sat/list.htm	Not Applicable	
If you used a select agent, is the security level of the lab appropriate and reported in the manuscript?	Not Applicable	
If a study is subject to dual use research of concern regulations, is the name of the **authority granting approval and reference number** for the regulatory approval provided in the manuscript?	Not Applicable	

Reporting

The MDAR framework recommends adoption of discipline-specific guidelines, established and endorsed through community initiatives. Journals have their own policy about requiring specific guidelines and recommendations to complement MDAR.

Adherence to community standards	Information included in the manuscript?	In which section is the information available? (Reagents and Tools Table, Materials and Methods, Figures, Data Availability Section)
State if relevant guidelines or checklists (e.g., **ICMJE, MIBBI, ARRIVE, PRISMA**) have been followed or provided.	Not Applicable	
For **tumor marker prognostic studies,** we recommend that you follow the **REMARK** reporting guidelines (see link list at top right). See author guidelines, under 'Reporting Guidelines'. Please confirm you have followed these guidelines.	Not Applicable	
For **phase II and III randomized controlled trials**, please refer to the **CONSORT** flow diagram (see link list at top right) and submit the CONSORT checklist (see link list at top right) with your submission. See author guidelines, under 'Reporting Guidelines'. Please confirm you have submitted this list.	Not Applicable	

Data Availability

Data availability	Information included in the manuscript?	In which section is the information available? (Reagents and Tools Table, Materials and Methods, Figures, Data Availability Section)
Have **primary datasets** been deposited according to the journal's guidelines (see 'Data Deposition' section) and the respective accession numbers provided in the Data Availability Section?	Yes	Data Availability Section
Were **human clinical and genomic datasets** deposited in a public access-controlled repository in accordance to ethical obligations to the patients and to the applicable consent agreement?	Not Applicable	
Are **computational models** that are central and integral to a study available without restrictions in a machine-readable form? Were the relevant accession numbers or links provided?	Not Applicable	
If publicly available data were reused, provide the respective **data citations in the reference list**.	Yes	Figure legends
